# General Approach to Silica-Supported Salens and Salophens and Their Use as Catalysts for the Synthesis of Cyclic Carbonates from Epoxides and Carbon Dioxide

**DOI:** 10.1021/acs.joc.2c02104

**Published:** 2022-12-01

**Authors:** Ryan E. Barker, Liping Guo, Claudio J. A. Mota, Michael North, Leonardo P. Ozorio, William Pointer, Sarah Walberton, Xiao Wu

**Affiliations:** † Green Chemistry Centre of Excellence, Department of Chemistry, University of York, York YO10 5DD, U.K.; ‡ Jiaxing Key Laboratory of Molecular Recognition and Sensing, College of Biological, Chemical Sciences and Engineering, Jiaxing University, Jiaxing 314001, China; § Universidade Federal do Rio de Janeiro, Instituto de Química, 21941-909 Rio de Janeiro, Brazil; ∥ Universidade Federal do Rio de Janeiro, Escola de Química, 21941-909, Rio de Janeiro, Brazil; ⊥ INCT Energia & Ambiente, Universidade Federal do Rio de Janeiro, 21941-909, Rio de Janeiro, Brazil

## Abstract

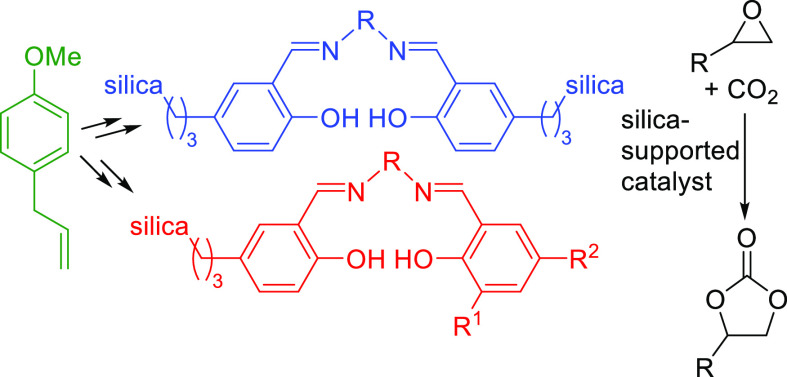

General routes for the synthesis of silica-immobilized symmetrical and unsymmetrical salophen and salen ligands and metal complexes have been developed starting from the natural product 4-allylanisole (methyl-chavicol and estragole). The key step of the syntheses is a microwave-assisted, platinum oxide catalyzed hydrosilylation of the terminal alkene of 5-allyl-2-hydroxybenzaldehyde to afford a sol–gel precursor which can be immobilized into silica before or after conversion to salen and salophen ligands to afford unsymmetrical and symmetrical silica-supported ligands, respectively. Both the symmetrical and unsymmetrical silica-supported salophens were found to catalyze the formation of cyclic carbonates from epoxides and carbon dioxide with catalytic activities at least comparable to those previously reported for non-immobilized homogeneous salophens. This reaction could also be carried out in a multi-phase flow reactor using ethyl acetate solutions of 3-phenoxypropylene oxide. Metal complexes of the silica-immobilized ligands could be prepared, and the aluminum complexes were also found to catalyze cyclic carbonate formation.

## Introduction

**Figure 1:**
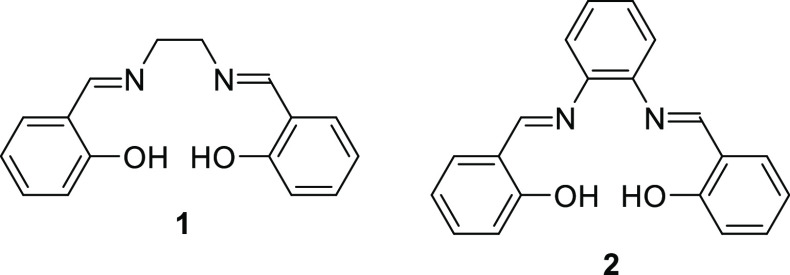
Structures of unfunctionalized salen (**1**) and salophen (**2**).

**Scheme 1:**
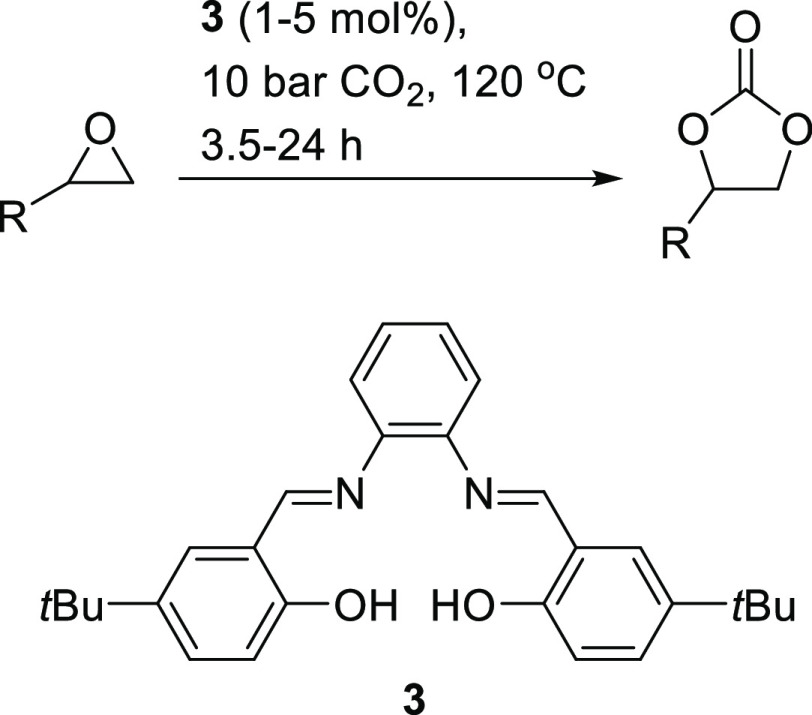
Synthesis of Cyclic Carbonates Catalyzed by Salophen **3** Acting as an Organocatalyst

Salen (**1**) and salophen (**2**) (Figure 1) are considered to be privileged ligands as they will coordinate with just about any metal to afford complexes which catalyze a wide range of reactions.^1^ The steric and electronic properties of these modular ligands can readily be varied to optimize their catalytic activity, and chirality can be introduced into the diamine- and/or aldehyde-derived units to form asymmetric catalysts. Recently, we have shown that even in the absence of a metal, salophens (optimally **3**) were capable of functioning as organocatalysts and catalyzing the formation of cyclic carbonates from epoxides and carbon dioxide (Scheme 1).^2,3^

Despite their enormous popularity as homogeneous catalysts, the immobilization of salens and salophens to provide heterogeneous catalysts, which would benefit from ease of separation/reuse and compatibility with flow reactor technology, has been more problematic. The literature in this area is extensive and has been the subject of a number of reviews.^4^ Various approaches have been investigated, including the synthesis of self-assembling polymeric complexes,^5^ copolymerization of alkene-functionalized salophens with other monomers,^6^ and attachment of salen complexes to the surface of silica,^7,8^ organic polymers,^8,9^ and other supports^10^ through covalent or ionic bonds or through electrostatic interactions. Both the ligand and the metal ion have been used as the attachment site in these methodologies. However, no generally applicable methodology for the synthesis of immobilized salens and salophens and their metal complexes has yet been developed, and the systems that have been developed have stability issues. For example, we developed a methodology to link bimetallic aluminum(salen) complexes to silica supports by the use of linkers containing quaternary ammonium ions. The resulting immobilized complexes catalyzed the synthesis of cyclic carbonates from epoxides and carbon dioxide in both batch and flow reactors but gradually lost their catalytic activity due to leaching of the metal and ligand due to reverse Menshutkin reactions, which caused dealkylation of the quaternary ammonium salts within the linker.^11^

Therefore, we sought to develop a general synthesis of silica-supported salens and salophens both as the free ligands and as metal complexes. The methodology should allow the synthesis of symmetrical and unsymmetrical ligands and use an unfunctionalized alkyl group as the linker. In this paper, we show that this goal can be achieved starting from a biomass-derived precursor. We also show that immobilized ligands and complexes prepared in this way are catalytically active for cyclic carbonate synthesis.

## Results and Discussion

### Synthesis of Silica-Supported Salens and Salophens

**Scheme 2:**
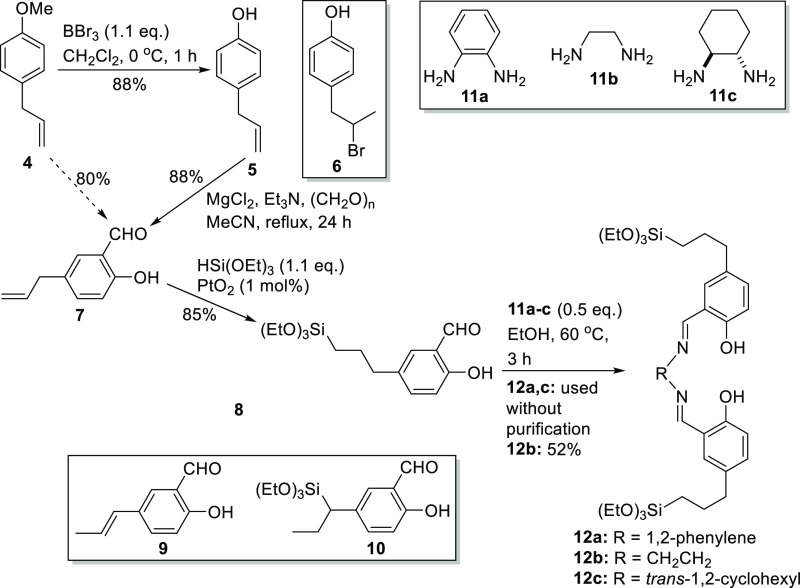
Synthesis of Immobilization Precursors **8** and **12a–c**

4-Allylanisole **4** (also known as methyl-chavicol and estragole) is an inexpensive phenylpropenoid produced naturally by common aromatic plants and herbs, including tarragon, sweet basil, sweet fennel, star anise, and anise vert.^12^ It is commonly used as a food and drink additive and finds applications in the fragrance industry.^13^ The combination of a masked phenol which could be a precursor to a salen/salophen unit and an allyl group which could be used to link to silica also appeared to make 4-allyl anisole an ideal starting material for this project. Thus, conversion of anisole **4** into 4-allylphenol **5** (Scheme 2) was accomplished by a literature procedure^14^ but with a slightly modified work-up (use of aqueous base rather than water) to avoid the formation of the brominated byproduct **6**. Compound **5** produced in this way was pure enough to be used without purification, though it could also be purified by chromatography with 88% isolated yield. Treatment of compound **5** with paraformaldehyde in the presence of triethylamine and magnesium chloride afforded 2-hydroxy-5-allylbenzaldehyde **7** in 88% yield after purification by chromatography.^15^ Compound **7** could also be obtained in 80% yield directly from 4-allylanisole **4** without purification of intermediate **5**.

The key step in the synthesis was the hydrosilylation of compound **7** using triethoxysilane to afford 2-hydroxy-5-(3-triethoxysilylpropyl)benzaldehyde **8**. Initially, this reaction was attempted using tetrakis(triphenylphosphine)rhodium(0) as a homogeneous catalyst,^16^ but this gave a complex reaction mixture in which compound **8** was a minor component. Reasoning that a ligand-free, heterogeneous catalyst would produce fewer contaminants, the use of platinum oxide as a catalyst^17^ was therefore investigated. Under the thermal conditions reported in the literature (80 °C, 20 h), this did afford product **8** in 35% yield after column chromatography along with byproducts **9** and **10**. Compounds **9** and **10** clearly arise from the thermal isomerization of the terminal double bond in compound **7** induced by the lengthy heating at 80 °C. Hence, to optimize the formation of the desired compound **8**, reduction of the reaction temperature and/or time was investigated. This could not be achieved with conventional heating; therefore, as metal oxides are known to absorb microwaves,^18^ microwave heating was investigated using a CEM Discover microwave with a fixed power setting of 80 W. Under these conditions, a reaction carried out at 80 °C for just 10 min produced 100% conversion and 66% of the desired product **8**. Reducing the reaction temperature to 60 °C further inhibited the formation of byproducts **9** and **10**, resulting in the formation of 69% of compound **8** after a reaction time of 1 h. Finally, addition of a catalytic amount of acetic acid to suppress base-catalyzed rearrangements of compound **7** further improved the selectivity of the reaction (at 60 °C for 1 h), giving 85% of compound **8**. Compound **8** could be condensed with half an equivalent of 1,2-diamines **11a–c** to afford symmetrical salens/salophens **12a–c**.

**Scheme 3:**
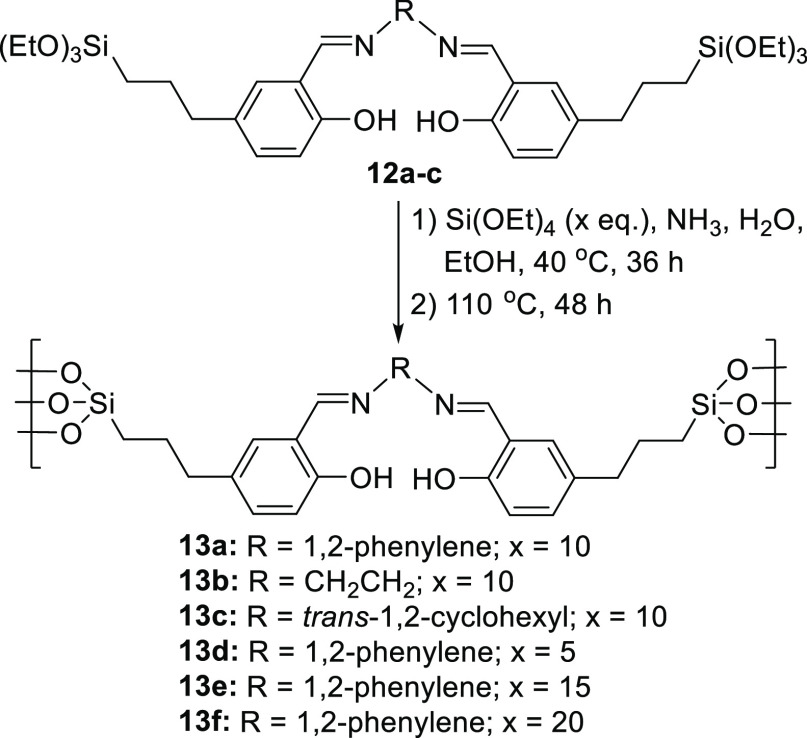
Synthesis of Silica-Supported Symmetrical Salophens and Salens **13a–f**

Compounds **12a–c** and **8** were the key precursors for the synthesis of silica-supported symmetrical and unsymmetrical salens/salophens, respectively, using the sol–gel methodology.^19^ Therefore, compounds **12a–c** were initially treated with 10 equiv of tetraethoxysilane as shown in Scheme 3 to afford silica-supported symmetrical salophen (**13a**) and salens (**13b,c**). Using salophen **12a**, the tetraethoxysilane-to-salophen ratio used also varied, from 5:1 to 20:1, to afford silica-supported symmetrical salophens **13d–f**. These ratios allowed the formation of functionalized silica particles with catalyst loadings that were high enough to analyze and suitable for use in catalytic reactions. Solid-state ^13^C NMR spectra of materials **13a–f** were particularly useful in showing that the salen/salophen unit had survived the sol–gel process while combustion analysis allowed the ratio of salen/salophen units to silica in the materials to be determined.

**Figure 2:**
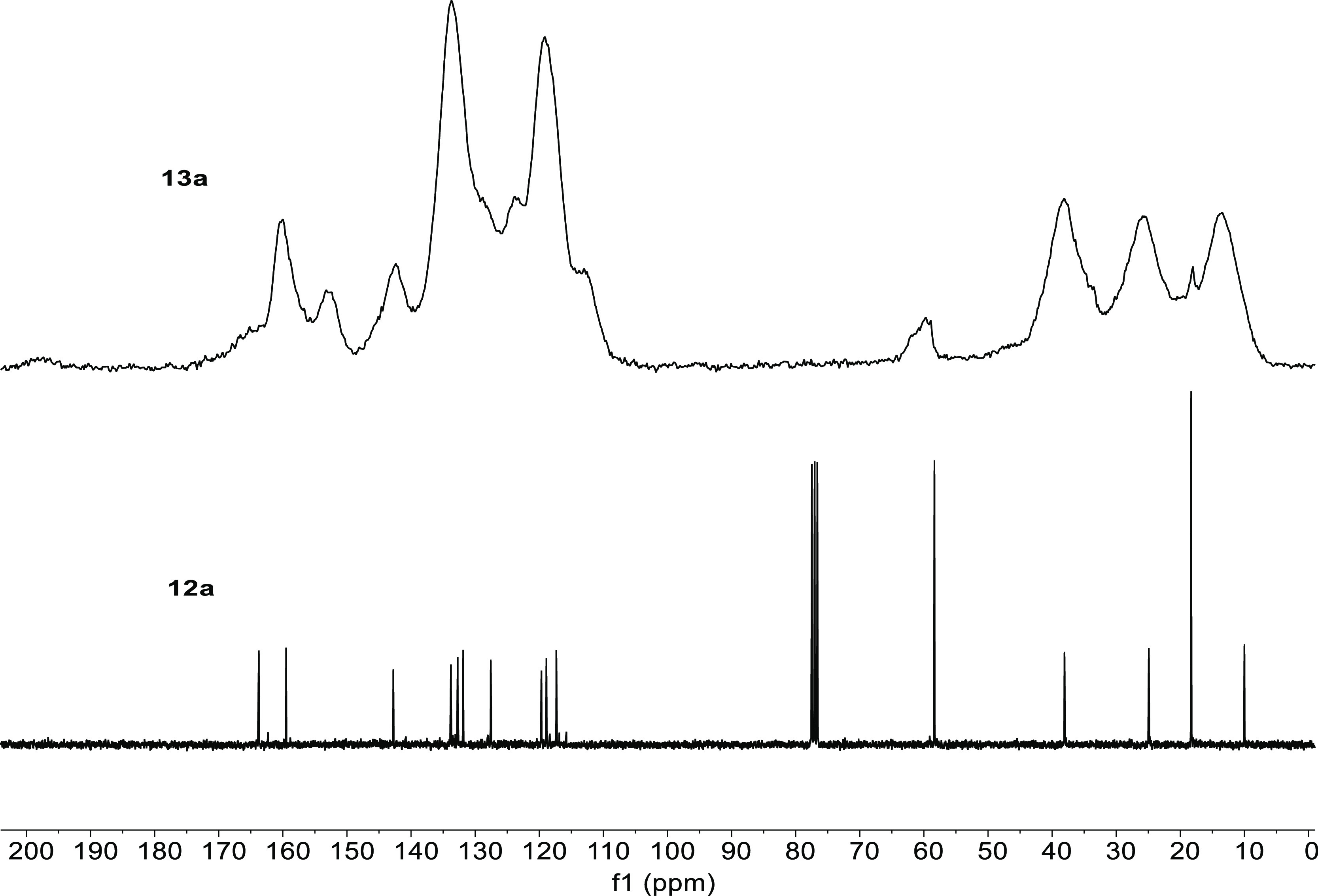
Comparison of the solution-state ^13^C NMR spectrum of **12a** and the solid-state ^13^C NMR spectrum of **13a**.

Figure 2 shows a comparison of the solution-state ^13^C NMR spectrum of salophen **12a** with the solid-state ^13^C NMR spectrum of silica-supported salophen **13a**. Almost all the signals in the spectrum of salophen **12a** map directly onto the signals of silica-supported salophen **13a**, showing that the salophen unit is intact. In particular, the three signals of equal intensity at 10, 25, and 38 ppm corresponding to the three methylene groups linking the triethoxysilyl group to the aromatic ring in the spectrum of **12a** map directly onto the corresponding signals of equal intensity in the spectrum of **13a**. The intense signals at 18 and 58 ppm in the spectrum of **12a** correspond to the six ethoxy groups and are much reduced in intensity in the spectrum of **13a**. This indicates that, as expected, not all of the ethoxy groups have been hydrolyzed during the sol–gel preparation process as some become trapped within the silica matrix. Of the nine aromatic carbons between 117 and 160 ppm in the spectrum of **12a**, only the four signals between 127 and 134 ppm do not map directly onto well-resolved peaks in the solid-state spectrum of **13a**. Three of these signals (those at 132–134 ppm) merge into the most intense signal in the solid-state spectrum, while the peak at 127 ppm in the spectrum of **12a** appears as a shoulder on edge of this peak. Importantly, the signal corresponding to the imine carbon at 164 ppm in the spectrum of **12a** is still clearly present in the solid-state spectrum of **13a**. No spinning side bands are present in the solid-state ^13^C NMR spectrum.

Table 1 details the analytical data obtained for silica-based materials **13a–f** based on a combination of combustion analysis and thermogravimetric analysis (TGA). The combustion analysis data indicated that the ratio of salen/salophen to silica in materials **13a–f** was generally just slightly less than the ratio of **12a–c** to tetraethoxysilane used. Physisorbed water was found to be present in all of materials **13a–f**, and the amount of water needed to fit the empirical formula to the combustion analysis data matched the amount of water determined to be present by TGA.

**Table 1:** Analytical Data for Silica-Supported Salens and Salophens **13a–f**

material	Si(OEt)_4_/**12** ratio used	empirical formula^a^	water in empirical formula (%)^a^	water found by TGA (%)^b^
**13a**	10:1 (12a)	(C_26_H_26_N_2_O_5_Si_2_)(SiO_2_)_11_(H_2_O)_2_	3.0	3.0
**13b**	10:1 (**12b**)	(C_22_H_26_N_2_O_5_Si_2_)(SiO_2_)_10.5_(H_2_O)	1.6	1.5
**13c**	10:1 (**12c**)	(C_26_H_32_N_2_O_5_Si_2_)(SiO_2_)_12.5_(H_2_O)_1.5_	2.1	2.0
**13d**	5:1 (**12a**)	(C_26_H_26_N_2_O_5_Si_2_)(SiO_2_)_7_(H_2_O)_0.6_	1.1	1.1
**13e**	15:1 (**12a**)	(C_26_H_26_N_2_O_5_Si_2_)(SiO_2_)_15_(H_2_O)_1.5_	1.9	1.8
**13f**	20:1 (**12a**)	(C_26_H_26_N_2_O_5_Si_2_)(SiO_2_)_27.5_(H_2_O)_3.5_	2.8	2.7

a Determined by combustion analysis.

b Determined as the weight loss below 100 °C.

**Scheme 4:**
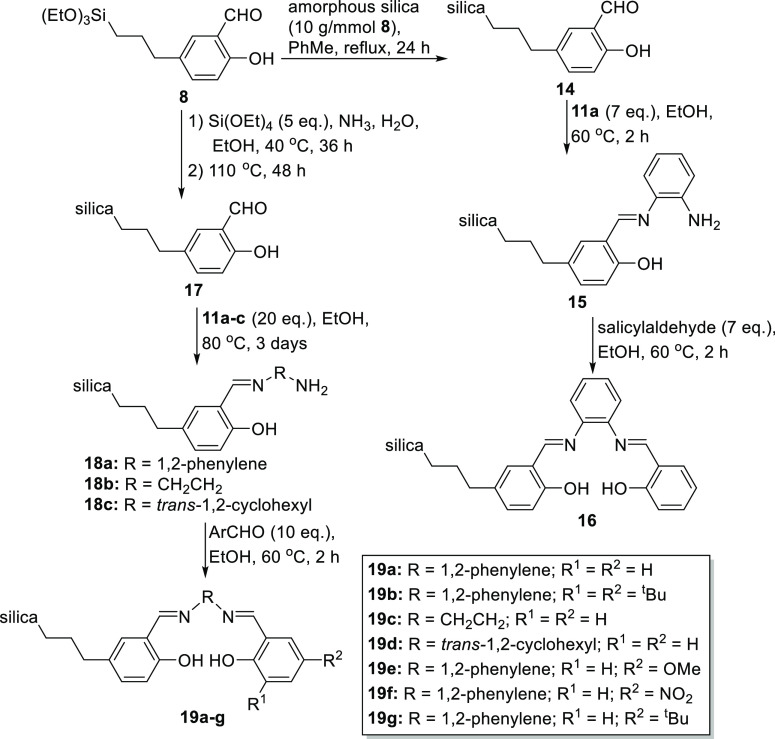
Synthesis of Unsymmetrical Silica-Supported Salophens and Salens from Salicylaldehyde **8**

Sol–gel immobilization of salens/salophens **12a–c** provided a very short and convenient synthesis of symmetrical silica-supported salens and salophens. In contrast, salicylaldehyde **8** could be used to prepare unsymmetrical silica-supported salens and salophens by two routes as shown in Scheme 4. Initially, compound **8** was treated with commercial amorphous silica to form silica-supported salicylaldehyde **14** with a loading of 0.18 mmol g^–1^ as determined by combustion analysis. Subsequent treatment of aldehyde **14** with excess 1,2-diaminobenzene **11a** afforded the silica-supported amine **15** (0.20 mmol g^–1^) as a bright yellow powder. Treatment of aldehyde **14** with excess salicylaldehyde afforded the silica-supported unsymmetrical salophen **16** as a pale yellow solid with a salophen loading of 0.13 mmol g^–1^.

To obtain silica-supported unsymmetrical salens and salophens with higher loadings, a sol–gel route also starting from aldehyde **8** was employed. Thus, aldehyde **8** was reacted with 5 equiv of tetraethoxysilane to afford the silica-supported aldehyde **17**. The solid-state ^13^C NMR spectrum of **17** confirmed that the aldehyde group was still present (198.9 ppm), and combustion analysis indicated a 1:9 ratio of aldehyde to silica. Aldehyde **17** was then reacted with 20 equiv of 1,2-diamines **1a–c** to afford silica-supported amines **18a–c**, which could then be reacted with 10 equiv of salicylaldehyde to afford silica-supported salens and salophens **19a–g**. Notably, this route allowed the steric and electronic properties of the silica-supported salophen to be varied by the introduction of *tert*-butyl (**19b**), methoxy (**19f**), or nitro (**19g**) groups and hence provided a highly versatile route to silica-supported salens and salophens.

### Cyclic Carbonate Synthesis Catalyzed by Silica-Supported Ligands and Their Metal Complexes

**Scheme 5:**
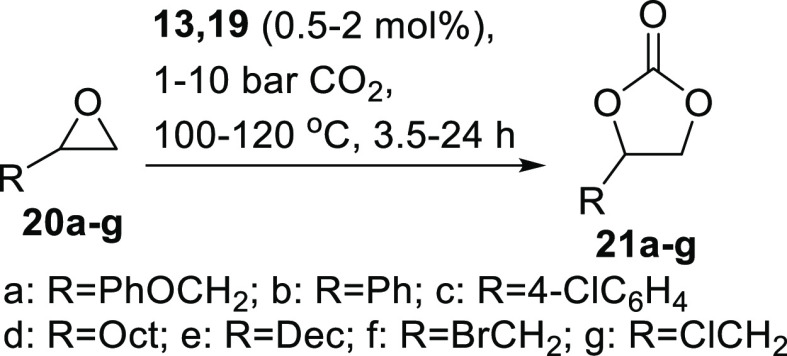
Synthesis of Cyclic Carbonates **21** from Epoxides **20**

Cyclic carbonates are commercially important chemicals^20^ with applications including as components of lithium-ion batteries,^21^ as monomers for the synthesis of poly(hydroxyurethane)s,^22^ and as polar aprotic solvents.^23^ Therefore, and in view of the precedent of using homogeneous salophen ligands as organocatalysts for the synthesis of cyclic carbonates from epoxides and carbon dioxide,^2^ the use of silica-supported salens and salophens **13a–d** and **19a–g** as catalysts for this reaction was investigated. Initial reactions were carried out using symmetrical silica-supported catalysts **13a–c** with 3-phenoxypropylene oxide **20a** as a substrate to form 3-phenoxypropylene carbonate **21a** (Scheme 5) under halide-free conditions previously optimized for the use of homogeneous salophen catalyst **3**.^2^ Results are presented in Table 2.

**Table 2:** Synthesis of Cyclic Carbonate **21a** Using Symmetrical Silica-Supported Catalysts **13a–f**

entry	catalyst [ratio] (mol %)^a^	CO_2_ pressure (bar)	*T* (°C)	time (h)	conversion (%)^b^	yield (%)^c^
1^d^	**3** (1)	10	120	3.5	93	88
2	**13a** [1:11] (1)	10	120	3.5	>99	87
3	**13b** [1:10.5] (1)	10	120	3.5	19	
4	**13c** [1:12.5] (1)	10	120	3.5	11	
5	**13d** [1:7] (1)	10	120	3.5	99	78
6	**13e** [1:15] (1)	10	120	3.5	88	
7	**13f** [1:27] (1)	10	120	3.5	92	
8	**13a** [1:11] (1)	10	100	3.5	40	
9	**13a** [1:11] (0.5)	10	120	3.5	92	
10	**13a** [1:11] (1)	1	120	24	>99	69
11^d^	**3** [1:11] (1)	1	120	24	68	
12	**13a** [1:11] (1)	1	120	7	53	
13	**13a** [1:11] (2)	1	120	7	97	62
14	SiO_2_^e^ (1)	10	120	3.5	2	
15	SiO_2_^e^ (1)	1	120	7	0	

a The incorporated ratio of salen/salophen to silica is given in square brackets, followed by the mol % of homogeneous catalyst **3** or mol % of active sites in silica-supported catalysts **13a–f** in curved brackets.

b Determined by ^1^H NMR spectroscopy of the reaction mixture.

c Isolated yield after chromatographic purification.

d Result taken from ref (2).

e Synthesized from Si(OEt)_4_ by the sol–gel method in the absence of compounds **12a–c**.

Entry 1 of Table 2 shows the previously reported^2^ result obtained using homogeneous salophen catalyst **3**. Entry 2 then shows that remarkably, under the same reaction conditions, silica-supported catalyst **13a** was able to produce a higher conversion and the same isolated chemical yield of cyclic carbonate **21a** as the homogeneous catalyst. Entries 3 and 4 show that silica-supported salen ligands (**13b,c**) are not effective catalysts for this reaction, a result which again matches the previous findings for the corresponding homogeneous ligands.^2^ In view of the high conversion obtained under the initially chosen conditions (Table 2, entry 2), experiments were carried out with catalyst **13a** to see if the reaction would occur under closer to ambient conditions. Reducing the reaction temperature by 20 °C resulted in a large decrease in conversion (Table 2, entry 8), but halving the catalyst loading had only a small effect on the conversion (Table 2, entry 9). When the carbon dioxide pressure was reduced to 1 bar, the reaction became slower but still went to complete conversion with 69% isolated yield of cyclic carbonate **21a** after 24 h (Table 2, entry 10). This was a significant improvement on the 68% conversion previously reported^2^ for the use of homogeneous catalyst **3** under these reaction conditions (Table 2, entry 11). A shorter reaction time of 7 h resulted in a significantly reduced conversion (Table 2, entry 12), but this could be restored to almost complete conversion and 62% isolated yield by doubling the catalyst loading (Table 2, entry 13).

The high catalytic activity of silica-supported salophen **13**, which was comparable to or better than that of the corresponding homogeneous salophen, was unexpected. One possible explanation was that silanol groups within the silica matrix were responsible for some or all of the catalysis as both homogeneous silanols^24^ and unmodified MCM-41 silica^25^ have been reported to catalyze cyclic carbonate synthesis. Therefore, a sample of silica was synthesized by the sol–gel route shown in Scheme 3 but in the absence of any compound **12a–c**. The resulting silica was found to possess very little catalytic activity at 10 bar carbon dioxide pressure (Table 2, entry 14) and none at all at 1 bar carbon dioxide pressure (Table 2, entry 15). Entries 2 and 5–7 of Table 2 show the effect of varying the salophen-to-silica ratio in the catalyst from 1:7 to 1:27 (**13a,d–f**) while keeping the mol % of salophen units used constant at 1 mol % relative to epoxide **20a**. The two highest conversions (Table 2, entries 2 and 5) were obtained with the catalysts which contain the lowest amounts of silica. These results are not consistent with catalysis solely by surface silanol groups within the silica support, but the silanol groups could be involved in synergistic catalysis with the salophen phenols as previously reported for synergistic catalysis by silica and tetraalkylammonium bromides.^26^ Notably, in this respect, the surface silanol groups of silica are much more acidic (p*K*_a_ values of 3.5–6.8 have been reported^27^) than the phenol groups in salophen **3**, which have p*K*_a_’s of 8–9.^28^

**Figure 3:**
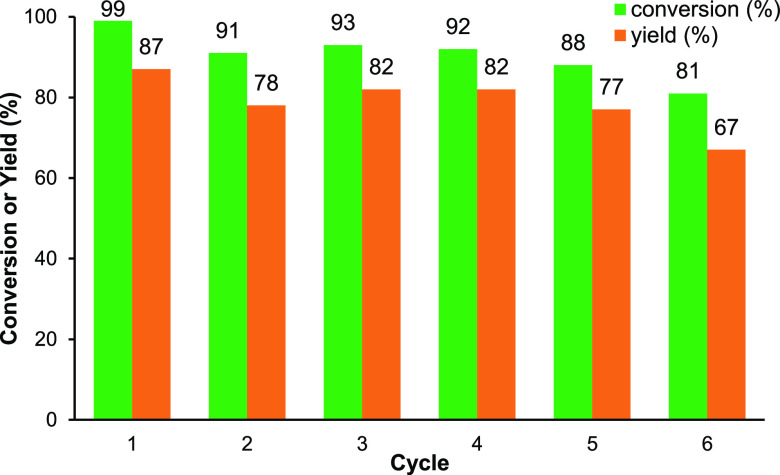
Reuse of silica-supported catalyst **13a** in the synthesis of cyclic carbonate **21a**.

Another possible explanation for the high catalytic activity of silica-supported salophen **13a** was catalyst leaching so that some or all of the catalysis was being carried out homogeneously. To investigate this, a catalyst recycling study was carried out under the conditions of Table 1, entry 2. At the end of each cycle, catalyst **13a** was isolated, washed, analyzed by Fourier-transform infrared (FT-IR) spectroscopy, and reused for a total of six cycles. Both the conversion to cyclic carbonate **21a** and the isolated yield of cyclic carbonate **21a** after chromatographic purification were obtained for each cycle, and the results are summarized in Figure 3. There is an 8–9% drop in catalyst activity between cycles 1 and 2, and then the conversions and yields remain approximately constant at 90 ± 3 and 80 ± 3%, respectively, through cycles 2–5, before another drop of 7–10% in cycle 6. The FT-IR spectra of catalyst **13a** showed no significant changes over the six cycles, and the ^1^H NMR spectra of the reaction mixture showed no evidence of fragments derived from the salophen ligand. These results suggest that silica-supported catalyst **13a** is a highly active, recyclable, heterogeneous catalyst and that no catalyst leaching occurs under the reaction conditions. In addition to its use in batch reactions, silica-supported salophen **13a** could also be used to catalyze the synthesis of cyclic carbonate **21a** from epoxide **20a** in a flow reactor as detailed in the Supporting Information.

The conversion of five other epoxides (**20b–f**) into cyclic carbonates **21b–f** was also studied using silica-supported catalyst **13d**, and the results along with those previously reported^2^ using homogeneous catalyst **3** are shown in Table 3. Styrene oxide (**20b**) was a slow reacting substrate, requiring a reaction time of 24 h to achieve complete conversion to styrene carbonate (**21b**) (Table 1, entry 3) compared to the 3.5 h needed for full reaction of epoxide **20a** (Table 3, entries 1 and 2). However, silica-supported catalyst **13d** was far more effective than homogeneous catalyst **3** (Table 1, entry 4) as the silica-supported catalyst gave double the conversion while using one-fifth the amount of catalyst and allowed styrene carbonate **21b** to be isolated in 95% yield. When 4-chlorostyrene oxide **20c** was used as a substrate (Table 1, entries 5 and 6), 1 mol % of silica-supported catalyst **13d** achieved 100% conversion and produced cyclic carbonate **21c** in 89% yield after a reaction time of just 3 h (Table 3, entry 5). In contrast, even 5 mol % of homogeneous catalyst **3** required a reaction time of 24 h to produce 96 conversion of epoxide **20c**, giving cyclic carbonate **21c** in 86% yield (Table 3, entry 6). Aliphatic epoxides **20d** and **20e** gave very similar results: both were slow reacting substrates which required a reaction time of 48 h to achieve full conversion with catalyst **13d**, giving cyclic carbonates **21d** and **21e** in isolated yields of 78 and 88%, respectively (Table 3, entries 7–9 and 11–13). Homogeneous catalyst **3** had been reported to achieve 71% conversion of epoxide **20d** and 100% conversion of epoxide **20e** after a reaction time of 24 h, but this was achieved by using five times as much catalyst (Table 3, entries 10 and 14). Finally, 3-bromopropylene oxide **20f** was a very reactive substrate for catalyst **20f**, producing complete conversion after 3.5 h and allowing 3-bromopropylene carbonate **21f** to be isolated in 58% yield. Overall, silica-supported catalysts **13** exhibited high activity for cyclic carbonate synthesis under comparable reaction conditions to those required by other metal- and halide-free, homogeneous,^29^ and purely organic polymeric catalysts.^30^ However, the modular nature of the salophen unit allows the catalyst structure to be readily varied and optimized, while the silica support is sufficiently robust to facilitate use in a flow reactor. The heterogeneous nature of silica-supported catalysts **13** enabled their facile separation from the cyclic carbonate product and reuse.

**Table 3:** Synthesis of Cyclic Carbonates **21a–f** Using Silica-Supported Catalyst **13d**^a^

entry	epoxide	catalyst [ratio] (mol %)^b^	time (h)	conversion (%)^c^	yield (%)^d^
1	**20a** (R = PhOCH_2_)	**13d** [1:7] (1)	3.5	99	78
2^e^	**20a** (R = PhOCH_2_)	**3** (1)	3.5	93	88
3	**20b** (R = Ph)	**13d** [1:7] (1)	24	100	95
4^e^	**20b** (R = Ph)	**3** (5)	24	50	
5	**20c** (R = 4-ClC_6_H_4_)	**13d** [1:7] (1)	3	100	89
6^e^	**20c** (R = 4-ClC_6_H_4_)	**3** (5)	24	96	86
7	**20d** (R = Oct)	**13d** [1:7] (1)	3.5	19	
8	**20d** (R = Oct)	**13d** [1:7] (1)	24	40	
9	**20d** (R = Oct)	**13d** [1:7] (1)	48	100	78
10^e^	**20d** (R = Oct)	**3** (5)	24	71	51
11	**20e** (R = Dec)	**13d** [1:7] (1)	3.5	12	
12	**20e** (R = Dec)	**13d** [1:7] (1)	24	40	
13	**20e** (R = Dec)	**13d** [1:7] (1)	48	100	88
14^e^	**20e** (R = Dec)	**3** (5)	24	100	89
15	**20f** (R = BrCH_2_)	**13d** [1:7] (1)	3.5	100	58

a All reactions were carried out at 120 °C and 10 bar carbon dioxide pressure.

b The incorporated ratio of salen/salophen to silica is given in square brackets, followed by the mol % of homogeneous catalyst **3** or mol % of active sites in silica-supported catalyst **13d** in curved brackets.

c Conversion to cyclic carbonate determined by ^1^H NMR spectroscopy of the reaction mixture.

d Isolated yield of cyclic carbonate after chromatographic purification.

e Result taken from ref (2).

The use of unsymmetrical silica-supported salophens **19a,e–g** as catalysts for the synthesis of cyclic carbonates **21a–c,e,g** from epoxides **20a–c,e,g** and carbon dioxide was also investigated. These reactions were all carried out at 1 bar carbon dioxide pressure for 24 h using 1 mol % of salophen units within the catalysts, which all had similar loadings of salophen units (0.66 ± 0.1 mmol g^–1^). The reaction temperature (100 or 120 °C) was chosen for each epoxide to produce a moderate conversion (40–60%) with the first catalyst tested. This allowed the four catalysts to be ranked in order of their catalytic activity for each epoxide and hence allowed the steric and electronic effect of substituents on just one of the salophen aromatic rings to be investigated. This type of study is not possible using homogeneous salophens due to difficulties in preparing ligands derived from two different aldehydes and to solubility differences between differently functionalized salophens.

The results of this study are summarized in Table 4 and show that there is no catalyst that is the best for all five substrates. Epoxides **20a,g** gave similar results, with silica-supported catalyst **19a** producing the highest conversions (and isolated yields) of cyclic carbonates **21a,g** while the other three catalysts all produced conversions of 52 ± 2% for epoxide **20a** and 58.5 ± 3.5% for epoxide **20g**. Notably, epoxides **20a,g** were the two most reactive epoxides for homogeneous salophen catalyst **3**,^2^ which may explain their relative insensitivity toward electronic effects within the catalyst. The other three substrates (**20b,c**, and **e**) all gave similar results, though their trend was different to that of epoxides **20a** and **g**. For these substrates, catalyst **19e** with a very electron-donating methoxy substituent always produced the highest conversions, while catalyst **19f** with a very electron-withdrawing nitro substituent was almost inactive with epoxides **20b,e** and the equal worst catalyst for substrate **20c**.

**Table 4:**
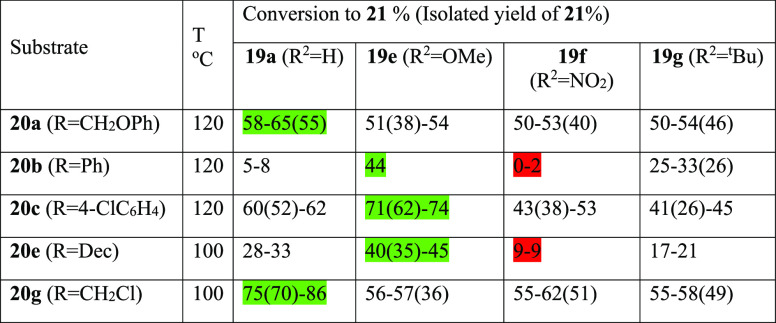
Synthesis of Cyclic Carbonates **21a–g** Using Unsymmetrical Silica-Supported Catalysts **19a,e–g**^a^

a Green shading shows the best catalyst for each substrate. Red shading shows the worst catalyst for each substrate, where one catalyst was clearly worse than the others.

These results are clearly not compatible with a mechanism in which the phenol attached to the same aromatic ring as the substituent acts as a Brønsted acid to activate the epoxide as a nitro substituent would afford the most acidic phenol. The results are however compatible with this phenol converting the carbon dioxide into a carbonic acid as this requires the phenol to act as a nucleophile which will be facilitated by an electron-donating *para*-methoxy substituent. The epoxide would then be activated by surface silanol groups possibly acting synergistically with the other phenol as discussed above.

### Silica-Supported Salen and Salophen Metal Complexes

**Scheme 6:**
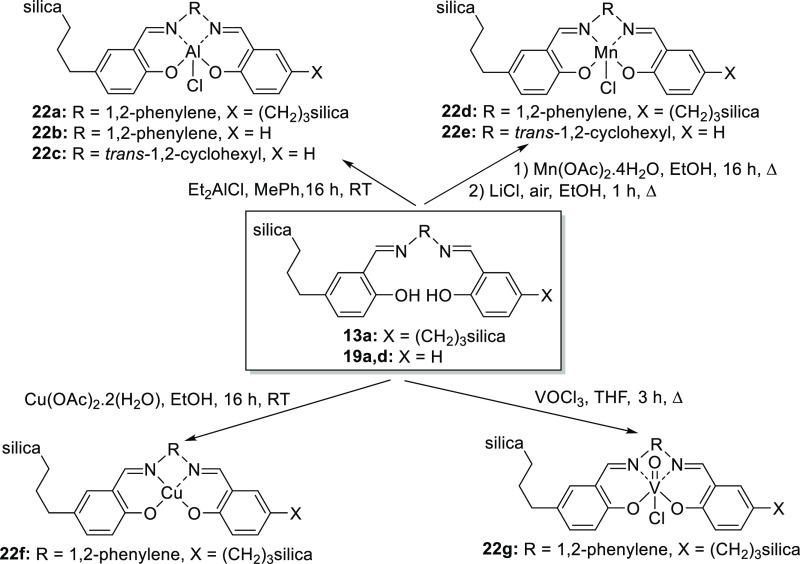
Synthesis of Silica-Supported Metal Complexes **22a–g**

Metal salen and salophen complexes are known to catalyze a wide range of reactions,^1^ so the formation of metal complexes from silica-supported ligands **13a** and **19a,d** was investigated. Treatment of immobilized ligands **13a** and **19a,d** with diethylaluminum chloride afforded aluminum complexes **22a–c**, while reaction of ligands **13a** and **19d** with manganese(II) acetate followed by oxidation and counterion exchange afforded manganese(III) complexes **22d,e** as shown in Scheme 6. Ligand **13a** was also reacted with copper(II) acetate to afford copper(II) complex **22f** and with vanadium(V) oxychloride to afford vanadium(V) complex **22g**. Between them, complexes **22a–g** include examples of salen/salophen complexes for which soluble analogues are known to be four-,^31^ five-,^32^ and six-coordinate.^33^

The incorporation of metals into complexes **22a–g** was confirmed by X-ray photoelectron spectroscopy (XPS) and inductively coupled plasma–optical emission spectrometry (ICP–OES). For aluminum and manganese(III) complexes **22a–e**, ICP–OES indicated aluminum contents of 0.1–1.0 mmol g^–1^, while the calculated values based on the silica–salophen ratios found for precursors **13a** and **19a,d** were 0.8–0.9 mmol g^–1^. XPS analysis of complexes **22a,d** confirmed the presence of both metal and chlorine on the surface of silica. For complex **22a**, a solid-state ^13^C NMR spectrum confirmed that the salophen unit was intact and showed small changes in chemical shift compared to non-metallated precursor **13a**. ICP–OES analysis of copper complex **22f** indicated that it contained 1.0 mmol g^–1^ of copper, which compares well with the calculated value of 0.8 mmol g^–1^. The presence of copper on the surface of complex **22f** was further supported by XPS analysis. Vanadium(V) complex **22g** could only be analyzed by XPS, which showed the presence of both vanadium (0.9%) and chlorine (2.3%) on the surface of the silica particles. The chlorine analysis agrees well with the 2.8% predicted chlorine content based on the silica-to-salophen ratio in precursor **13a**.

**Scheme 7:**
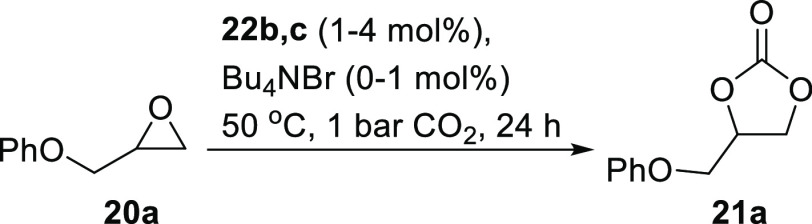
Synthesis of Cyclic Carbonate **21a** Using Silica-Supported Aluminum Complexes **22b,c**

Since salen and salophen complexes of aluminum are known to catalyze the formation of cyclic carbonates from epoxides and carbon dioxide,^11,34^ as well as related reactions,^35^ the use of silica-supported complexes **22b,c** as catalysts for the synthesis of cyclic carbonate **21a** was investigated (Scheme 7). These reactions were carried out at 50 °C and 1 bar carbon dioxide pressure for 24 h, and the results are presented in Table 5. In the absence of a cocatalyst, neither silica-supported complex was catalytically active (Table 5, entries 1 and 2), a result which mirrors the reactivity found for other aluminum(salen) complexes.^11,34^ However, in the presence of tetrabutylammonium bromide (1 mol %), both silica-supported complexes were catalytically active, and the catalytic activity increased as the amount of complex **22b,c** used increased (Table 5, entries 3–6).

**Table 5:** Synthesis of Cyclic Carbonate **21a** Using Silica-Supported Aluminum Complexes **22b,c**

entry	catalyst (mol %)^a^	Bu_4_NBr (mol %)	conversion (%)
1	**22b** (1)	0	0
2	**22c** (1)	0	0
3	**22b** (1)	1	35
4	**22c** (1)	1	30
5	**22b** (2)	1	43
6	**22c** (4)	1	44

a mol % of aluminum in the silica-supported catalyst.

## Conclusions

A general and versatile route for the synthesis of silica-supported salens and salophens and their metal complexes starting from bio-derived 4-allylanisole has been developed. The key step is a platinum oxide catalyzed hydrosilylation of 2-hydroxy-5-allylbenzaldehyde. This reaction was significantly optimized by the use of microwave irradiation and acidic reaction conditions. The resulting 2-hydroxy-5-(3-triethoxysilylpropyl)benzaldehyde is the central intermediate in the synthesis as it could be converted to symmetrical salens and salophens followed by sol–gel immobilization to afford symmetrical silica-immobilized salens and salophens. Alternatively, it could be directly immobilized using sol–gel chemistry and then unsymmetrical silica-immobilized salens and salophens constructed by solid-phase synthesis. Silica-supported metal complexes could then be prepared from the silica-supported ligands.

Silica-supported salophens were found to be highly active organocatalysts for the synthesis of cyclic carbonates from terminal epoxides and carbon dioxide, both in batch reactions and in a multi-phase flow reactor. Remarkably, the silica-supported salophens had similar or higher catalytic activity to the corresponding homogeneous catalysts. This is probably due to the silica-immobilized catalysts benefiting from synergistic catalysis involving both the phenol groups of the salophen and silanol groups from the silica support. Results with unsymmetrical silica-supported salophens indicated that the salophen phenol most distal from the silica support interacts with the carbon dioxide, while the phenol closest to the support and/or silanol units activates the epoxide by hydrogen bonding. In the presence of tetrabutylammonium bromide as a cocatalyst, aluminum complexes of both silica-supported salens and salophens were also shown to be active catalysts for cyclic carbonate synthesis.

The methodology developed in this work is clearly applicable to a wide range of salens and salophens and their metal complexes, allowing it to be used to prepare silica-immobilized catalysts for a wide range of reactions known to be catalyzed by salen and salophen derivatives.^1^ The recyclable nature of these catalysts when used in batch reactions and their compatibility with flow reactor technology will further enhance the utility of salen and salophen ligands in the synthesis.

## Experimental Section

Thermally heated reactions were heated using a stirrer hot plate with a DrySyn attachment. Microwave-heated reactions were carried out in a CEM Discover microwave using sealed vials. The reaction temperature was monitored, and temperature/time profiles were produced using the integral infrared temperature sensor mounted underneath the reactor vessel. Solution-state NMR spectra were recorded in CDCl_3_ on spectrometers operating at 400 or 300 MHz for ^1^H and 100 or 75 MHz for ^13^C. Solid-state ^13^C NMR spectra were recorded on a spectrometer operating at 100 MHz with cross-polarization and magic angle spinning at 10,000–12,000 Hz. High-resolution mass spectra were obtained using electrospray ionization operating in the positive-ion mode unless specified otherwise. Infrared spectra were recorded on undiluted materials using an attenuated total reflection attachment. TGA was carried out by heating the sample under a stream of nitrogen from ambient temperature to 500 °C with a heating rate of 10 °C min^–1^. XPS analysis was performed by the UK national XPS service at the University of Cardiff using a monochromated Al Kα X-ray source. Data were collected at a pass energy of 2150 eV for survey spectra and 40 eV for high-resolution scans. The spectra were collected at a pressure below 10^–7^ Torr and a temperature of 294 K. Nitrogen adsorption–desorption isotherms were measured on a volumetric adsorption analyzer at 77 K. Before analysis, powdered samples were degassed at 120 °C for 16 h. The BET surface area was calculated from the nitrogen adsorption data at a relative pressure range of 0.01–0.2; the total pore volume was estimated at a relative pressure of 0.99. Samples for ICP–OES analysis were digested with concentrated nitric acid and then diluted to afford a 5% nitric acid solution, which was analyzed using argon as the purge, plasma, and sheath gas.

### 4-Allylphenol (**5**)^14^

A solution of 4-allylanisole **4** (2.2 g, 15.0 mmol) in CH_2_Cl_2_ (14.5 mL) was cooled to 0 °C. Then, a solution of BBr_3_ (16.5 mL, 1 M in CH_2_Cl_2_, 1.1 equiv) was added dropwise. The reaction mixture was stirred at 0 °C for 1 h and then added dropwise to a 2 M solution of NaOH in H_2_O (100 mL). The resulting mixture was then neutralized with 1 M HCl solution. The product was extracted with CH_2_Cl_2_ (2 × 30 mL), washed with H_2_O (2 × 20 mL) and brine (20 mL), and dried (MgSO_4_). The solvent was removed in vacuo to afford 4-allylphenol **5**, which was pure enough to be used. The product could also be purified by silica gel column chromatography eluting with hexane/EtOAc (9/1) to afford 4-allylphenol **5** (1.8 g, 88%) as a yellow oil. *R*_F_ (9/1 hexane/EtOAc) 0.3; ν_max_: 3314 (br), 3050, 3004, 2960, 2850, 1610, 1600, 1592 and 1500 cm^–1^; ^1^H NMR (300 MHz, CDCl_3_): δ 7.08 (2H, d, *J* = 8.3 Hz), 6.80 (2H, dt, *J* = 8.4, 2.1 Hz), 6.1–5.9 (1H, m), 5.09 (1H, dt, *J* = 16.0, 1.7 Hz), 5.08 (1H, dt, *J* = 10.8, 1.6 Hz), 4.70 (1H, br), 3.35 (2H, d, *J* = 6.7 Hz); ^13^C{^1^H} NMR (75 MHz, CDCl_3_): δ 153.8, 137.8, 132.3, 129.7, 115.5, 115.2, 39.3; found (ESI), 135.0803, calcd for C_9_H_11_O [M + H]^+^, 135.0804.

### 2-Hydroxy-5-allylbenzaldehyde (**7**)

4-Allylphenol **5** (1.0 g, 7.7 mmol) was dissolved in MeCN (30 mL). MgCl_2_ (1.9 g, 19.5mmol, 2.5 equiv), Et_3_N (3.2 g, 31.2 mmol, 4 equiv), and paraformaldehyde (2.3 g, 78.0 mol, 10 equiv) were added, and then the reaction mixture was heated to reflux and stirred for 24 h. The reaction was cooled to room temperature and then quenched with aqueous HCl (40 mL, 1 M). The product was extracted with EtOAc (3 × 30 mL), and the combined organic phase was washed with H_2_O (2 × 30 mL) and brine (30 mL) and then dried (MgSO_4_)_._ The solvent was removed in vacuo, and the residue was purified by silica gel column chromatography eluting with petroleum ether/EtOAc (15/1) to afford 2-hydroxy-5-allylbenzaldehyde **7** as a yellow oil (1.1 g, 88%). *R*_F_ (15/1 petroleum ether/EtOAc) 0.3; ν_max_: 3150 (br), 2980, 2840, 1650 and 1590 cm^–1^; ^1^H NMR (300 MHz, CDCl_3_): δ 10.91 (1H, s), 9.89 (1H, s), 7.4–7.3 (2H, m), 6.96 (1H, d, *J* = 9.1 Hz), 5.97 (1H, ddt, *J* = 16.8, 10.2, 6.6 Hz), 5.2–5.1 (1H, m), 5.10 (1H, dq, *J* = 11.0, 1.6 Hz), 3.40 (2H, d, *J* = 6.0 Hz); ^13^C{^1^H} NMR (75 MHz, CDCl_3_): δ 196.5, 160.1, 137.6, 136.8, 133.1, 131.4, 120.4, 117.6, 116.4, 38.9; found (ESI negative ion mode), 161.0616, calcd for C_10_H_9_O_2_ [M – H]^+^, 161.0608.

### 2-Hydroxy-5-(3-triethoxysilylpropyl)benzaldehyde (**8**) Method A (Thermal Heating)

2-Hydroxy-5-allylbenzaldehyde **7** (0.24 g, 1.5 mmol) was combined with HSi(OEt)_3_ (0.28 g, 1.7 mmol, 1.1 equiv) and solid PtO_2_ (3.4 mg, 1.5 × 10^–2^ mmol, 0.01 equiv) and heated to 80 °C with stirring for 20 h in a sealed glass vial. The reaction mixture was then spun in a centrifuge for 5 min to separate the PtO_2_ catalyst, and the remaining liquid was concentrated under vacuum to afford compound **8**, which was pure enough to be used. The product could also be purified by silica gel column chromatography eluting with petroleum ether/EtOAc (9/1) to afford 2-hydroxy-5-(3-triethoxysilylpropyl)benzaldehyde **8** (0.26 g, 54%) as a colorless oil. *R*_F_ (9/1 petroleum ether/EtOAc) 0.3; ν_max_: 3169 (br), 3079, 2901, 2837, 1652, 1624 and 1588 cm^–1^; ^1^H NMR (300 MHz, CDCl_3_): δ 10.85 (1H, s), 9.86 (1H, s), 7.4–7.3 (1H, m), 7.34 (1H, s), 6.91 (1H, d, *J* = 9.1 Hz), 3.81 (6H, q, *J* = 6.0 Hz), 2.63 (2H, t, *J* = 6.0 Hz), 1.73 (2H, pent, *J* = 7.9 Hz), 1.22 (9H, t, *J* = 6.0 Hz), 0.65 (2H, t, *J* = 8.2 Hz); ^13^C{^1^H} NMR (75 MHz, CDCl_3_): δ 196.6, 159.8, 137.5, 133.8, 133.0, 120.4, 117.4, 58.4, 37.8, 24.8, 18.3, 10.0; found (ESI), 349.1445, calcd for C_16_H_26_O_5_SiNa [M + Na]^+^, 349.1442; found (ESI), 327.1625, calcd for C_16_H_27_O_5_Si [M + H]^+^, 327.1622.

### 2-Hydroxy-5-(3-triethoxysilylpropyl) benzaldehyde (**8**) Method B (Acid-Catalyzed Microwave Heating)

2-Hydroxy-5-allylbenzaldehyde **7** (0.24 g, 1.5 mmol) was combined with HSi(OEt)_3_ (0.28 g, 1.7 mmol, 1.1 equiv) and solid PtO_2_ (3.4 mg, 1.5 × 10^–2^ mmol, 0.01 equiv) in a 7 mL glass microwave vial. One drop of acetic acid was added. The mixture was microwaved in a CEM Discover microwave set to 60 °C for 1 h with a power setting of 80 watts. Work-up as above afforded 2-hydroxy-5-(3-triethoxysilylpropyl) benzaldehyde **8** (0.42 g, 85%) as a yellow oil.

### *N*,*N*′-Bis(2-hydroxy-5-(3-triethoxysilylpropyl)benzylidene)-1,2-diaminobenzene (**12a**)

Freshly prepared compound **8** (1.0 g, 3.1 mmol) and 1,2-diaminobenzene **11a** (0.169 g, 0.775 mmol) were dissolved in EtOH (4 mL) and heated to 60 °C for 3 h. The solvent was then removed in vacuo to afford *N*,*N*′-bis(2-hydroxy-5-(3-triethoxysilylpropyl)benzylidene)-1,2-diaminobenzene **12a** as an orange oil, which was pure enough to be used without purification. ν_max_: 2973, 2926, 2883, 1615 and 1574 cm^–1^; ^1^H NMR (300 MHz, CDCl_3_): δ 12.94 (2H, s), 8.70 (2H, s), 7.5–7.2 (8H, m), 7.06 (2H, d, *J* = 9.1 Hz), 3.91 (12H, q, *J* = 7.0 Hz), 2.70 (4H, t, *J* = 7.5 Hz), 1.83 (4H, pentet, *J* = 7.8 Hz), 1.32 (18H, t, *J* = 7.0 Hz), 0.77 (4H, t, *J* = 8.2 Hz); ^13^C{^1^H} NMR (75 MHz, CDCl_3_): δ 163.8, 159.5, 142.8, 133.8, 132.7, 131.9, 127.6, 119.7, 118.9, 117.3, 58.4, 38.1, 24.9, 18.3, 10.0; found (ESI), 725.3673, calcd for C_38_H_57_N_2_O_8_Si_2_ [M + H]^+^, 725.3648, found, 747.3490, calcd for C_38_H_56_N_2_O_8_Si_2_Na [M + Na]^+^, 747.3467.

### *N*,*N*′-Bis(2-hydroxy-5-(3-triethoxysilylpropyl)benzylidene)-1,2-diaminoethane (**12b**)

Freshly prepared compound **8** (0.23 g, 0.70 mmol) and 1,2-diaminoethane **11b** (0.021 g, 0.35 mmol) were dissolved in EtOH (1 mL), resulting in the immediate formation of a yellow precipitate. The mixture was heated to 60 °C for 3 h, then cooled, and centrifuged. The resulting solid was washed with EtOH (3 × 5 mL) and dried at 80 °C in vacuo to afford *N*,*N*′-bis(2-hydroxy-5-(3-triethoxysilylpropyl)benzylidene)-1,2-diaminoethane **12b** (0.12 g, 52%) as a yellow solid. ν_max_: 2974, 2928, 2883, 1628 and 1585 cm^–1^; ^1^H NMR (300 MHz, CDCl_3_): δ 12.98 (2H, s), 8.34 (2H, s), 7.11 (2H, dd, *J* = 8.4, 2.2 Hz), 7.02 (2H, d, *J* = 2.2 Hz), 6.86 (2H, d, *J* = 8.4 Hz), 3.93 (4H s), 3.80 (12H, q, *J* = 7.0 Hz), 2.57 (4H, t, *J* = 7.6 Hz), 1.69 (4H, pent, *J* = 8.0 Hz), 1.22 (18H, t, *J* = 7.0 Hz), 0.65 (4H, t, *J* = 8.3 Hz); ^13^C{^1^H} NMR (75 MHz, CDCl_3_): δ 166.5, 159.0, 132.7, 132.5, 131.1, 118.3, 116.7, 60.0, 58.3, 38.1, 24.9, 18.3, 10.0; found (ESI), 677.3657, calcd for C_34_H_57_N_2_O_8_Si_2_ [M + H]^+^, 677.3648; found, 699.3475, calcd for C_34_H_56_N_2_O_8_Si_2_Na [M + Na]^+^, 699.3467.

### *N*,*N*′-Bis(2-hydroxy-5-(3-triethoxysilylpropyl)benzylidene)-*trans*-1,2-diaminocyclohexane (**12c**)

Freshly prepared compound **8** (0.245 g, 0.75 mmol) and *trans*-1,2-diaminocyclohexane **11c** (0.043 g, 0.375 mmol) were dissolved in EtOH (1 mL) and heated to 60 °C for 3 h. The solvent was then removed in vacuo to afford *N*,*N*′-bis(2-hydroxy-5-(3-triethoxysilylpropyl)benzylidene)-*trans*-1,2-diaminocyclohexane **12c** as a yellow oil, which was pure enough to be used without purification. ν_max_: 2973, 2925, 2881, 2861, 1632 and 1589 cm^–1^; ^1^H NMR (300 MHz, CDCl_3_): δ 13.09 (2H, s), 8.24 (2H, s), 7.06 (2H, dd, *J* = 8.4, 2.2 Hz), 6.95 (2H, d, *J* = 2.2 Hz), 6.80 (2H, d, *J* = 8.4 Hz), 3.79 (12H, q, *J* = 7.0 Hz), 3.4–3.2 (2H, m), 2.51 (4H, t, *J* = 7.6 Hz), 2.0–1.8 (4H, m), 1.8–1.3 (8H, m), 1.20 (18H, t, *J* = 7.0 Hz), 0.63 (4H, t, *J* = 8.2 Hz); ^13^C{^1^H} NMR (75 MHz, CDCl_3_): δ 164.6, 159.0, 132.4, 131.1, 118.3, 116.7, 116.5, 72.8, 58.3, 38.1, 33.2, 24.9, 24.2, 18.3, 10.1; found (ESI), 731.4144, calcd for C_38_H_63_N_2_O_8_Si_2_ [M + H]^+^, 731.4117; found, 753.3967, calcd for C_38_H_62_N_2_O_8_Si_2_Na [M + Na]^+^, 753.3937.

### General Procedure for the Synthesis of Silica-Supported Symmetrical Salens and Salophens (**13a–f**)

A solution of Si(OEt)_4_ (1.875–7.5 mmol, 5–20 equiv) in EtOH (1 mL) was added dropwise with vigorous stirring to an aqueous solution of NH_3_ (25–28% NH_3_, 1 mL). After 3 min, a white suspension formed, and a solution of **12a–c** (0.375 mmol, 1 equiv) in EtOH (1 mL) was added to afford a 0.125 M solution of **12a–c**. The mixture was stirred at 40 °C for 36 h, then heated, opened to air, and undisturbed at 110 °C for 48 h. The resulting powder was washed with EtOH (2 × 10 mL), H_2_O (2 × 10 mL), and EtOAc (2 × 10 mL) and then dried at 80 °C in vacuo for 24 h to afford silica-supported symmetrical salens and salophens **13a–f**.

#### 13a

Obtained as an orange-yellow powder (0.38 g, 85%) from a reaction using **12a** and 10 equiv of Si(OEt)_4_. This reaction could also be carried out on a 4 times higher scale using **12a** (1.09 g, 1.5 mmol) to produce **13a** (1.56 g, 87%). ν_max_: 2940, 2860, 1644, 1614, 1487, and 1055 cm^–1^; ^13^C{^1^H} NMR (100 MHz, solid): δ 160.1, 153.2, 142.4, 134.1, 128.5, 123.7, 119.3, 112.7, 59.8, 38.1, 25.9, 18.2, 13.5; Anal. Calcd for C_26_H_26_N_2_O_5_Si_2_ + 11(SiO_2_) + 2(H_2_O): C, 26.0; H, 2.5; N, 2.3. Found: C, 25.9; H, 2.6; N, 2.2; TGA 3% weight loss below 100 °C; Porosimetry: BET surface area 2.6 m^2^ g^–1^, adsorption pore volume 0.01 cm^3^ g^–1^.

#### 13b

Obtained as a yellow powder (0.27 g, 66%) from a reaction using **12b** and 10 equiv of Si(OEt)_4_. ν_max_: 2932, 2853, 1635, 1492, and 1059 cm^–1^; ^13^C{^1^H} NMR (100 MHz, solid): δ 167.7, 160.4, 133.1, 118.9, 60.9, 59.6, 38.0, 25.6, 18.1, 13.1; Anal. Calcd for C_22_H_26_N_2_O_5_Si_2_ + 10.5(SiO_2_) + H_2_O: C, 23.9; H, 2.6; N, 2.5. Found: C, 23.8; H, 2.7; N, 2.2; TGA 1.5% weight loss below 100 °C; Porosimetry: BET surface area 5.2 m^2^ g^–1^, adsorption pore volume 0.03 cm^3^ g^–1^.

#### 13c

Obtained as a bright yellow powder (0.40 g, 83%) from a reaction using **12c** and 10 equiv of Si(OEt)_4_. ν_max_: 2927, 2856, 1632, 1589, 1494, and 1058 cm^–1^; ^13^C{^1^H} NMR (100 MHz, solid): δ 166.1, 160.4, 132.8, 119.0, 73.8, 59.4, 38.1, 34.2, 25.5, 19.4, 18.0, 13,7; Anal. Calcd for C_26_H_32_N_2_O_5_Si_2_ + 12.5(SiO_2_) + 1.5(H_2_O): C, 24.3; H, 2.7; N, 2.2. Found: C, 24.2; H, 2.9; N, 1.9; TGA 2.0% weight loss below 100 °C; Porosimetry: BET surface area 2.1 m^2^ g^–1^, adsorption pore volume 0.01 cm^3^ g^–1^.

#### 13d

Obtained as a yellow powder (0.30 g, 91%) from a reaction using **12a** (0.35 mmol) and 5 equiv of Si(OEt)_4_. ν_max_: 2850, 1614, 1484, and 1056 cm^–1^; ^13^C{^1^H} NMR (100 MHz, solid): δ 158.4, 153.1, 142.7, 133.7, 124.4, 118.6, 113.2, 61.8, 38.3, 25.7, 13.5; Anal. Calcd for C_26_H_26_N_2_O_5_Si_2_ + 7(SiO_2_) + 0.6(H_2_O): C, 33.4; H, 2.9; N, 3.0. Found: C, 33.3; H, 3.2; N, 3.4; TGA 1.1% weight loss below 100 °C.

#### 13e

Obtained as a yellow powder (0.43 g, 86%) from a reaction using **12a** (0.35 mmol) and 15 equiv of Si(OEt)_4_. ν_max_: 1610, 1490, and 1056 cm^–1^; ^13^C{^1^H} NMR (100 MHz, solid): δ 158.4, 153.4, 142.5, 133.2, 124.2, 118.4, 113.6, 61.0, 38.3, 25.7, 18.2, 13.8; Anal. Calcd for C_26_H_26_N_2_O_5_Si_2_ + 15(SiO_2_) + 1.5(H_2_O): C, 21.8; H, 2.0; N, 2.0. Found: C, 21.8; H, 2.4; N, 2.2; TGA 1.8% weight loss below 100 °C.

#### 13f

Obtained as a yellow powder (0.50 g, 65%) from a reaction using **12a** (0.35 mmol) and 20 equiv of Si(OEt)_4_. ν_max_: 2850, 1610, and 1056 cm^–1^; ^13^C{^1^H} NMR (100 MHz, solid): δ 158.6, 153.1, 142.3, 133.7, 124.5, 117.4, 113.4, 61.4, 38.1, 25.4, 18.5, 13.3; Anal. Calcd for C_26_H_26_N_2_O_5_Si_2_ + 27.5(SiO_2_) + 3.5(H_2_O): C, 14.1; H, 1.5; N, 1.3. Found: C, 13.8; H, 1.9; N, 1.5; TGA 2.7% weight loss below 100 °C.

### Silica-Supported Aldehyde **14**

2-Hydroxy-5-(3-triethoxysilylpropyl)benzaldehyde **8** (0.26 g, 0.80 mmol) was dissolved in toluene (50 mL), and amorphous silica (8.0 g) was added. The mixture was heated to reflux under N_2_ for 24 h and then cooled to room temperature. The solid product was isolated by filtration, washed with EtOAc (3 × 20 mL) and Et_2_O (2 × 10 mL), and dried at 85 °C in vacuo for 18 h to afford silica-supported aldehyde **14** (8.15 g) as an off-white solid. ν_max_: 3385 (br), 2987, 2924, 1650, 1048 and 799 cm^–1^; ^13^C{^1^H} NMR (100 MHz, solid): δ 200.7, 161.6, 139.5, 136.4, 122.9, 119.1, 61.3, 39.2, 26.4, 18.1, 12.7; Anal. Calcd for C_10_H_11_O_4_Si + 87(SiO_2_) + 6(H_2_O): C, 2.2; H, 0.4. Found: C, 2.2; H, 0.4; TGA 2.0% weight loss below 100 °C.

### Silica-Supported Amine (**15**)

1,2-Diaminobenzene **11a** (0.14 g, 1.3 mmol) was dissolved in EtOH (2 mL) at 60 °C, and then silica-supported aldehyde **14** (1.00 g, 0.18 mmol of aldehyde) was added. The mixture was stirred at 60 °C for 2 h and then cooled to room temperature. The solid product was isolated by filtration, washed with MeOH (3 × 15 mL) and EtOAc (3 × 15 mL), and dried at 80 °C in vacuo for 18 h to afford silica-supported amine **15** (0.90 g, 100%) as a yellow powder. ν_max_: 3411 (br), 2987, 1716, 1647, 1046 and 799 cm^–1^; ^13^C{^1^H} NMR (100 MHz, solid): δ 161.2, 139.4, 136.2, 132.7, 129.4, 127.6, 123.2, 118.6, 61.5, 39.1, 26.6, 18.0, 13.9; Anal. Calcd for C_16_H_17_N_2_O_3_Si + 78(SiO_2_) + 2(H_2_O): C, 3.8; H, 0.4. Found: C, 3.8; H, 0.5; TGA 0.7% weight loss below 100 °C.

### Silica-Supported Salophen (**16**)

Salicylaldehyde (92 mg, 0.75 mmol) was dissolved in EtOH (2 mL), and then silica-supported amine **15** (0.50 g, 0.1 mmol of amine) was added. The mixture was stirred at 60 °C for 2 h and then cooled to room temperature. The solid product was isolated by filtration, washed with EtOH (3 × 10 mL) and EtOAc (3 × 10 mL), and dried at 80 °C in vacuo for 18 h to afford silica-supported salophen **16** (0.46 g, 64%) as a light yellow powder was obtained. ν_max_: 3387 (br), 2988, 1789, 1653, 1052 and 798 cm^–1^; ^13^C{^1^H} NMR (100 MHz, solid): δ 161.4, 139.4, 136.7, 127.9, 122.8, 118.8, 61.2, 39.2, 26.1, 17.9, 13.2; Anal. Calcd for C_23_H_21_N_2_O_4_Si + 113(SiO_2_) + 2(H_2_O): C, 3.8; H, 0.4. Found: C, 3.7; H, 0.5; TGA 0.4% weight loss below 100 °C.

### Silica-Supported aldehyde (**17**)

To a solution of Si(OEt)_4_ (1.6 g, 7.5 mmol, 5.0 equiv) in EtOH (1 mL), an aqueous solution of NH_3_ (25–28% NH_3_, 1 mL) was added dropwise with vigorous stirring. After 5 min, a white suspension formed, and aldehyde **8** (0.5 g, 1.5 mmol) in EtOH (1 mL) was added. The mixture was stirred at 40 °C for 36 h, then heated, opened to air, and undisturbed at 110 °C for 48 h. The resulting powder was washed with EtOH (2 × 30 mL), H_2_O (2 × 30 mL), and EtOAc (2 × 30 mL) and then dried at 80 °C under reduced pressure for 24 h to afford silica-supported aldehyde **17** (0.9 g, 79%) as a yellow powder. ν_max_: 3350 (br), 2933, 2862, 1655 and 1056 cm^–1^; ^13^C{^1^H} NMR (100 MHz, solid): δ 198.1, 160.6, 134.2, 121.3, 117.8, 59.7, 37.8, 25.4, 18.7, 12.8; Anal. Calcd for C_10_H_11_O_3.5_Si + 9(SiO_2_) + 0.5(H_2_O): C, 15.7; H, 1.6. Found: C, 15.3; H, 2.0; TGA 1.2% weight loss below 100 °C.

### General Procedure for the Synthesis of Silica-Supported Amines (**18a–c**)

To a stirred suspension of silica-supported aldehyde **17** (0.5 g, 0.65 mmol of aldehydes, 1 equiv) in EtOH (2 mL) was added diamines **11a–c** (13 mmol, 20.0 equiv) to afford a 6.5 M solution of **11a–c**. The resulting mixture was heated at 60 °C for 3 h, and then the solid was filtered, washed with EtOH (3 × 20 mL), and dried in vacuo for 24 h to afford silica-supported amines **18a–c**.

#### 18a

Obtained as a yellow powder (0.5 g, 79%). ν_max_: 3378 (br), 2960, 2863, 1620, 1493, and 1060 cm^–1^; ^13^C{^1^H} NMR (100 MHz, solid): δ 159.9, 153.6, 142.2, 133.7, 128.9, 119.4, 59.9, 38.1, 25.7, 19.1, 13.7; Anal. Calcd for C_16_H_17_N_2_O_2.5_Si + 11(SiO_2_) + 0.25(H_2_O): C, 19.8; H, 1.8. Found: C, 19.5; H, 1.8; TGA 0.4% weight loss below 100 °C.

#### 18b

Obtained as a pale yellow powder (0.4 g, 81%) starting from 0.4 g of aldehyde **17**. ν_max_: 3284 (br), 2930, 2852, 1634, 1494, and 1043 cm^–1^; ^13^C{^1^H} NMR (100 MHz, solid): δ 168.8, 161.8, 134.2, 119.9, 62.0, 40.3, 27.1, 20.1, 15.1; Anal. Calcd for C_12_H_17_N_2_O_2.5_Si + 11(SiO_2_) + 1.5(H_2_O): C, 15.3; H, 2.1; N, 3.0. Found: C, 15.1; H, 2.2; N, 2.8; TGA 3.0% weight loss below 100 °C.

#### 18c

Obtained as a pale yellow powder (0.5 g, 59%). ν_max_: 3396 (br), 2931, 1635, 1492, and 1069 cm^–1^; ^13^C{^1^H} NMR (100 MHz, solid): δ 167.5, 162.2, 134.4, 120.0, 70.6, 62.8, 39.5, 36.3, 32.6, 27.2, 19.9, 15.3; Anal. Calcd for C_16_H_23_N_2_O_2.5_Si + 16(SiO_2_) + 2(H_2_O): C, 14.7; H, 2.1. Found: C, 14.5; H, 1.9; TGA 2.7% weight loss below 100 °C.

### General Procedure for the Synthesis of Silica-Supported Salens and Salophens (**19a–g**)

To a stirred suspension of silica-supported amines **18a–c** (0.63 mmol, 1 equiv) in EtOH (8 mL) was added a salicylaldehyde (6.35 mmol, 10.0 equiv) to afford a 0.8 M solution of salicylaldehyde. The resulting mixture was heated at 60 °C for 3 h; then, the suspension was filtered, washed with EtOH (3 × 20 mL), and then dried under reduced pressure for 24 h to afford silica-supported salens and salophens **19a–g**.

#### 19a

Obtained as a pale yellow powder (0.60 g, 71%). ν_max_: 3382 (br), 2982, 2932, 1612, 1576, 1495, 1457, and 1056 cm^–1^; ^13^C{^1^H} NMR (100 MHz, solid): δ 158.3, 153.3, 142.7, 133.7, 124.8, 118.8, 113.2, 61.6, 38.0, 25.3, 18.5, 14.1; Anal. Calcd for C_23_H_21_N_2_O_3.5_Si + 15(SiO_2_) + 1.5(H_2_O): C, 20.6; H, 1.8. Found: C, 20.6; H, 1.9; TGA 2.0% weight loss below 100 °C.

#### 19b

Obtained as a yellow powder (0.65 g, 66%). ν_max_: 2938 (br), 1636, 1405, and 1087 cm^–1^; ^13^C{^1^H} NMR (100 MHz, solid): δ 153.4, 134.5, 118.2, 114.2, 59.8, 38.5, 19.0; Anal. Calcd for C_31_H_37_N_2_O_3.5_Si + 17(SiO_2_) + 0.5(H_2_O): C, 24.0; H, 2.5; N, 1.8. Found: C, 23.8; H, 2.4; N, 1.8; TGA 0.5% weight loss below 100 °C.

#### 19c

Obtained as a yellow powder (0.24 g, 49%) starting from 0.31 g of amine **18b**. ν_max_: 3200 (br), 2982, 2933, 1633, 1493, 1049, and 784 cm^–1^; ^13^C{^1^H} NMR (100 MHz, solid): δ 169.0, 161.8, 134.3, 120.1, 61.9, 39.8, 27.2, 19.9, 15.2; Anal. Calcd for C_19_H_21_N_2_O_3.5_Si + 17(SiO_2_) + 3(H_2_O): C, 15.9; H, 1.9; N, 2.0. Found: C, 16.1; H, 1.8; N, 1.9; TGA 3.7% weight loss below 100 °C.

#### 19d

Obtained as a pale yellow powder (0.4 g, 55%). ν_max_: 3429 (br), 2938, 1636, 1492, and 1087 cm^–1^; ^13^C{^1^H} NMR (100 MHz, solid): δ 160.8, 133.3, 119.2, 61.6, 36.9, 25.7, 18.4, 14.5; Anal. Calcd for C_23_H_27_N_2_O_3.5_Si + 12(SiO_2_) + 0.5(H_2_O): C, 24.1; H, 2.5; N, 2.4. Found: C, 23.8; H, 2.4; N, 2.3; TGA 0.8% weight loss below 100 °C.

#### 19e

Obtained as a pale yellow powder (640 mg, 56%). ν_max_: 3435 (br), 2990, 2924, 1617, 1497, and 1061 cm^–1^; ^13^C{^1^H} NMR (100 MHz, solid): δ 161.2, 157.4, 134.5, 130.8, 125.7, 119.9, 113.4, 63.0, 55.3, 39.5, 27.3, 19.5, 15.2; Anal. Calcd for C_24_H_23_N_2_O_4.5_Si + 22(SiO_2_) + 2(H_2_O): C, 16.0; H, 1.5. Found: C, 16.1; H, 1.4; TGA 2.0% weight loss below 100 °C.

#### 19f

Obtained as a pale yellow powder (630 mg, 66%). ν_max_: 3369 (br), 2929, 1618, 1492, and 1048 cm^–1^; ^13^C{^1^H} NMR (100 MHz, solid): δ 167.8, 161.5, 154.7, 143.2, 133.2, 131.4, 120.0, 62.9, 39.5, 27.2, 19.8, 15.0; Anal. Calcd for C_23_H_20_N_3_O_5.5_Si + 17(SiO_2_) + 2(H_2_O): C, 18.3; H, 1.6. Found: 18.6; H, 1.9; TGA 2.25% weight loss below 100 °C.

#### 19g

Obtained as a pale yellow powder (610 mg, 64%). ν_max_: 3397 (br), 2937, 1628, 1495, and 1056 cm^–1^; ^13^C{^1^H} NMR (100 MHz, solid): δ 161.7, 154.1, 132.8, 128.5, 120.0, 62.9, 39.9, 32.6, 27.9, 19.5, 15.5; Anal. Calcd for C_27_H_29_N_2_O_3.5_Si + 17(SiO_2_) + 1.5(H_2_O): C, 21.4; H, 2.1. Found: 21.5; H, 2.3; TGA 2.0% weight loss below 100 °C.

### General Procedure for the Synthesis of Cyclic Carbonates (**21a–f**) by Silica-Supported Catalysts **13** at 10 bar Pressure

Epoxide (2.0 mmol, 1 equiv) and catalyst (0.5–1 mol % of salophen units, 0.005–0.01 equiv) were added to a 7 mL glass sample vial fitted with a magnetic stir bar, which was then placed in a 500 mL stainless steel autoclave which had been preheated to 100–120 °C. The autoclave was sealed and flushed three times with CO_2_ before being pressurized to 10 bar with CO_2_. The reaction mixture was stirred (350 rpm) for 3–48 h; then, the autoclave was cooled to room temperature and further cooled with liquid N_2_ before the pressure vessel was opened, and the contents were allowed to warm to room temperature. EtOAc (2 mL) was added to the reaction vial, and the mixture was transferred to a 2 mL centrifuge vial. The reaction mixture was centrifuged for 5 min; then, the EtOAc solution was pipetted away from the solid catalyst. The solvent was evaporated in vacuo, and the residue was analyzed by ^1^H NMR spectroscopy to determine the conversion. The residue was purified by flash chromatography to afford cyclic carbonates **21a–f**.

#### **21a**(34)

Obtained as a white solid (338 mg, 87%) from a reaction carried out at 120 °C using catalyst **13a** (1 mol % of salophen units) for 3.5 h and purified by flash chromatography eluting with 3:2 petroleum ether (60–40)/EtOAc. ν_max_: 3060, 2990, 1790, 1610, and 1600 cm^–1^; ^1^H NMR (300 MHz, CDCl_3_): δ 7.4–7.2 (2H, m), 7.04 (1H, t, *J* = 8.5 Hz), 6.94 (2H, d, *J* = 8.5 Hz), 5.1–5.0 (1H, m), 4.63 (1H, t, *J* = 8.4 Hz), 4.55 (1H, dd, *J* = 8.5, 6.0 Hz), 4.26 (1H, dd, *J* = 10.6, 4.0 Hz), 4.16 (1H, dd, *J* = 10.6, 6.1 Hz); ^13^C{^1^H} NMR (75 MHz, CDCl_3_): δ 157.8, 154.7, 129.7, 122.0, 114.7, 74.2, 66.9, 66.2; found (ESI), 217.0465, calcd for C_10_H_10_O_4_Na [M + Na]^+^, 217.0471.

#### **21b**(2,11,34)

Obtained as a white solid (312 mg, 95%) from a reaction carried out at 120 °C using catalyst **13d** (1 mol % of salophen units) for 24 h and purified by flash chromatography eluting with 3:2 petroleum ether (60–40)/EtOAc. ν_max_: 3040, 3020, 2920, 2850, and 1780 cm^–1^; ^1^H NMR (300 MHz, CDCl_3_): δ 7.5–7.3 (m, 5H), 5.70 (1H, t, *J* = 8.3 Hz), 4.82 (1H, t, *J* = 8.3 Hz), 4.37 (1H, t, *J* = 8.3 Hz); ^13^C{^1^H} NMR (75 MHz, CDCl_3_): δ 154.8, 135.8, 129.8, 129.3, 125.9, 78.0, 71.1; found (ESI), 187.0365, calcd for C_9_H_8_O_3_Na [M + Na]^+^, 187.0366.

#### **21c**(2,11,34)

Obtained as a white solid (354 mg, 89%) from a reaction carried out at 120 °C using catalyst **13d** (1 mol % of salophen units) for 3 h and purified by flash chromatography eluting with 3:2 petroleum ether (60–40)/EtOAc. ν_max_: 3088, 2964, and 1790 cm^–1^; ^1^H NMR (300 MHz, CDCl_3_): δ 7.45 (2H, d, *J* = 8.6 Hz), 7.33 (2H, d, *J* = 8.3 Hz), 5.68 (1H, t, *J* = 8.1 Hz), 4.82 (1H, t, *J* = 8.5 Hz), 4.33 (1H, dd, *J* = 8.7, 7.9 Hz); ^13^C{^1^H} NMR (75 MHz, CDCl_3_): δ 154.5, 135.8, 134.3, 129.5, 127.2, 77.2, 71.0; found (ESI), 220.9976, calcd for C_9_H_7_^35^ClO_3_Na [M + Na]^+^, 220.9976.

#### **21d**(2,11,34)

Obtained as a colorless oil (312 mg, 78%) from a reaction carried out at 120 °C using catalyst **13d** (1 mol % of salophen units) for 48 h and purified by flash chromatography eluting with 3:2 petroleum ether (60–40)/EtOAc. ν_max_: 2930, 2834, and 1798 cm^–1^; ^1^H NMR (300 MHz, CDCl_3_): δ 4.8–4.6 (1H, m), 4.54 (1H, t, *J* = 8.2 Hz), 4.08 (1H, dd, *J* = 8.3, 7.2 Hz), 1.9–1.6 (2H, m), 1.5–1.2 (12H, m), 0.90 (3H, t, *J* = 6.7 Hz); ^13^C{^1^H} NMR (75 MHz, CDCl_3_): δ 155.1, 77.0, 69.4, 33.9, 31.8, 29.3, 29.1, 29.0, 24.4, 22.6, 14.1; found (ESI), 223.1302, calcd for C_11_H_20_O_3_Na [M + Na]^+^, 223.1305; found (ESI), 201.1486, calcd for C_11_H_21_O_3_ [M + H]^+^, 201.1485.

#### **21e**(2,11,34)

Obtained as a colorless oil (401 mg, 88%) from a reaction carried out at 120 °C using catalyst **13d** (1 mol % of salophen units) for 48 h and purified by flash chromatography eluting with 3:2 petroleum ether (60–40)/EtOAc. ν_max_: 2930, 2830, and 1798 cm^–1^; ^1^H NMR (300 MHz, CDCl_3_): δ 4.8–4.6 (1H, m), 4.54 (1H, t, *J* = 8.1 Hz), 4.08 (1H, dd, *J* = 8.3, 7.2 Hz), 1.9–1.6 (2H, m), 1.5–1.2 (16H, m), 0.90 (3H, t, *J* = 6.7 Hz); ^13^C{^1^H} NMR (75 MHz, CDCl_3_): δ 155.1, 77.0, 69.4, 33.9, 31.9, 29.5, 29.4, 29.3, 29.2, 29.1, 24.4, 22.7, 14.1; found (ESI), 251.1613, calcd for C_13_H_24_O_3_Na [M + Na]^+^, 251.1618; found (ESI), 229.1804, calcd for C_13_H_25_O_3_ [M + H]^+^, 229.1798.

#### **21f**(11)

Obtained as a colorless oil (210 mg, 58%) from a reaction carried out at 120 °C using catalyst **13d** (1 mol % of salophen units) for 3.5 h and purified by flash chromatography eluting with 3:2 petroleum ether (60–40)/EtOAc. ν_max_: 2980 and 1790 cm^–1^; ^1^H NMR (300 MHz, CDCl_3_): δ 5.0–4.9 (1H, m), 4.62 (1H, t, *J* = 8.5 Hz), 4.38 (1H, dd, *J* = 8.9, 5.9 Hz), 3.62 (1H, dd, *J* = 11.0, 4.4 Hz), 3.57 (1H, dd, *J* = 11.0, 6.5 Hz); ^13^C{^1^H} NMR (75 MHz, CDCl_3_): δ 154.0, 74.0, 68.1, 31.0; found (ESI), 202.9312, calcd for C_4_H_5_^79^BrO_3_Na [M + Na]^+^, 202.9314.

### Catalyst **13a** Recyclability Study

A reaction with epoxide **20a** was first carried out as described above using catalyst **13a**. The solid obtained after centrifugation was washed with EtOAc (3 × 2 mL) and then dried at 80 °C in vacuo overnight. The catalyst was analyzed by FT-IR and then used again for cyclic carbonate synthesis with the mass of epoxide used being adjusted to keep the epoxide-to-catalyst ratio constant. This process was repeated five times.

### General Procedure for the Synthesis of Cyclic Carbonates (**21a–g**) by Silica-Supported Catalysts **13a** and **19a,e–g** at 1 bar Pressure

Epoxides **20a–g** (2.0 mmol, 1 equiv) and catalyst (1–2 mol % of salophen units, 0.01–0.02 equiv) were added to a glass sample vial fitted with a magnetic stir bar. The vial was sealed with a plastic lid, and a balloon filled with CO_2_ and attached to a needle was used to purge the vial with CO_2_ three times. The balloon was then left in place, and the vial was heated to 100–120 °C before being stirred for 7–24 h. The vial was then allowed to cool to room temperature. EtOAc (2 mL) was added, and the mixture was transferred to a 2 mL centrifuge vial. The reaction mixture was centrifuged for 5 min, and then the EtOAc solution was pipetted away from the solid catalyst. The solvent was evaporated in vacuo, and the residue was analyzed by ^1^H NMR spectroscopy to determine the conversion. The residue was purified by flash chromatography to afford cyclic carbonates **21a–g**. Purification and spectroscopic data for compounds **21a–f** were identical to those reported above for reactions carried out at 10 bar CO_2_ pressure.

#### **21g**(2,11,34)

Obtained as a white solid (191 mg, 70%) from a reaction carried out at 100 °C using catalyst **19a** (1 mol % of salophen units) for 24 h and purified by flash chromatography eluting with 3:2 hexane/EtOAc. Mp 68–70 °C (lit.^2,11,34^ 68–69 °C); ν_max_: 2963, 2917, and 1781 cm^–1^; ^1^H NMR (400 MHz, CDCl_3_): δ 5.0–4.9 (1H, m), 4.57 (1H, t, *J* = 8.6 Hz), 4.38 (1H, dd, *J* = 8.9, 5.7 Hz), 3.78 (1H, dd, *J* = 12.2, 5.2 Hz), 3.70 (1H, dd, *J* = 12.2, 3.7 Hz); ^13^C{^1^H} NMR (100 MHz, CDCl_3_): δ 154.5, 74.5, 67.1, 43.0; found (ESI), 158.9817, calcd for C_4_H_5_^35^ClO_3_Na [M + Na]^+^, 158.9819.

### General Procedure for the Synthesis of Silica-Supported Aluminum Complexes **22a–c**

To a stirred suspension of silica-supported ligands **13a** and **19a,d** (0.1 mmol, 1 equiv) in dry toluene (2 mL) under a N_2_ atmosphere was added Et_2_AlCl (0.9 M in toluene, 2.22 mL, 2.0 mmol, 20 equiv) to afford a 0.5 M solution of Et_2_AlCl. The resulting mixture was stirred at room temperature for 16 h. Then, the mixture was filtered, washed with EtOAc (3 × 10 mL), and dried under reduced pressure at 80 °C for 24 h to afford complexes **22a–c** as yellow powders.

#### 22a

Obtained as a yellow powder (84 mg, 67%). ν_max_: 3355 (br), 2934, 1654, 1488, and 1061 cm^–1^; ^13^C{^1^H} NMR (100 MHz, solid) 158.5, 153.1, 142.1, 133.2, 124.4, 118.4, 113.3, 61.4, 38.1, 25.8, 13.8; found (ICP–OES) Al = 1.0 mmol g^–1^; calcd for C_26_H_24_AlClN_2_O_27_Si_13_ Al = 0.8 mmol g^–1^; XPS: Al (3.9%), Cl (1.4%).

#### 22b

Obtained as a yellow powder (140 mg, 100%). ν_max_: 3305 (br), 2928, 1625, 1504, and 1069 cm^–1^; ^13^C{^1^H} NMR (100 MHz, solid) 161.9, 134.1, 120.1, 72.4, 63.0, 38.9, 32.1, 27.2, 20.3, 15.2; found (ICP–OES) Al = 0.2 mmol g^–1^; calcd for C_23_H_19_AlClN_2_O_25.5_Si_12_ Al = 0.9 mmol g^–1^.

#### 22c

Obtained as a yellow powder (109 mg, 90%). ν_max_: 3357 (br), 2931, 2861, 1645, 1634, 1599, 1495, and 1061 cm^–1^; ^13^C{^1^H} NMR (100 MHz, solid) 160.7, 155.3, 143.8, 134.4, 125.7, 121.6, 61.3, 39.4, 31.7, 27.1, 19.3, 15.0; found (ICP–OES) Al = 0.5 mmol g^–1^; calcd for C_23_H_25_AlClN_2_O_25.5_Si_12_ Al = 0.9 mmol g^–1^.

### General Procedure for the Synthesis of Silica-Supported Manganese Complexes **22d,e**

To a stirred suspension of silica-supported ligands **13a** and **19d** (0.078 mmol, 1 equiv) in EtOH (5 mL) was added Mn(OAc)_2_·4H_2_O (29 mg, 0.086 mmol, 1.1 equiv) to afford a 0.02 M solution of Mn(OAc)_2_, and the resulting mixture was heated at reflux for 3 h. Then, LiCl was added, and the resulting mixture was heated at reflux for 30 min. The mixture was cooled and filtered, and the solid was washed with EtOH (3 × 10 mL) before being dried under reduced pressure at 80 °C for 24 h to afford complexes **22d,e**.

#### 22d

Obtained as a brown powder (104 mg, 81%). ν_max_: 3298, 2927, 1618, 1504, and 1065 cm^–1^; found (ICP–OES) Mn = 0.1 mmol g^–1^; calcd for C_26_H_24_MnClN_2_O_27_Si_13_ Mn = 0.8 mmol g^–1^; XPS: Mn (0.9%), Cl (0.2%).

#### 22e

Obtained as a brown powder (105 mg, 85%). ν_max_: 3420 (br), 2929, 2859, 1634, 1488, and 1049 cm^–1^; found (ICP–OES) Mn = 0.3 mmol g^–1^; calcd for C_23_H_25_MnClN_2_O_25.5_Si_12_ Mn = 0.9 mmol g^–1^.

### Synthesis of Silica-Supported Copper Complex **22f**

To a stirred suspension of silica-supported ligand **13a** (100 mg, 0.078 mmol) in EtOH (5 mL) was added Cu(OAc)_2_·4H_2_O (22 mg, 0.086 mmol), and the resulting mixture was stirred at room temperature for 16 h. Then, the mixture was filtered, and the solid was washed with EtOH (3 × 10 mL) and dried under reduced pressure at 80 °C for 24 h to afford complex **22f** as a dark yellow powder (80 mg, 74%). ν_max_: 3289 (br), 2988, 2935, 1623, 1503, and 1076 cm^–1^; found (ICP–OES) Cu = 1.0 mmol g^–1^; calcd for C_26_H_24_CuN_2_O_27_Si_13_ Cu = 0.8 mmol g^–1^; XPS: Cu (1.3%).

### Synthesis of Silica-Supported Vanadium Complex **22g**

To a stirred suspension of silica-supported ligand **13a** (100 mg, 0.078 mmol) in THF (5 mL) was added VOCl_3_ (0.01 mL, 0.12 mmol) under N_2_. The resulting mixture was heated at reflux for 3 h. Then, the mixture was filtered, and the solid was washed with THF (3 × 10 mL) and dried under reduced pressure at 80 °C for 24 h to afford complex **22g** as a green powder (100 mg, 90%). ν_max_: 3276 (br), 2927, 1624, 1514, 1463 and 1072 cm^–1^; XPS: V (0.9%), Cl (2.3%).

### Synthesis of 3-Phenoxypropylene Carbonate **21a** at 1 bar CO_2_ Pressure Using Aluminum Complexes **22b,c**

Epoxide **20a** (0.25 g, 1.66 mmol), catalysts **22b,c** (1–4 mol % of aluminum), and Bu_4_NBr (5.3 mg, 0.017 mmol) were placed in a flask fitted with a magnetic stirrer bar and sealed with a subaseal. CO_2_ (from a balloon) was flushed through the flask, and then a balloon of CO_2_ was left attached to the reaction. The flask was heated to 50 °C for 24 h and then cooled to room temperature. EtOAc (2 mL) was added, and the mixture was transferred to a 2 mL centrifuge vial. The reaction mixture was centrifuged for 5 min, and then the EtOAc solution was pipetted away from the solid catalyst. The solvent was evaporated in vacuo, and the residue was analyzed by ^1^H NMR spectroscopy to determine the conversion of epoxide **20a** into cyclic carbonate **21a**.

## Data Availability

Data Availability: The data underlying this study are openly available in the White Rose repository at DOI: 10.15124/a75a10c5-b08b-4d17-8b0c-cb8ad34e0a77.

## Supporting Information Available

Copies of infrared and NMR spectra along with TGA traces, analytical calculations, and details of the flow reactor experiments (PDF)
