# Supplementary material for: General Approach to Silica-Supported Salens and Salophens and Their Use as Catalysts for the Synthesis of Cyclic Carbonates from Epoxides and Carbon Dioxide
Source: J Org Chem. 2022 Dec 1;87(24):16410–23. doi: 10.1021/acs.joc.2c02104 (PMC9764361; doi:10.1021/acs.joc.2c02104)
Supplement: Supplementary file 1 — jo2c02104_si_001.pdf [file jo2c02104_si_001.pdf]

General Approach to Silica-Supported Salens and Salophens and Their Use as Catalysts for the Synthesis of Cyclic Carbonates from Epoxides and Carbon Dioxide

Supporting Information

Ryan E. Barker, Liping Guo, Claudio J. A. Mota, Michael North*, Leonardo P. Ozorio, William Pointer, Sarah Walberton and Xiao Wu

Characterizing data for:

4-Allylphenol (**5**) S3

2-Hydroxy-5-allylbenzaldehyde (**7**) S6

2-Hydroxy-5-(3-triethoxysilylpropyl)benzaldehyde (**8**) S9

N,*N*'-Bis(2-hydroxy-5-(3-triethoxysilylpropyl)benzylidene)-1,2-diaminobenzene (**12a**) S12

N,*N*'-Bis(2-hydroxy-5-(3-triethoxysilylpropyl)benzylidene)-1,2-diaminoethane (**12b**) S15

N,*N*'-Bis(2-hydroxy-5-(3-triethoxysilylpropyl)benzylidene)-*trans*-1,2-diaminocyclohexane (**12c**) S18

Silica-supported salophen **13a** S21

Silica-supported salen **13b** S25

Silica-supported salen **13c** S29

Silica-supported salophen **13d** S33

Silica-supported salophen **13e** S37

Silica-supported salophen **13f** S41

Silica-supported aldehyde **14** S45

Silica-supported amine **15** S49

Silica-supported salophen **16** S53

Silica-supported aldehyde **17** S57

Silica-supported amine **18a** S61

Silica-supported amine **18b** S65

Silica-supported amine **18c** S69

Silica-supported salophen **19a** S73

Silica-supported salophen **19b** S77

Silica-supported salen **19c** S81

Silica-supported salen **19d** S85

Silica-supported salophen **19e** S89

Silica-supported salophen **19f** S93

Silica-supported salophen **19g** S96

3-Phenoxypropylene carbonate (**21a**) S100

Styrene carbonate (**21b**) S103

4-Chlorostyrene carbonate (**21c**) S106

Dec-1-ene carbonate (**21d**) S109

Dodec-1-ene carbonate (**21e**) S112

3-Bromopropylene carbonate (**21f**) S115

3-Chloropropylene carbonate (**21g**) S118

Silica-supported aluminum salophen complex **22a** S122

Silica-supported aluminum salophen complex **22b** S124

Silica-supported aluminum salen complex **22c** S126

Silica-supported manganese salophen complex **22d** S128

Silica-supported manganese salen complex **22e** S129

Silica-supported copper salophen complex **22f** S130

Silica-supported vanadyl salophen complex **22g** S131

Flow reactor results S132

4-Allylphenol (5)

IR spectrum

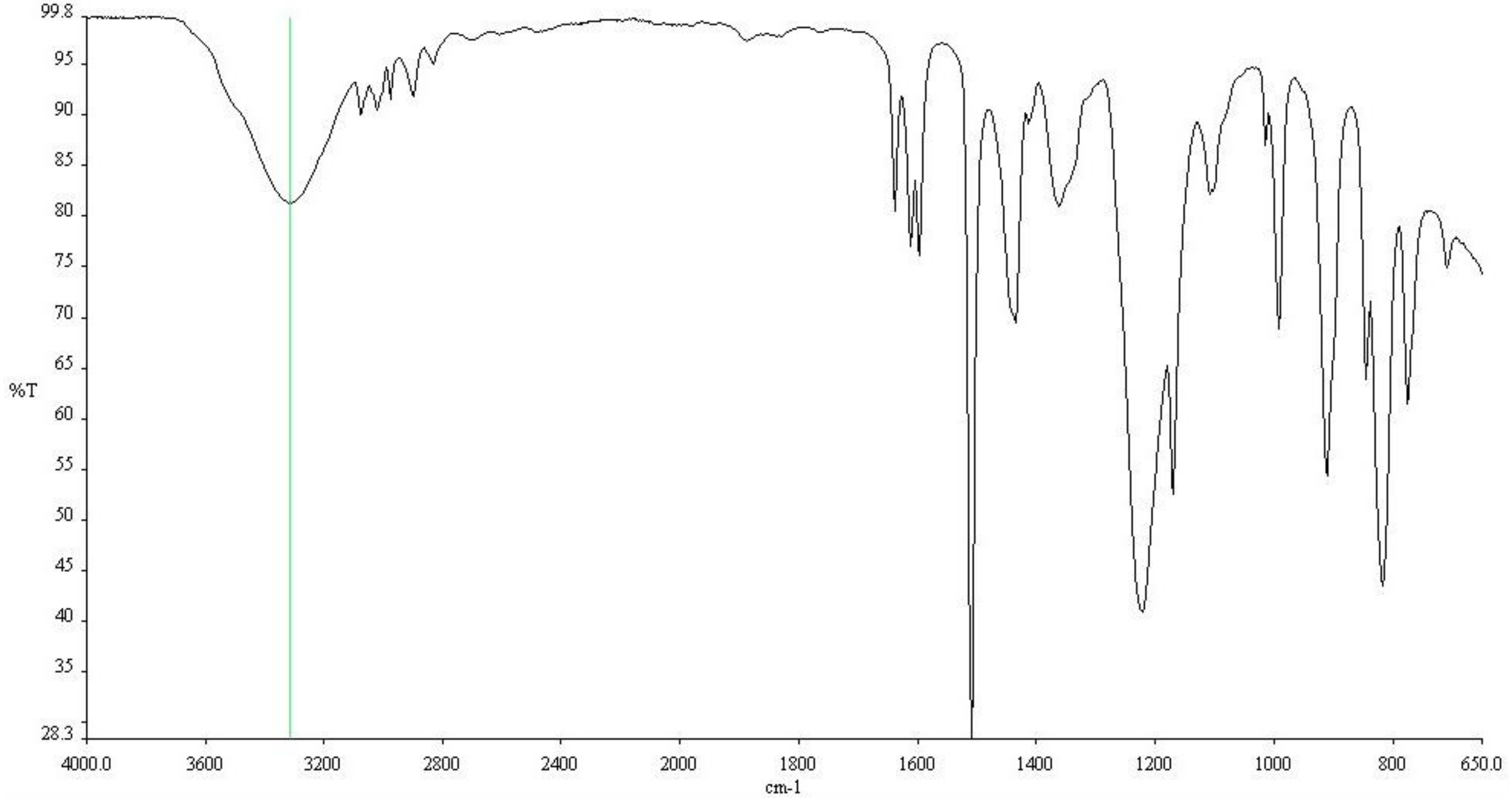

^{1}H NMR Spectrum (300 MHz, $CDCl_3$)

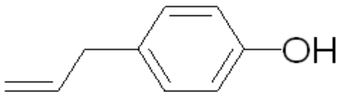

5

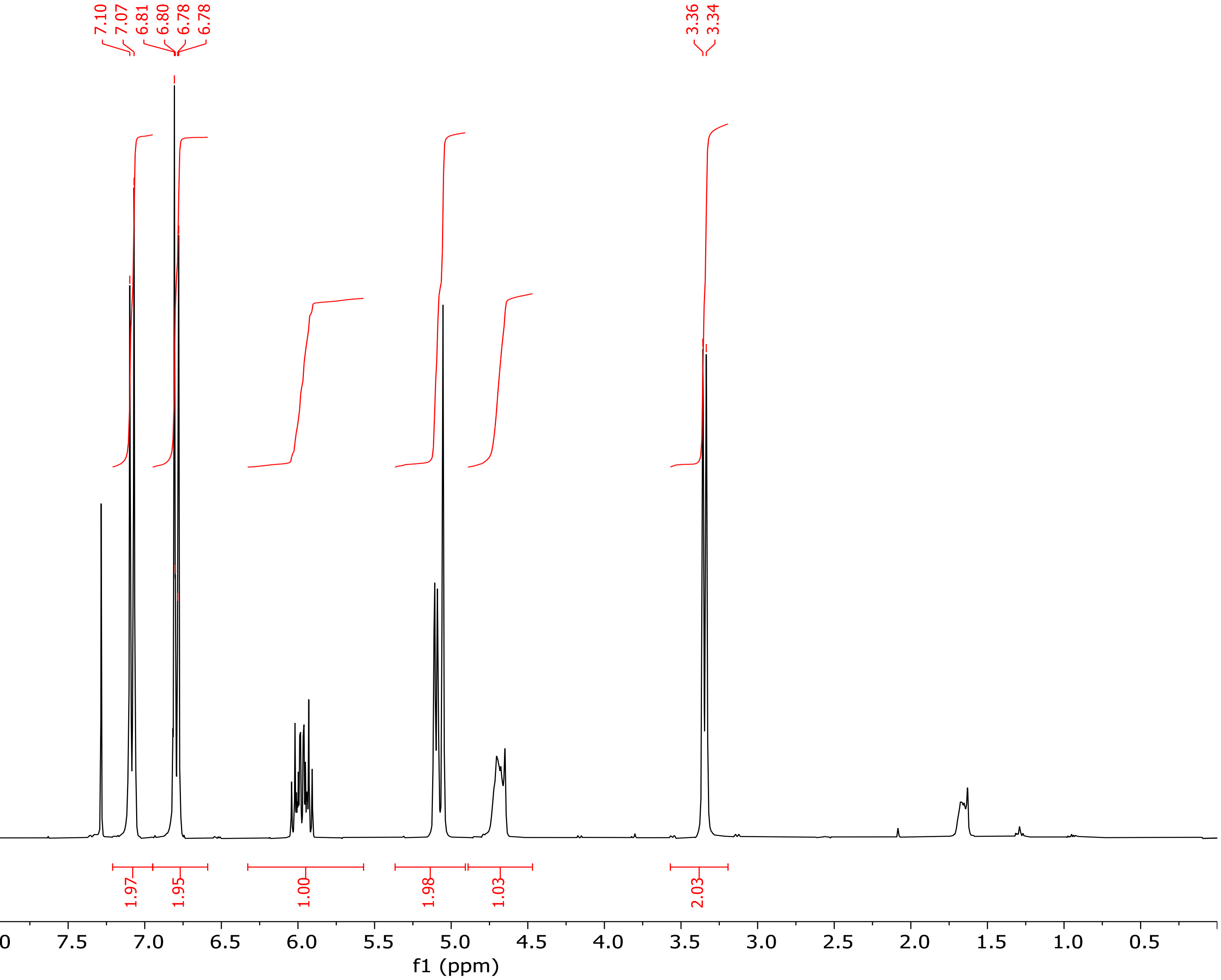

$^{13}C\{^{1}H\}$ NMR Spectrum (75 MHz, $CDCl_3$)

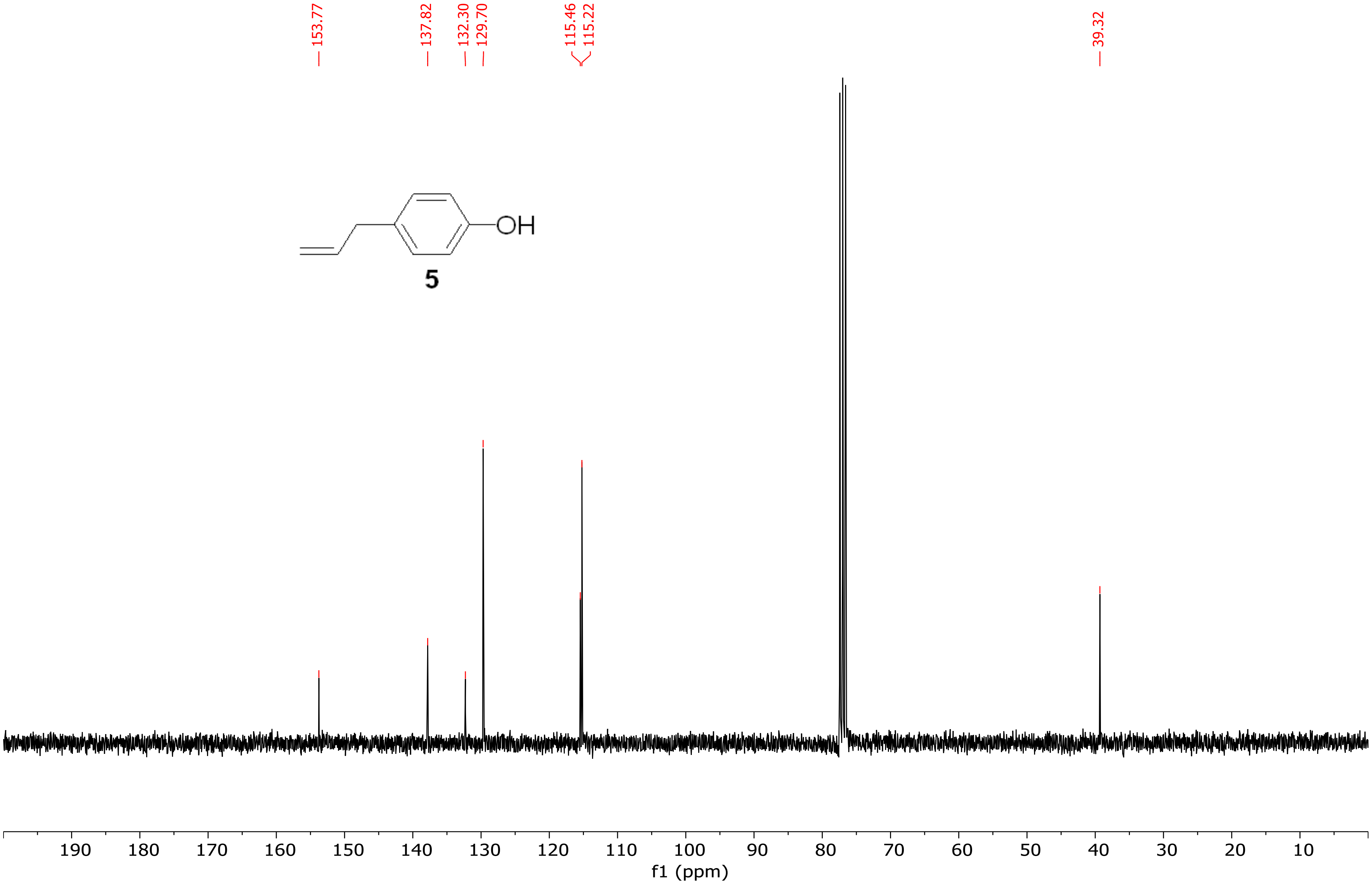

2-Hydroxy-5-allylbenzaldehyde (7)

IR spectrum

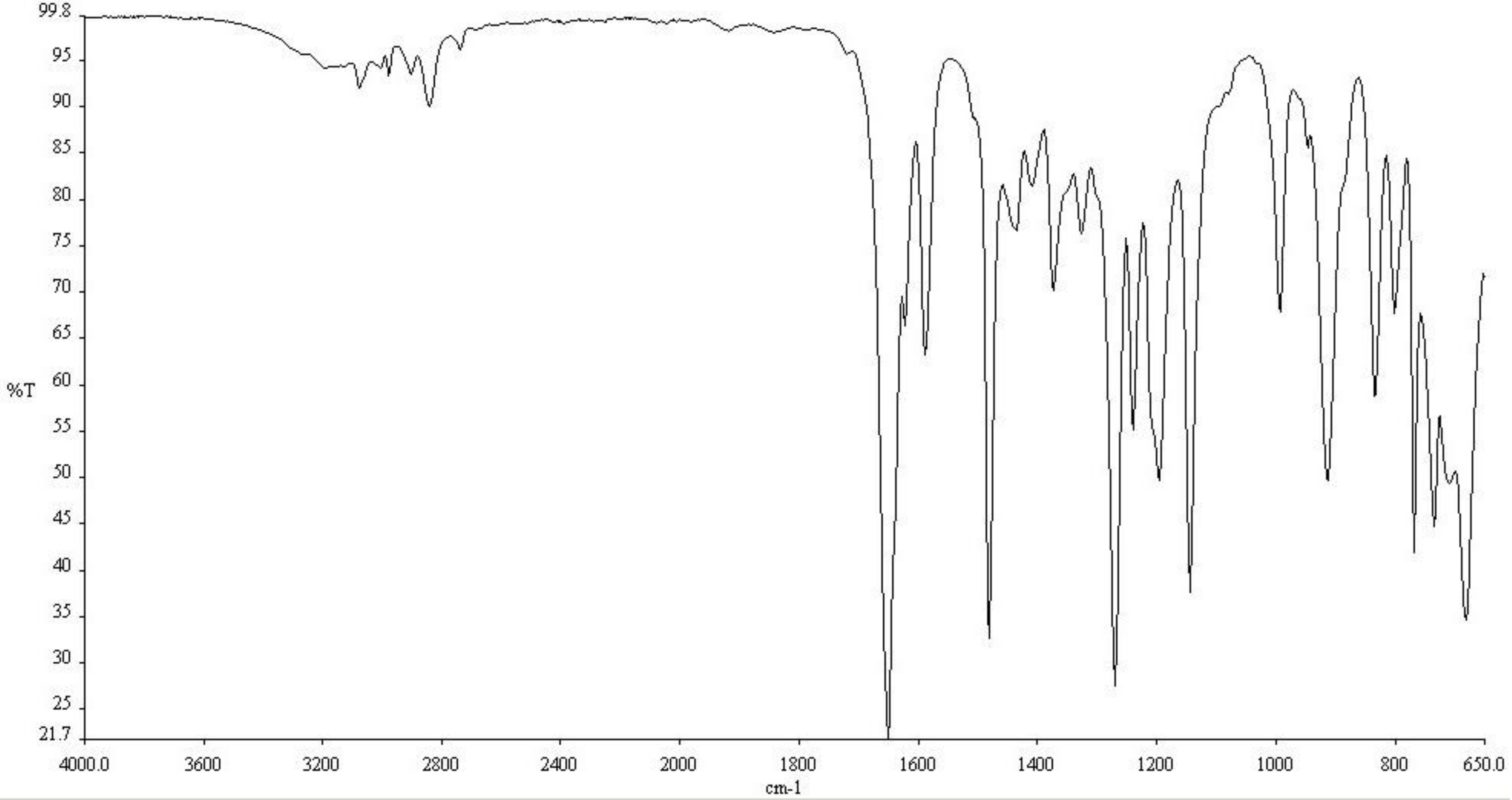

^{1}H NMR Spectrum (300 MHz, $CDCl_3$)

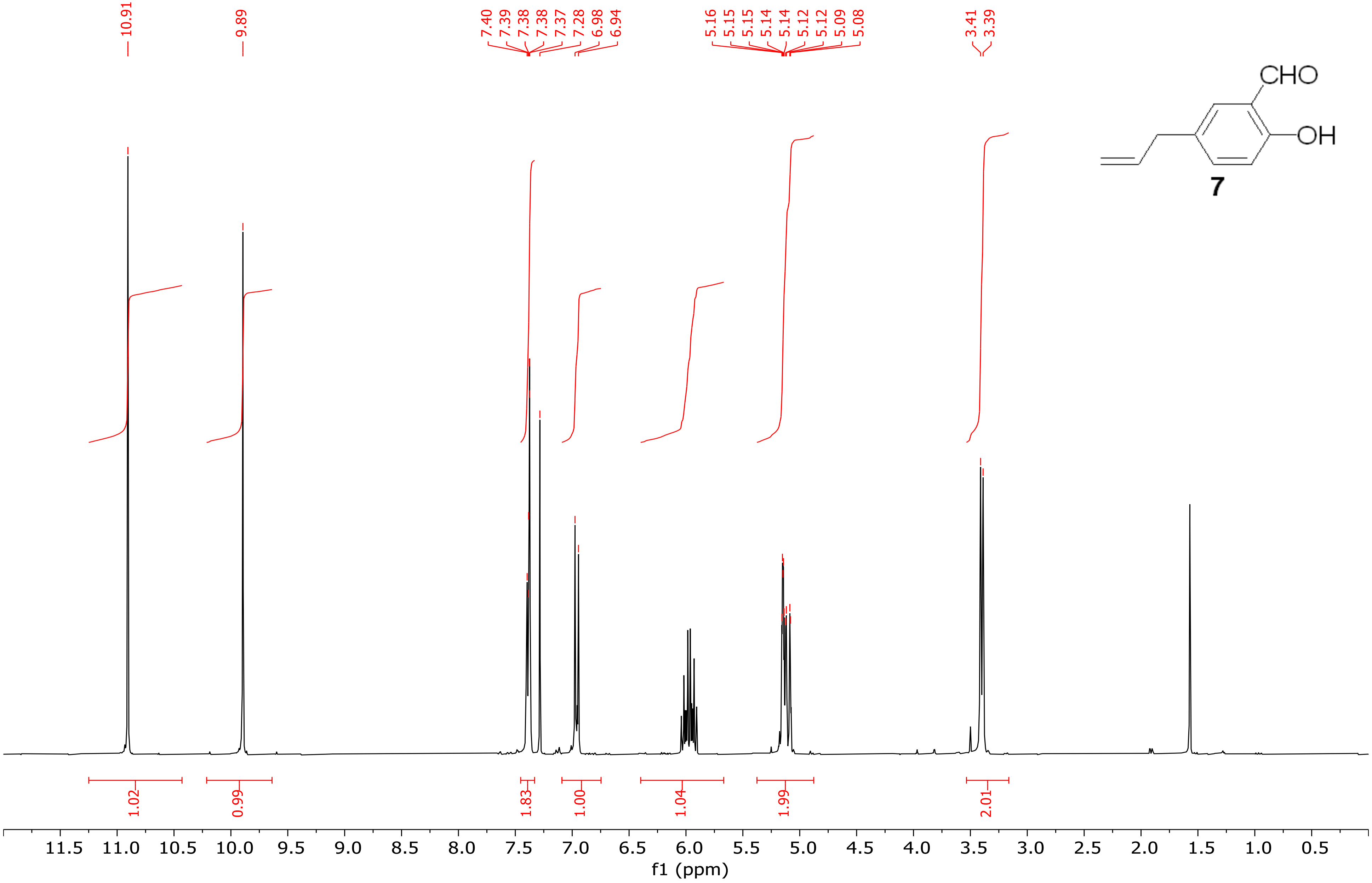

$^{13}C\{^{1}H\}$ NMR Spectrum (75 MHz, $CDCl_3$)

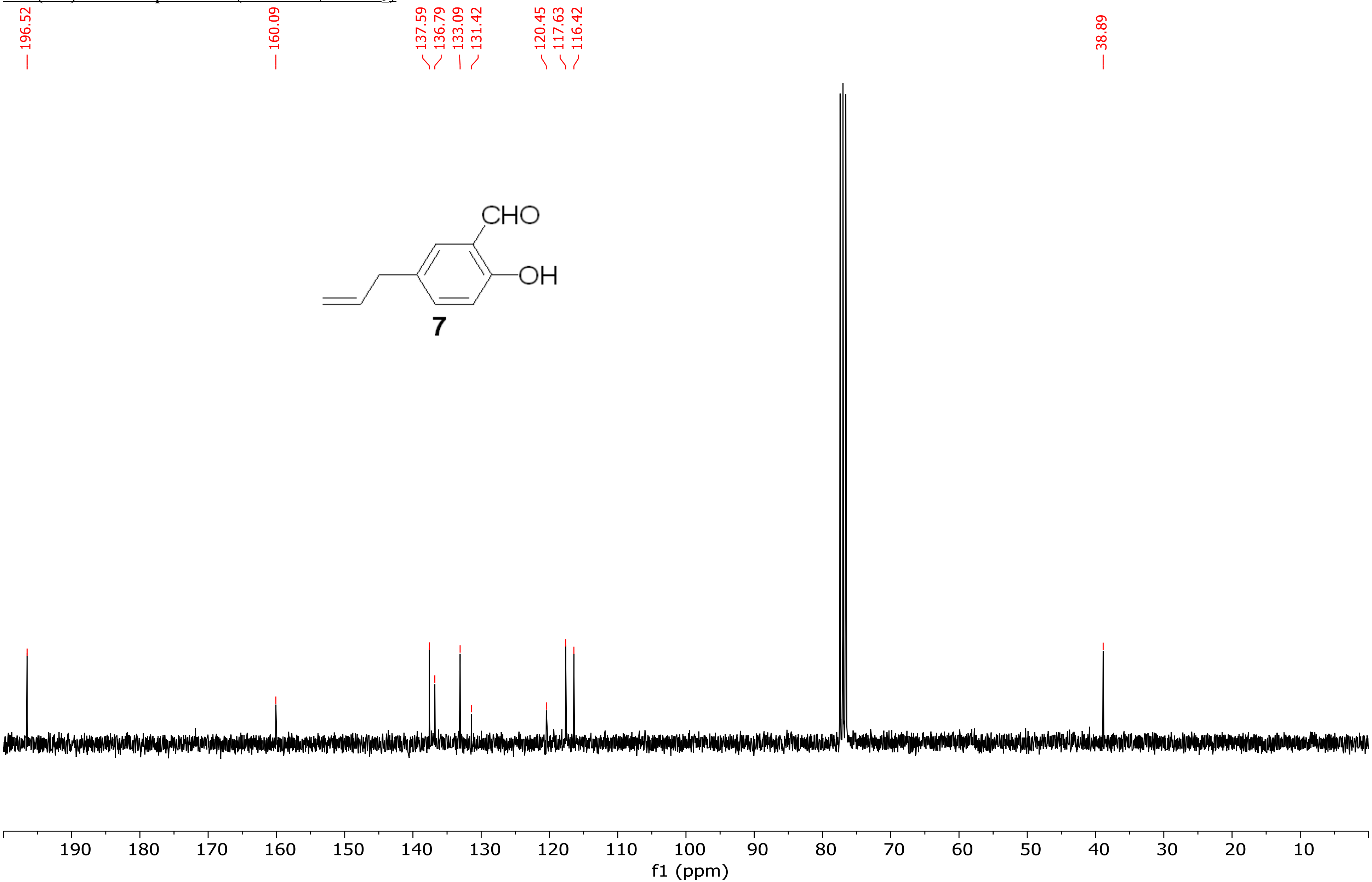

2-Hydroxy-5-(3-triethoxysilylpropyl)benzaldehyde (8)

IR spectrum

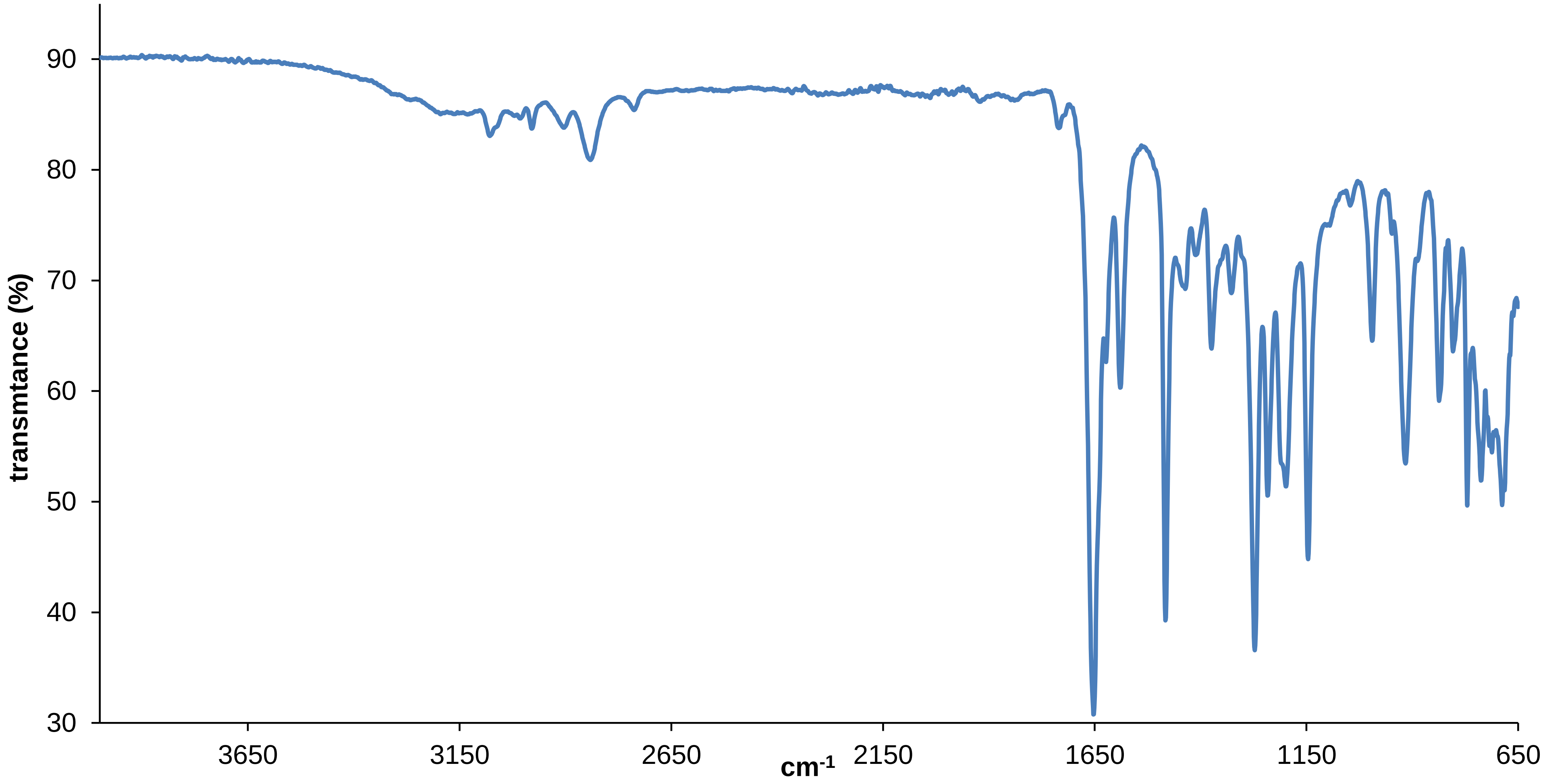

^{1}H NMR Spectrum (300 MHz, $CDCl_3$)

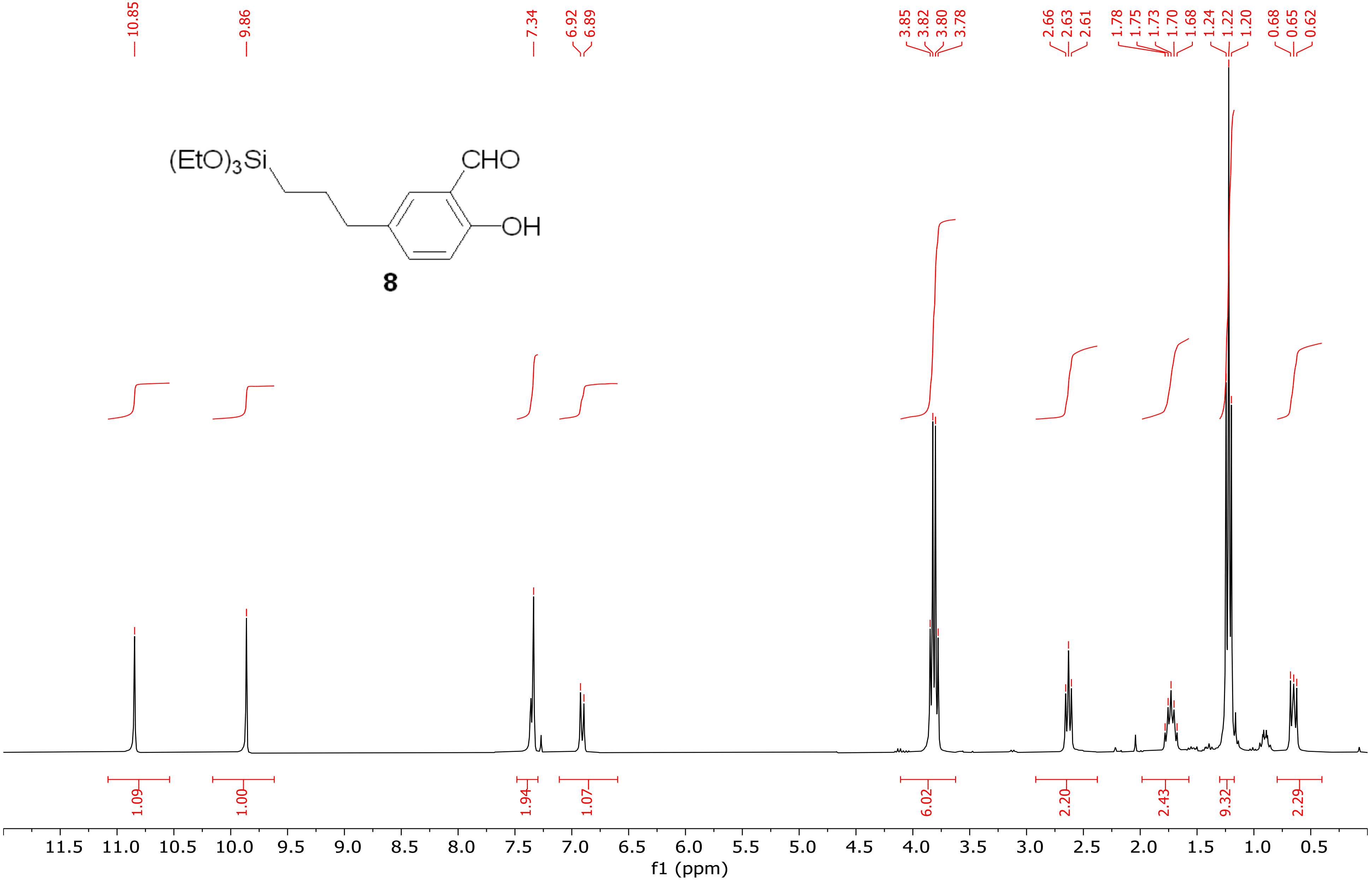

$^{13}C\{^1H\}$ NMR Spectrum (75 MHz, $CDCl_3$)

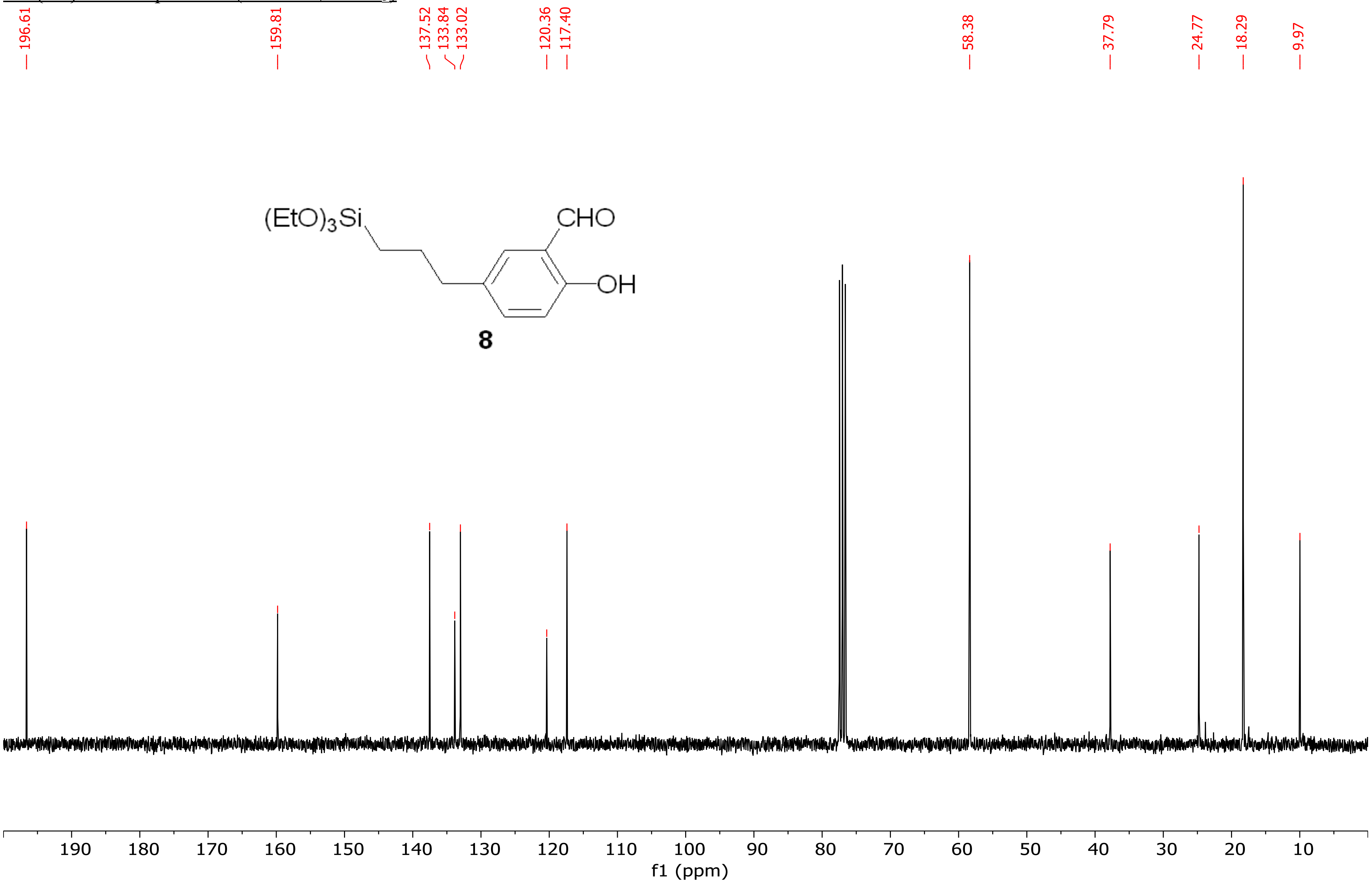

_N_,_N_'-Bis(2-hydroxy-5-(3-triethoxysilylpropyl)benzylidene)-1,2-diaminobenzene (12a)

<u>IR spectrum</u>

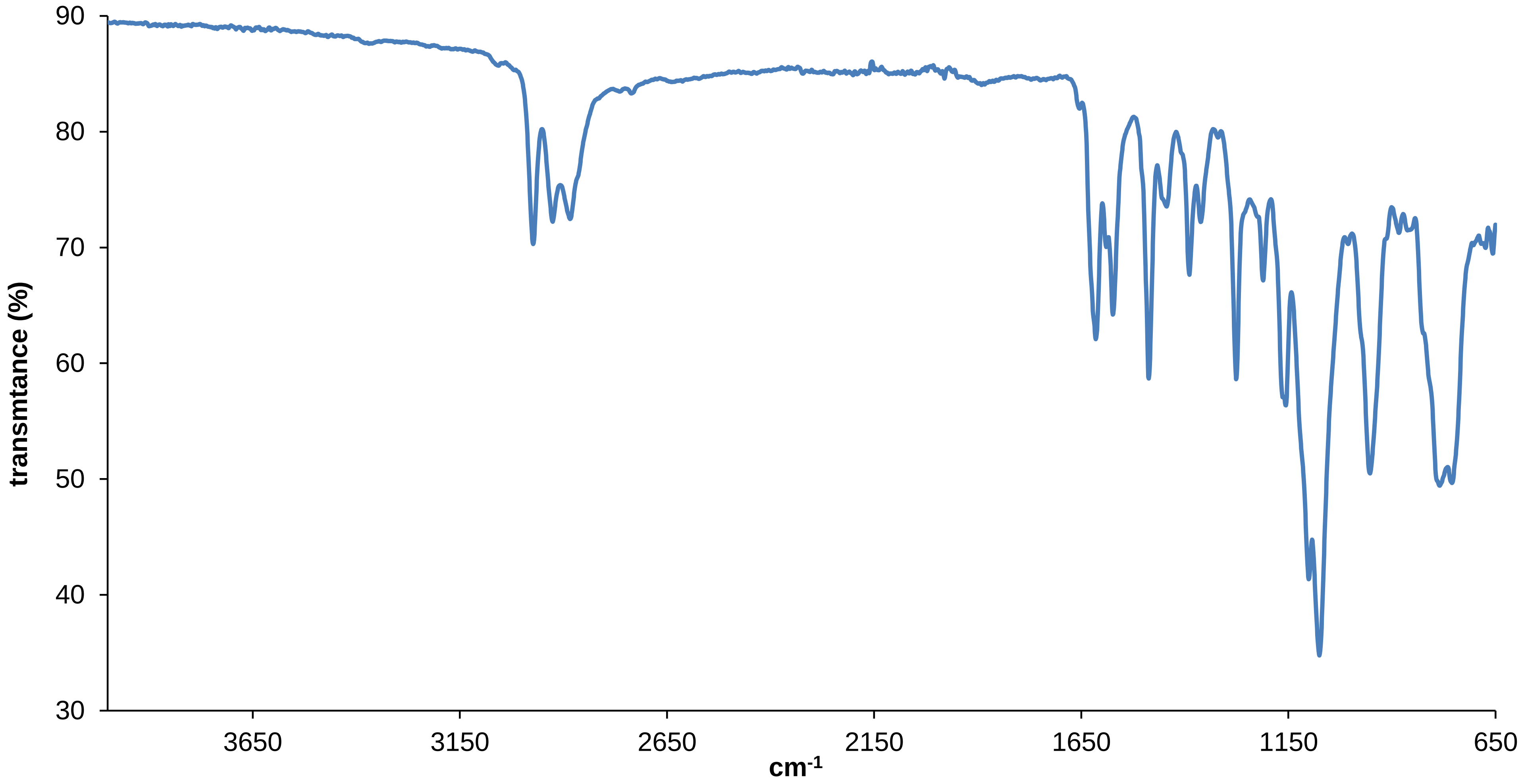

^{1}H NMR Spectrum (300 MHz, $CDCl_3$)

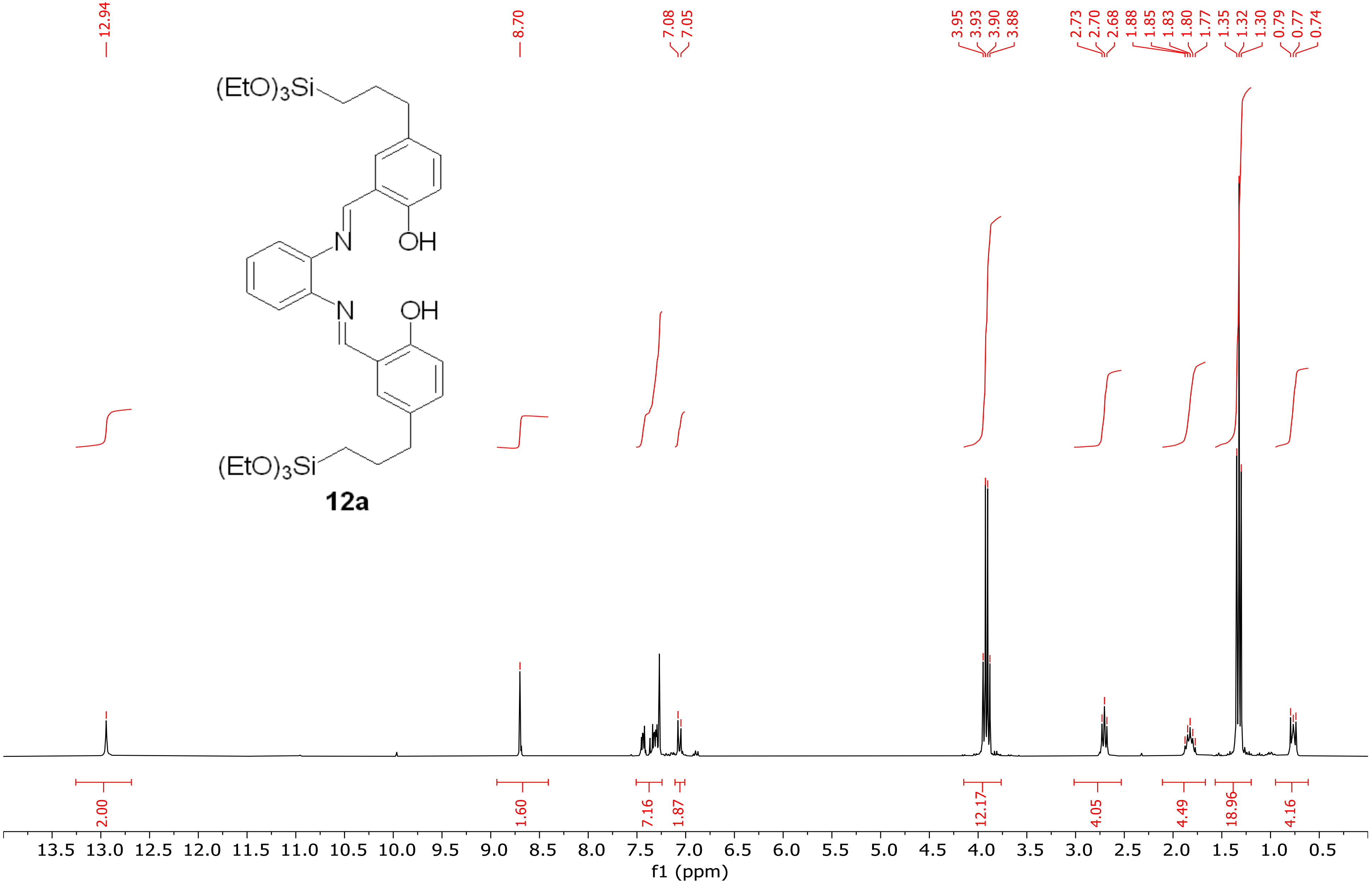

$^{13}C\{^{1}H\}$ NMR Spectrum (75 MHz, $CDCl_3$)

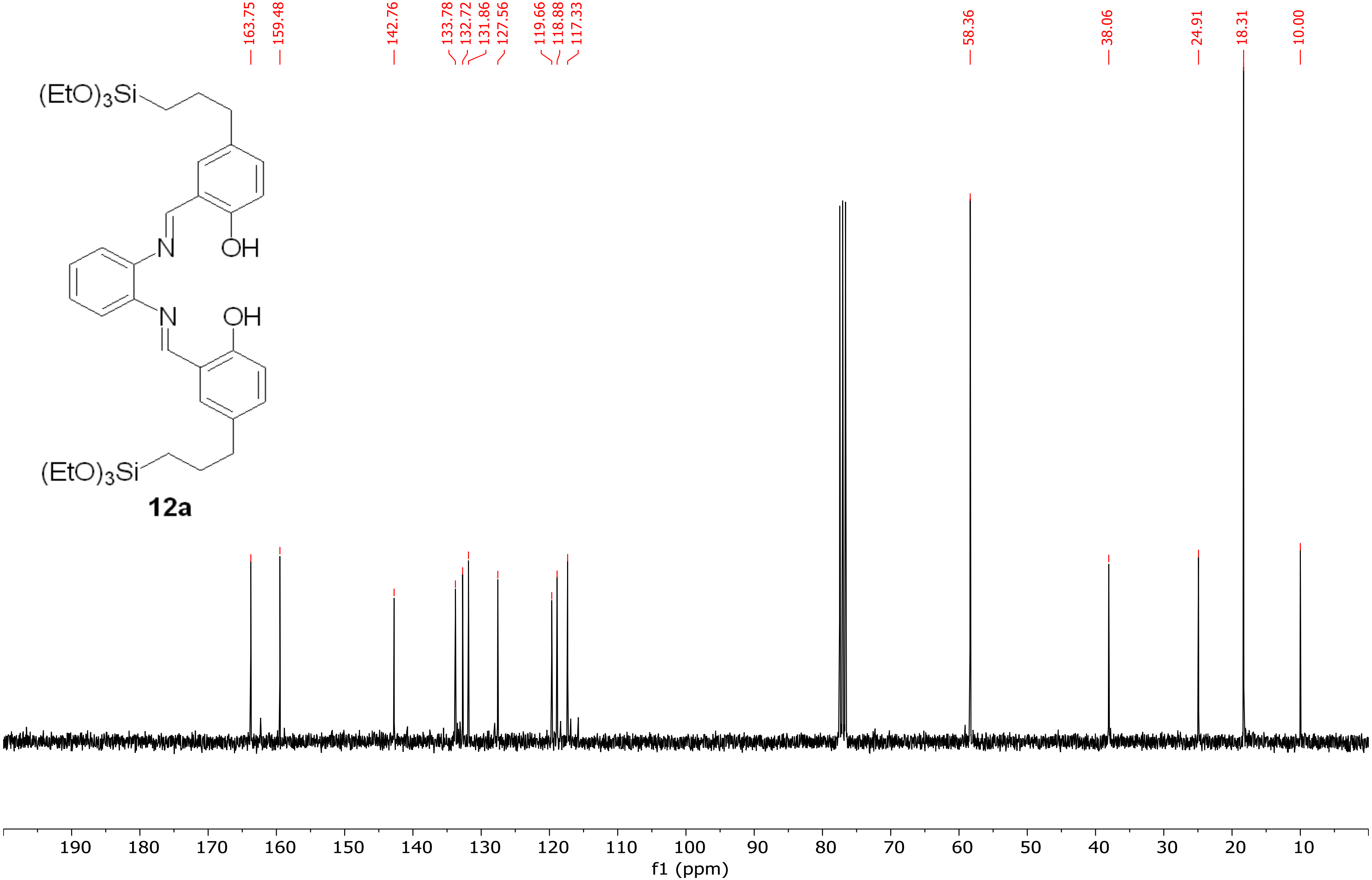

***N*,*N'*-Bis(2-hydroxy-5-(3-triethoxysilylpropyl)benzylidene)-1,2-diaminoethane (12b)**

<u>IR spectrum</u>

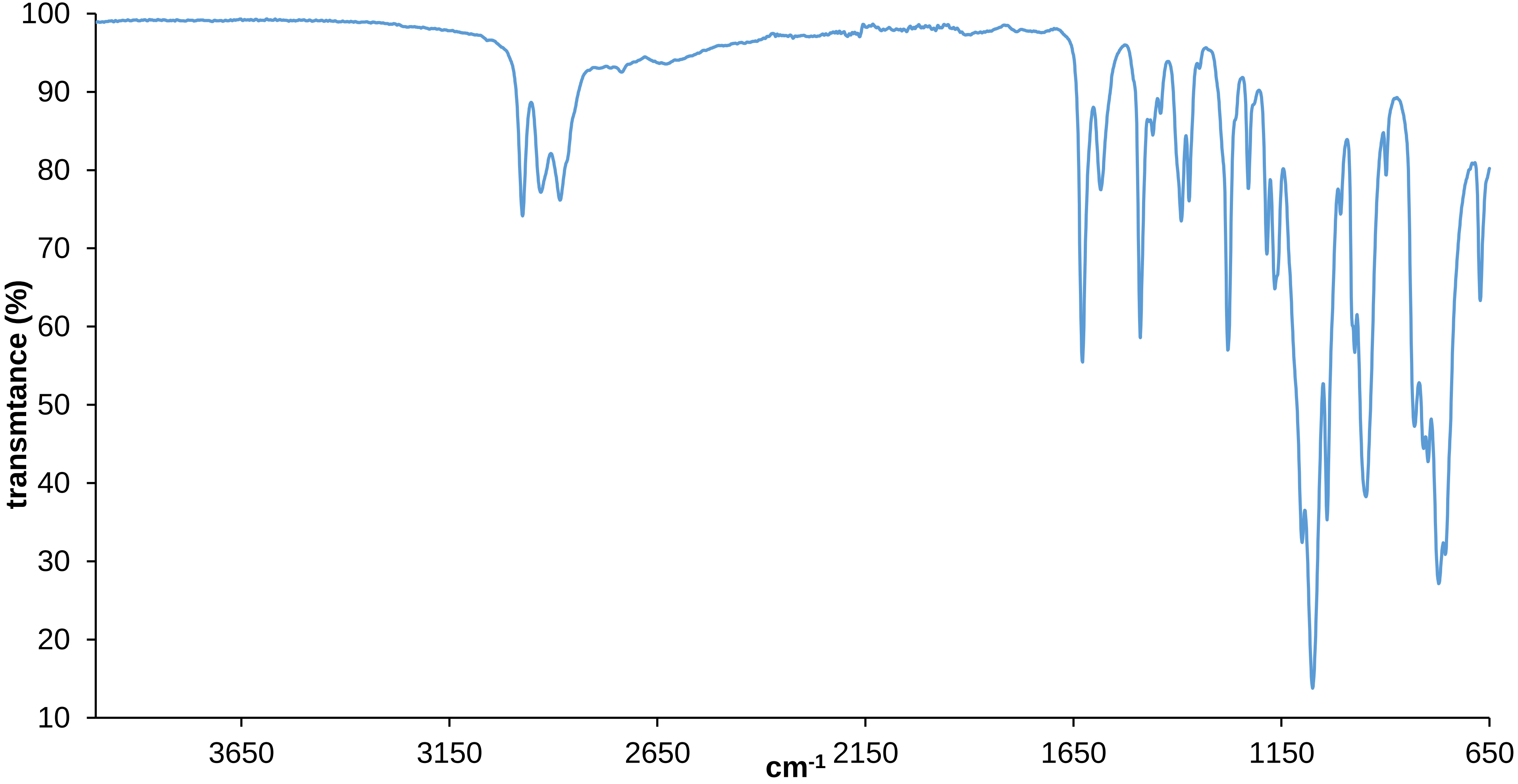

^{1}H NMR Spectrum (300 MHz, $CDCl_3$)

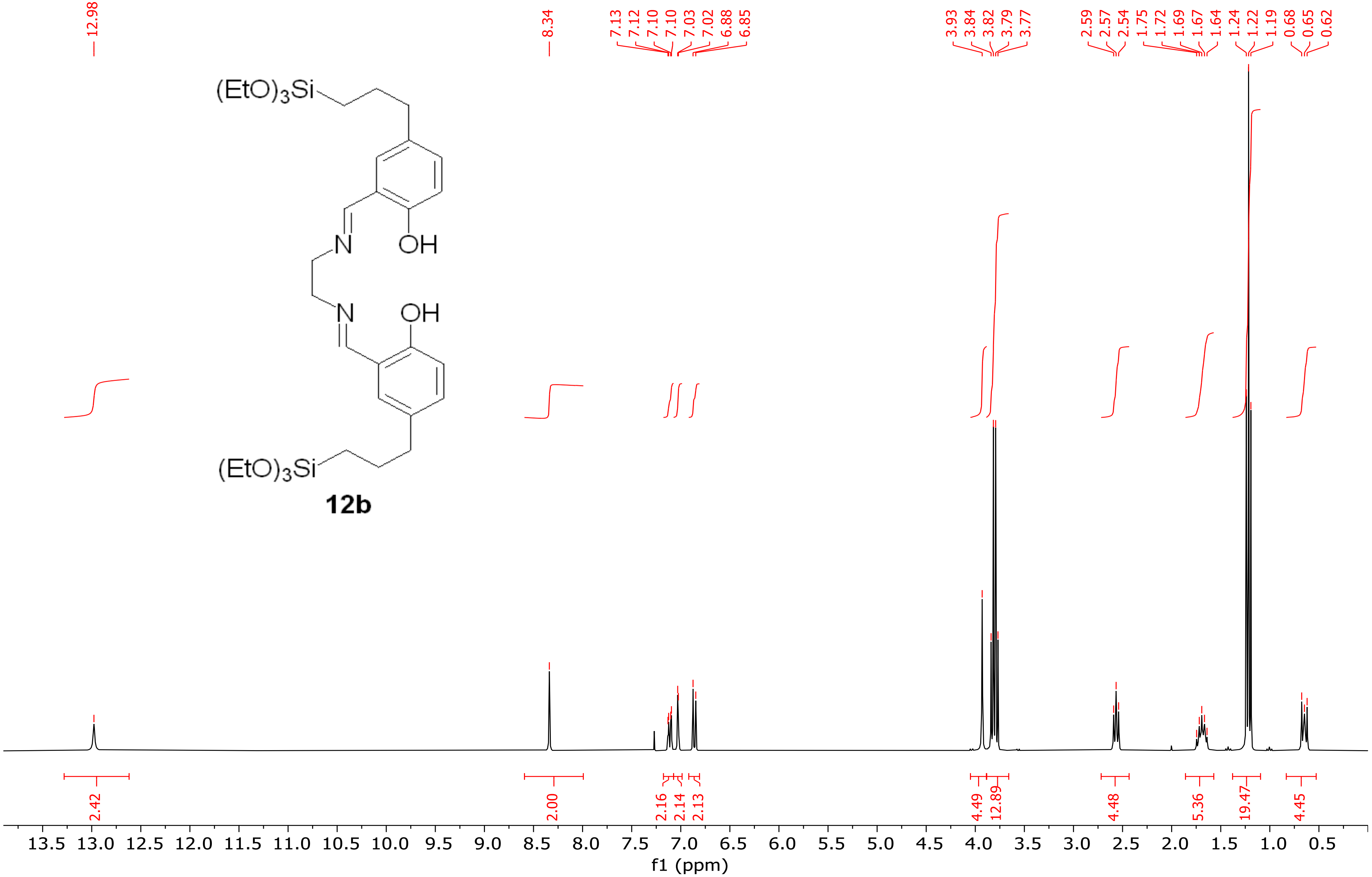

$^{13}C\{^1H\}$ NMR Spectrum (75 MHz, $CDCl_3$)

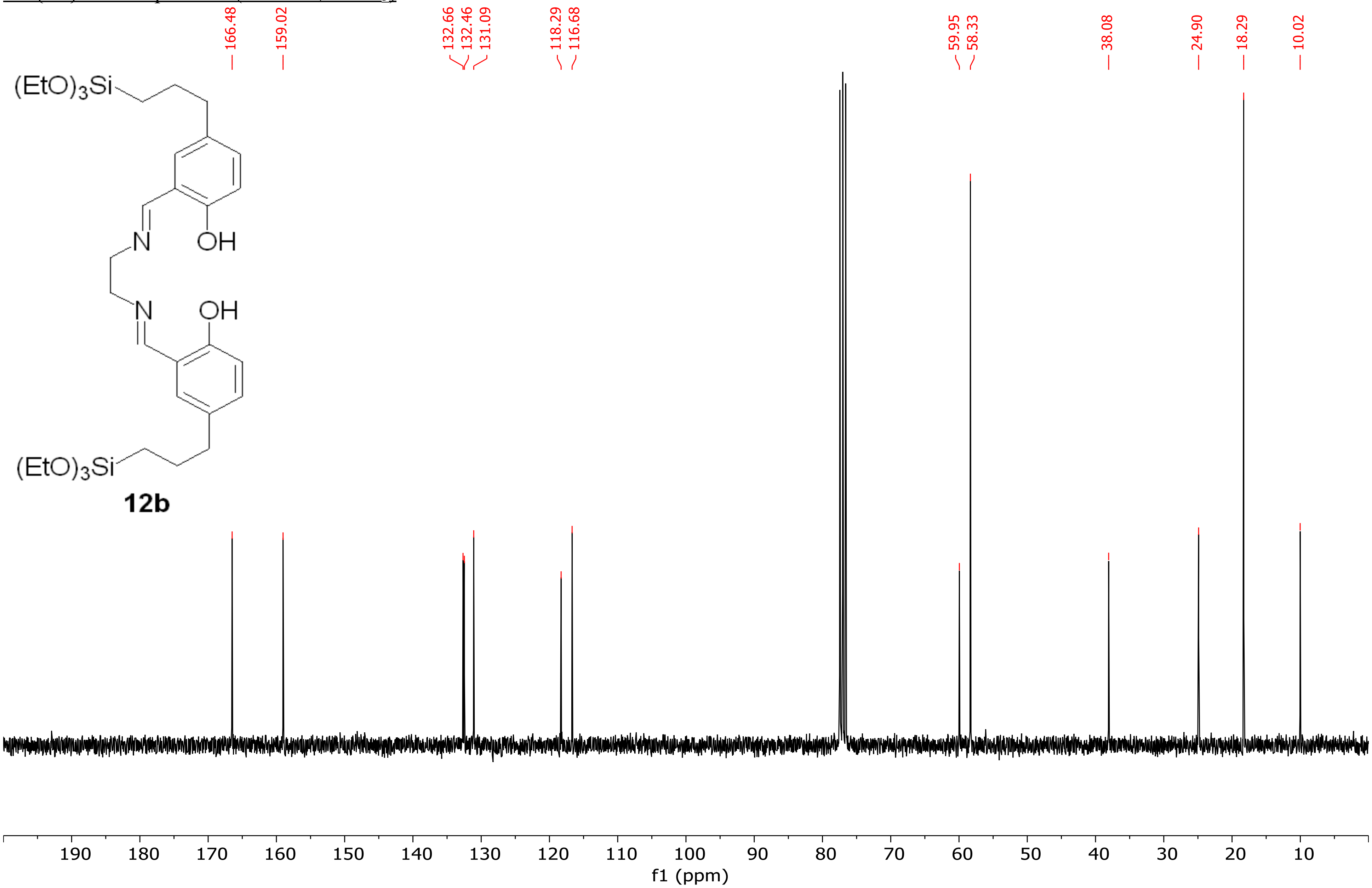

N,N'-Bis(2-hydroxy-5-(3-triethoxysilylpropyl)benzylidene)-*trans*-1,2-diaminocyclohexane (12c)

IR spectrum

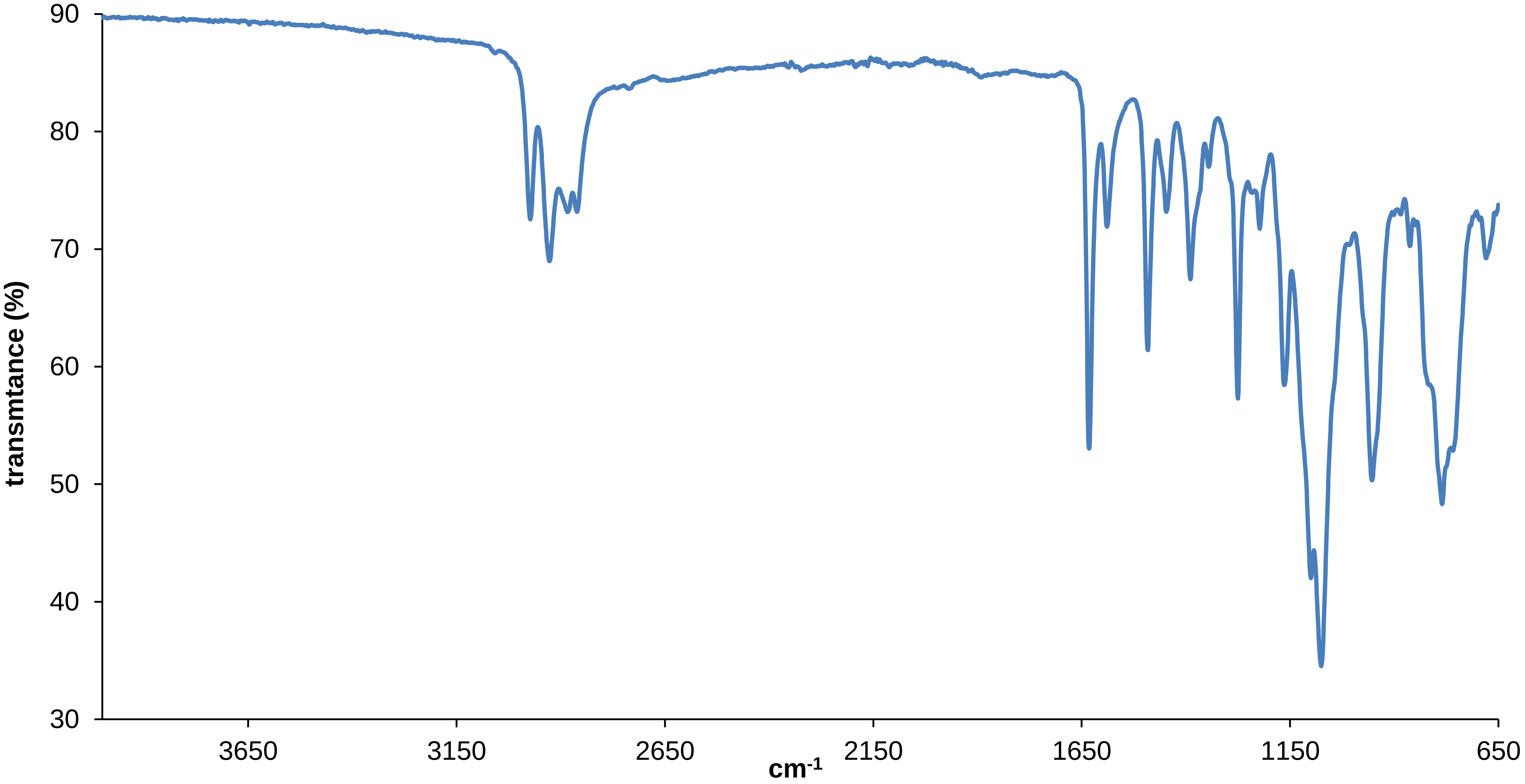

^{1}H NMR Spectrum (300 MHz, $CDCl_3$)

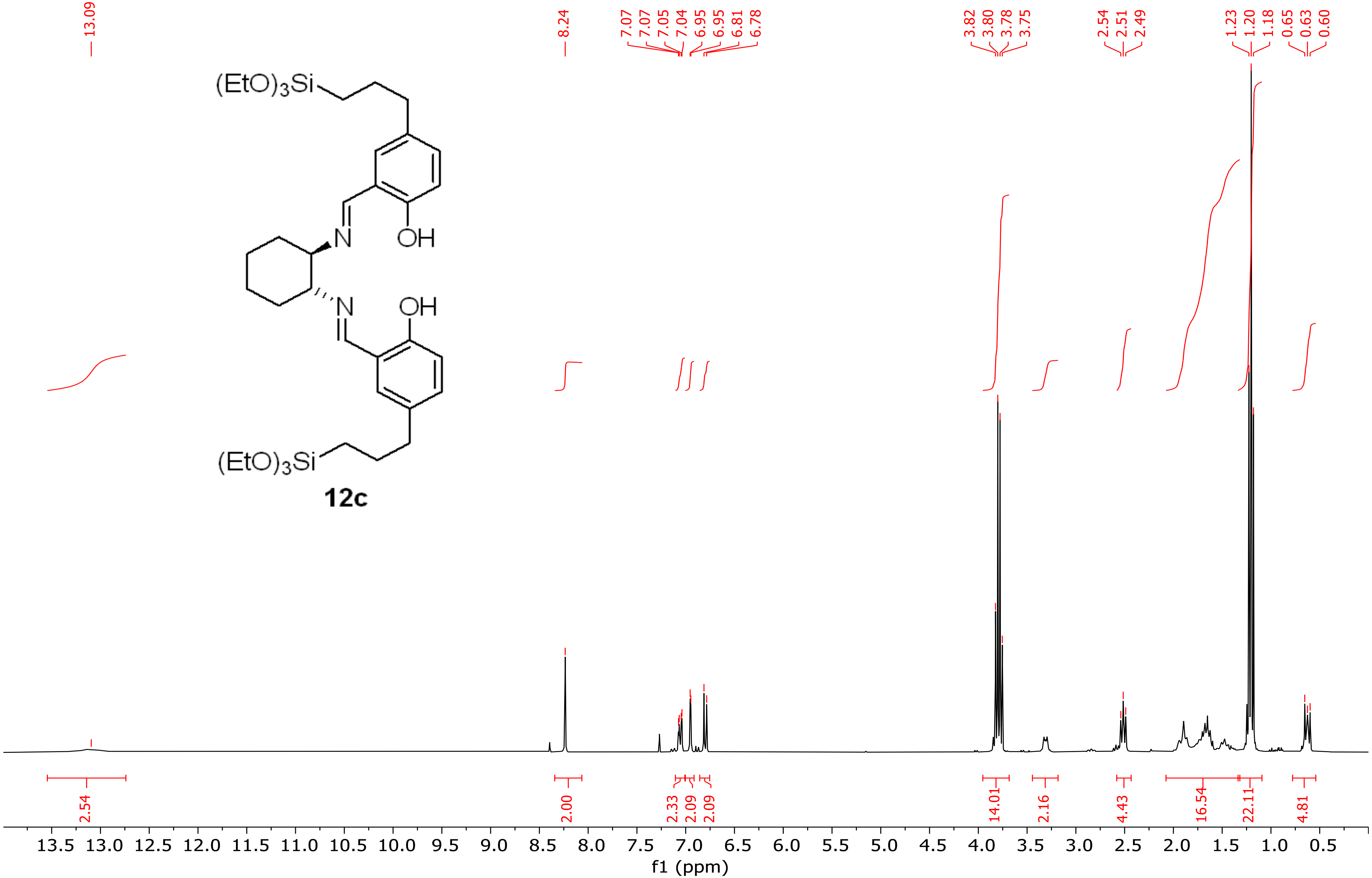

12c

$^{13}C\{^{1}H\}$ NMR Spectrum (75 MHz, $CDCl_3$)

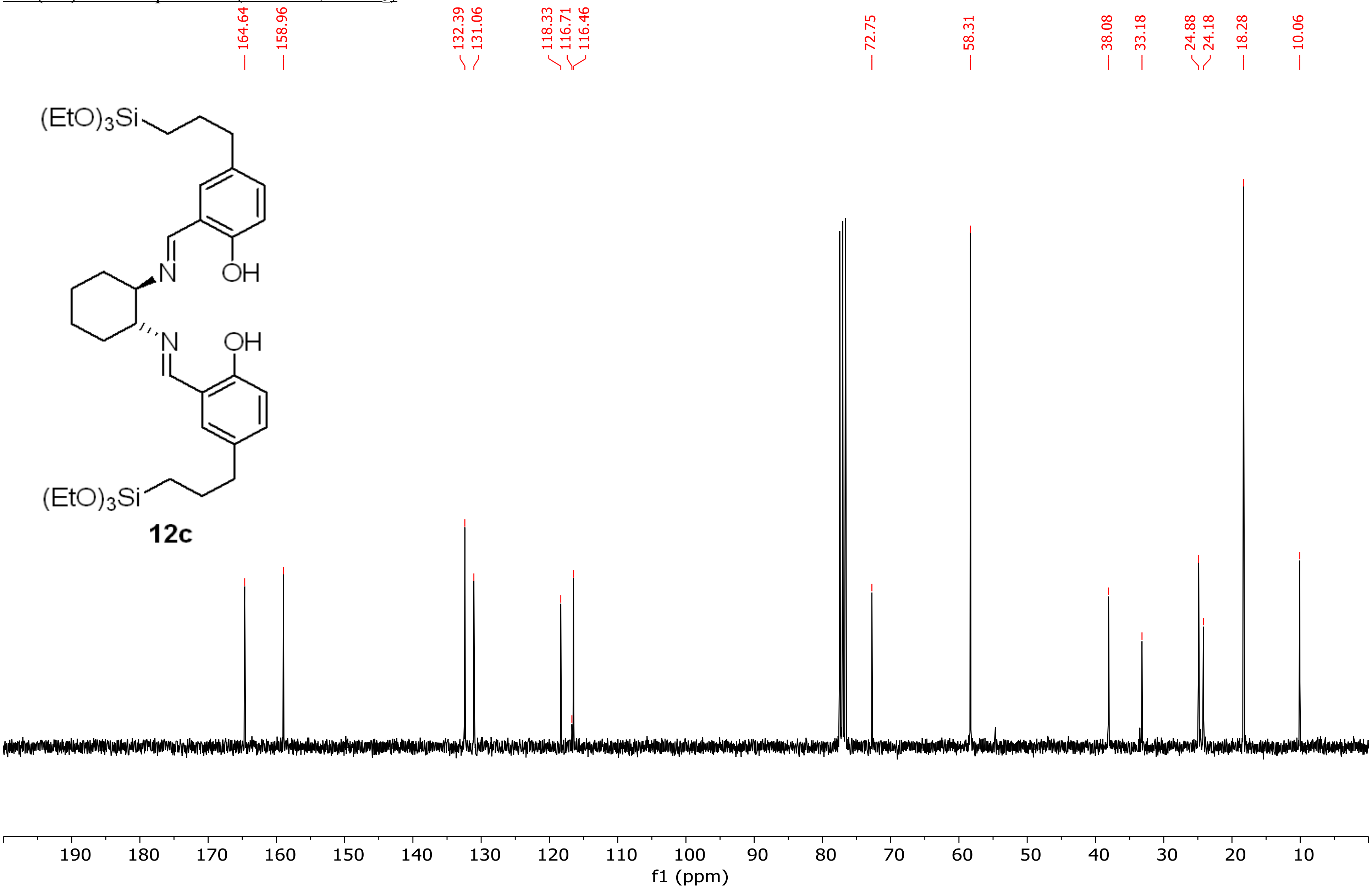

Silica-supported salophen 13a

<u>Analysis</u>

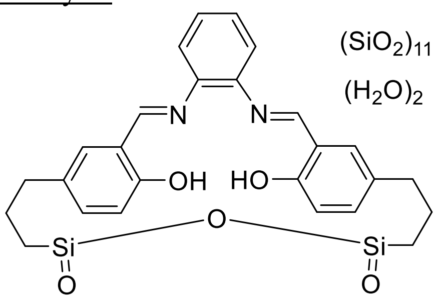

Chemical Formula: $C_{26}H_{30}N_2O_{29}Si_{13}$
Molecular Weight: 1199.62
Elemental Analysis: C, 26.03; H, 2.52; N, 2.34

Found: C, 25.9; H, 2.6; N, 2.2%

Mass of water:2x18 = 36
So predicted %water: = 3.0%
TGA weight loss below 100 ºC: = 3.0%

%yield calculation:
Product should contain
0.375 mmol of salophen unit with RMM of 502 = 502 x 0.375 mg = 188.25 mg
4.125 mmol of SiO_2 with RMM of 60 = 60 x 4.125 mg = 247.5 mg
0.75 mmol of H_2O with RMM of 18 = 18 x 0.75 mg = 13.5 mg
So 100% yield = 449.25 mg

Actual mass of product = 380 mg
So %yield = 85%

Loading calculation
1.200 g contains 1 mmol of salophen
So loading is: 1/1.2 = 0.83 mmol of salophen per gram

Thermogravimetric Analysis

Full trace

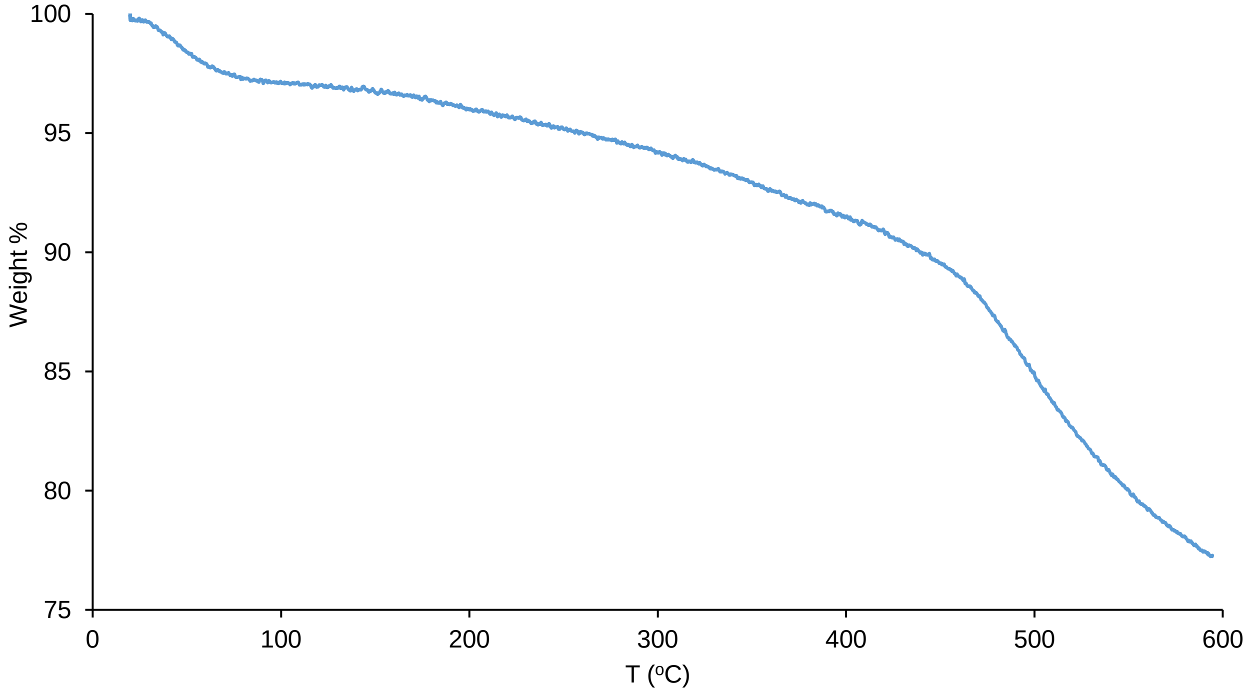

Expansion of region below 150 ºC

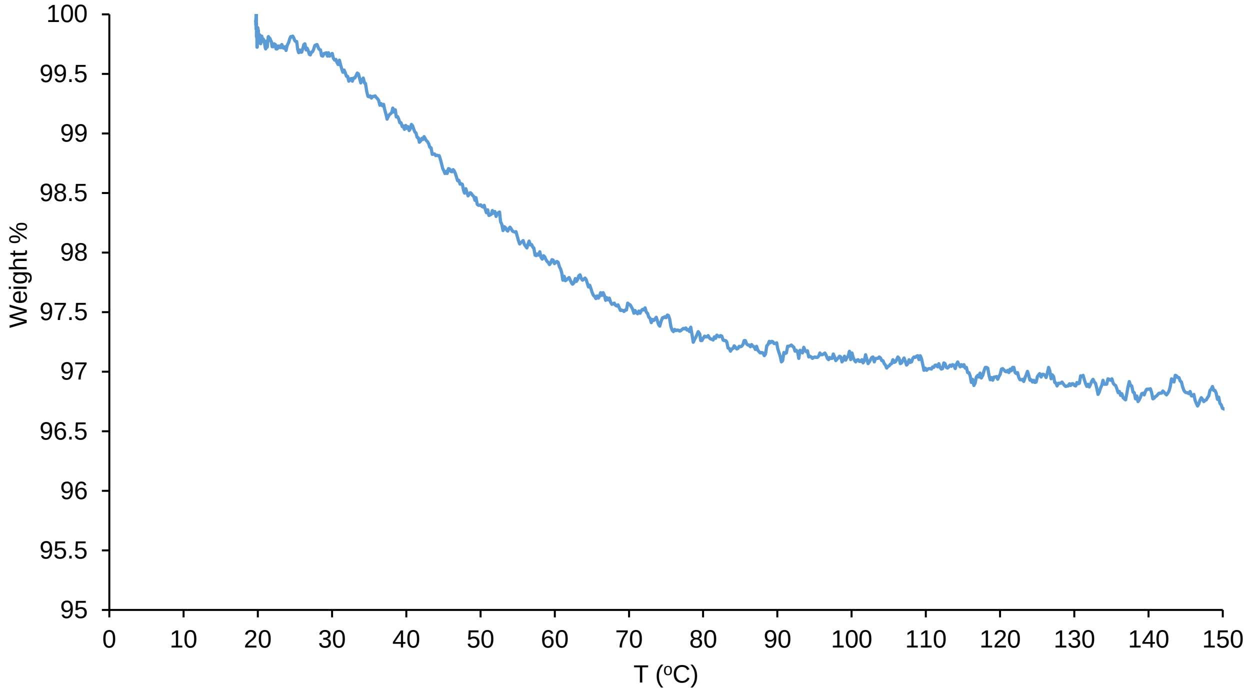

IR spectrum

Full spectrum

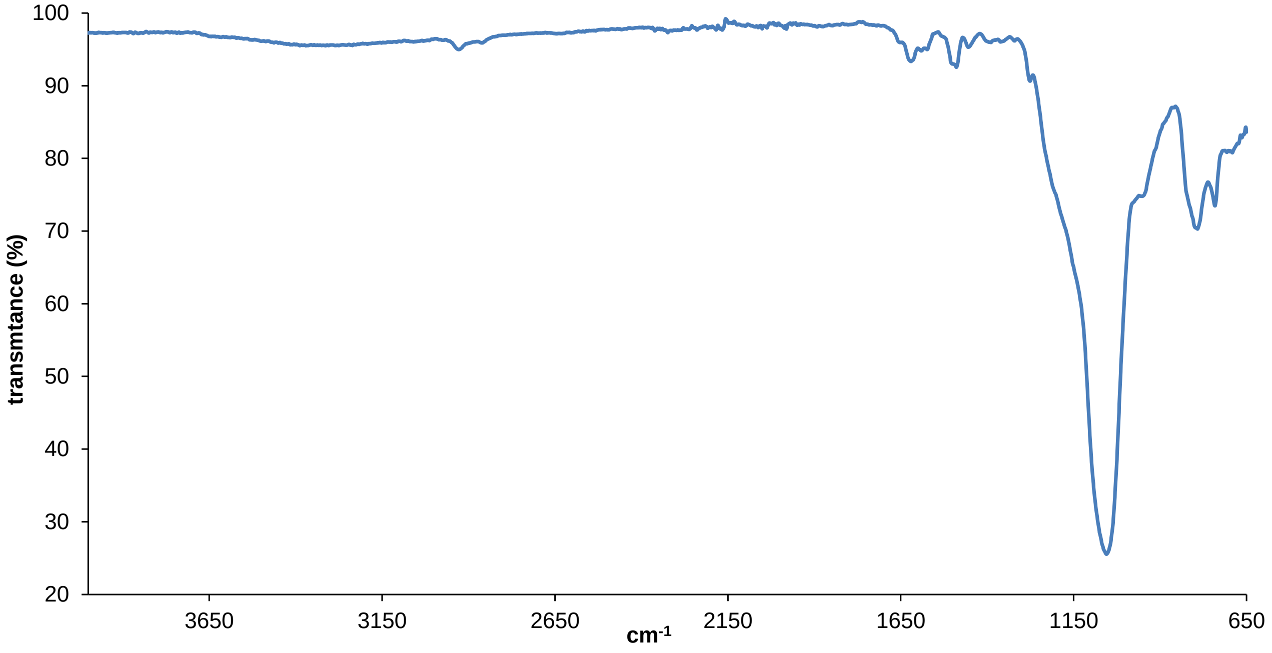

Expansion of 4000-1400 cm^{-1}

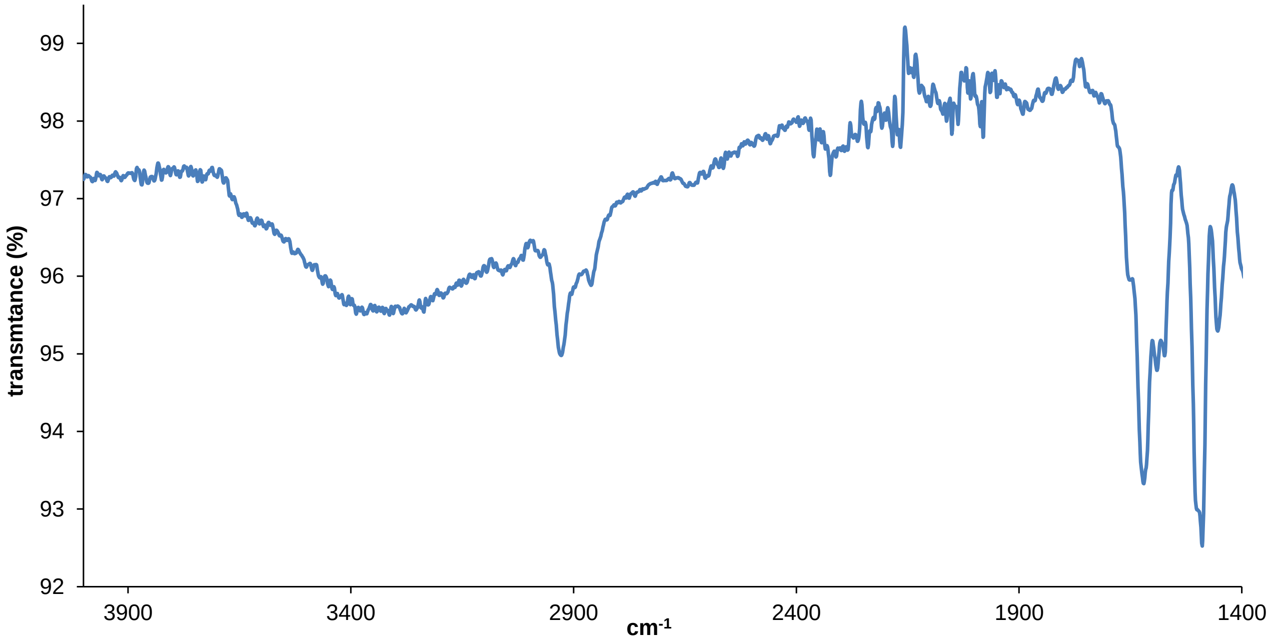

Solid State $^{13}C\{^{1}H\}$ NMR Spectrum (100 MHz)

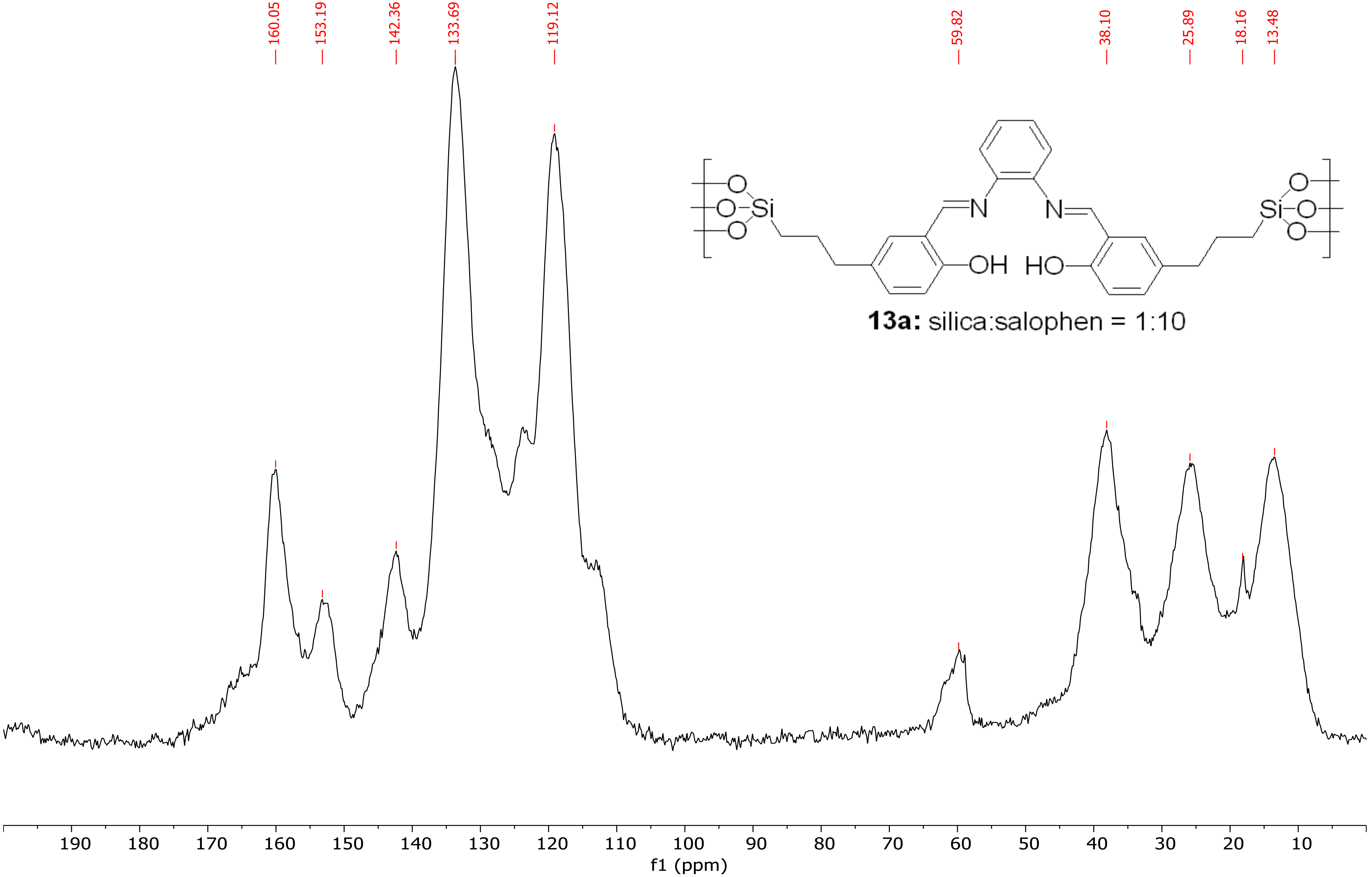

Silica-supported salen 13b

Analysis

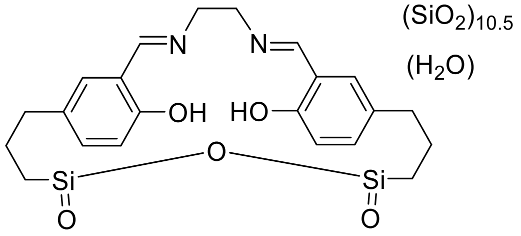

Chemical Formula: $C_{22}H_{28}N_2O_{27}Si_{12.5}$
Molecular Weight: 1103.52
Elemental Analysis: C, 23.95; H, 2.56; N, 2.54

Found: C, 23.8; H, 2.7; N, 2.2%.

Mass of water: 1x18 = 18
So predicted %water: = 1.6%
TGA weight loss below 100 °C: = 1.5%

% yield calculation:
Product should contain
0.375 mmol of salen unit with RMM of 454 = 454 x 0.375 mg = 170.25 mg
3.938 mmol of SiO_2 with RMM of 60 = 60 x 3.938 mg = 236.25 mg
0.375 mmol of H_2O with RMM of 18 = 18 x 0.375 mg = 6.75 mg
So 100% yield = 413.25 mg

Actual mass of product = 273 mg
So %yield = 66%

Loading calculation
1.104 g contains 1 mmol of salen
So loading is: 1 / 1.104 = 0.91 mmol of salen per gram

Thermogravimetric Analysis

Full trace

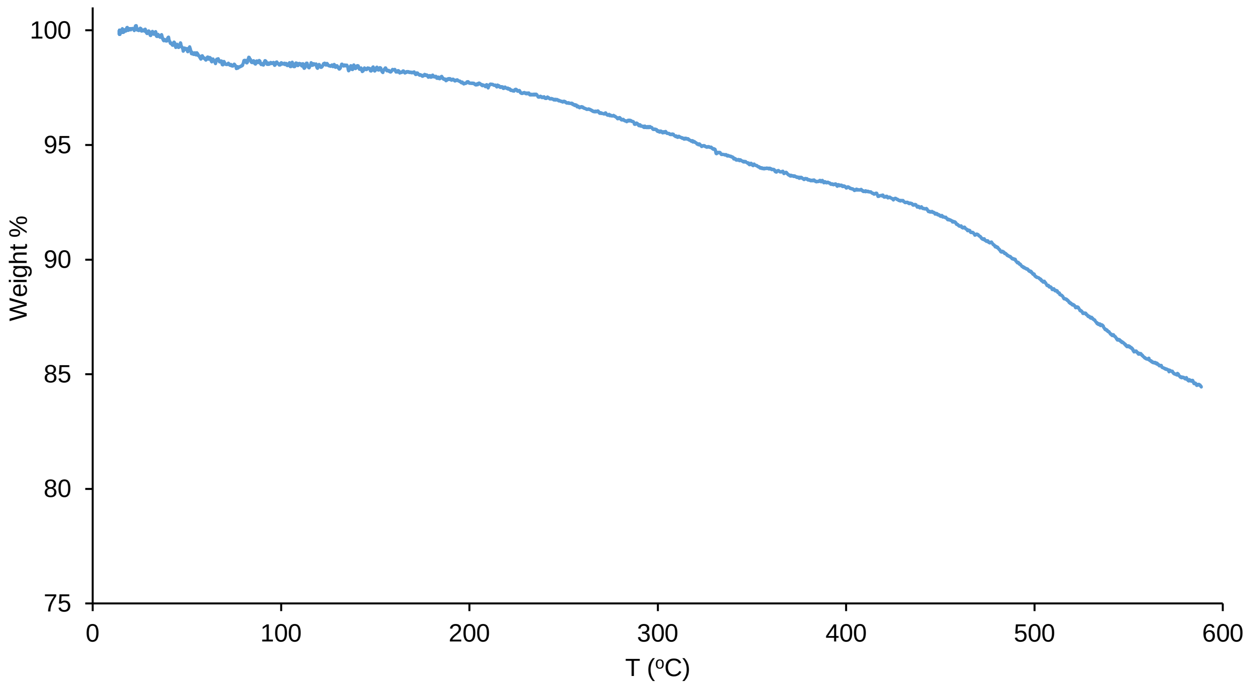

Expansion of region below 150 ºC

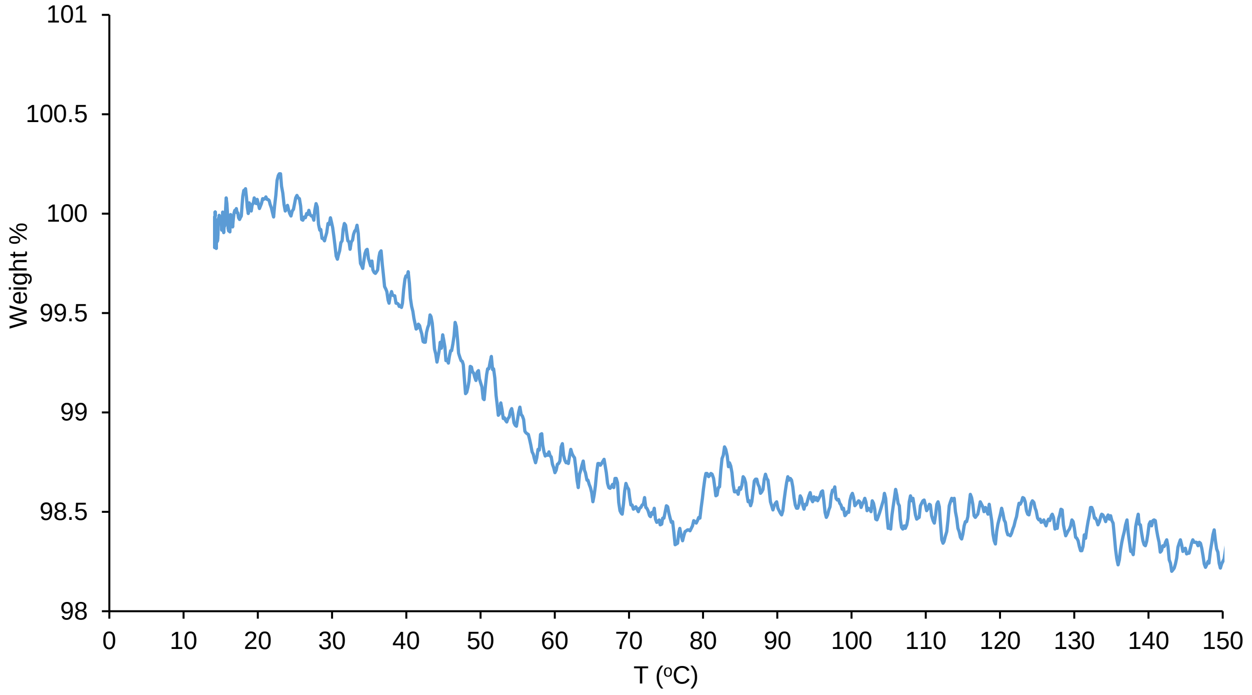

IR spectrum

Full spectrum

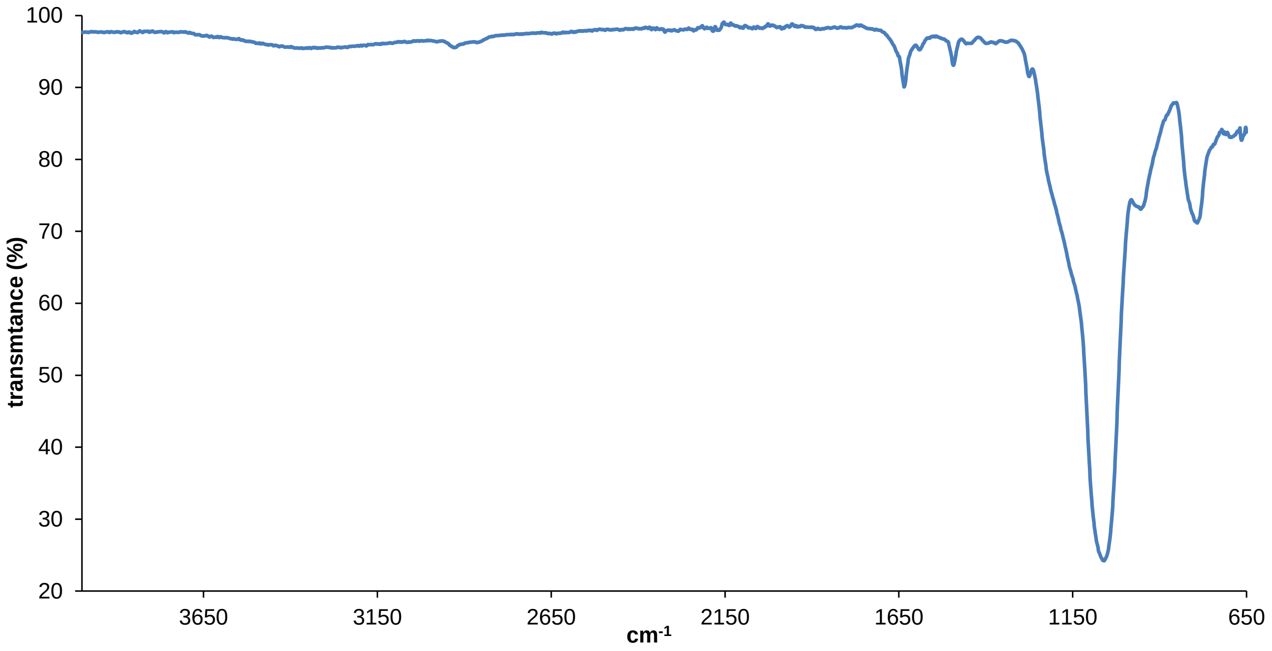

Expansion of 4000-1400 cm^{-1}

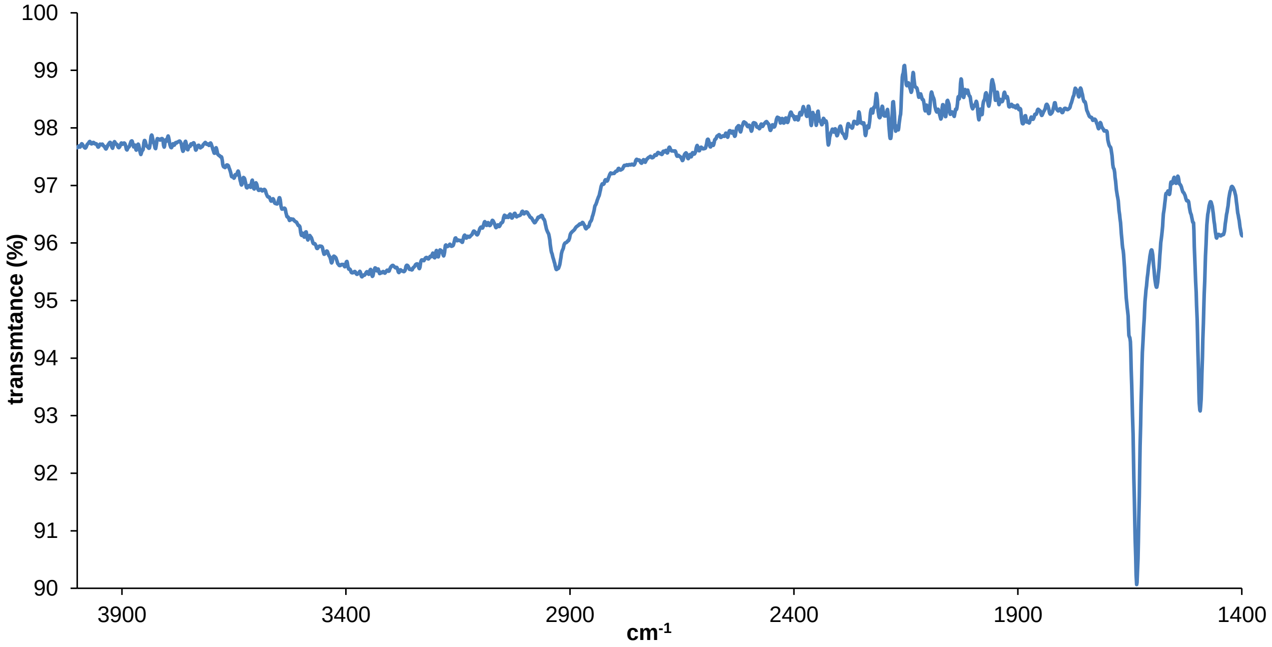

Solid State $^{13}C\{^1H\}$ NMR Spectrum (100 MHz)

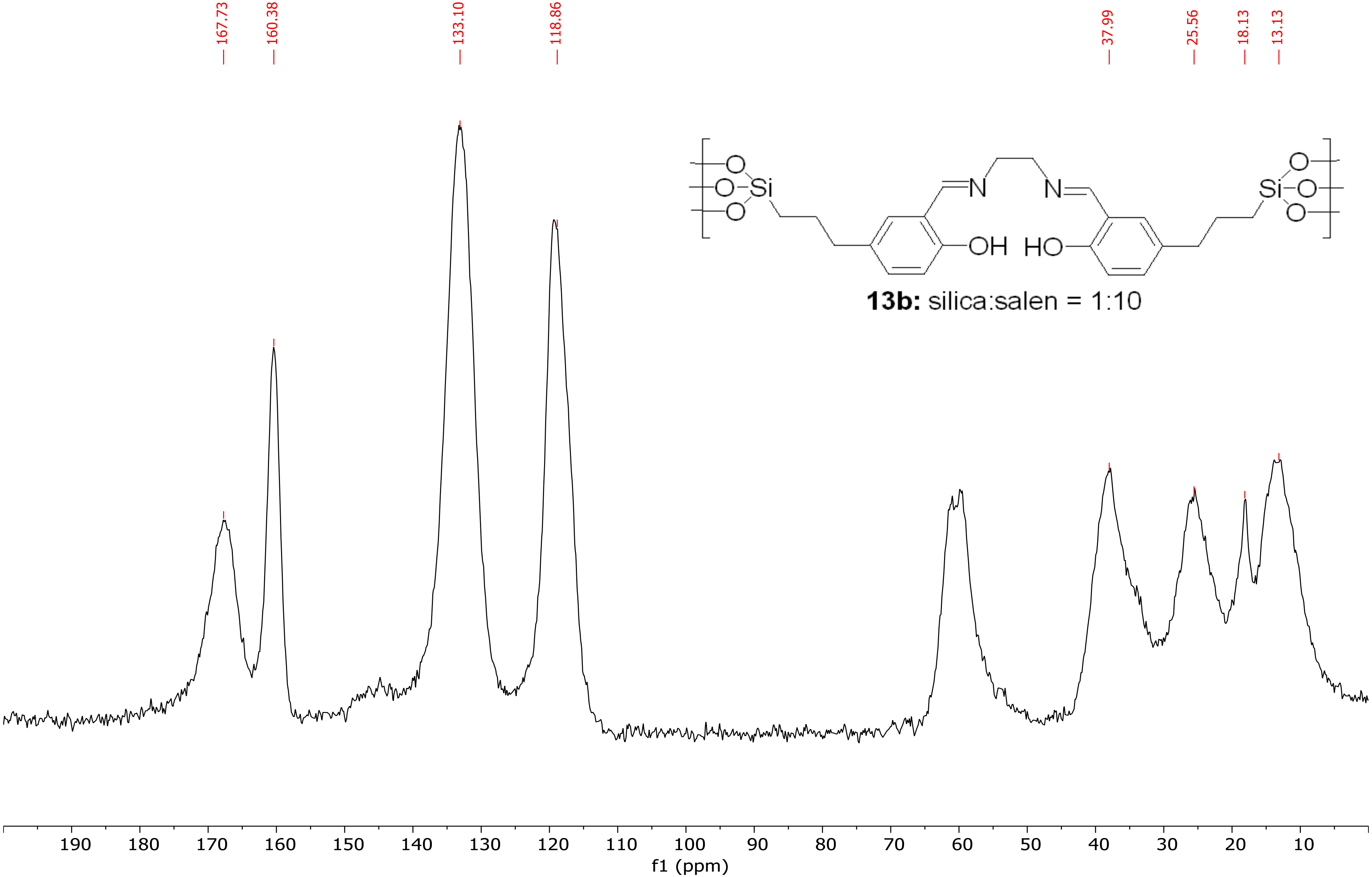

Silica-supported salen 13c

<u>Analysis</u>

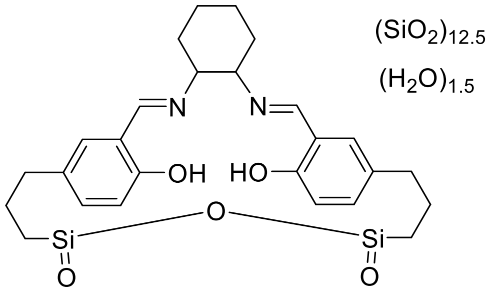

Chemical Formula: $C_{26}H_{35}N_2O_{31.5}Si_{14.5}$
Molecular Weight: 1286.78
Elemental Analysis: C, 24.27; H, 2.74; N, 2.18%

Found: C, 24.2; H, 2.9; N, 1.9%.

Mass of water: 1.5x18 = 27
So predicted %water: = 2.1%
TGA weight loss below 100 °C: = 2.0%

% yield calculation:
Product should contain
0.375 mmol of salen unit with RMM of 508 = 508 x 0.375 mg = 190.5 mg
4.69 mmol of SiO_2 with RMM of 60 = 60 x 4.688 mg = 281.25 mg
0.563 mmol of H_2O with RMM of 18 = 18 x 0.563 mg = 10.1 mg
So 100% yield = 481.9 mg

Actual mass of product = 400 mg
So %yield = 83%

Loading calculation
1.287 g contains 1 mmol of salen
So loading is: 1 / 1.287 = 0.78 mmol of salen per gram

Thermogravimetric Analysis

Full trace

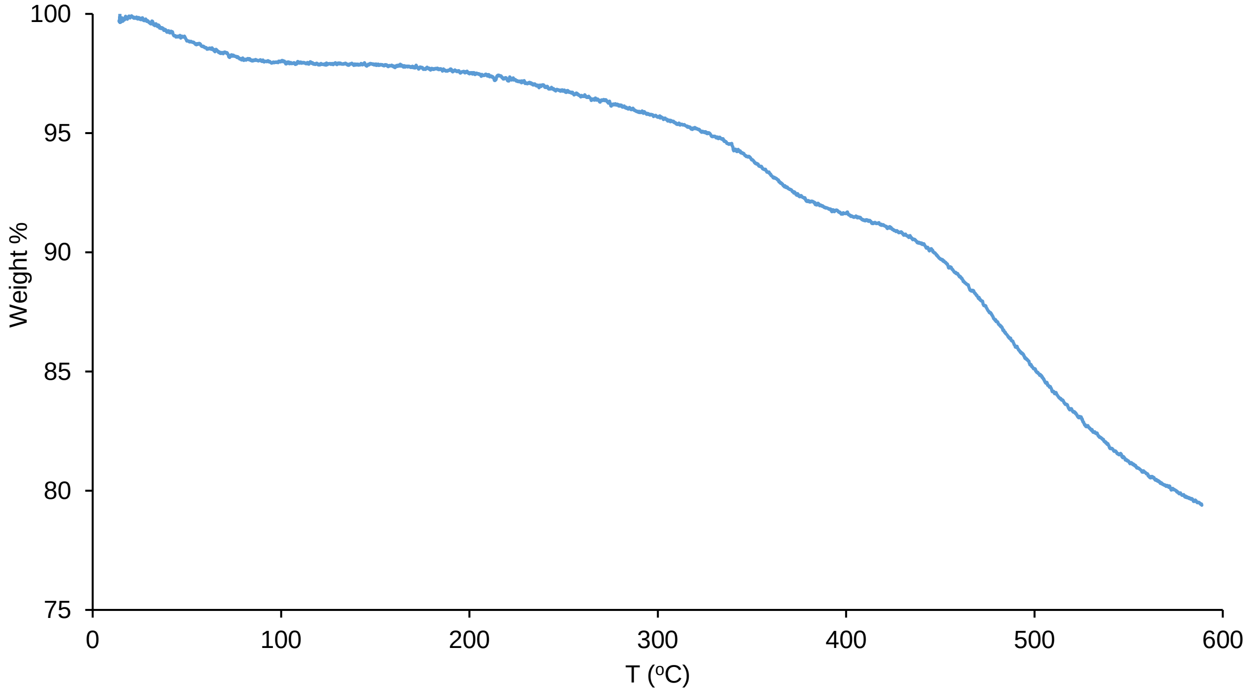

Expansion of region below 150 ºC

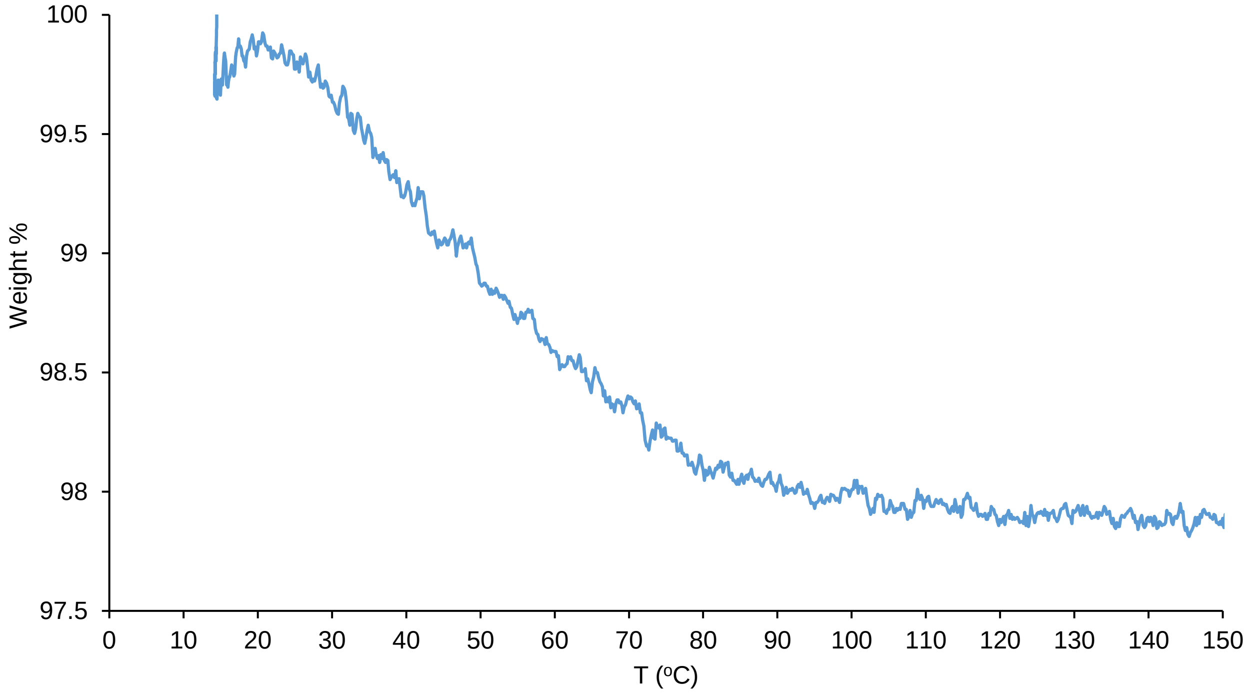

IR spectrum

Full spectrum

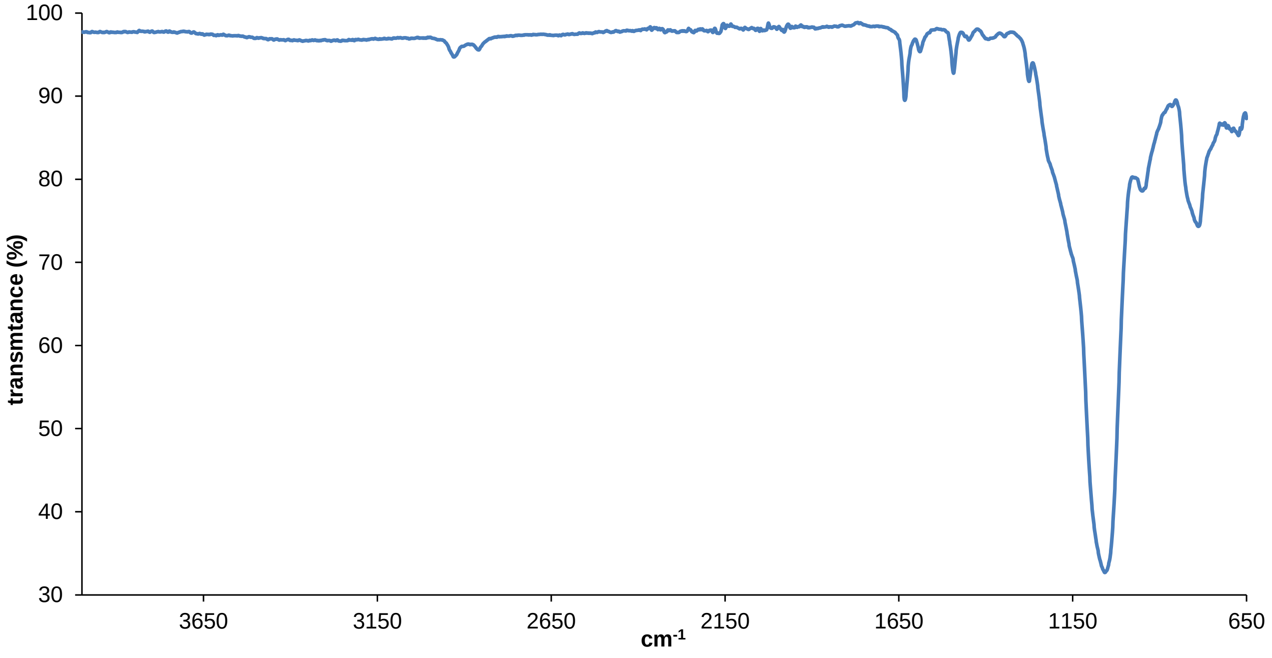

Expansion of 4000-1400 cm^{-1}

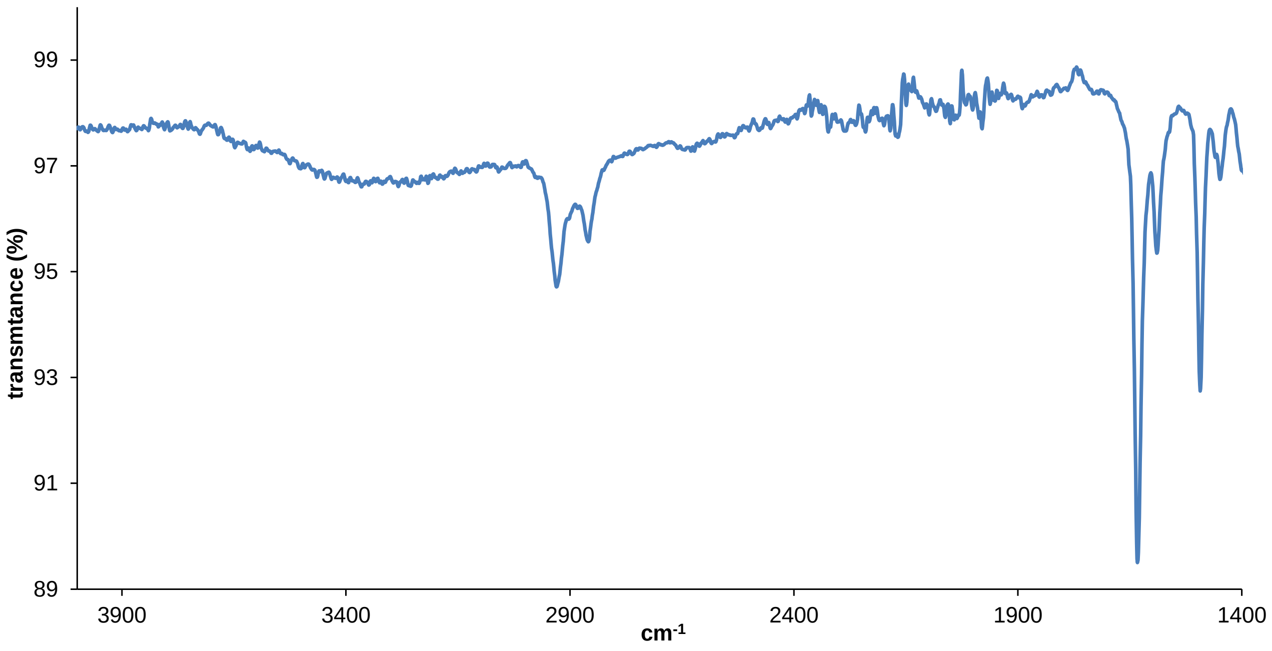

Solid State $^{13}C\{^{1}H\}$ NMR Spectrum (100 MHz)

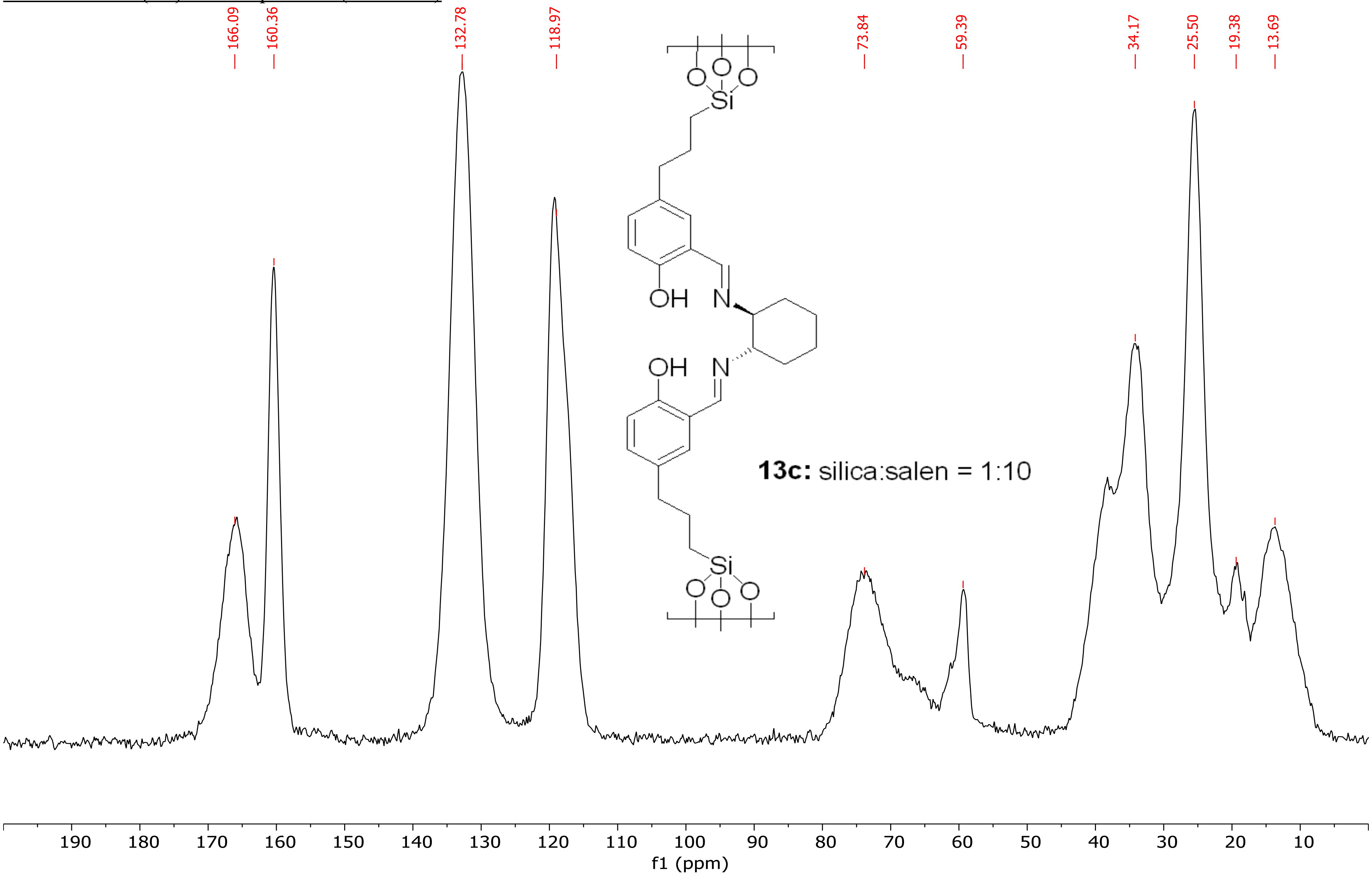

Silica-supported salophen 13d

Analysis

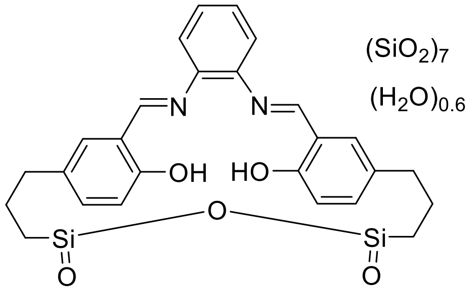

Chemical Formula: $C_{26}H_{27.2}N_2O_{19.6}Si_9$
Molecular Weight: 934.06
Elemental Analysis: C, 33.43; H, 2.94; N, 3.00%

Found: C, 33.4; H, 2.9; N, 3.0%.

Mass of water: 0.6x18 = 10.8
So predicted %water: = 1.1%
TGA weight loss below 100 °C: = 1.1%

% yield calculation:
Product should contain
0.35 mmol of salen unit with RMM of 503 = 503 x 0.35 mg = 176.1 mg
2.45 mmol of SiO_2 with RMM of 60 = 60 x 2.45 mg = 147 mg
0.21 mmol of H_2O with RMM of 18 = 18 x 0.21 mg = 3.8 mg
So 100% yield = 326.9 mg

Actual mass of product = 297 mg
So %yield = 91%

Loading calculation
0.934 g contains 1 mmol of salophen
So loading is: 1 / 0.934 = 1.07 mmol of salophen per gram

Thermogravimetric Analysis

Full trace

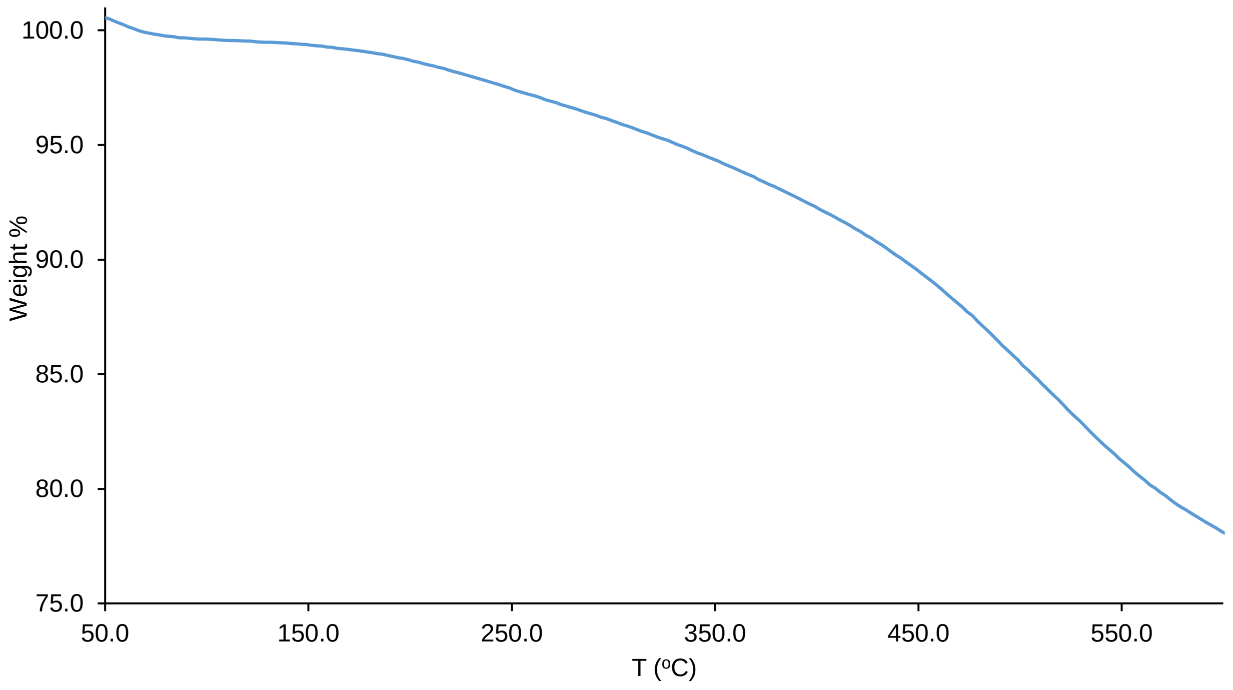

Expansion of region below 150 ºC

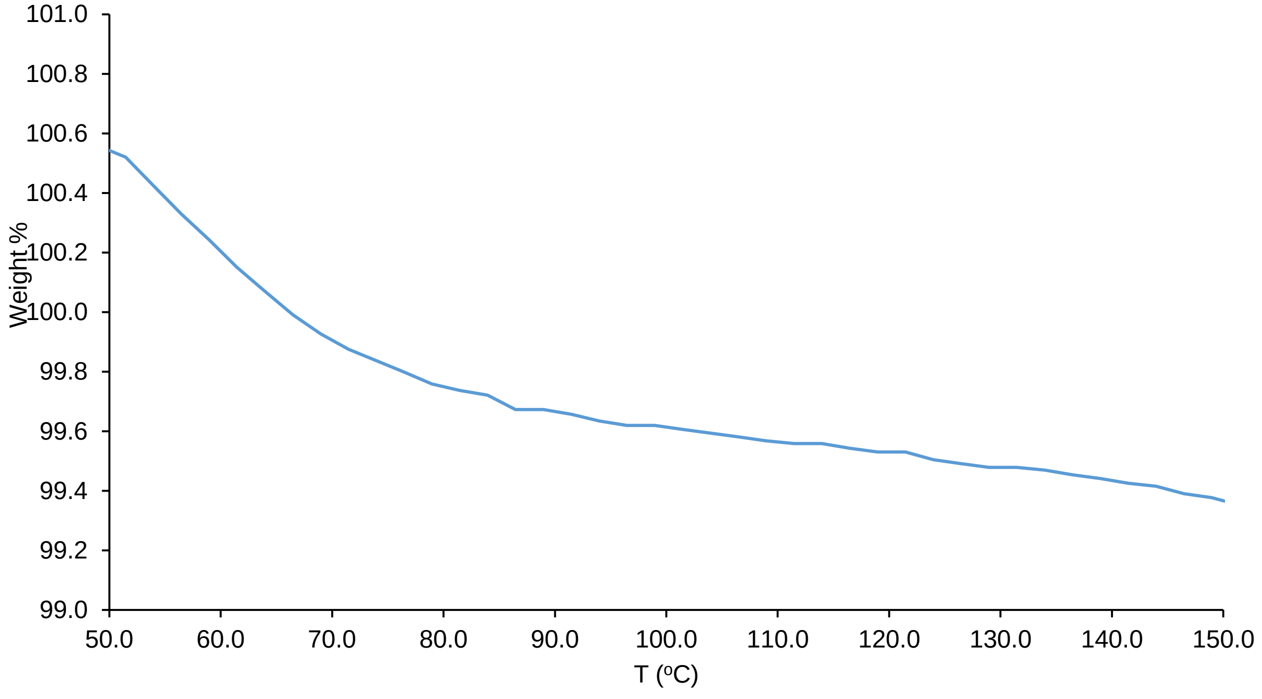

IR spectrum

Full spectrum

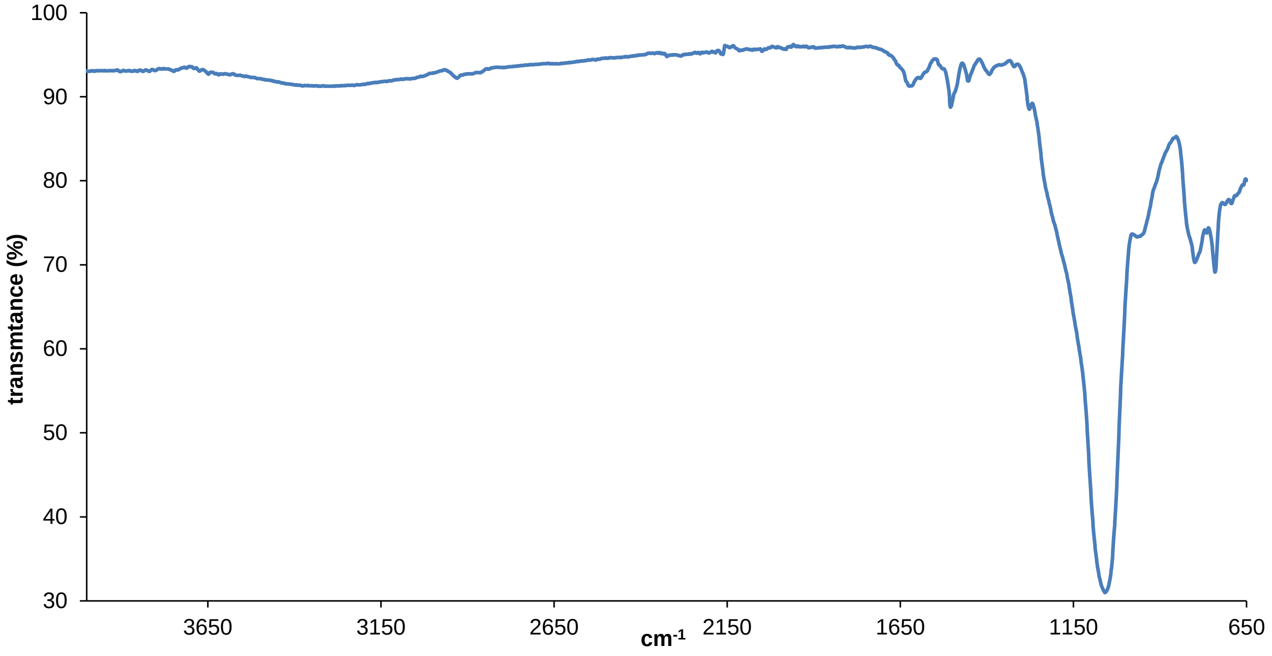

Expansion of 4000-1400 cm^{-1}

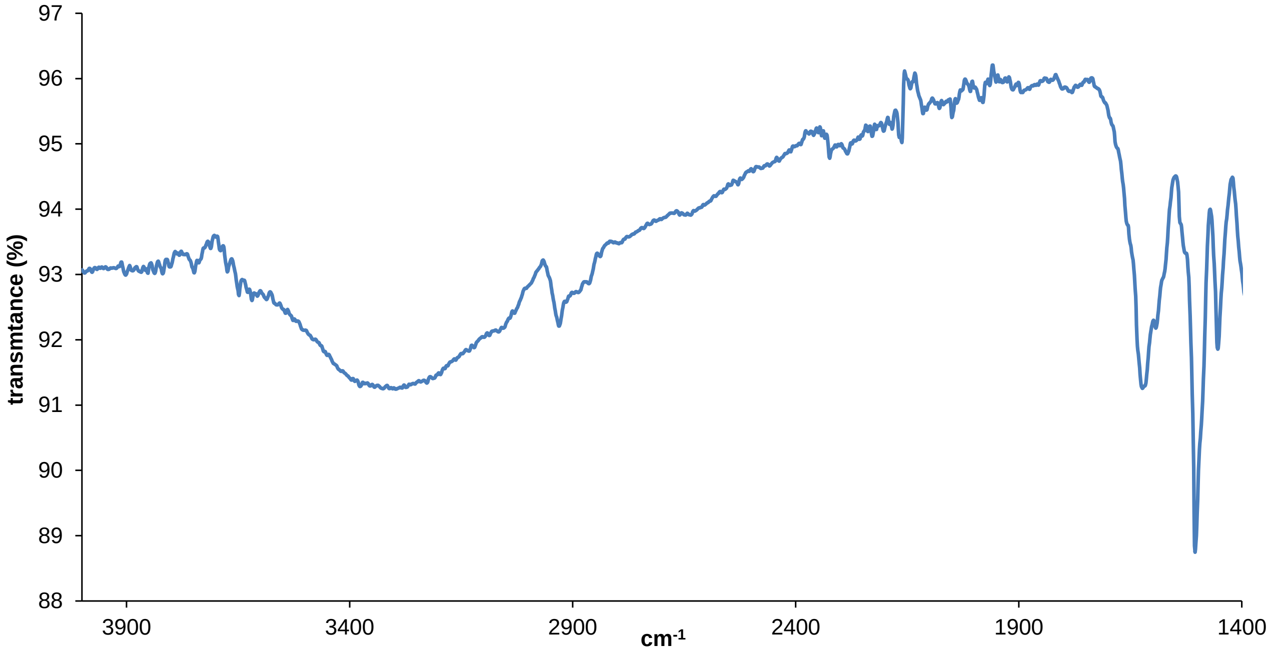

Solid State $^{13}C\{^1H\}$ NMR Spectrum (100 MHz)

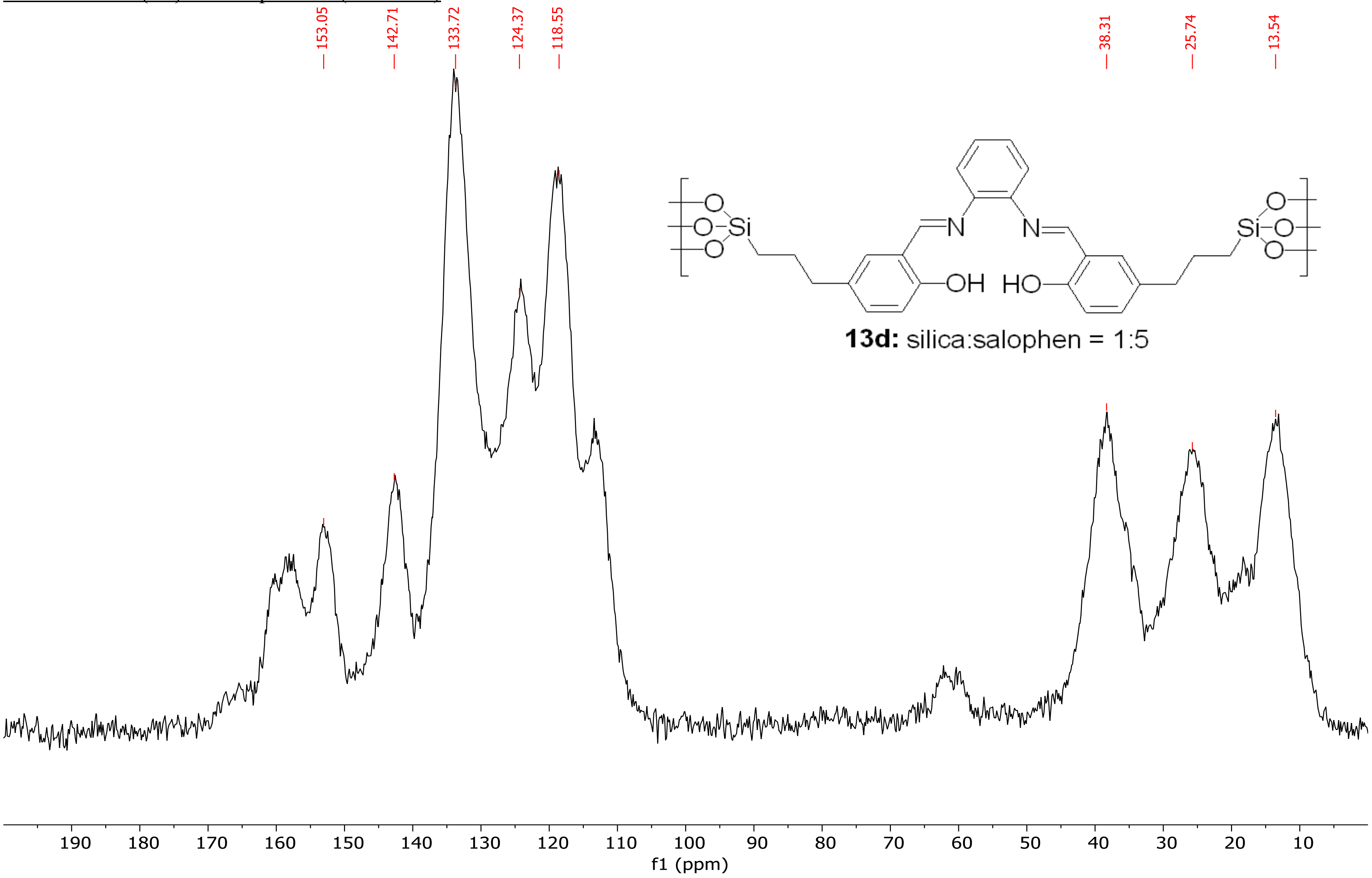

Silica-supported salophen 13e

Analysis

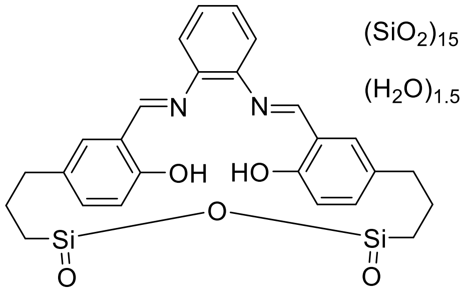

Chemical Formula: $C_{26}H_{29}N_2O_{36.5}Si_{17}$
Molecular Weight: 1430.94
Elemental Analysis: C, 21.82; H, 2.04; N, 1.96%

Found: C, 21.8; H, 2.4; N, 2.25%.

Mass of water: 1.5x18 = 27
So predicted %water: = 1.9%
TGA weight loss below 100 °C: = 1.8%

% yield calculation:
Product should contain
0.35 mmol of salen unit with RMM of 503 = 503 x 0.35 mg = 176.1 mg
5.25 mmol of SiO_2 with RMM of 60 = 60 x 5.25 mg = 315 mg
0.53 mmol of H_2O with RMM of 18 = 18 x 0.53 mg = 9.5 mg
So 100% yield = 500.6 mg

Actual mass of product = 431 mg
So %yield = 86%

Loading calculation
1.431 g contains 1 mmol of salophen
So loading is: 1 / 1.1431 = 0.70 mmol of salophen per gram

Thermogravimetric Analysis

Full trace

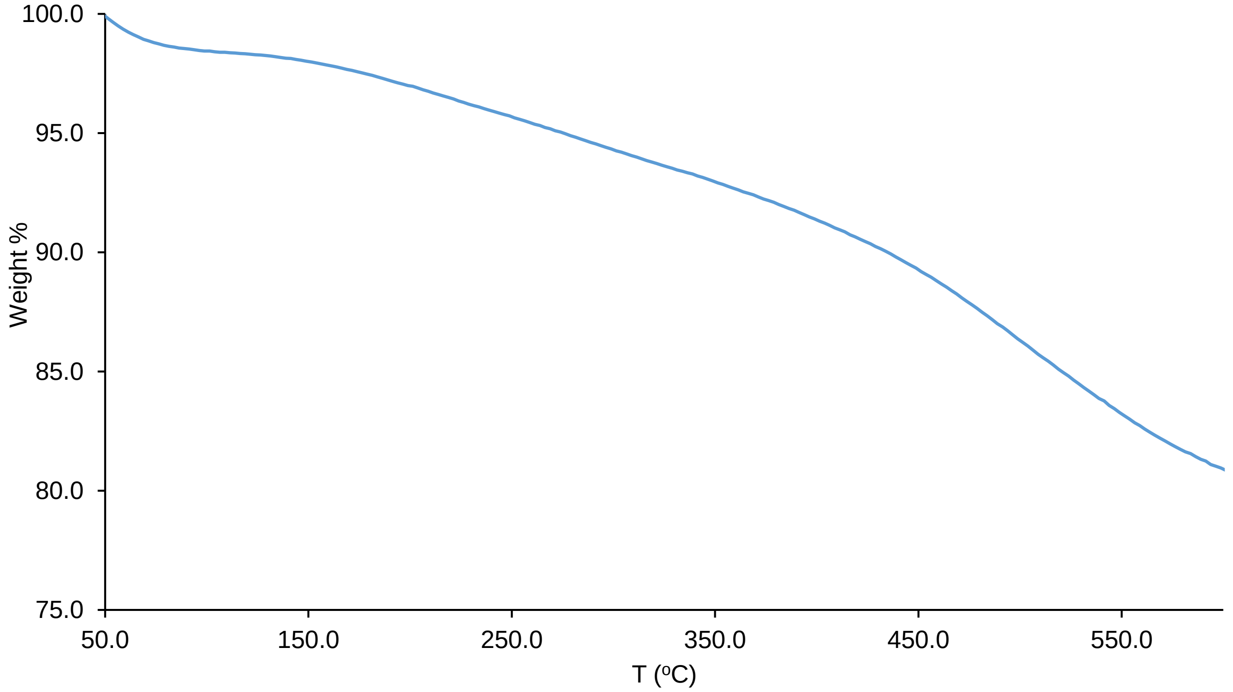

Expansion of region below 150 ºC

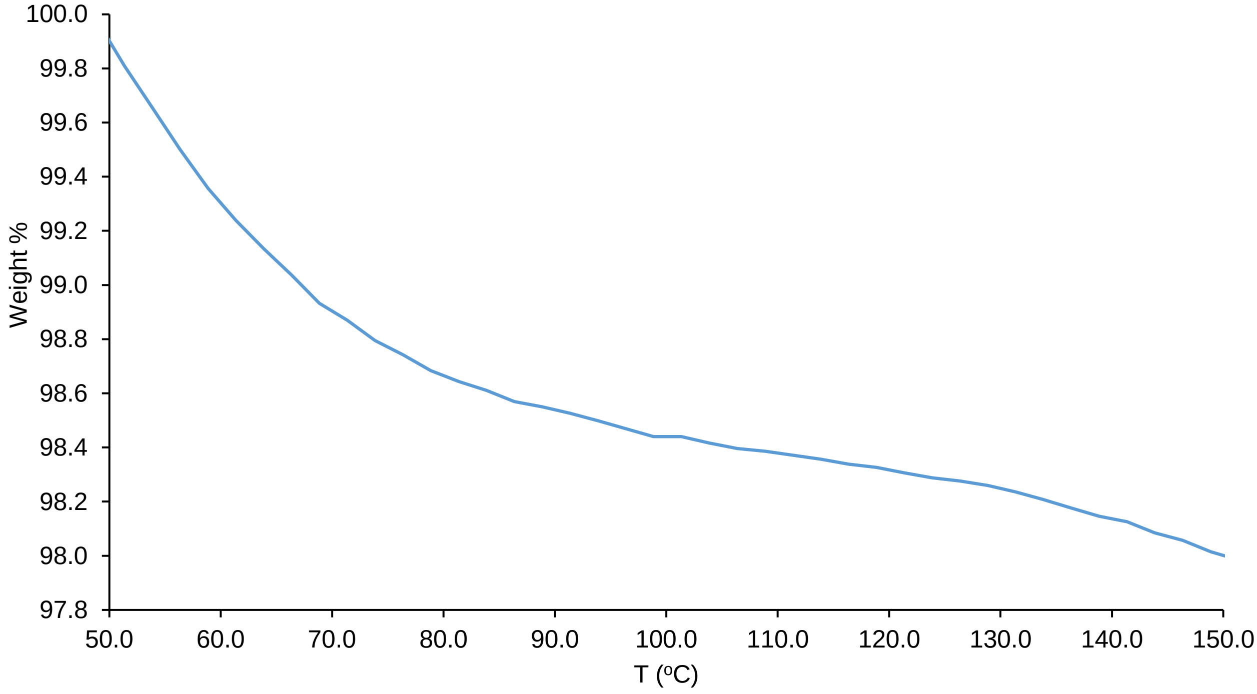

IR spectrum

Full spectrum

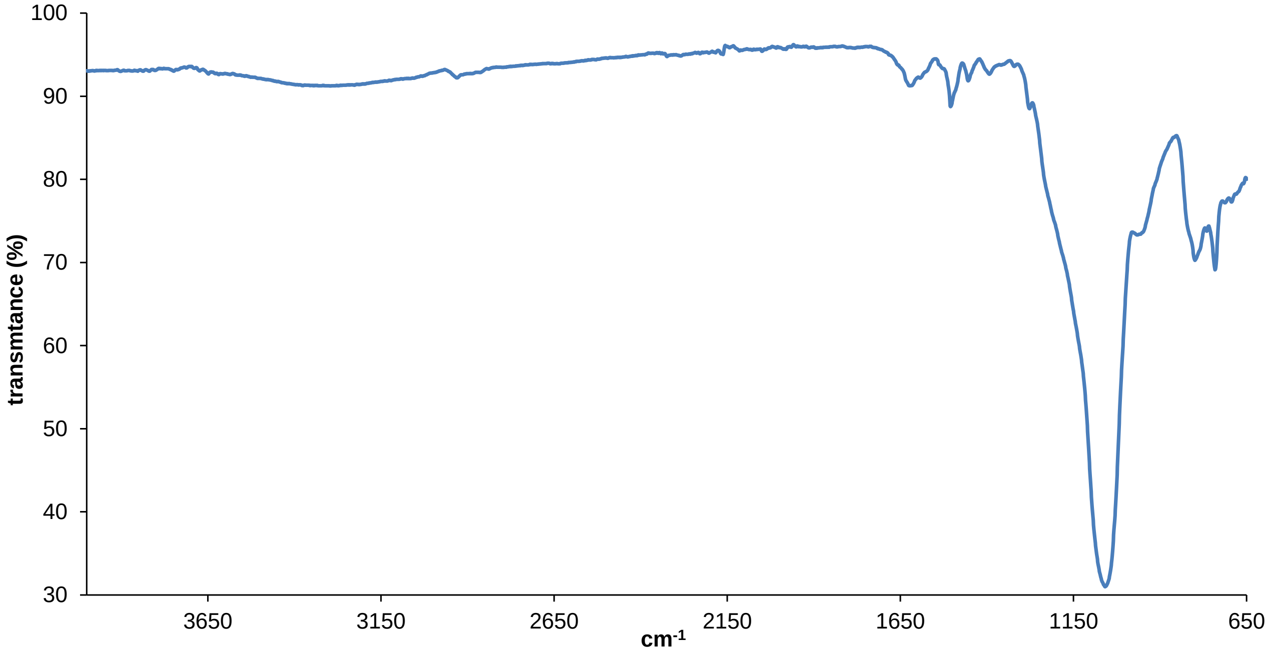

Expansion of 4000-1400 cm^{-1}

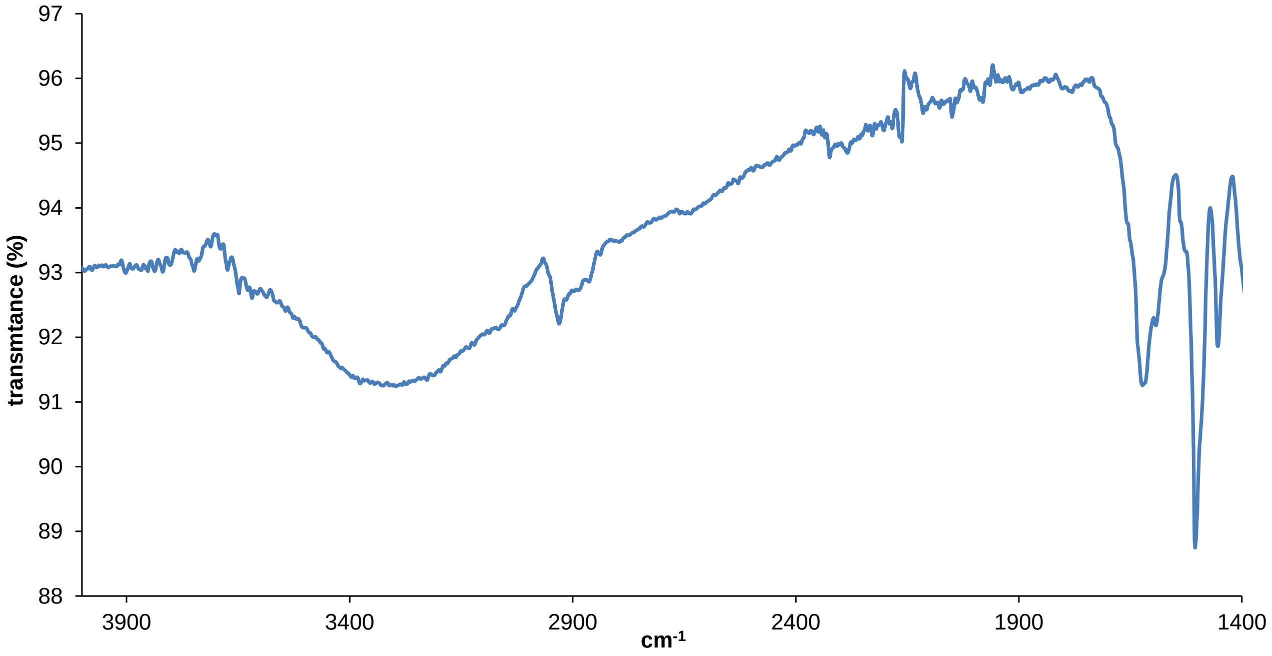

Solid State $^{13}C\{^{1}H\}$ NMR Spectrum (100 MHz)

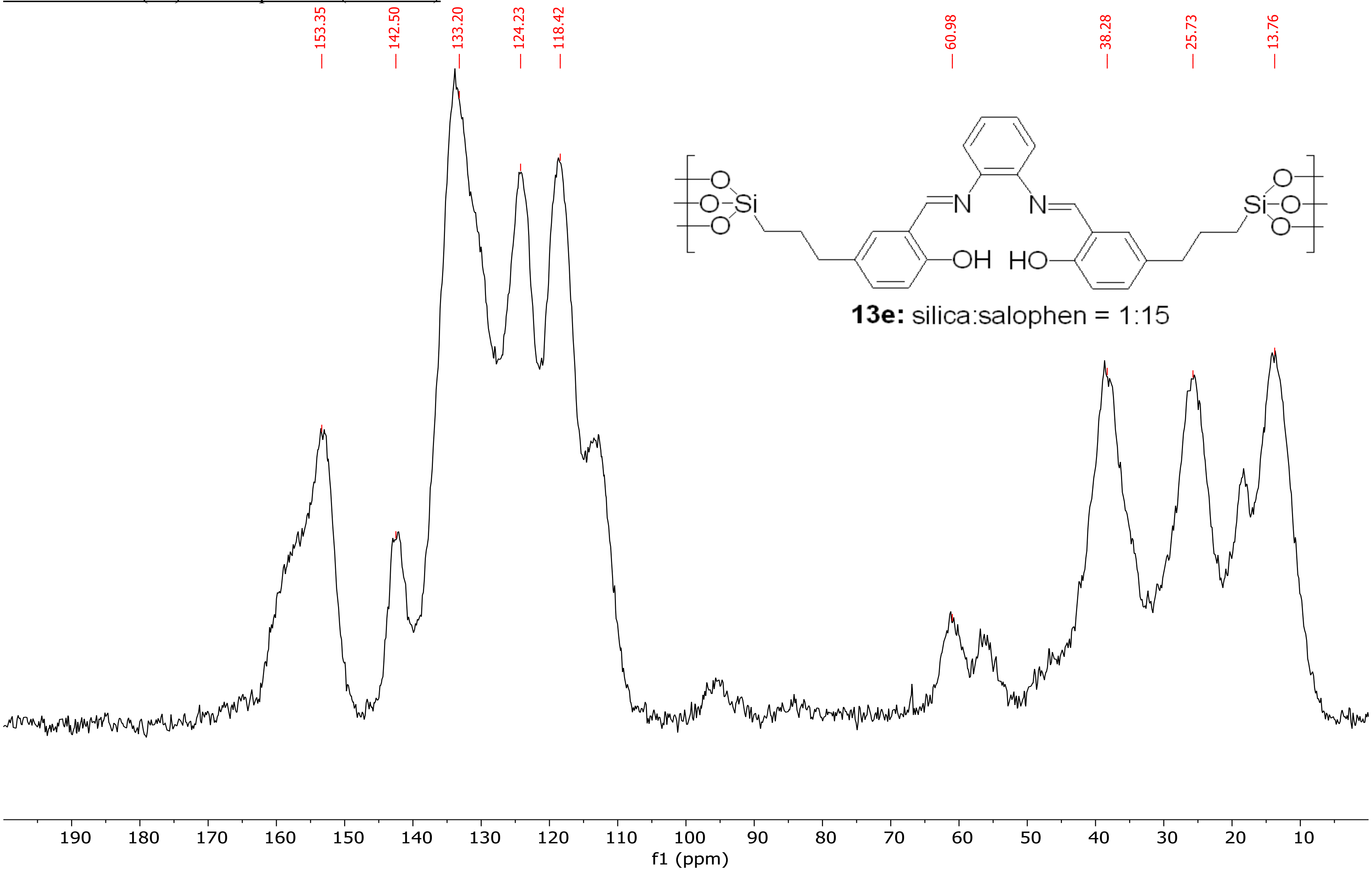

Silica-supported salophen 13f

Analysis

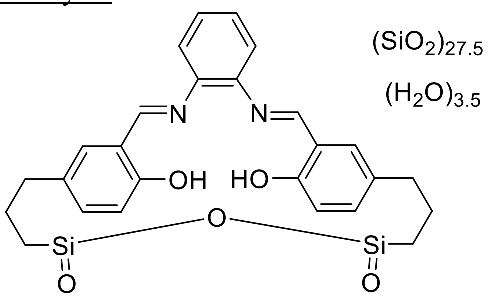

Chemical Formula: $C_{26}H_{33}N_2O_{63.5}Si_{29.5}$
Molecular Weight: 2218.01
Elemental Analysis: C, 14.08; H, 1.50; N, 1.26%

Found: C, 13.8; H, 1.9; N, 1.5%.

Mass of water: 3.5x18 = 63
So predicted %water: = 2.8%
TGA weight loss below 100 ºC: = 2.7%

% yield calculation:
Product should contain
0.35 mmol of salen unit with RMM of 503 = 503 x 0.35 mg = 176.1 mg
9.63 mmol of SiO_2 with RMM of 60 = 60 x 9.63 mg = 577.5 mg
1.23 mmol of H_2O with RMM of 18 = 18 x 1.23 mg = 22.1 mg
So 100% yield = 775.7 mg

Actual mass of product = 431 mg
So %yield = 86%

Loading calculation
2.218 g contains 1 mmol of salophen
So loading is: 1 / 2.218 = 0.45 mmol of salophen per gram

Thermogravimetric Analysis

Full trace

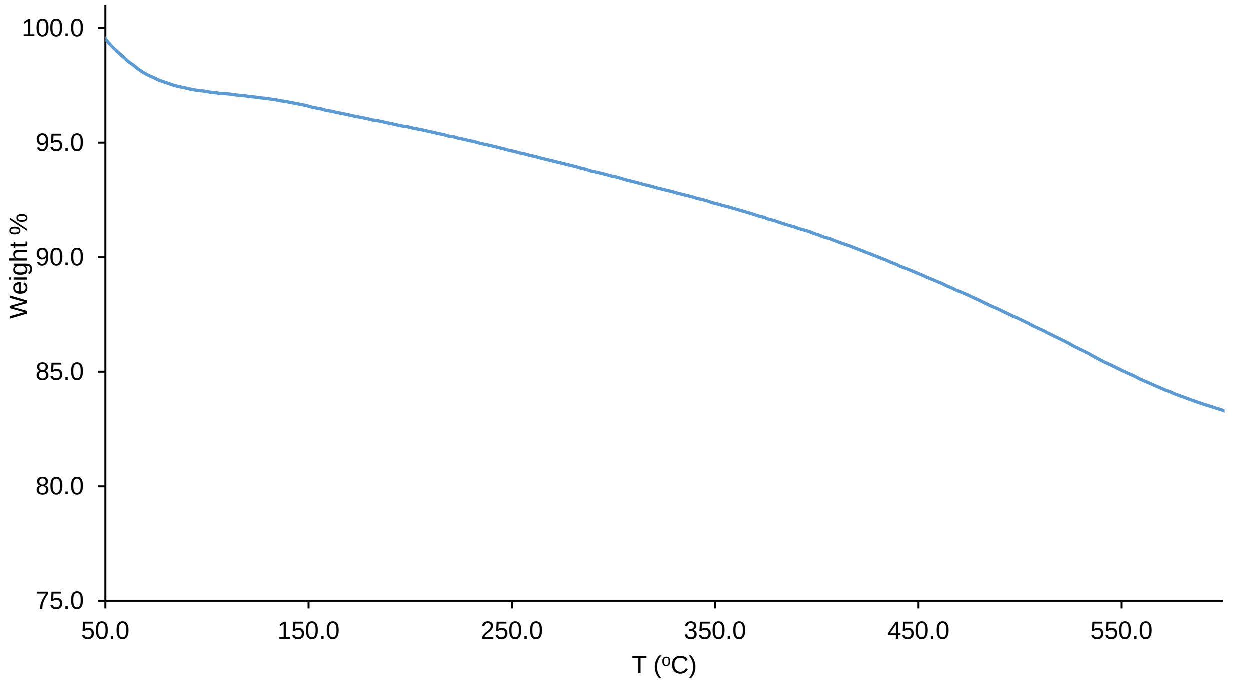

Expansion of region below 150 ºC

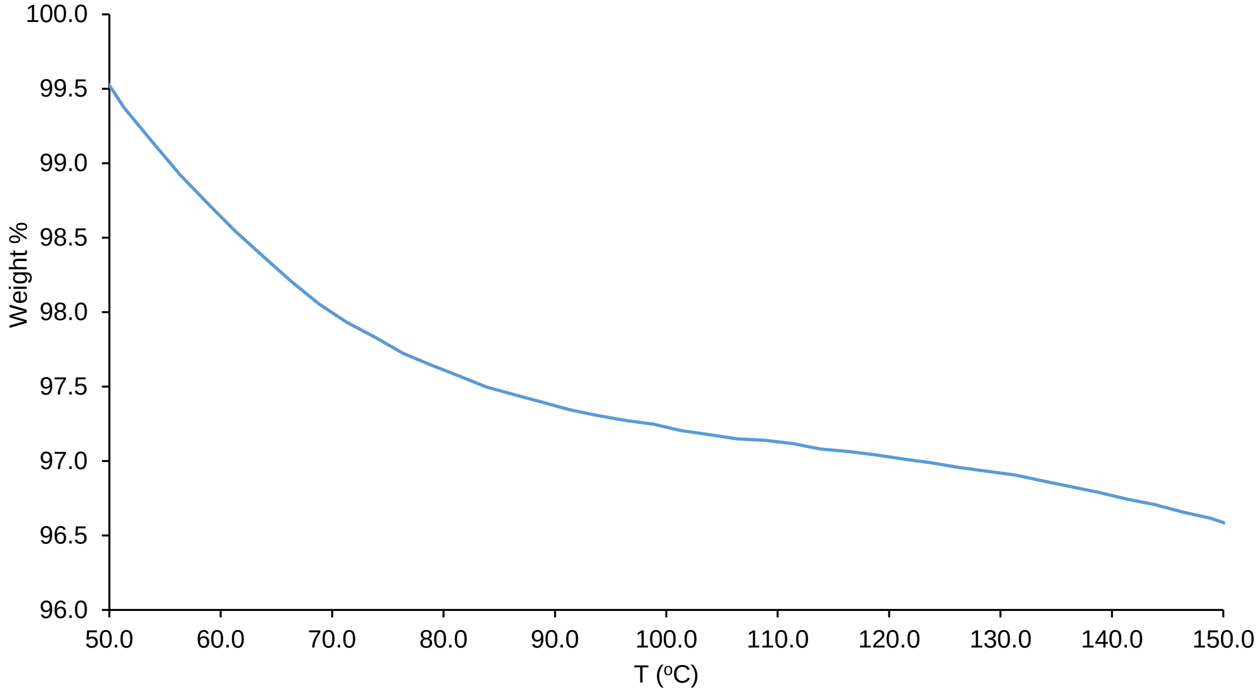

IR spectrum

Full spectrum

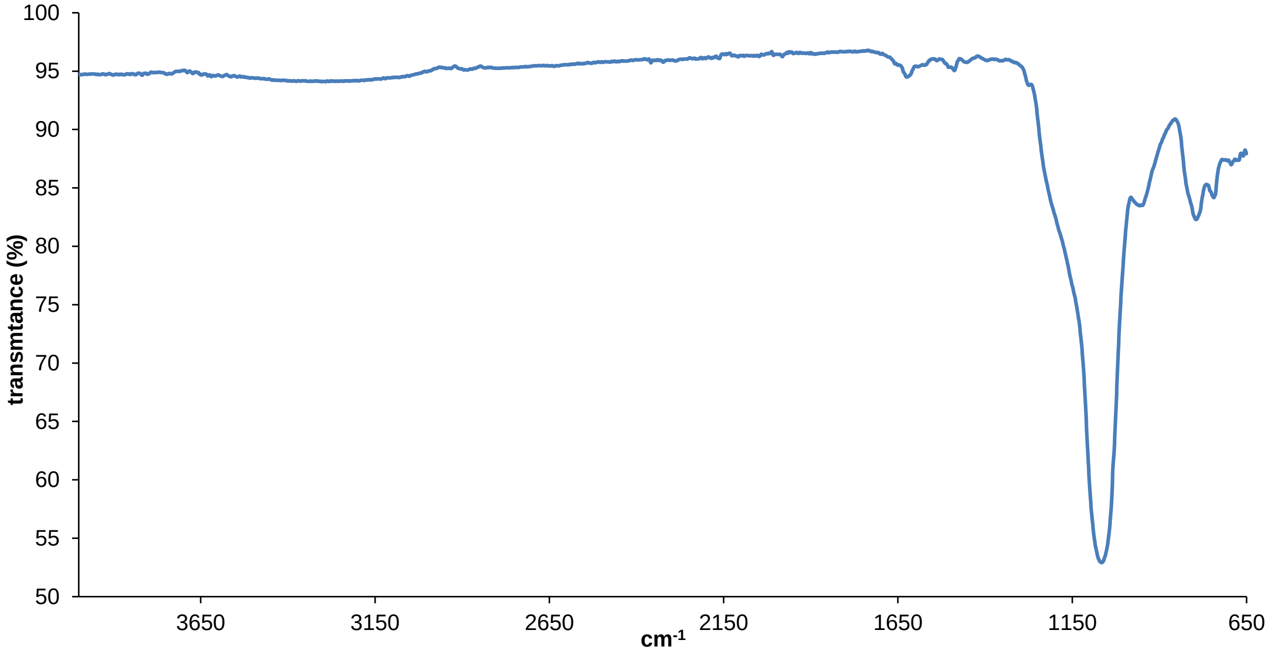

Expansion of 4000-1400 cm^{-1}

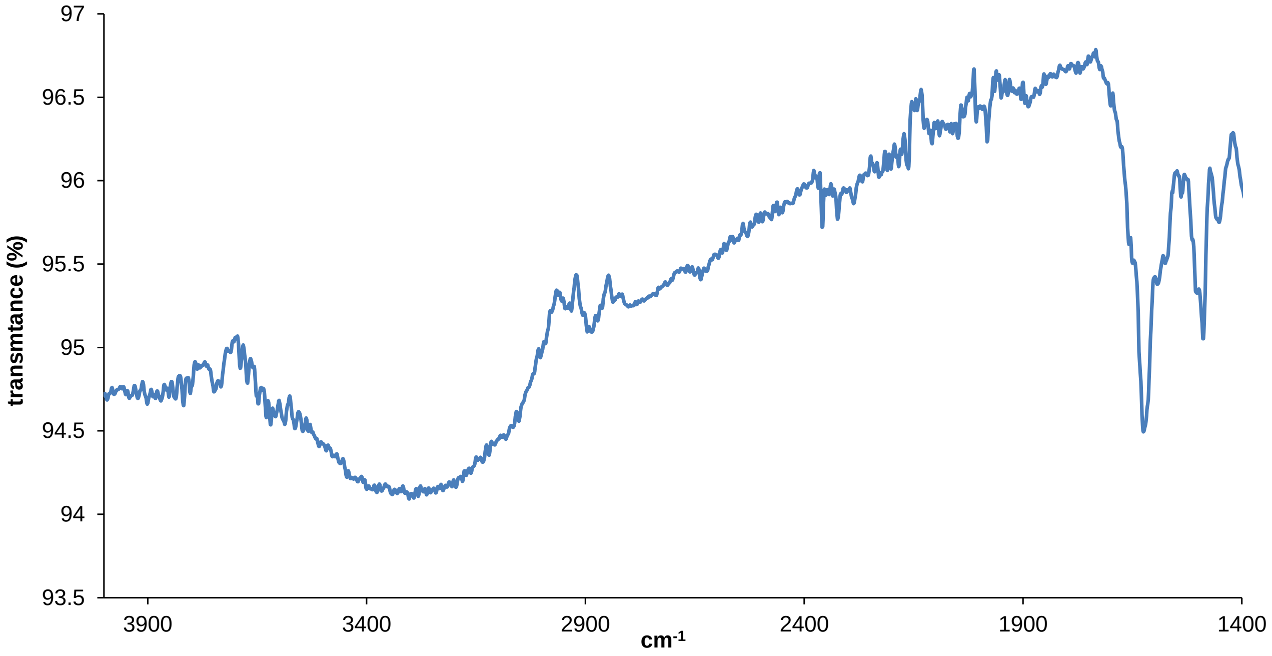

Solid State $^{13}C\{^{1}H\}$ NMR Spectrum (100 MHz)

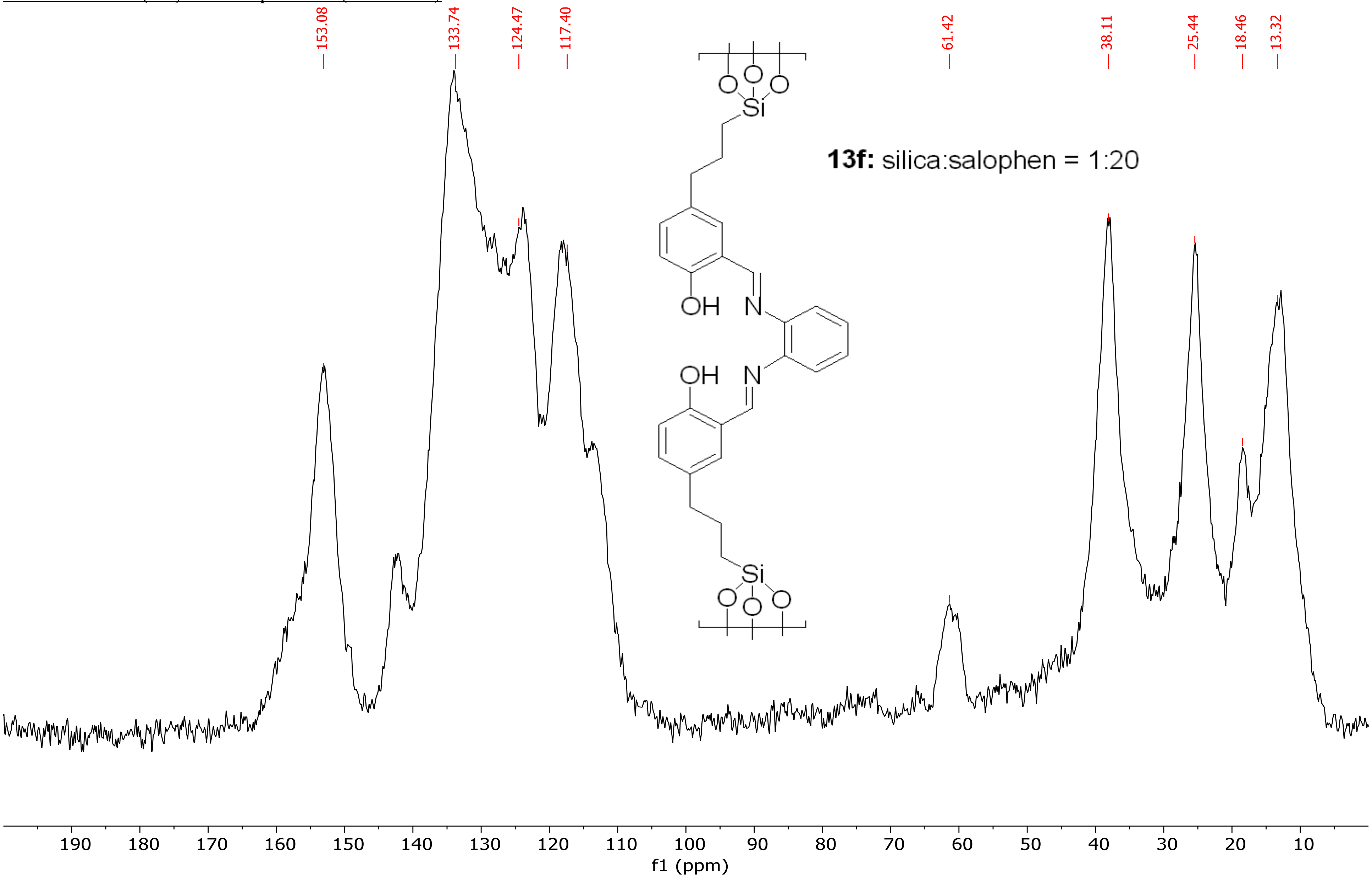

Silica-supported aldehyde 14

<u>Analysis</u>

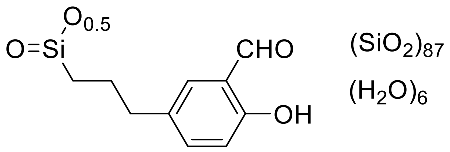

Chemical Formula: $C_{10}H_{23}O_{183.5}Si_{88}$
Exact Mass: 5540.22
Elemental Analysis: C, 2.16; H, 0.42%

Found: C, 2.2; H, 0.4%.

Mass of water: 6x18 = 108
So predicted %water: = 1.9%
TGA weight loss below 100 °C: = 2.0%

Loading calculation
5.540 g contains 1 mmol of aldehyde
So loading is: 1 / 5.54 = 0.18 mmol of aldehyde per gram

Thermogravimetric Analysis

Full trace

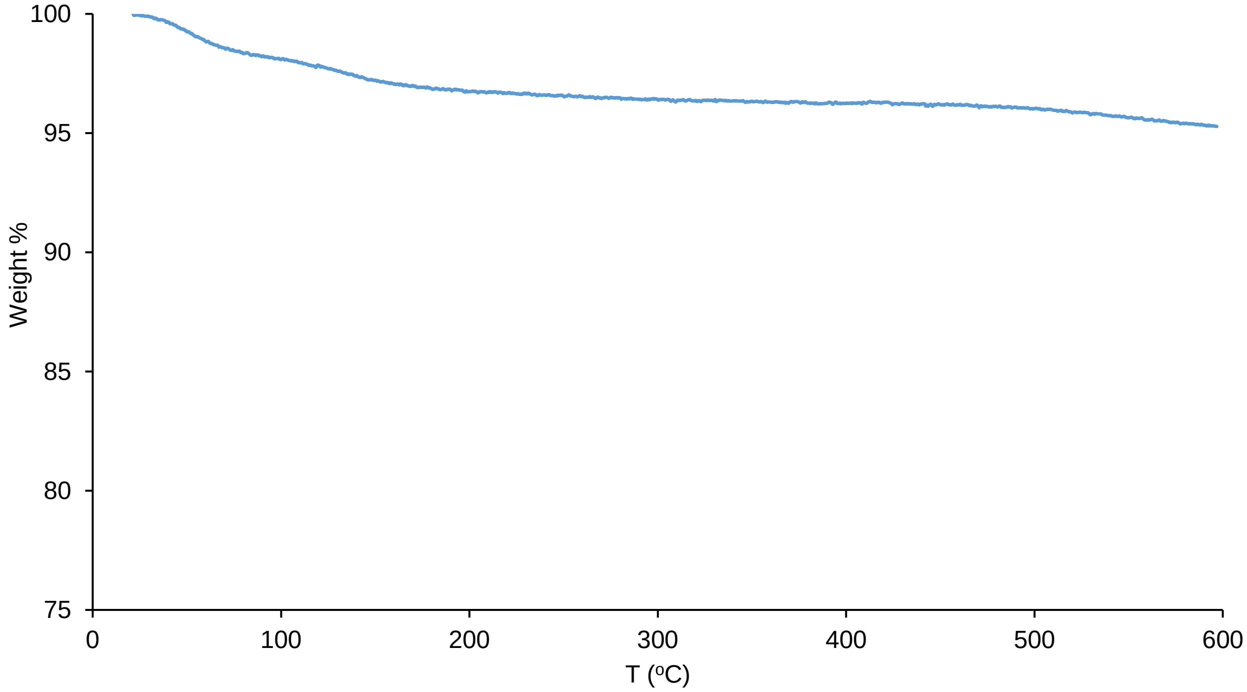

Expansion of region below 150 ºC

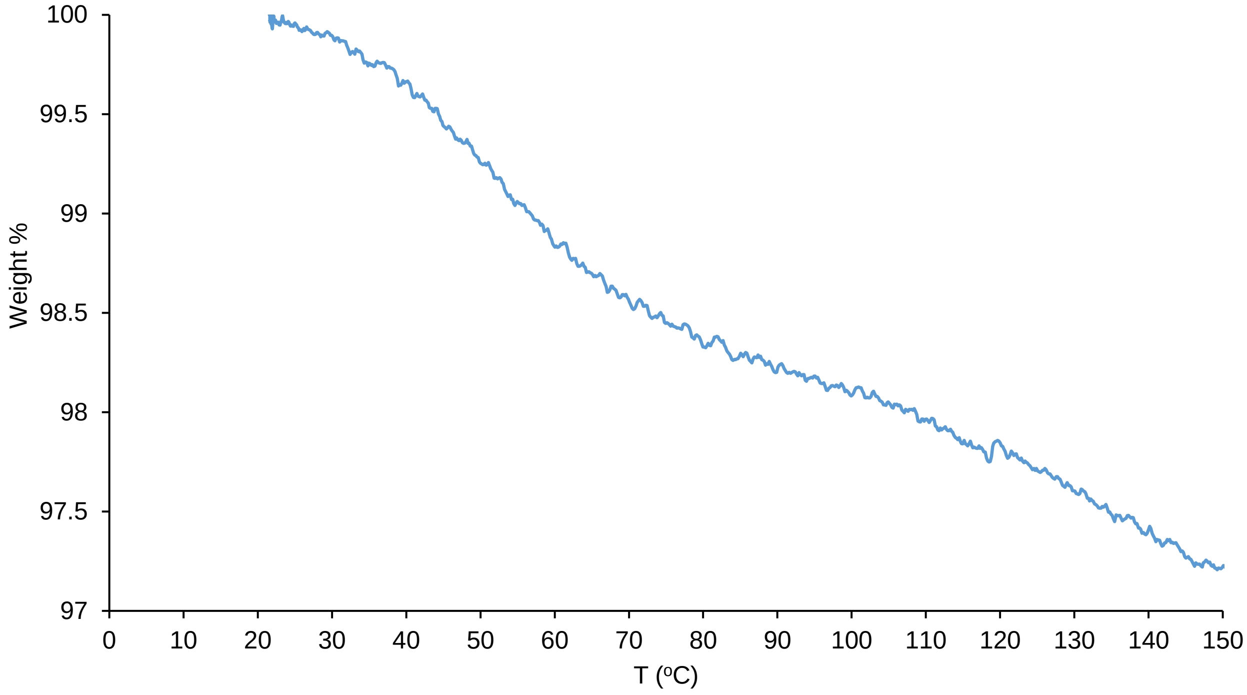

IR spectrum

Full spectrum

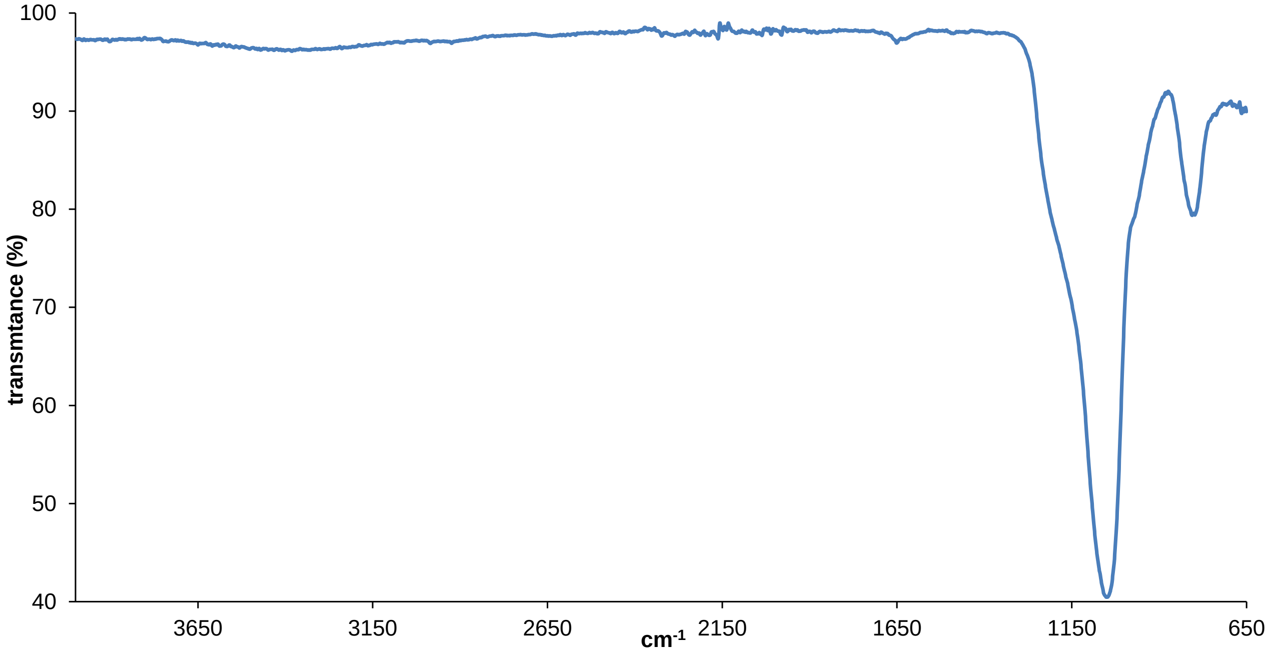

Expansion of 4000-1400 cm^{-1}

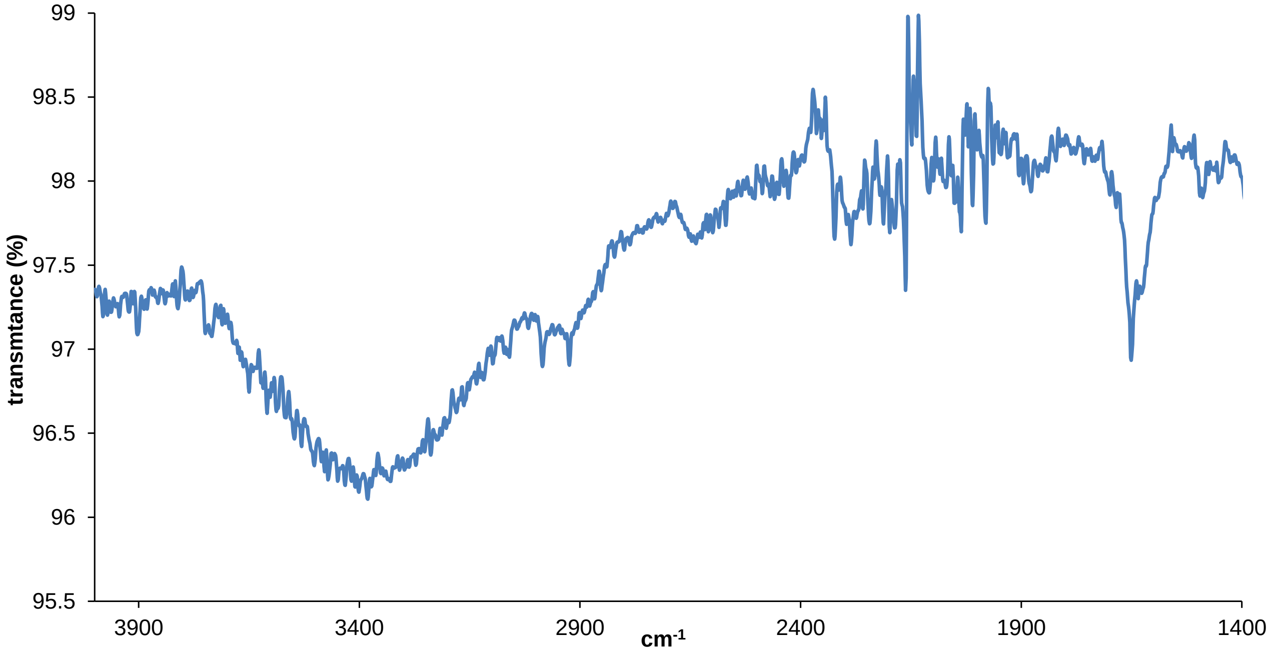

Solid State $^{13}C\{^1H\}$ NMR Spectrum (100 MHz)

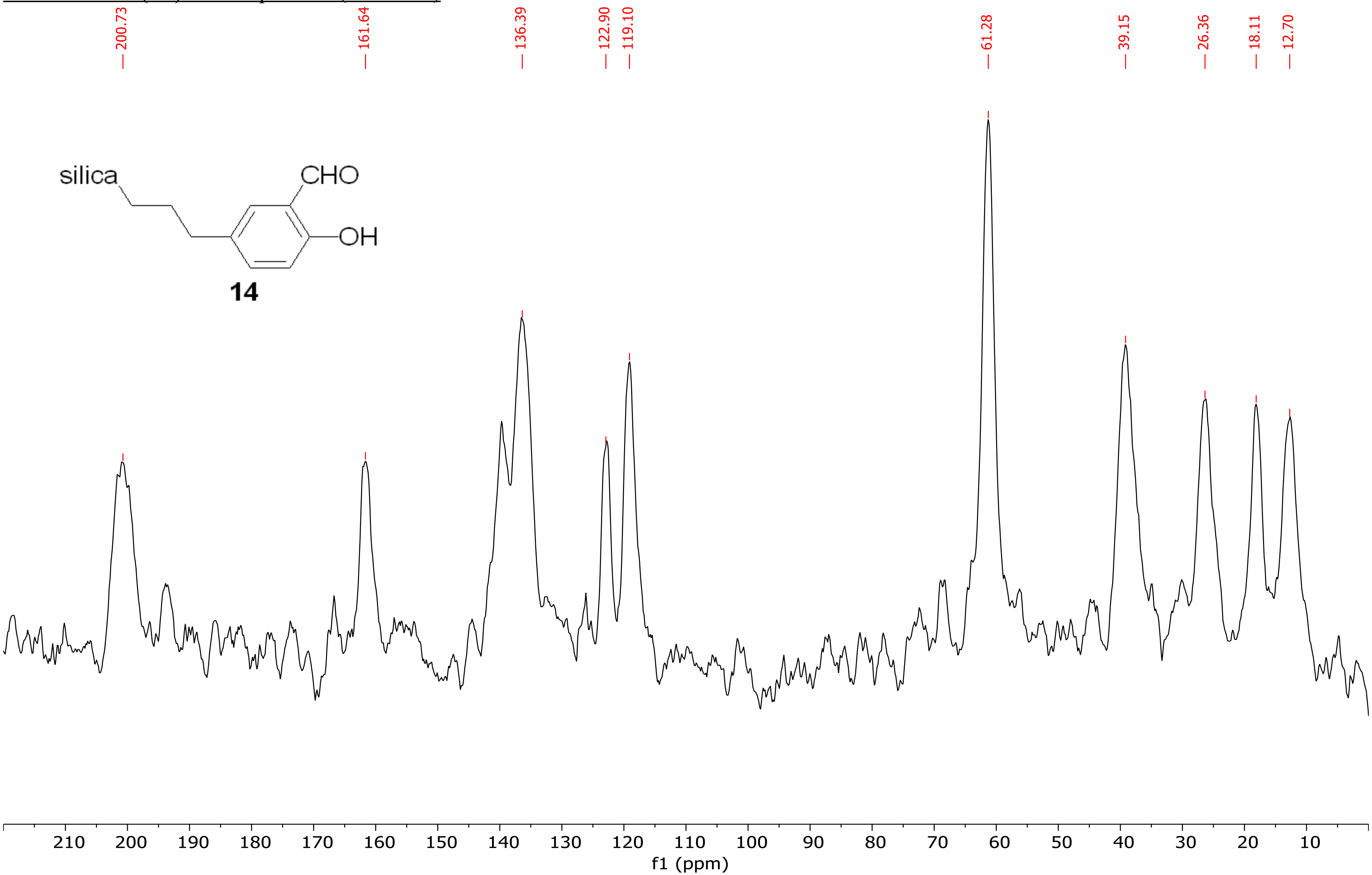

Silica-supported amine 15

Analysis

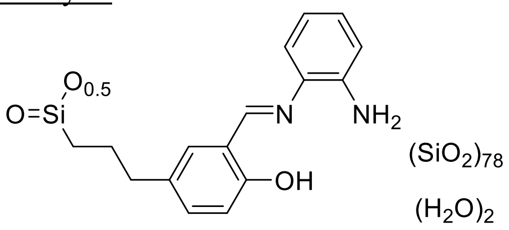

Chemical Formula: $C_{16}H_{21}N_2O_{160.5}Si_{79}$
Molecular Weight: 5027.91
Elemental Analysis: C, 3.82; H, 0.42%

Found: C, 3.8; H, 0.5%.

Mass of water: 2x18 = 36
So predicted %water: = 0.7%
TGA weight loss below 100 ºC: = 0.7%

% yield calculation:
Product should contain
0.18 mmol of amino-phenol unit with RMM of 305 = 305 x 0.18 mg = 54.9 mg
14.04 mmol of SiO_2 with RMM of 60 = 60 x 14.04 mg = 842.4 mg
0.36 mmol of H_2O with RMM of 18 = 18 x 0.36 mg = 6.5 mg
So 100% yield = 903.8 mg

Actual mass of product = 900 mg
So %yield = 99.6%

Loading calculation
5.0279 g contains 1 mmol of amine
So loading is: 1 / 5.0279 = 0.20 mmol of amine per gram

Thermogravimetric Analysis

Full trace

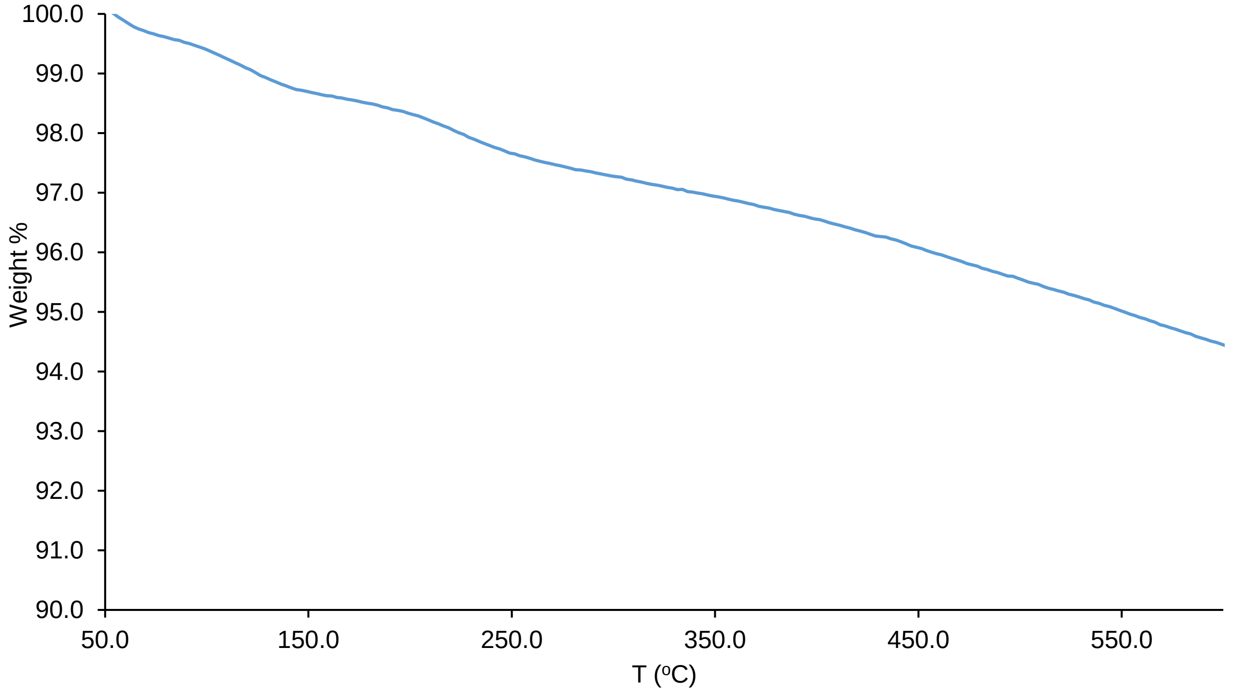

Expansion of region below 150 °C

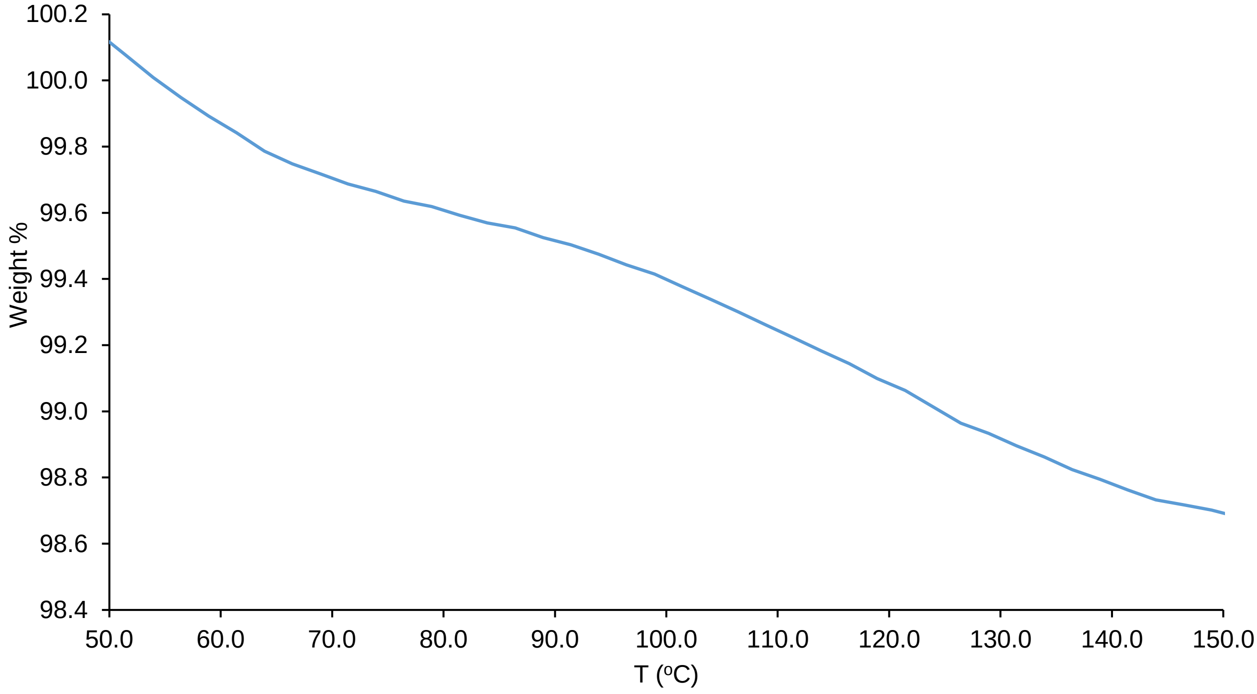

IR spectrum

Full spectrum

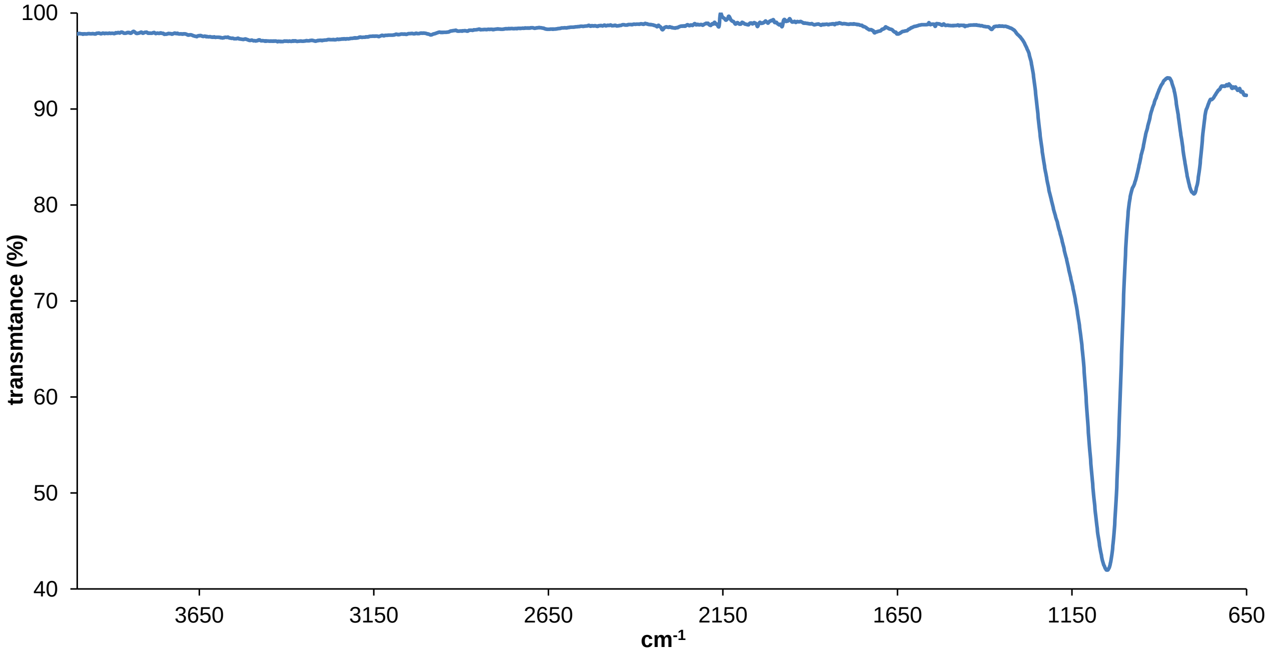

Expansion of 4000-1400 cm^{-1}

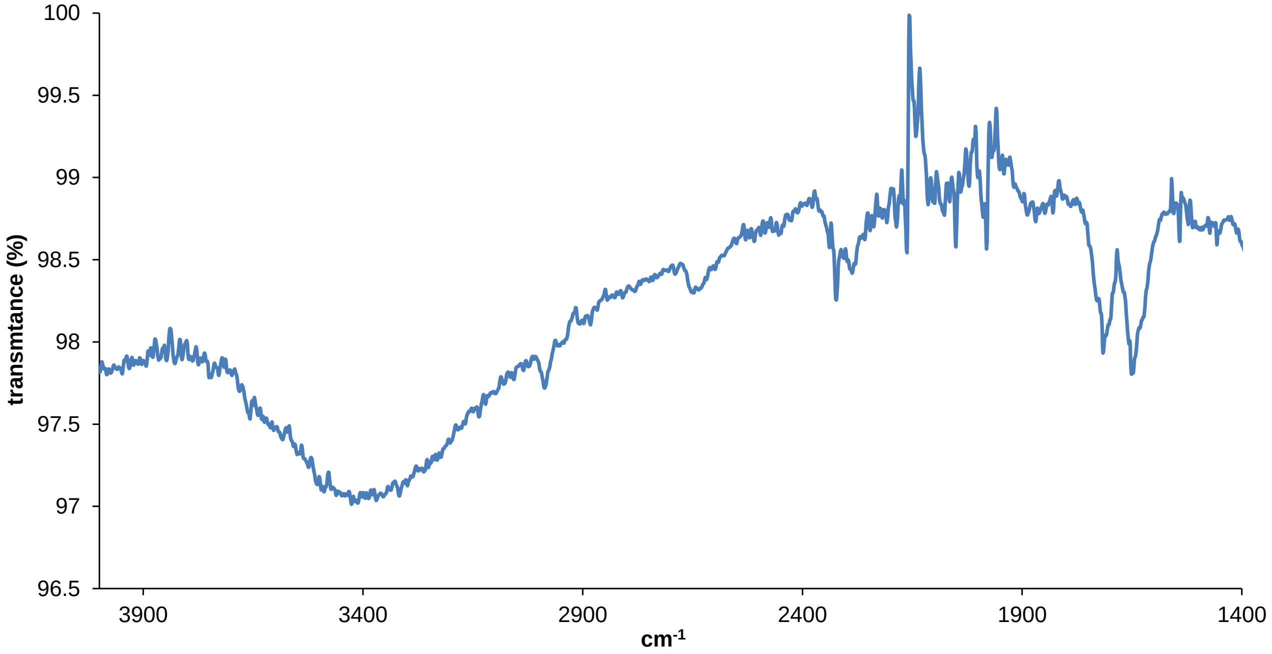

Solid State $^{13}C\{^1H\}$ NMR Spectrum (100 MHz)

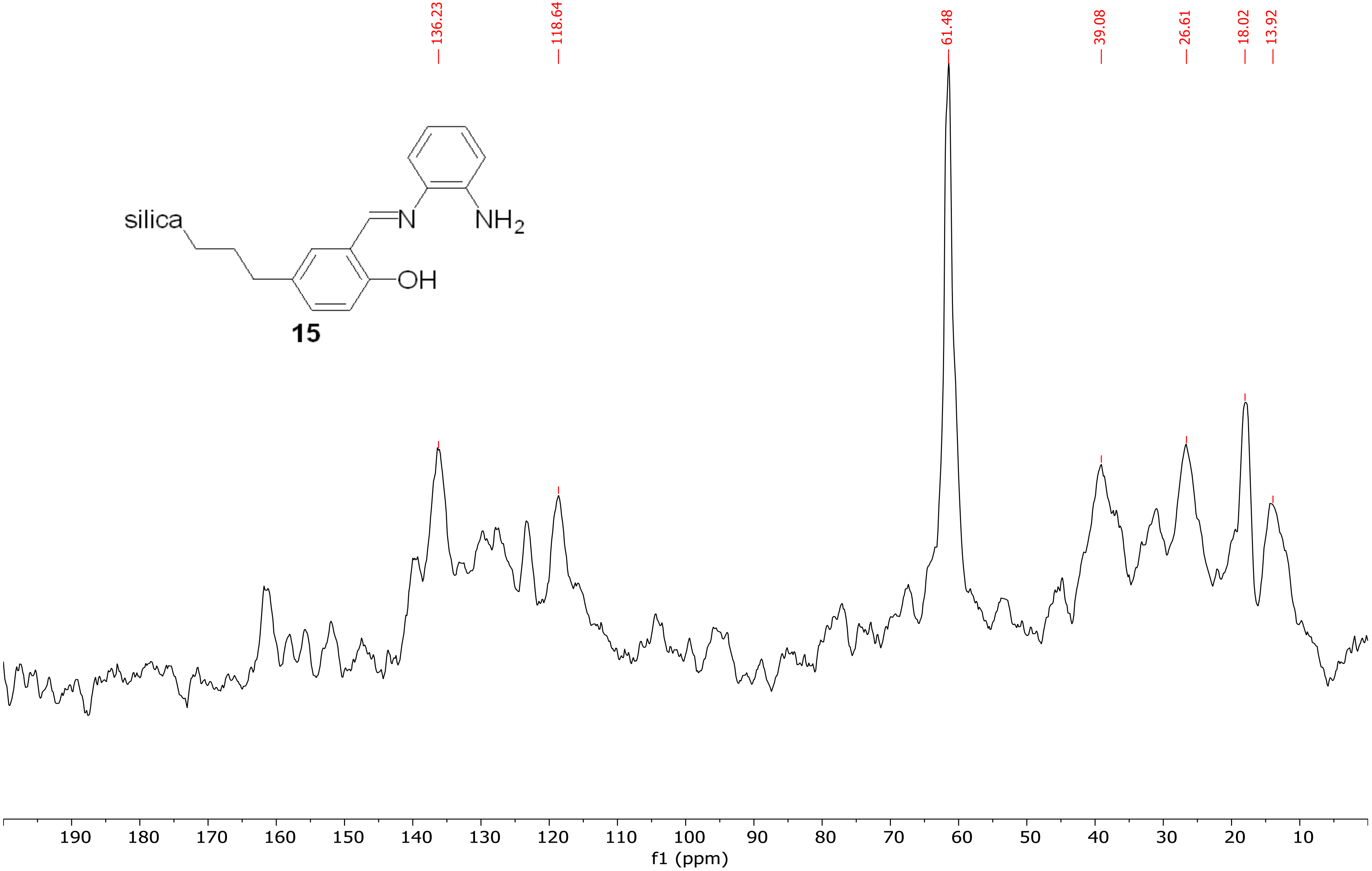

Silica-supported salophen 16

Analysis

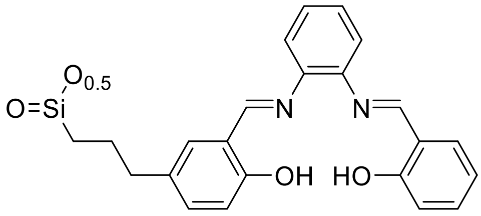

$(SiO_2)_{113}$

$(H_2O)_2$

Chemical Formula: $C_{23}H_{25}N_2O_{231.5}Si_{114}$
Molecular Weight: 7234.93
Elemental Analysis: C, 3.82; H, 0.35%

Found: C, 3.7; H, 0.5%.

Mass of water:2x18 = 36
So predicted %water: = 0.5%
TGA weight loss below 100 ºC: = 0.4%

% yield calculation:
Product should contain
0.1 mmol of salophen unit with RMM of 409.5 = 409.5 x 0.1 mg = 40.95 mg
11.3 mmol of SiO_2 with RMM of 60 = 60 x 11.3 mg = 678 mg
0.2 mmol of H_2O with RMM of 18 = 18 x 0.2 mg = 3.6 mg
So 100% yield = 723 mg

Actual mass of product = 460 mg
So %yield = 64%

Loading calculation
7.2349 g contains 1 mmol of salophen
So loading is: 1 / 7.2349 = 0.14 mmol of salophen per gram

Thermogravimetric Analysis

Full trace

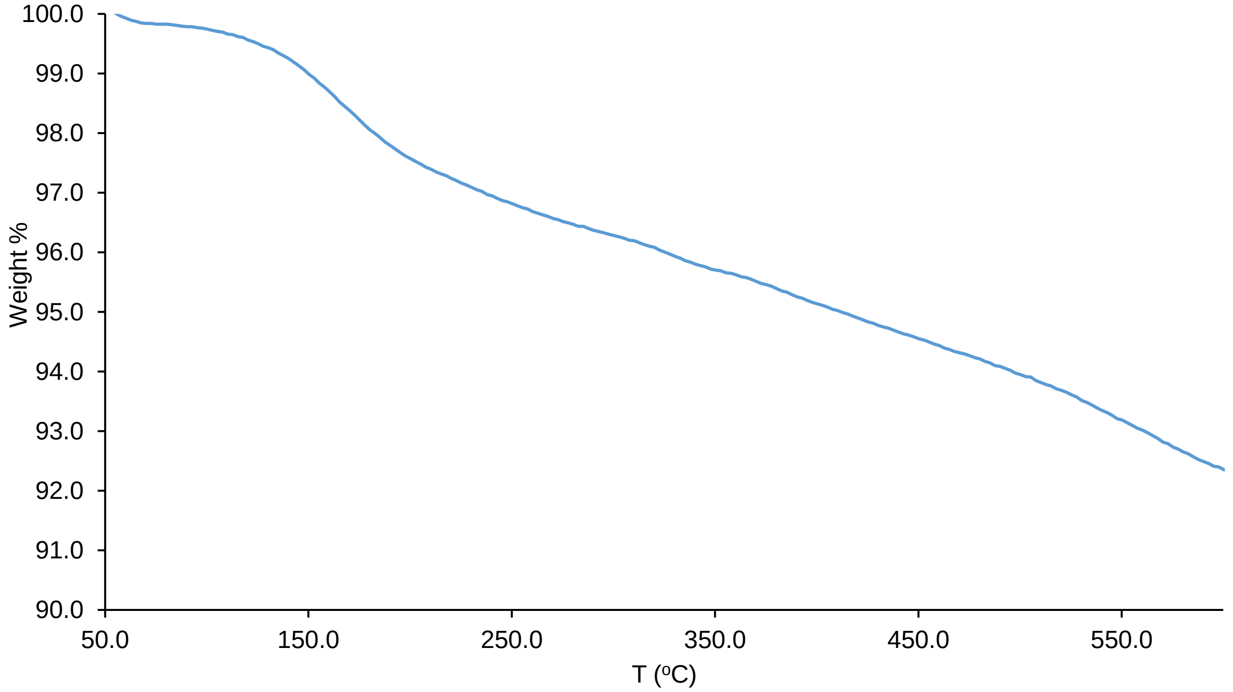

Expansion of region below 150 ºC

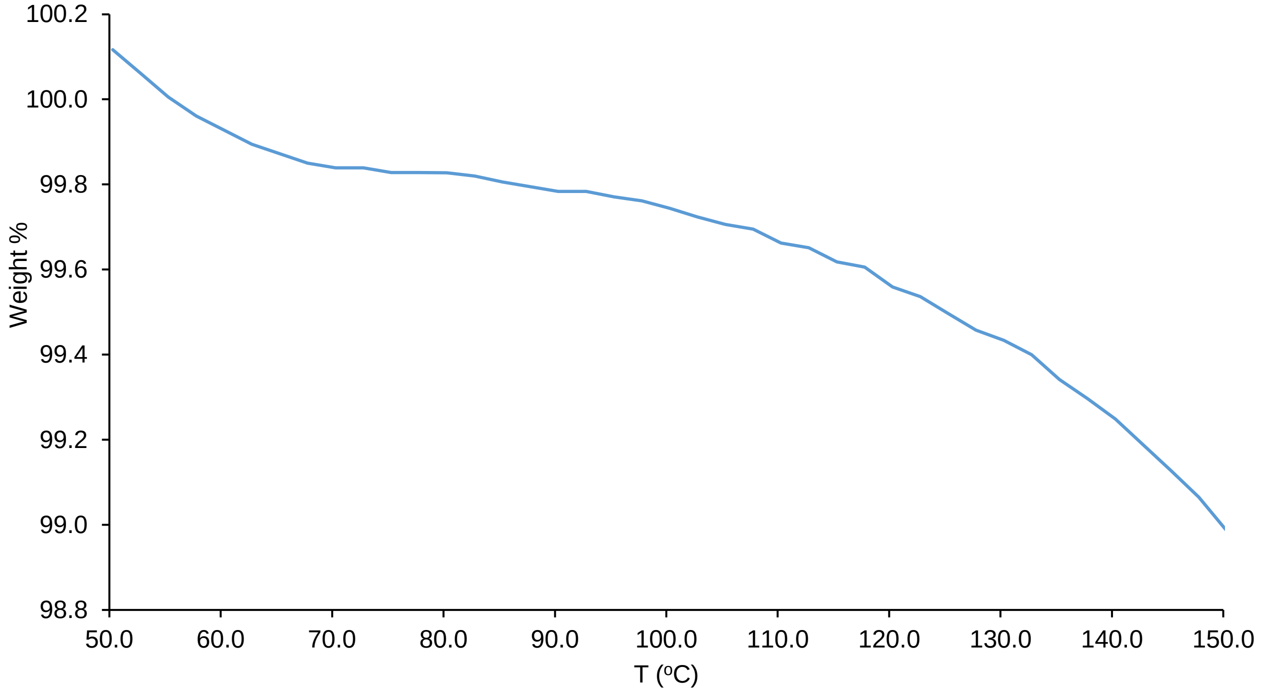

IR spectrum

Full spectrum

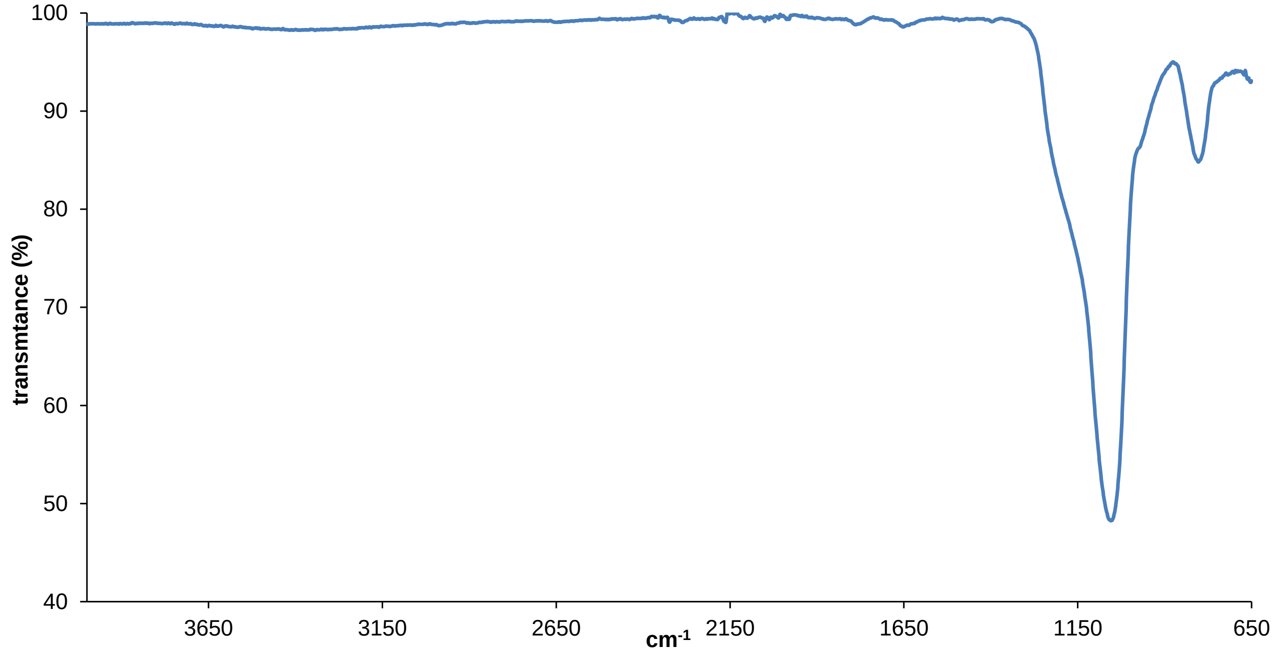

Expansion of 4000-1400 cm^{-1}

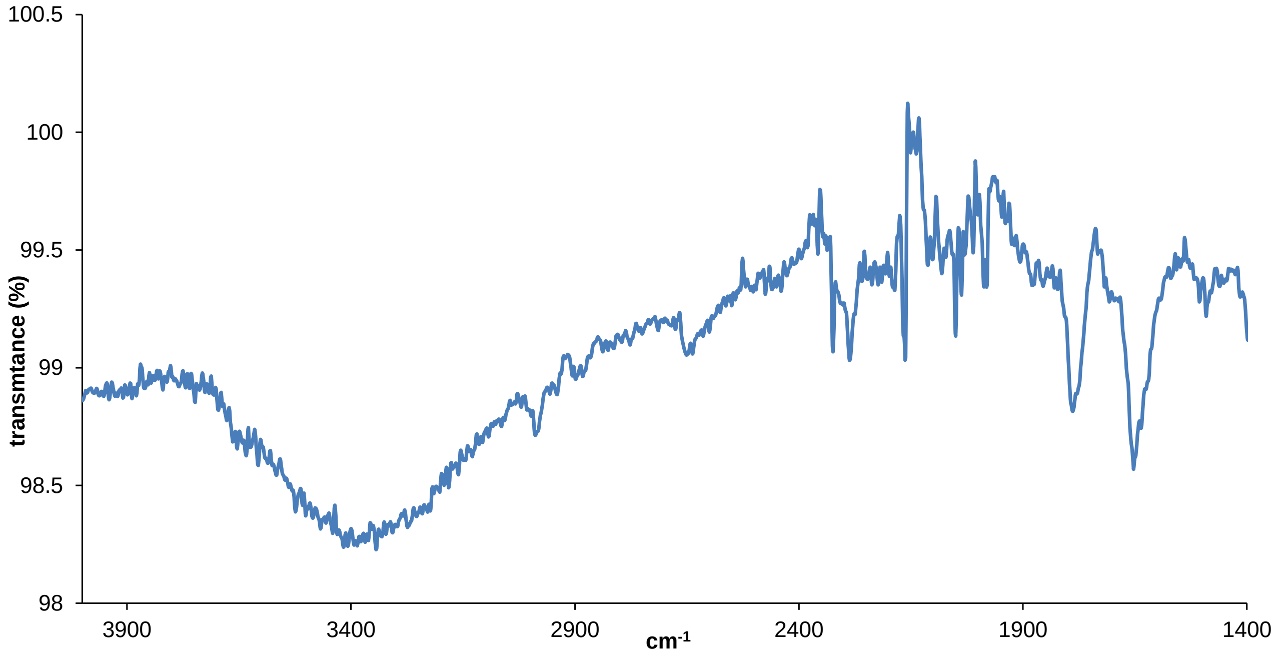

Solid State $^{13}C\{^1H\}$ NMR Spectrum (100 MHz)

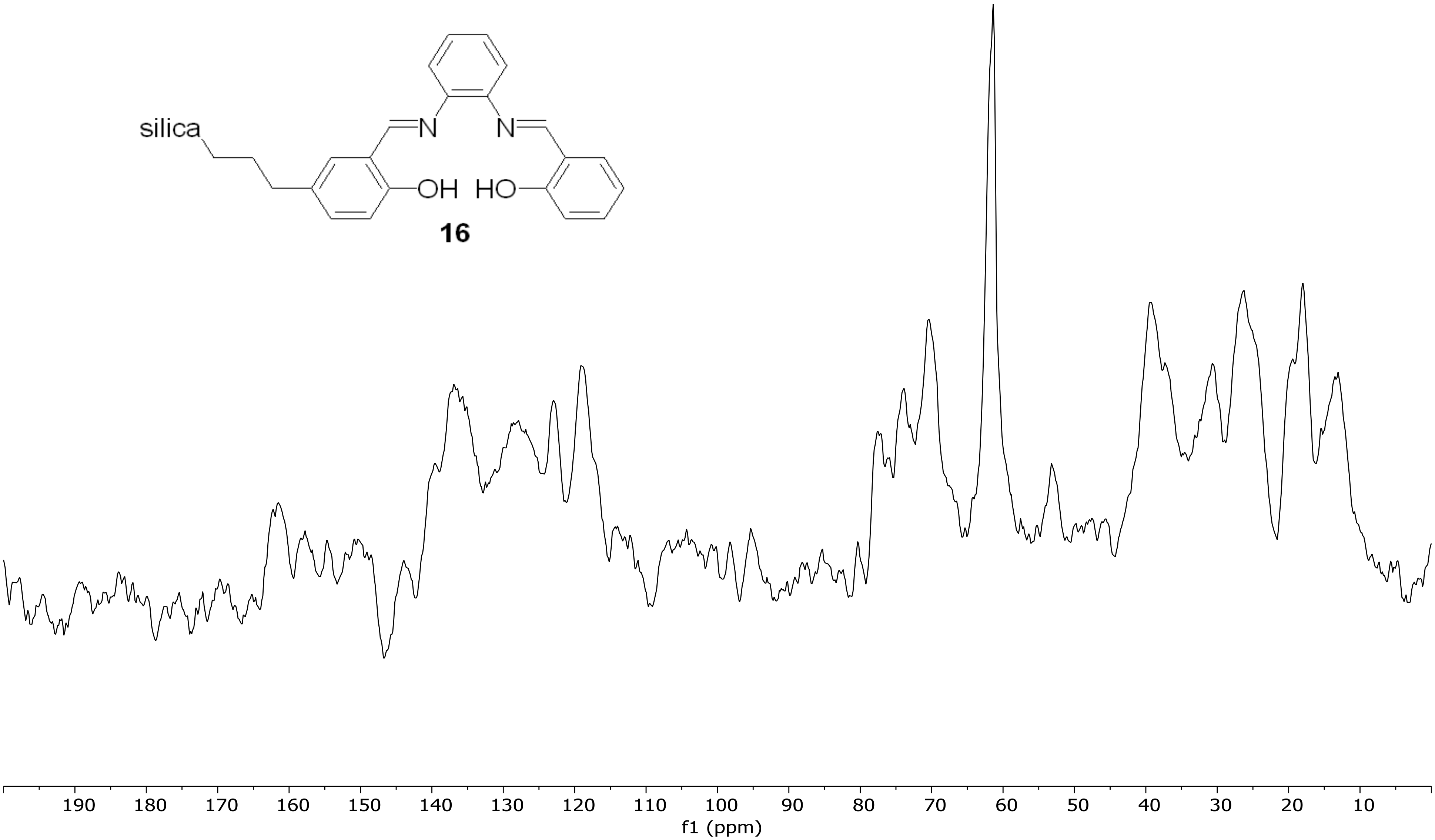

Silica-supported aldehyde 17

Analysis

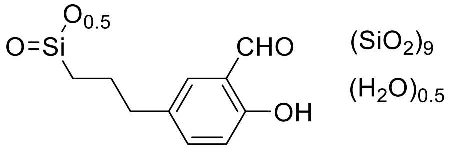

Chemical Formula: $C_{10}H_{12}O_{22}Si_{10}$
Molecular Weight: 765.03
Elemental Analysis: C, 15.70; H, 1.58%

Found: C, 15.3; H, 2.0%.

Mass of water: 0.5x18 = 9
So predicted %water: = 1.2%
TGA weight loss below 100 ºC: = 1.2%

% yield calculation:
Product should contain
1.5 mmol of aldehyde with RMM of 215 = 215 x 1.5 mg = 322.5 mg
13.5 mmol of SiO_2 with RMM of 60 = 60 x 13.5 mg = 810 mg
0.75 mmol of H_2O with RMM of 18 = 18 x 0.75 mg = 13.5 mg
So 100% yield = 1.146 g

Actual mass of product = 900 mg
So %yield = 79%

Loading calculation
0.765 g contains 1 mmol of aldehyde
So loading is: 1 / 0.765 = 1.3 mmol of aldehyde per gram

Thermogravimetric Analysis

Full trace

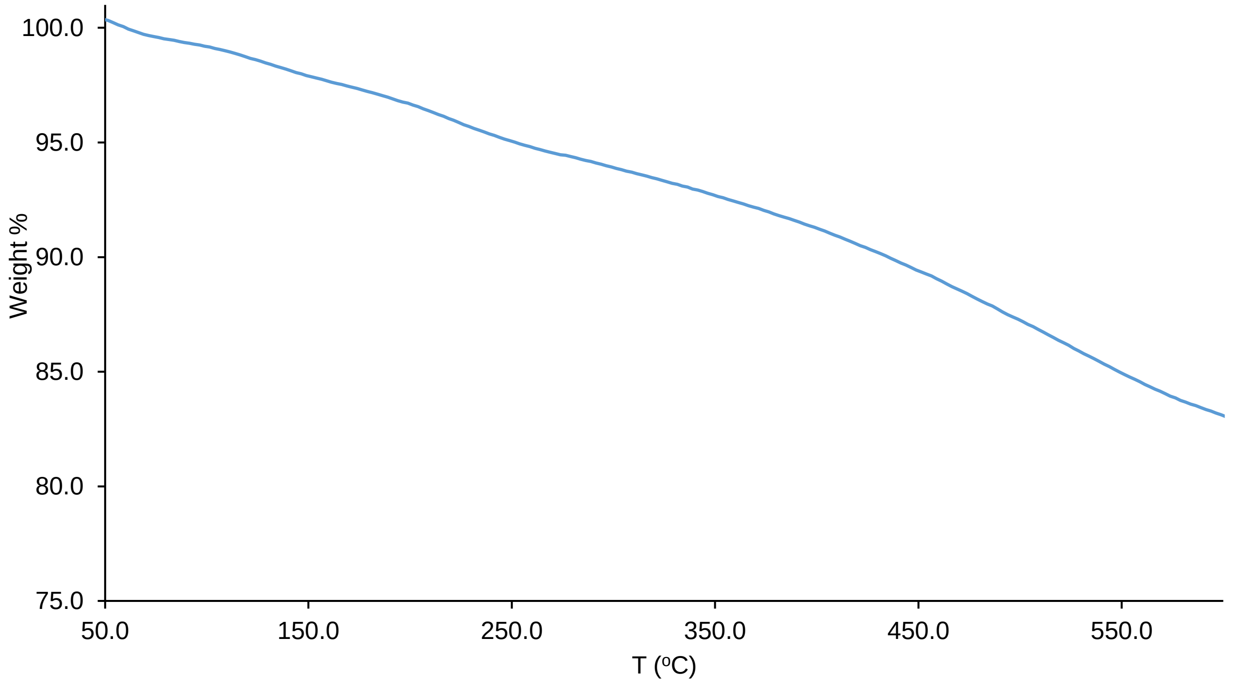

Expansion of region below 150 ºC

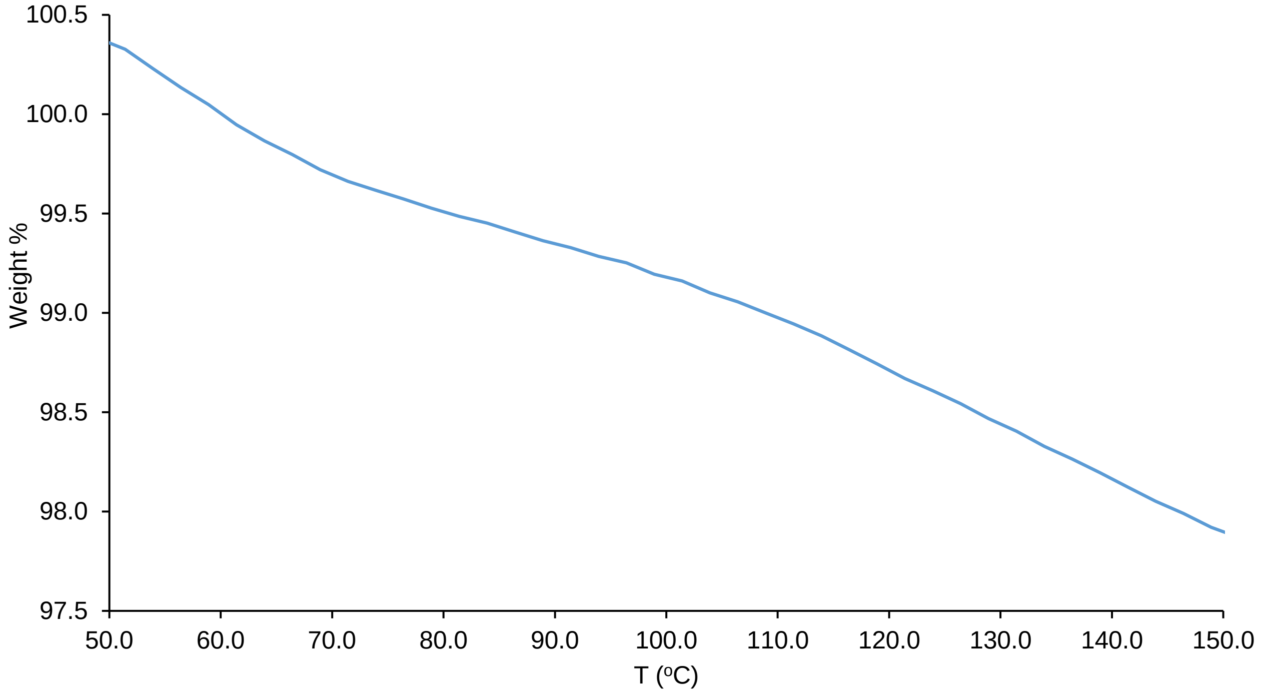

IR spectrum

Full spectrum

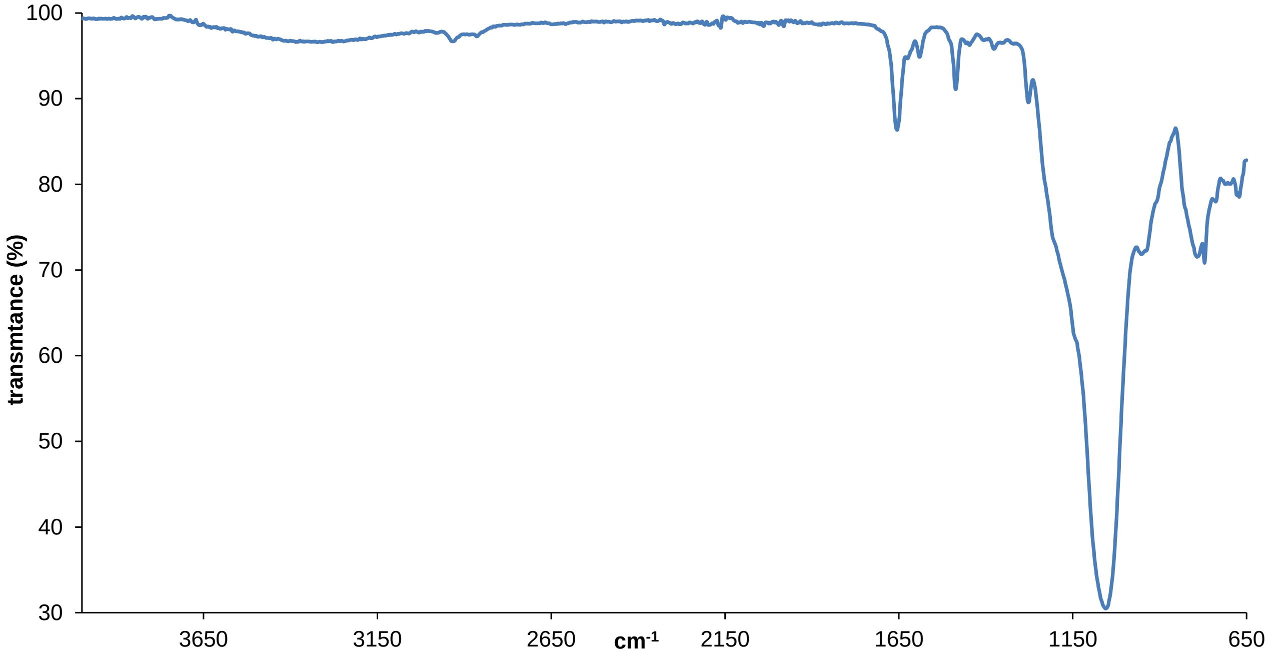

Expansion of 4000-1400 cm^{-1}

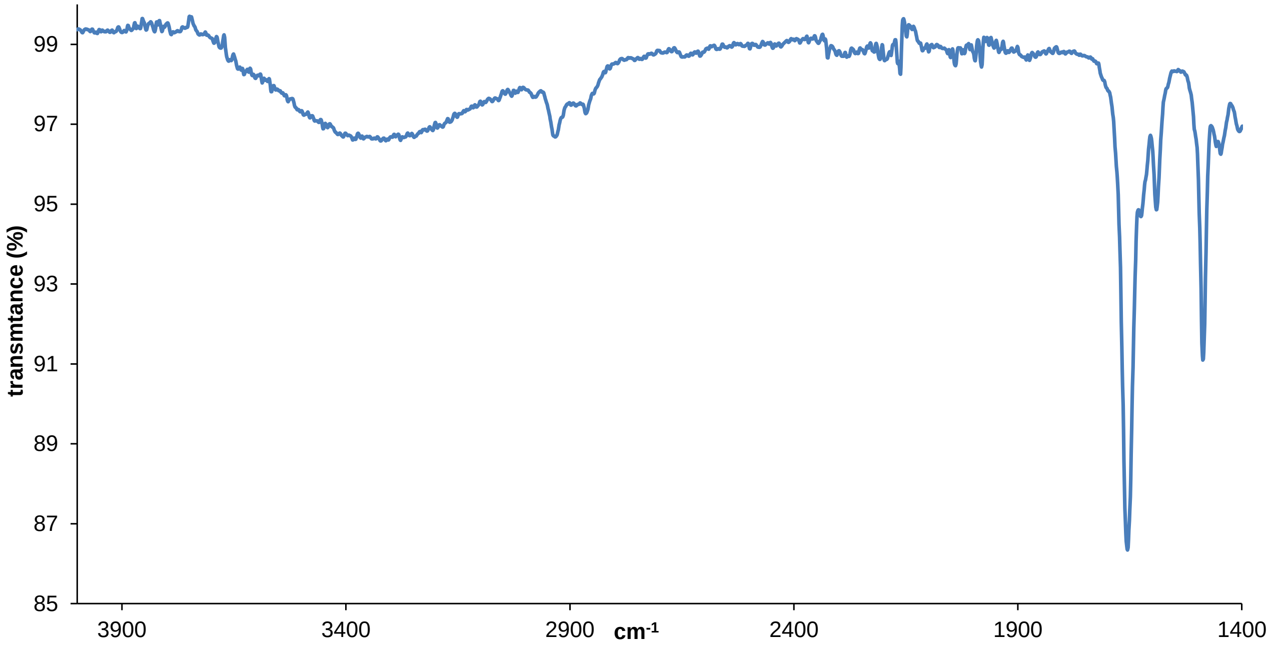

Solid State $^{13}C\{^{1}H\}$ NMR Spectrum (100 MHz)

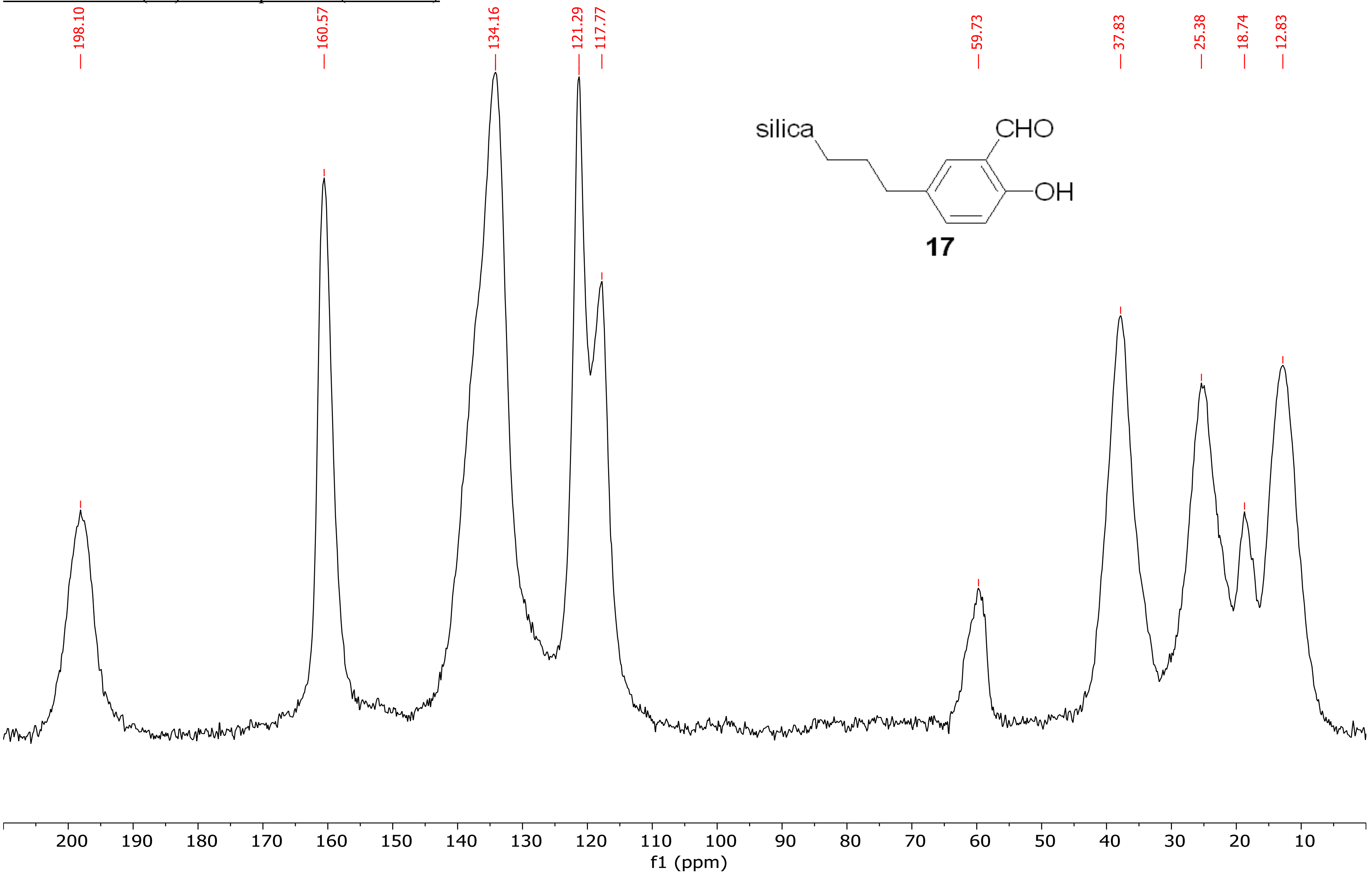

Silica-supported amine 18a

Analysis

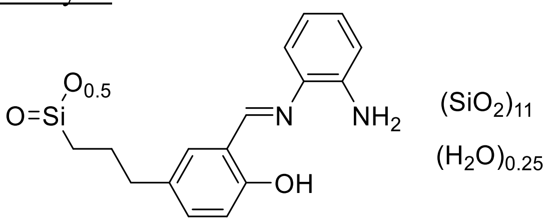

Chemical Formula: $C_{16}H_{17.5}N_2O_{24.75}Si_{12}$
Molecular Weight: 970.83
Elemental Analysis: C, 19.80; H, 1.82%

Found: C, 19.5; H, 1.8%.

Mass of water: 0.5x18 = 9
So predicted %water: = 0.5%
TGA weight loss below 100 ºC: = 0.4%

% yield calculation:
Product should contain
0.65 mmol of amine with RMM of 305 = 305 x 0.65 mg = 198.25 mg
7.2 mmol of SiO_2 with RMM of 60 = 60 x 7.2 mg = 429 mg
0.16 mmol of H_2O with RMM of 18 = 18 x 0.16 mg = 2.88 mg
So 100% yield = 630 mg

Actual mass of product = 500 mg
So %yield = 79%

Loading calculation
0.971 g contains 1 mmol of amine
So loading is: 1 / 0.971 = 1.0 mmol of amine per gram

Thermogravimetric Analysis

Full trace

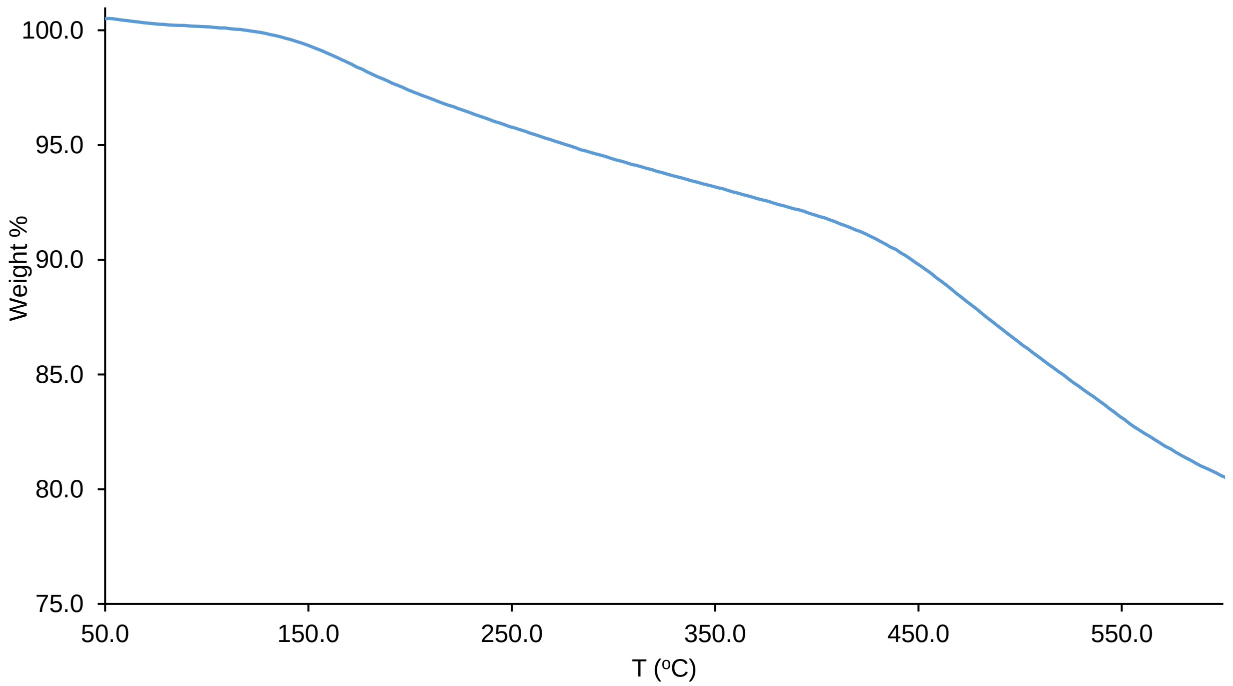

Expansion of region below 150 ºC

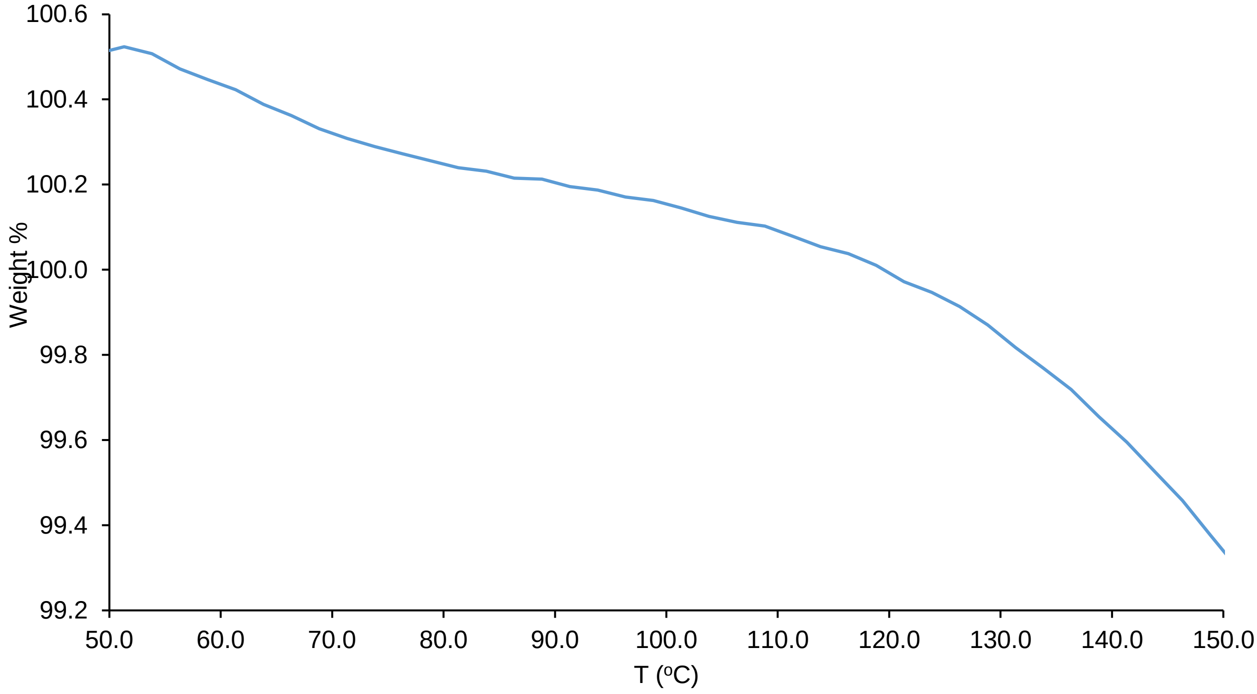

IR spectrum

Full spectrum

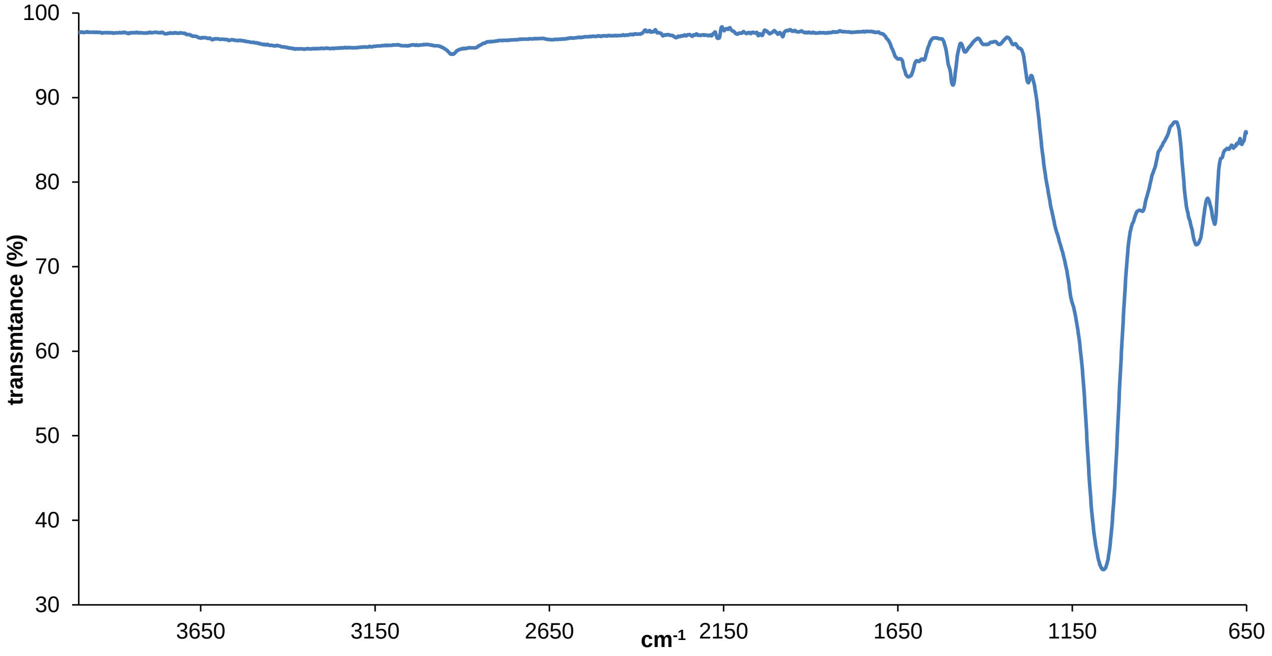

Expansion of 4000-1400 cm^{-1}

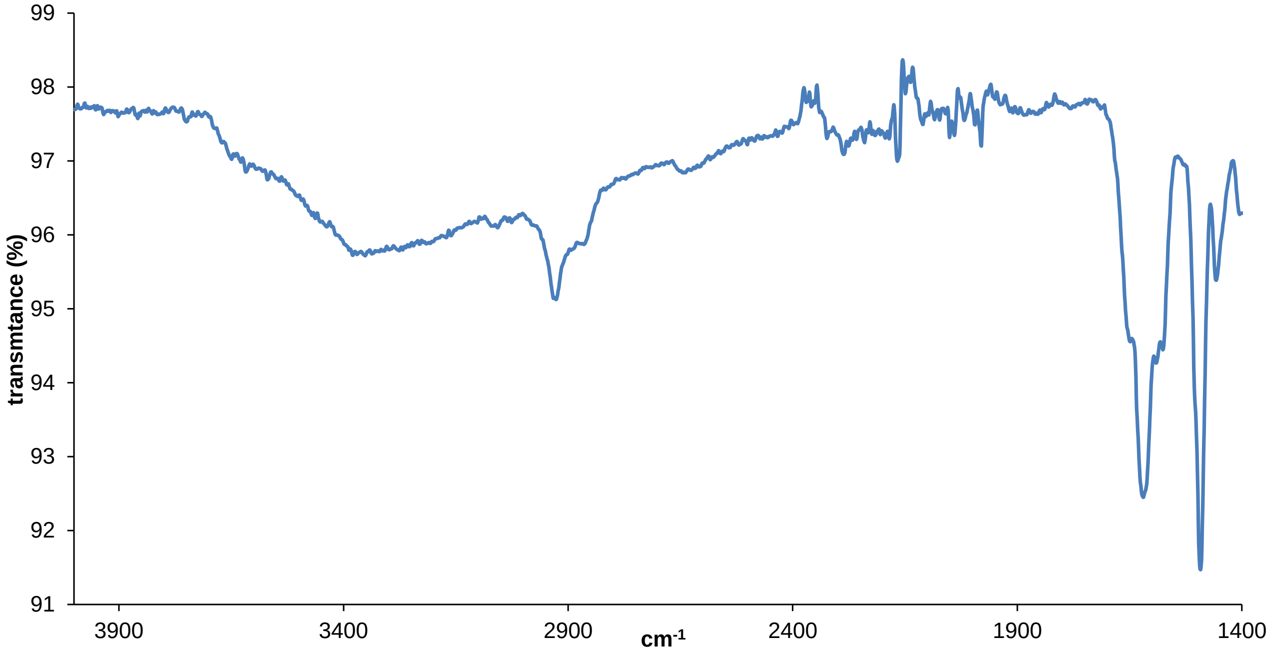

Solid State $^{13}C\{^{1}H\}$ NMR Spectrum (100 MHz)

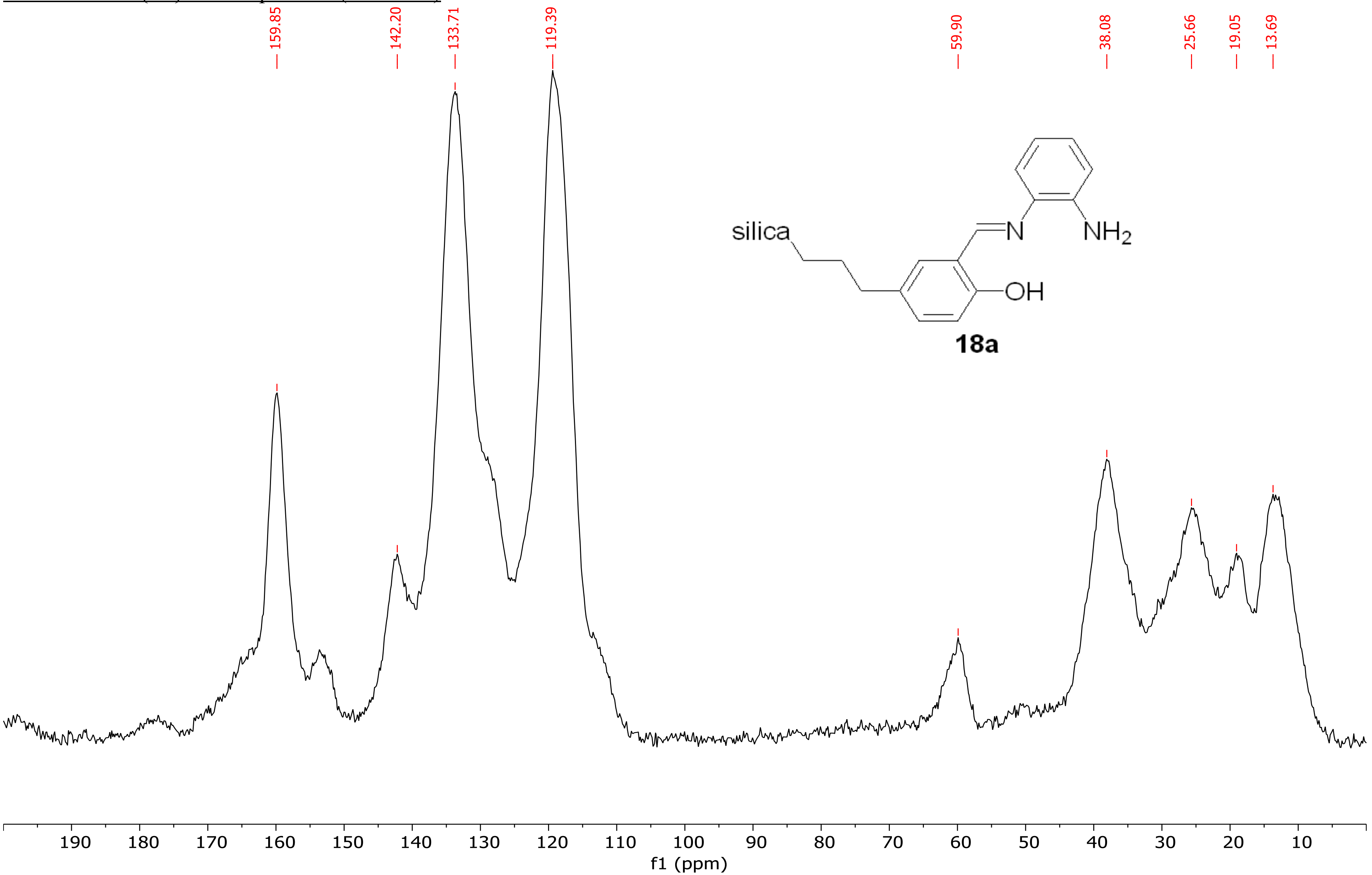

Silica-supported amine 18b

Analysis

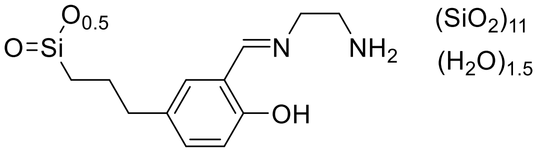

Chemical Formula: $C_{12}H_{20}N_2O_{26}Si_{12}$
Molecular Weight: 945.30
Elemental Analysis: C, 15.25; H, 2.13; N, 2.96;

Found: C, 15.1; H, 2.2; N, 2.8%.

Mass of water: 1.5x18 = 27
So predicted %water: = 2.9%
TGA weight loss below 100 °C: = 3.0%

% yield calculation:
Product should contain
0.52 mmol of amine with RMM of 257 = 257 x 0.52 mg = 133.64 mg
5.72 mmol of SiO_2 with RMM of 60 = 60 x 5.72 mg = 343.2 mg
0.78 mmol of H_2O with RMM of 18 = 18 x 0.78 mg = 14.04 mg
So 100% yield = 491 mg

Actual mass of product = 400 mg
So %yield = 81%

Loading calculation
0.945 g contains 1 mmol of amine
So loading is: 1 / 0.945 = 1.1 mmol of amine per gram

Thermogravimetric Analysis

Full trace

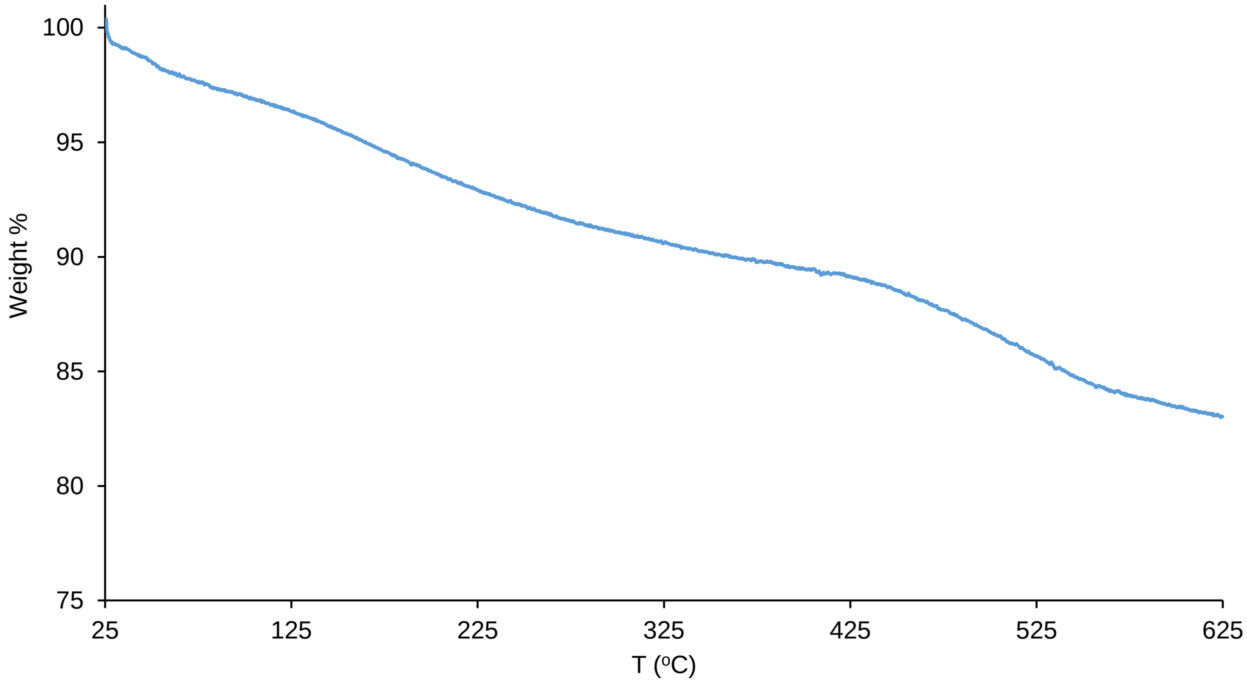

Expansion of region below 150 ºC

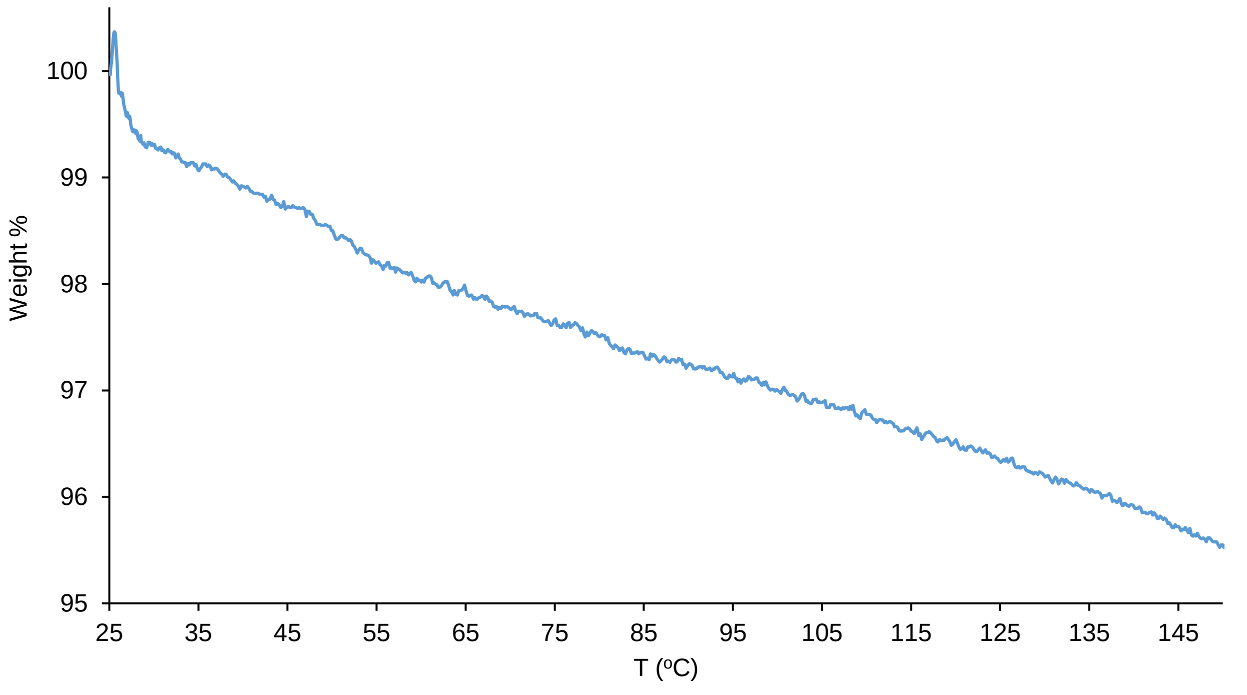

IR spectrum

Full spectrum

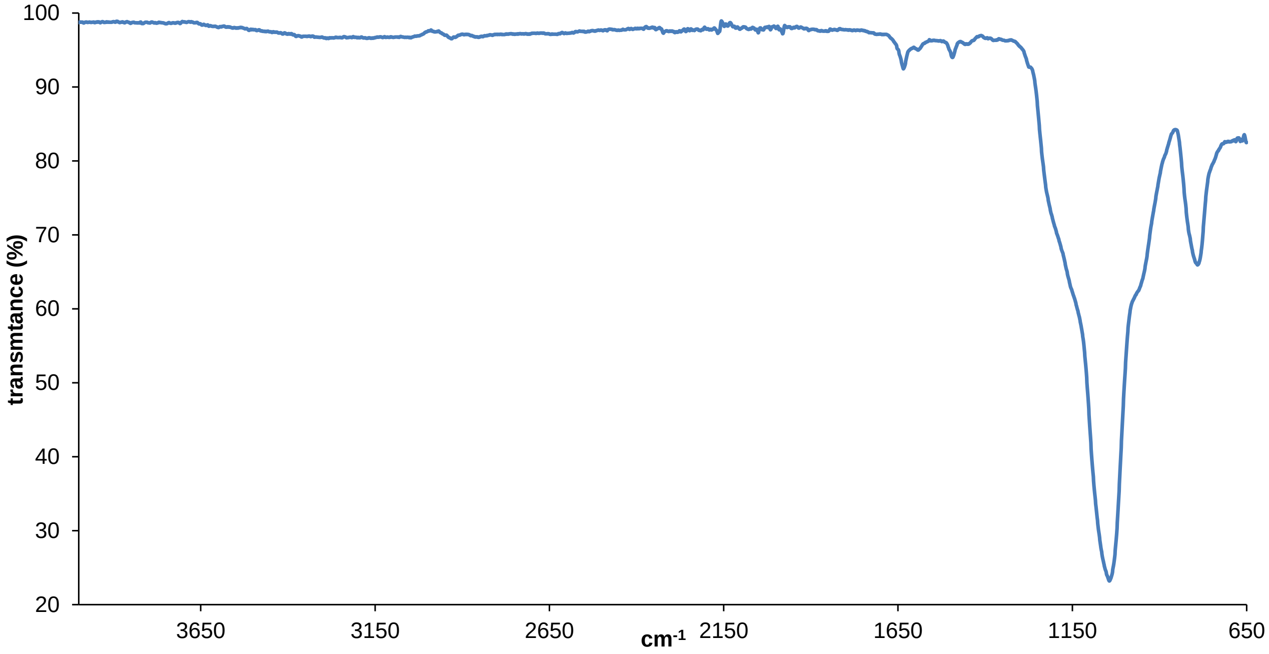

Expansion of 4000-1400 cm^{-1}

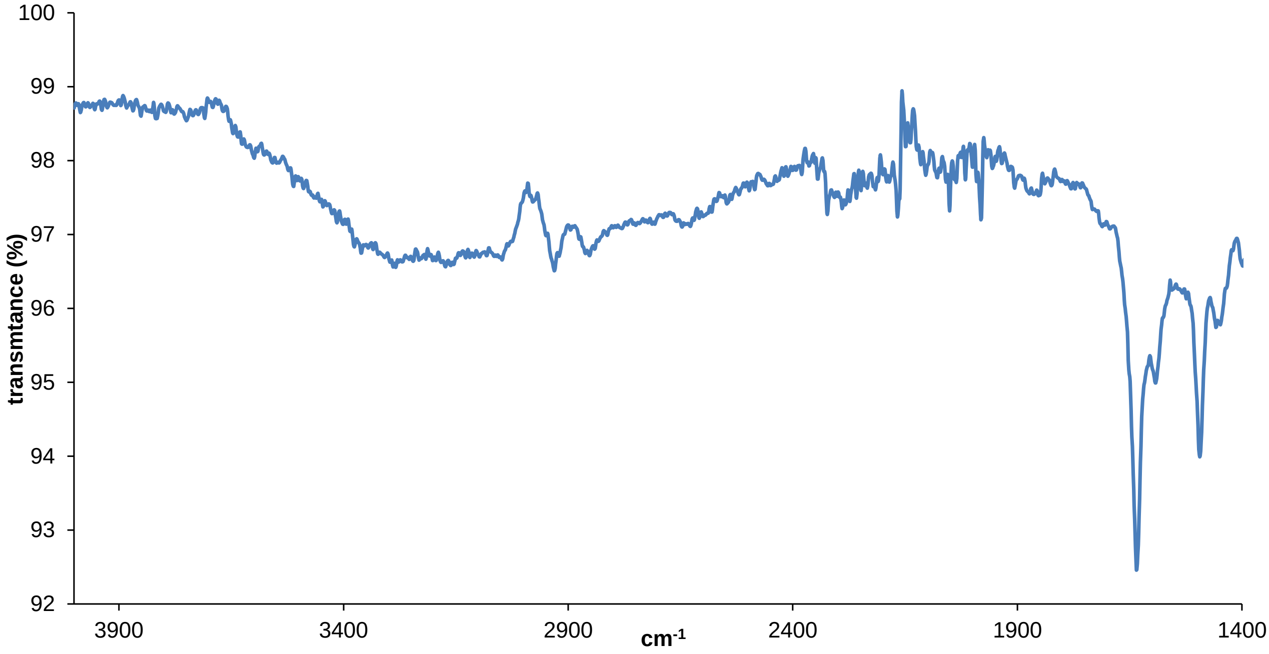

Solid State $^{13}C\{^{1}H\}$ NMR Spectrum (100 MHz)

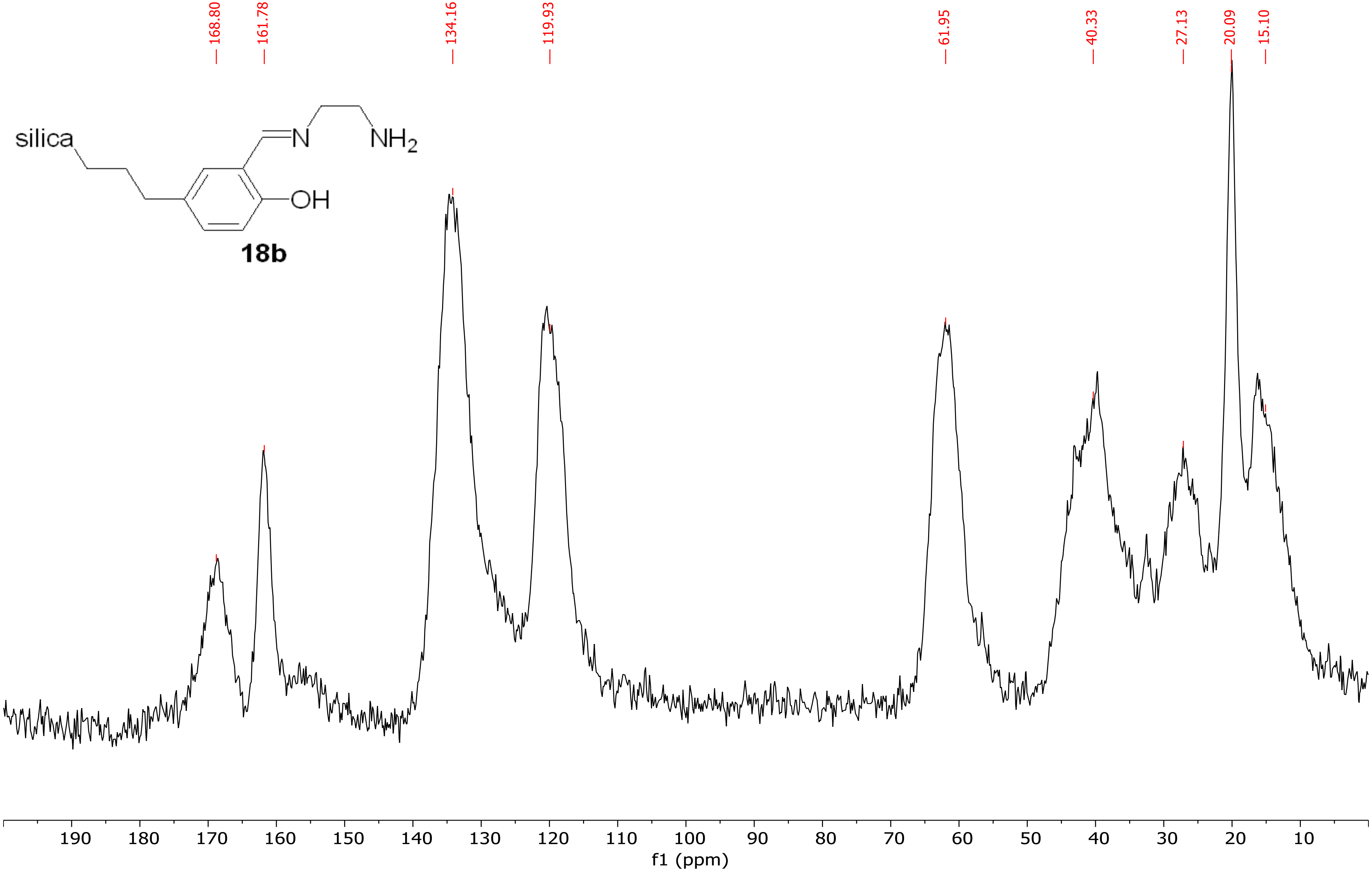

Silica-supported amine 18c

Analysis

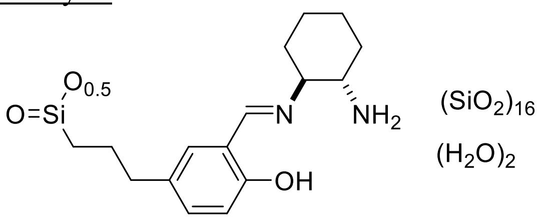

Chemical Formula: $C_{16}H_{27}N_2O_{36.5}Si_{17}$
Molecular Weight: 1308.8145
Elemental Analysis: C, 14.68; H, 2.08

Found: C, 14.5; H, 1.9

Mass of water:2x18 = 36
So predicted %water: = 2.75%
TGA weight loss below 100 ºC: = 2.7%

% yield calculation:
Product should contain
0.65 mmol of amine with RMM of 311 = 311 x 0.65 mg = 202.15 mg
10.4 mmol of SiO_2 with RMM of 60 = 60 x 10.4 mg = 624 mg
1.3 mmol of H_2O with RMM of 18 = 18 x 1.3 mg = 23.4 mg
So 100% yield = 850 mg

Actual mass of product = 500 mg
So %yield = 59%

Loading calculation
1.309 g contains 1 mmol of amine
So loading is: 1 / 1.309 = 0.76 mmol of amine per gram

Thermogravimetric Analysis

Full trace

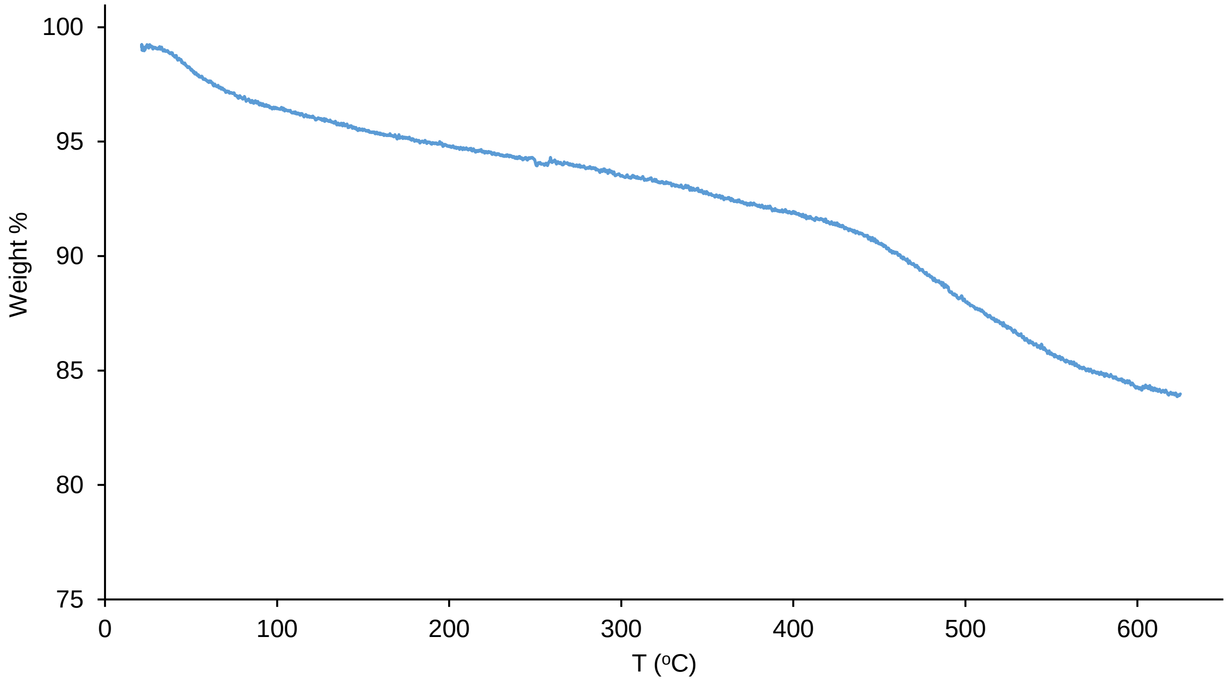

Expansion of region below 150 ºC

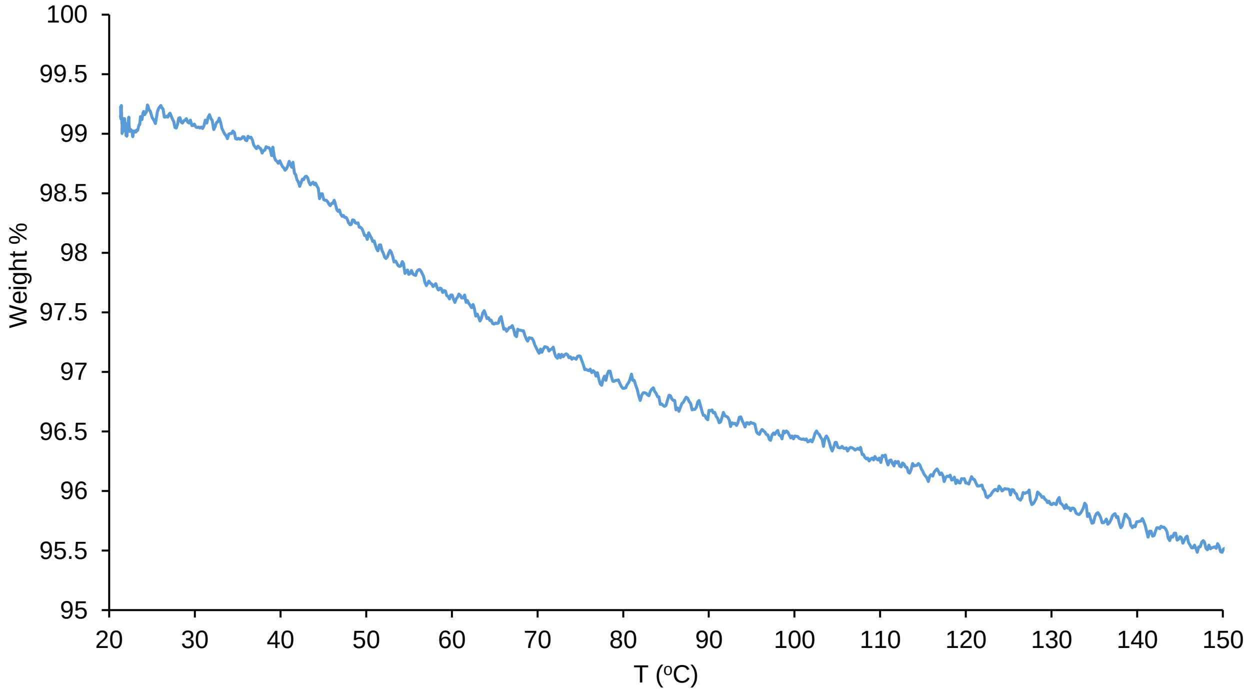

IR spectrum

Full spectrum

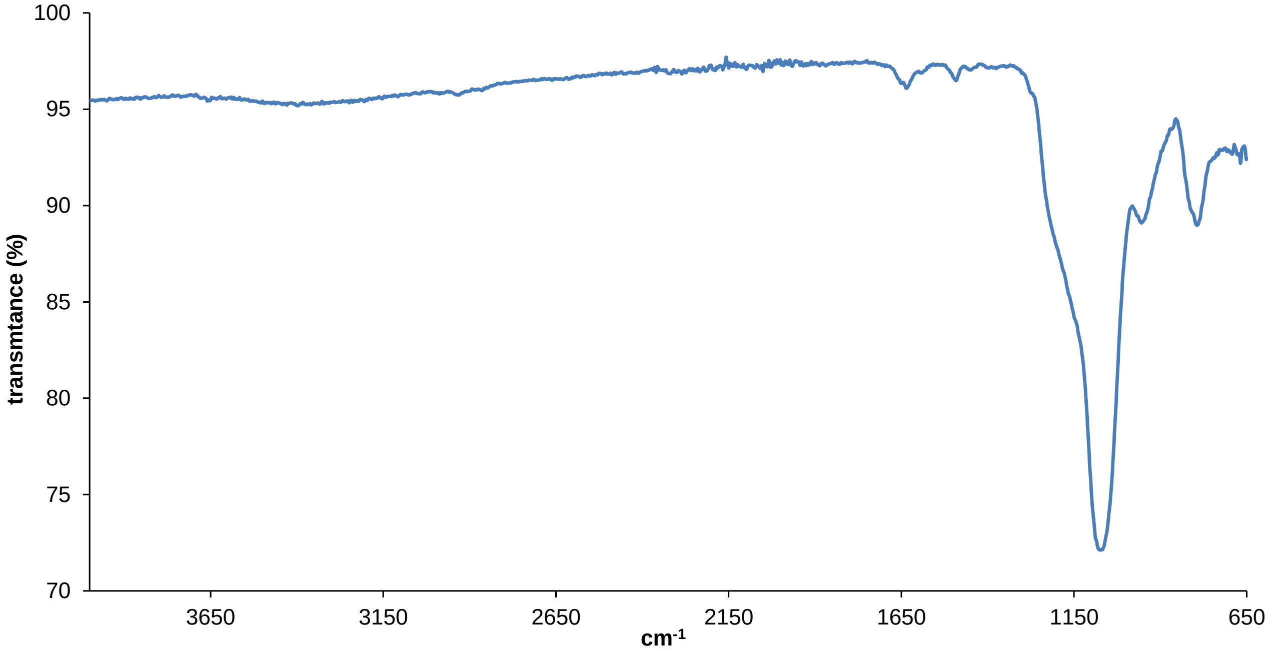

Expansion of 4000-1400 cm^{-1}

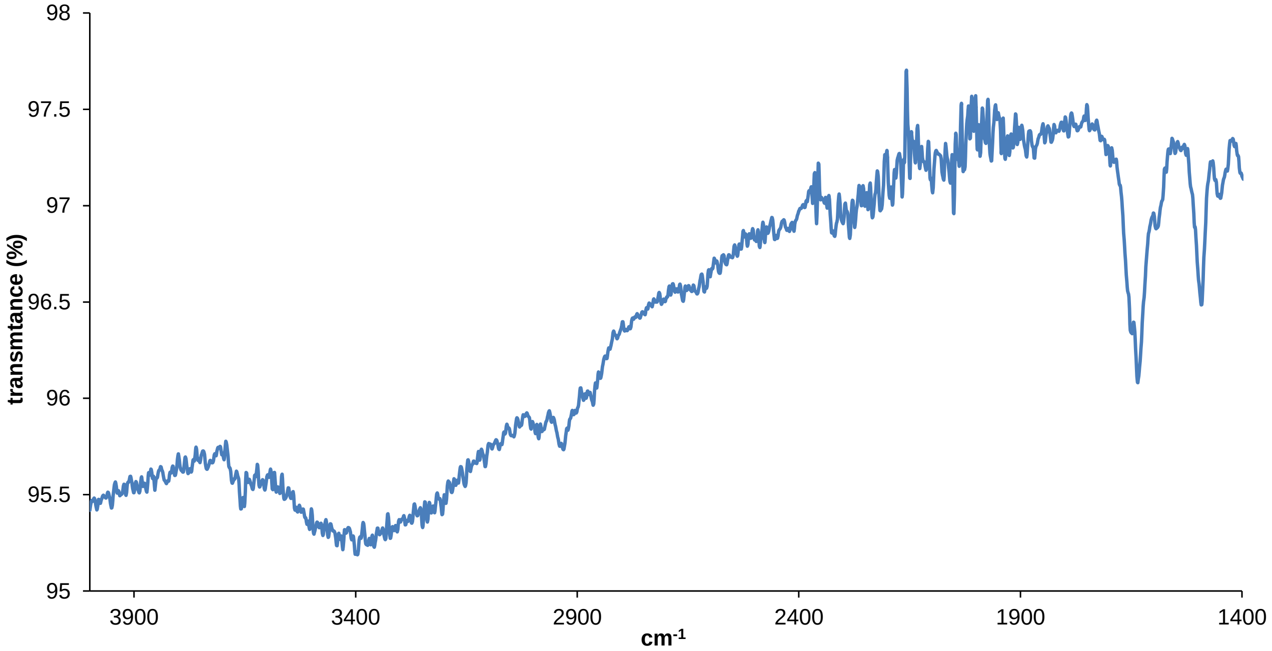

Solid State $^{13}C\{^{1}H\}$ NMR Spectrum (100 MHz)

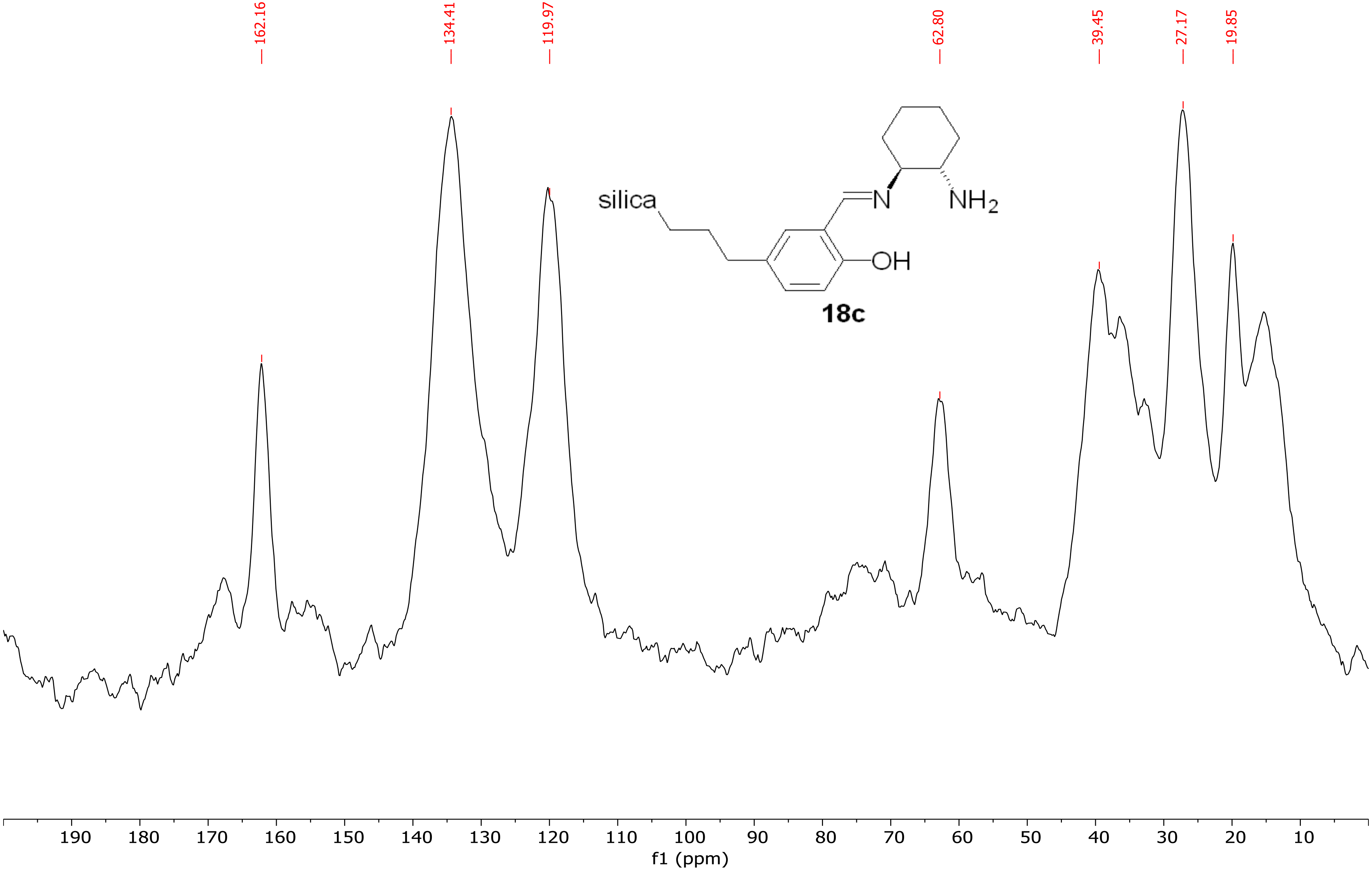

Silica-supported salophen 19a

Analysis

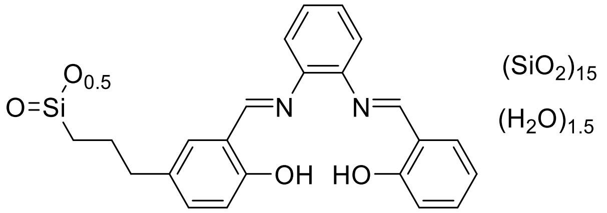

Chemical Formula: $C_{23}H_{24}N_2O_{35}Si_{16}$
Molecular Weight: 1337.78
Elemental Analysis: C, 20.65; H, 1.81%

Found: C, 20.6; H, 1.9%.

Mass of water: 1.5x18 = 27
So predicted %water: = 2.0%
TGA weight loss below 100 ºC: = 2.0%

% yield calculation:
Product should contain
0.63 mmol of salophen unit with RMM of 409.5 = 409.5 x 0.63 mg = 258 mg
9.45 mmol of SiO_2 with RMM of 60 = 60 x 9.45 mg = 567 mg
0.945 mmol of H_2O with RMM of 18 = 18 x 0.7 mg = 17 mg
So 100% yield = 842 mg

Actual mass of product = 600 mg
So %yield = 71%

Loading calculation
1.338 g contains 1 mmol of salophen
So loading is: 1 / 1.338 = 0.75 mmol of salophen per gram

Thermogravimetric Analysis

Full trace

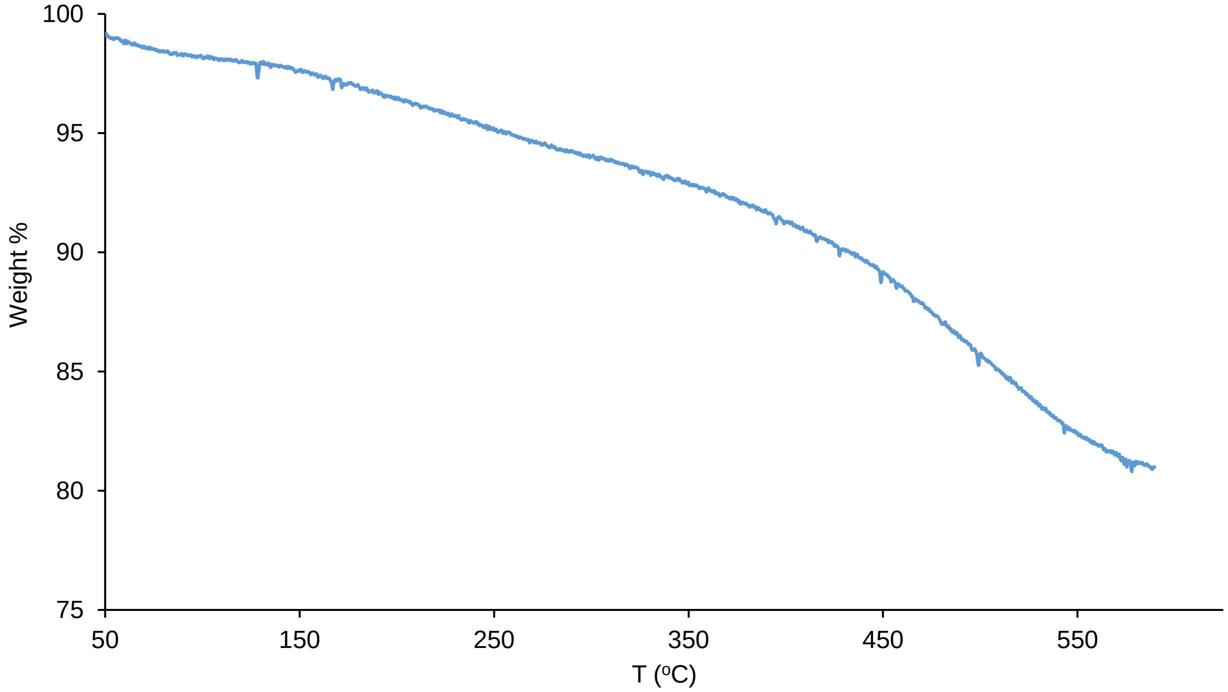

Expansion of region below 150 ºC

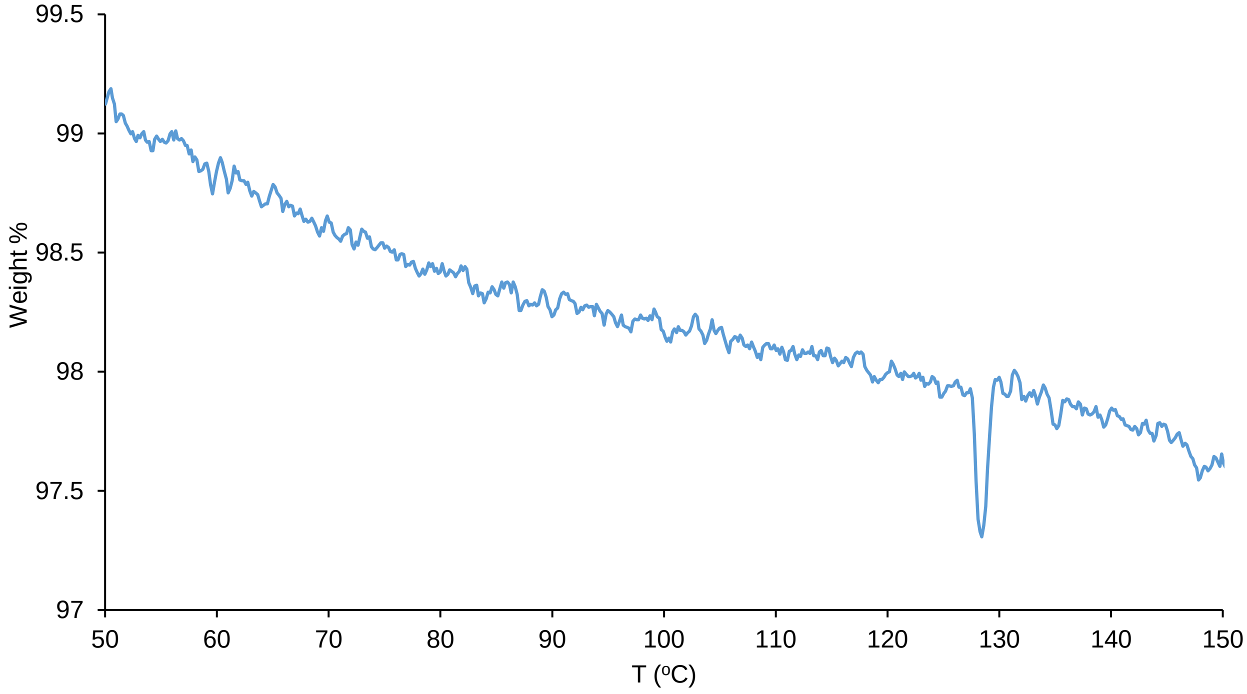

IR spectrum

Full spectrum

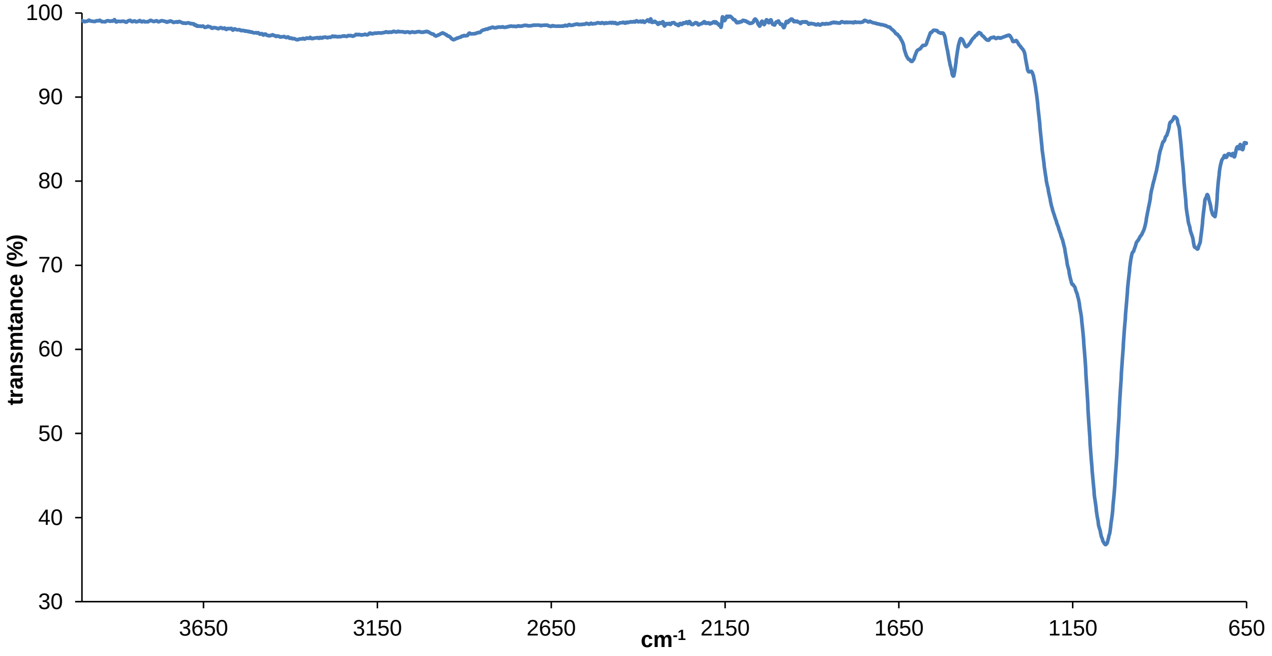

Expansion of 4000-1400 cm^{-1}

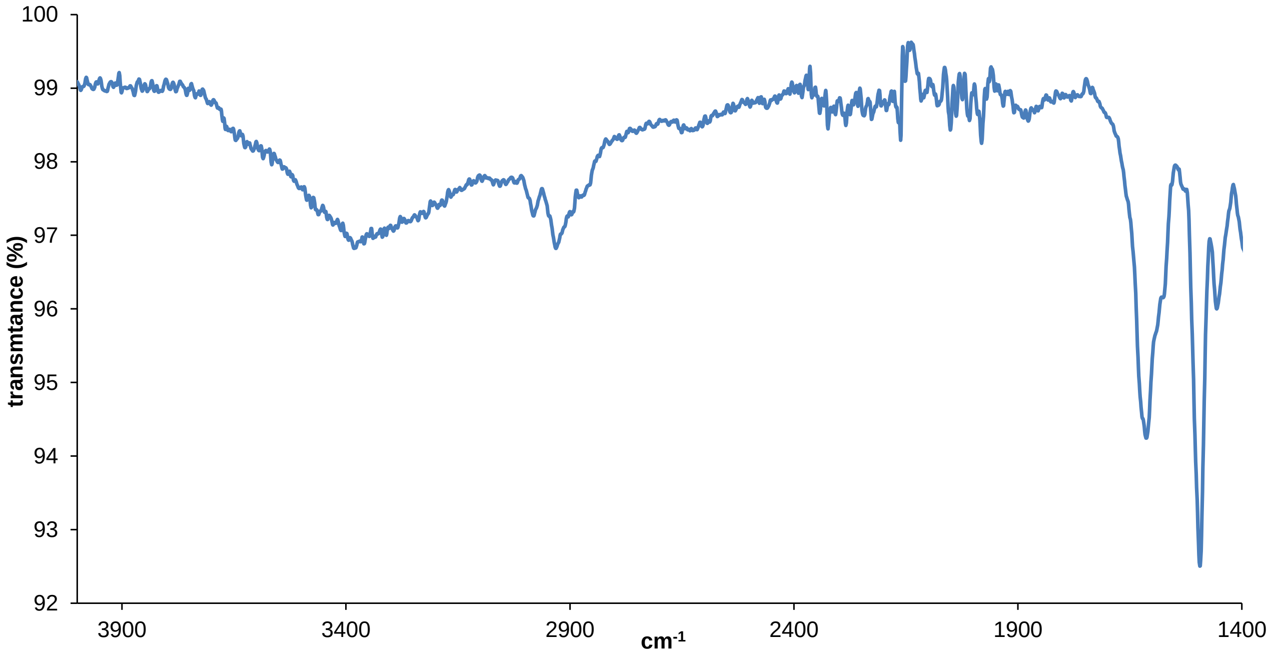

Solid State $^{13}C\{^1H\}$ NMR Spectrum (100 MHz)

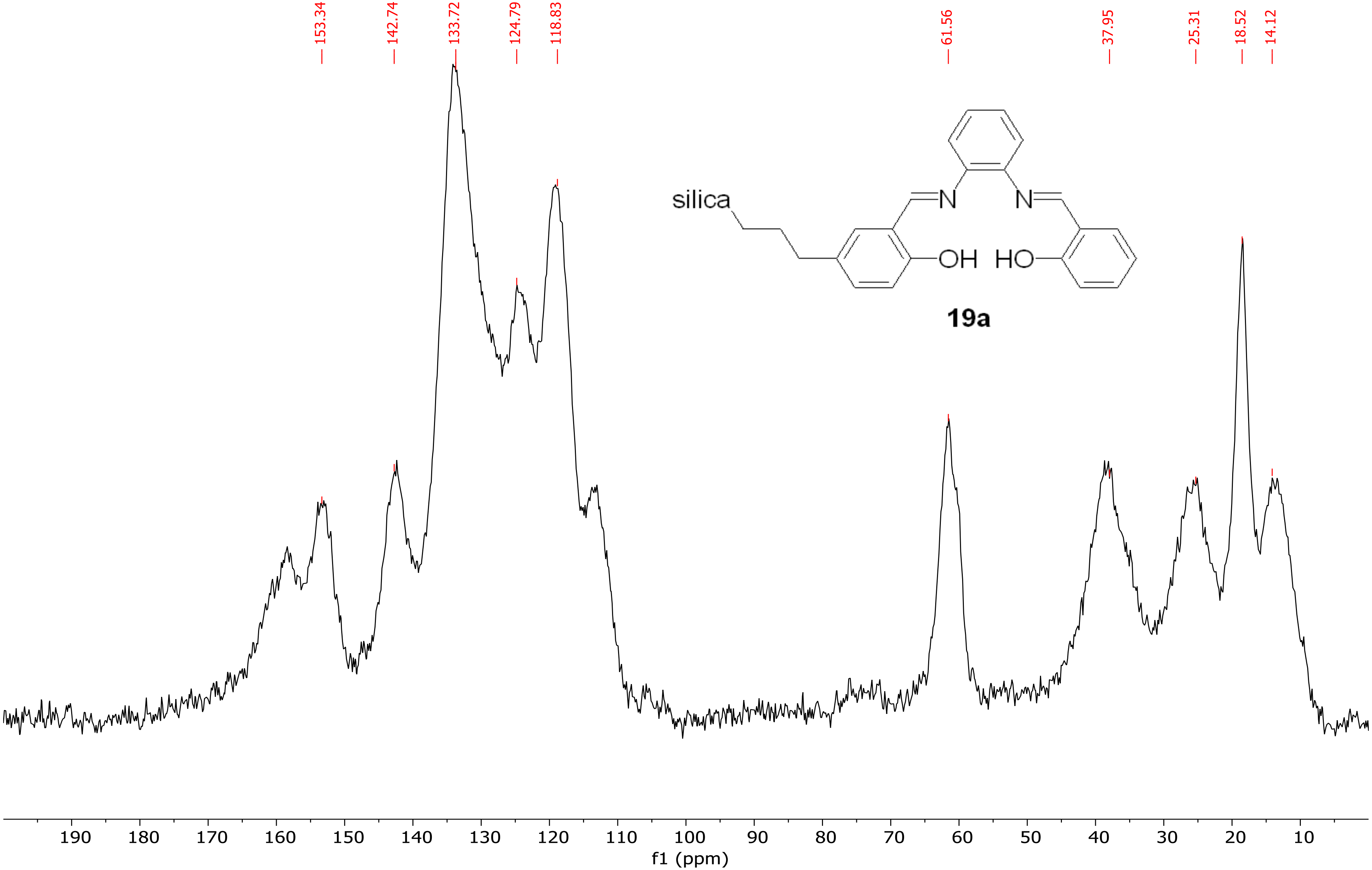

Silica-supported salophen 19b

Analysis

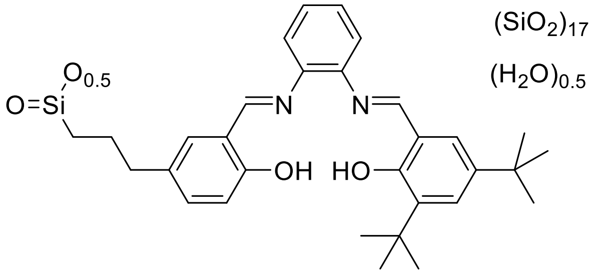

Chemical Formula: $C_{31}H_{38}N_2O_{38}Si_{18}$
Molecular Weight: 1552.1510
Elemental Analysis: C, 23.99; H, 2.47; N, 1.80

Found: C, 23.8; H, 2.4; N, 1.8.

Mass of water: 0.5x18 = 9
So predicted %water: = 0.6%
TGA weight loss below 100 °C: = 0.5%

% yield calculation:
Product should contain
0.63 mmol of salophen unit with RMM of 521.7 = 521.7 x 0.63 mg = 328.7 mg
10.71 mmol of SiO_2 with RMM of 60 = 60 x 10.71 mg = 642.6 mg
0.32 mmol of H_2O with RMM of 18 = 18 x 0.32 mg = 5.8 mg
So 100% yield = 977 mg

Actual mass of product = 645 mg
So %yield = 66%

Loading calculation
1.552 g contains 1 mmol of salophen
So loading is: 1 / 1.552 = 0.64 mmol of salophen per gram

Thermogravimetric Analysis

Full trace

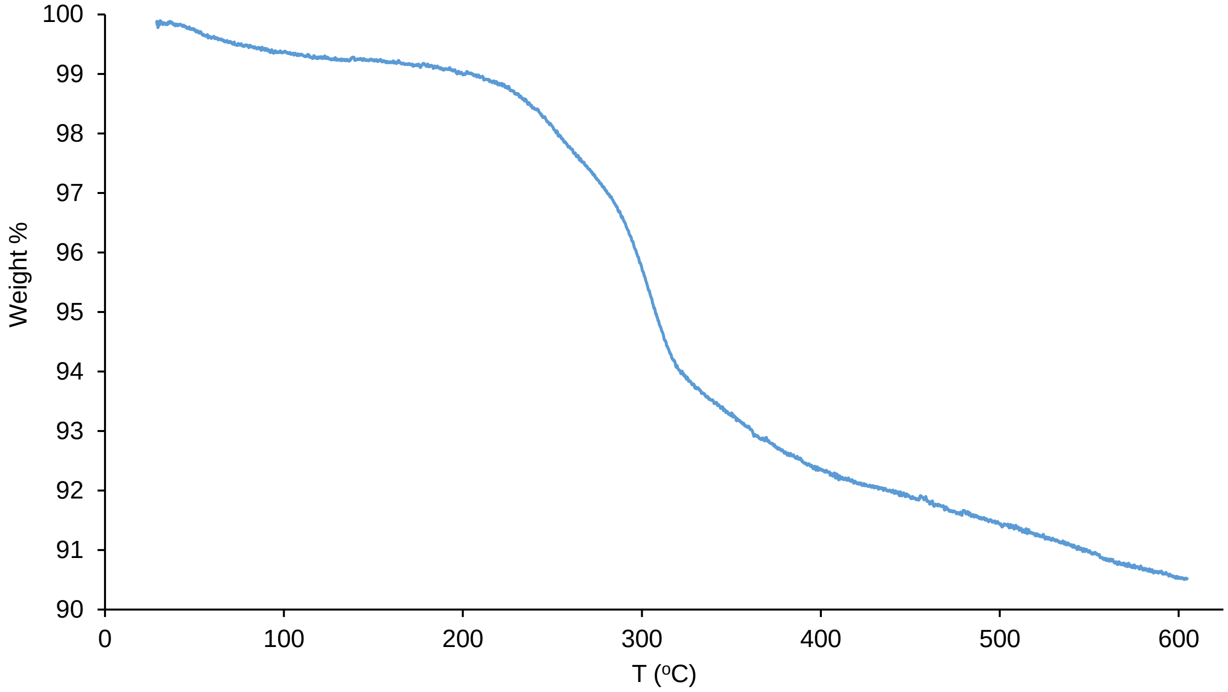

Expansion of region below 150 ºC

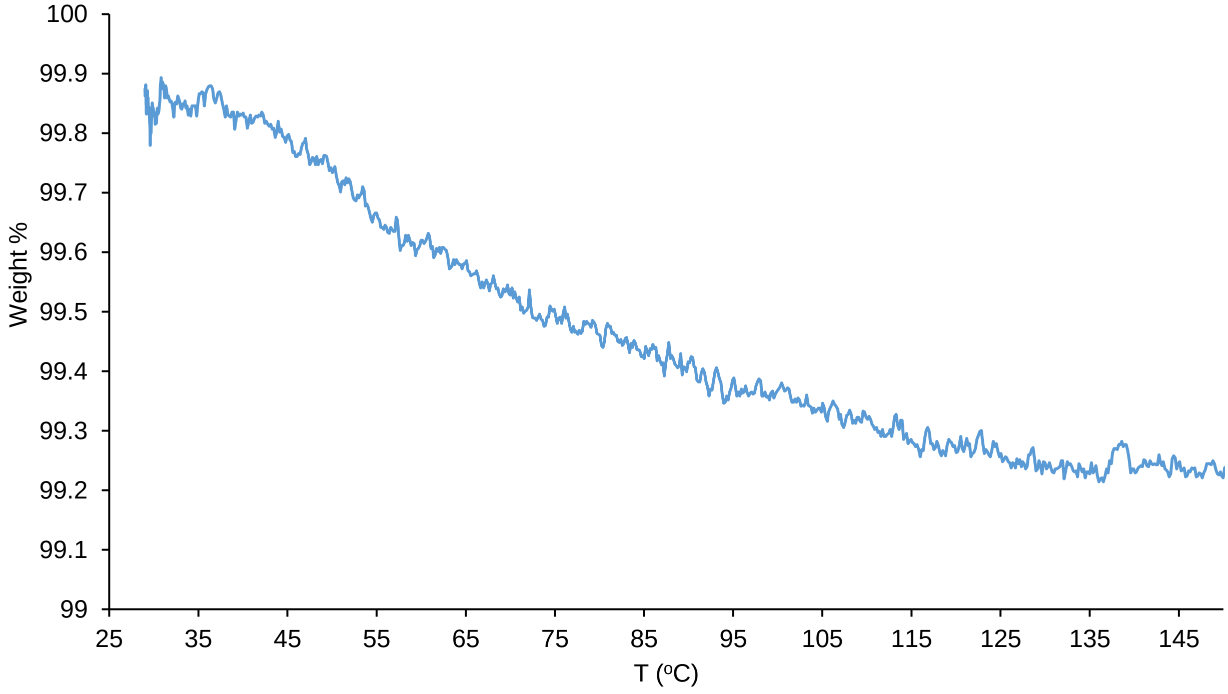

IR spectrum

Full spectrum

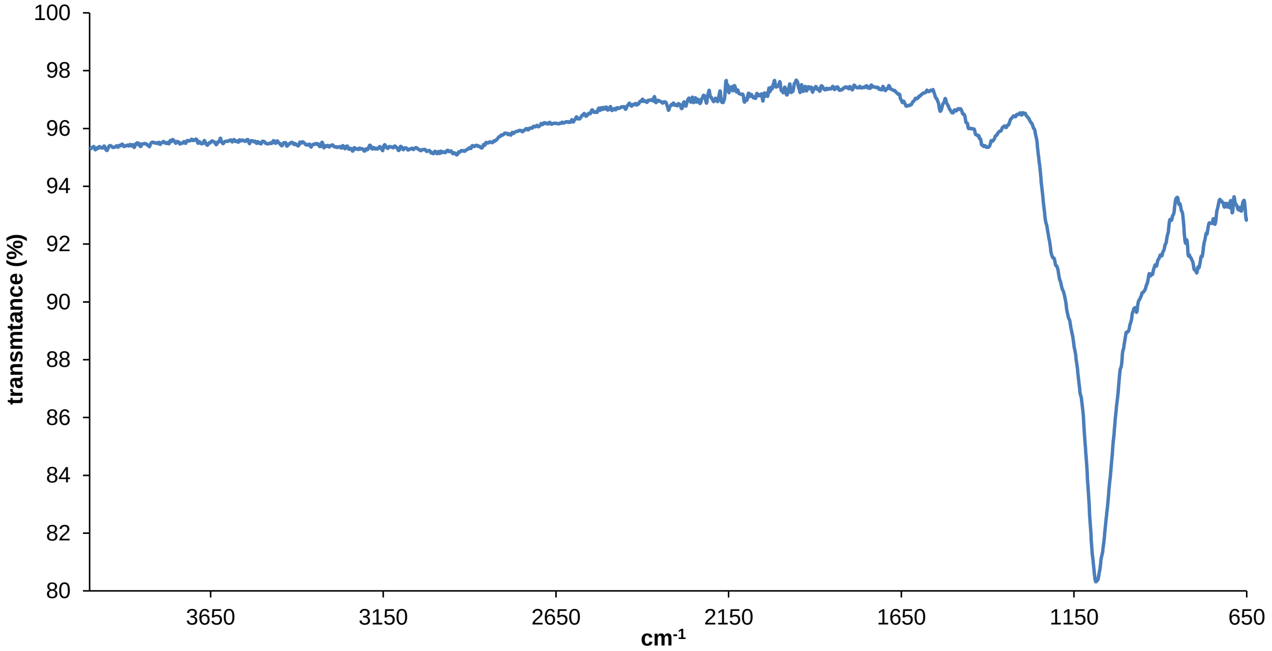

Expansion of 4000-1400 cm^{-1}

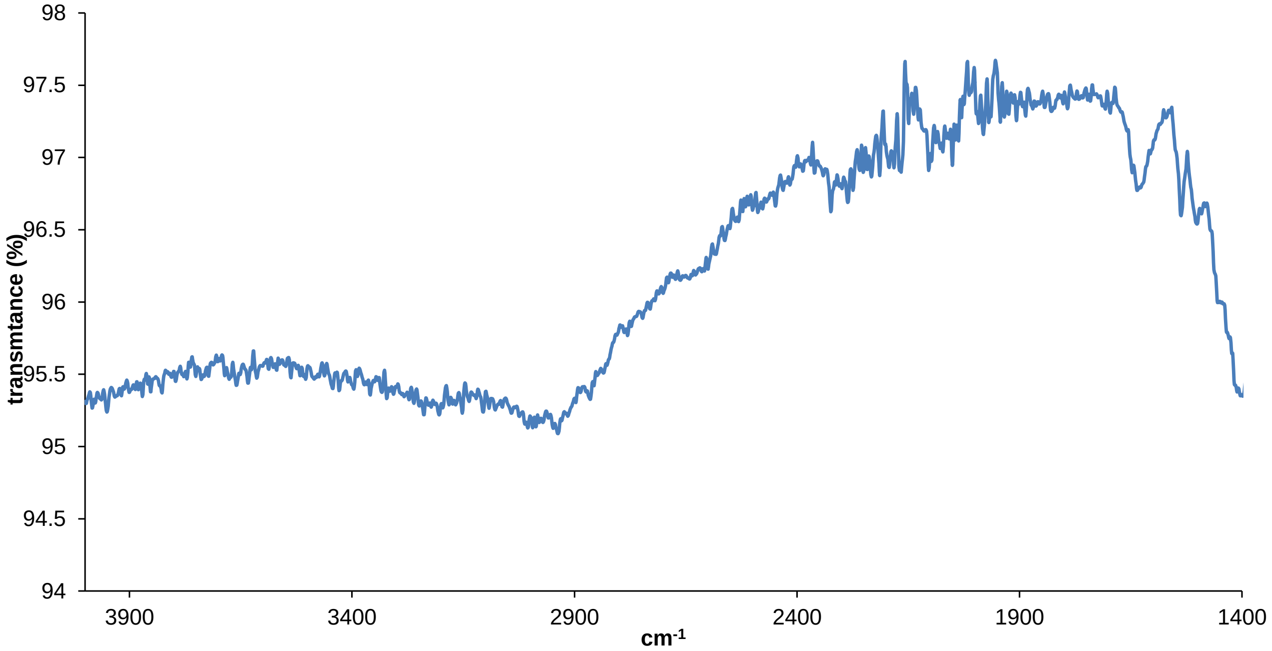

Solid State $^{13}C\{^{1}H\}$ NMR Spectrum (100 MHz)

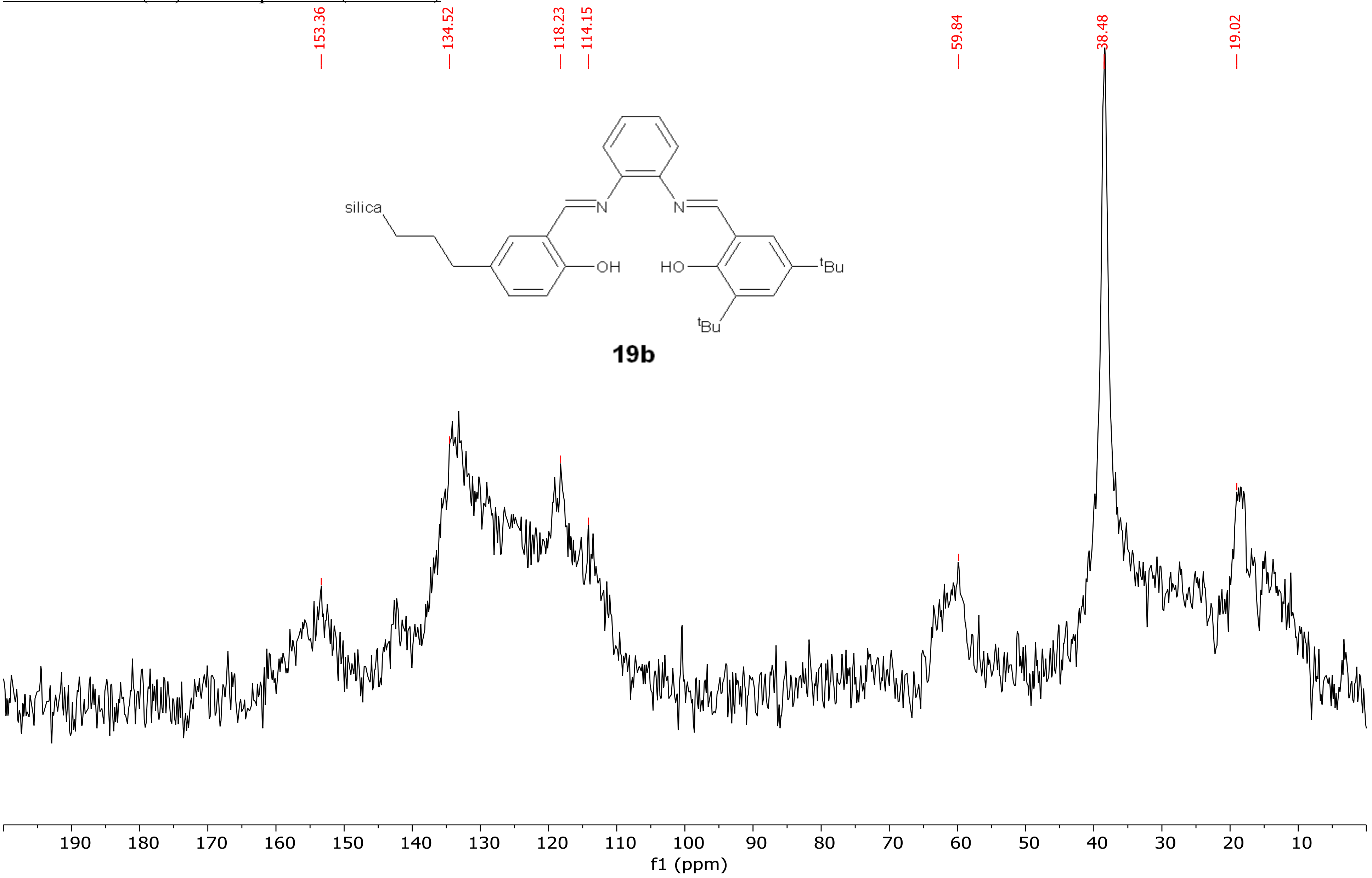

Silica-supported salen 19c

Analysis

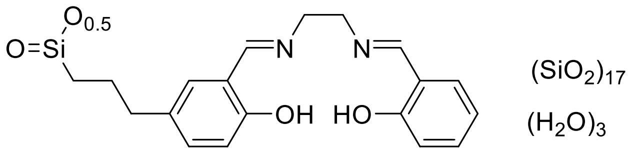

Chemical Formula: $C_{19}H_{27}N_2O_{40.5}Si_{18}$
Molecular Weight: 1436.93
Elemental Analysis: C, 15.88; H, 1.89; N, 1.95

Found: C, 16.1; H, 1.8; N, 1.9.

Mass of water: 3x18 = 54
So predicted %water: = 3.8%
TGA weight loss below 100 ºC: = 3.7%

% yield calculation:
Product should contain
0.34 mmol of salophen unit with RMM of 361.5 = 361.5 x 0.34 mg = 122.9 mg
5.78 mmol of SiO_2 with RMM of 60 = 60 x 5.78 mg = 346.8 mg
1.02 mmol of H_2O with RMM of 18 = 18 x 1.02 mg = 18.4 mg
So 100% yield = 488 mg

Actual mass of product = 240 mg
So %yield = 49%

Loading calculation
1.437 g contains 1 mmol of salophen
So loading is: 1 / 1.437 = 0.70 mmol of salophen per gram

Thermogravimetric Analysis

Full trace

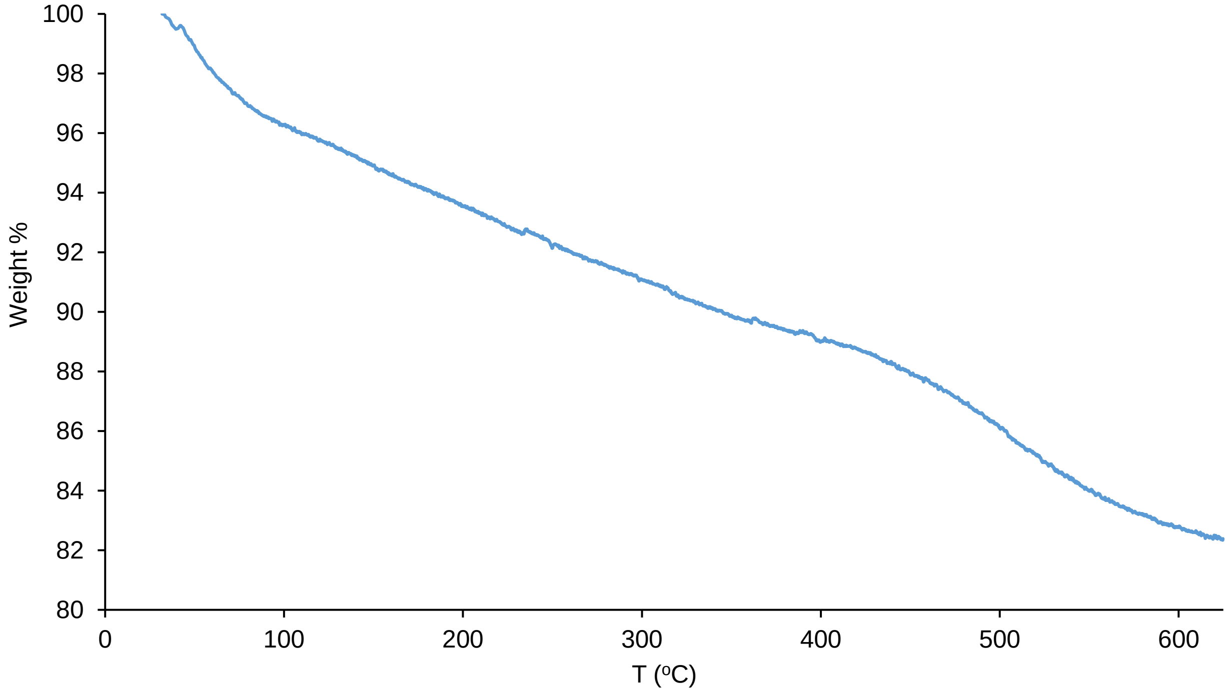

Expansion of region below 150 ºC

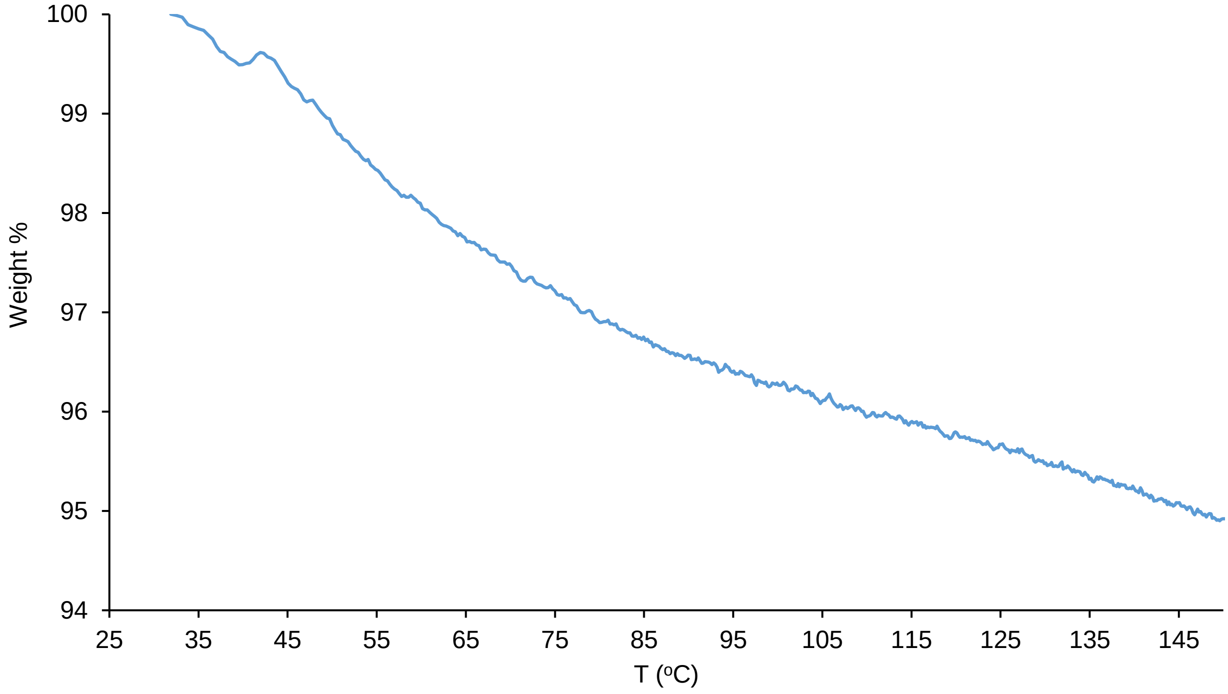

IR spectrum

Full spectrum

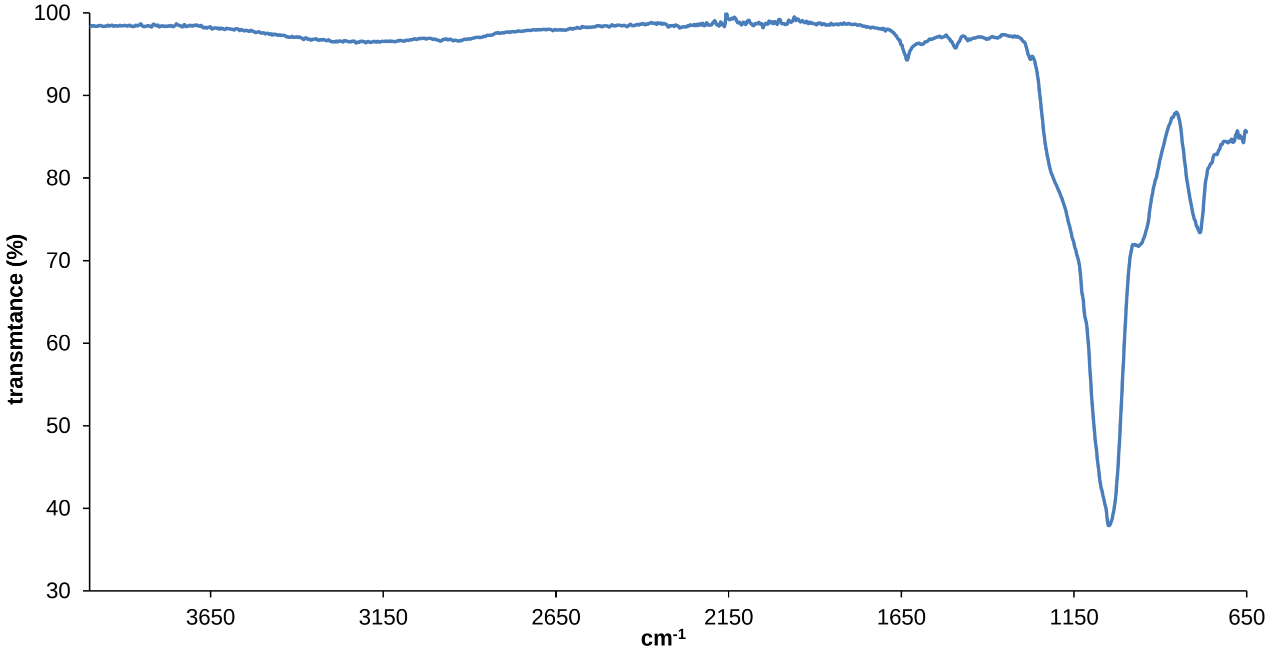

Expansion of 4000-1400 cm^{-1}

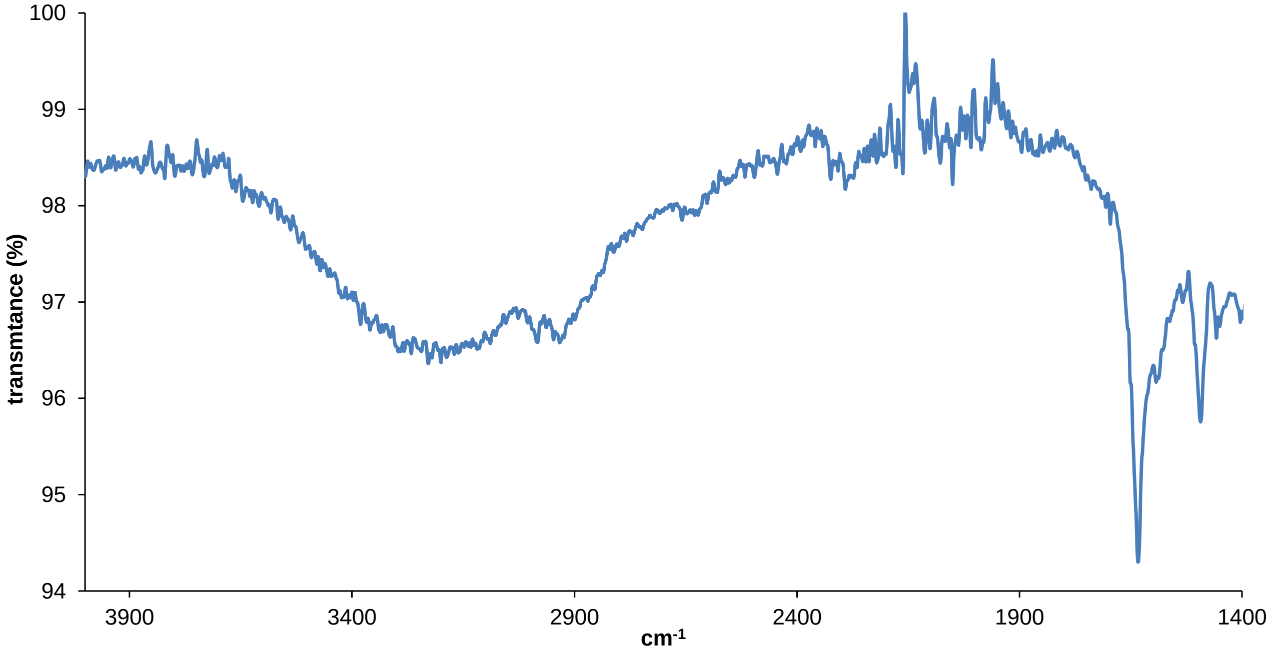

Solid State $^{13}C\{^{1}H\}$ NMR Spectrum (100 MHz)

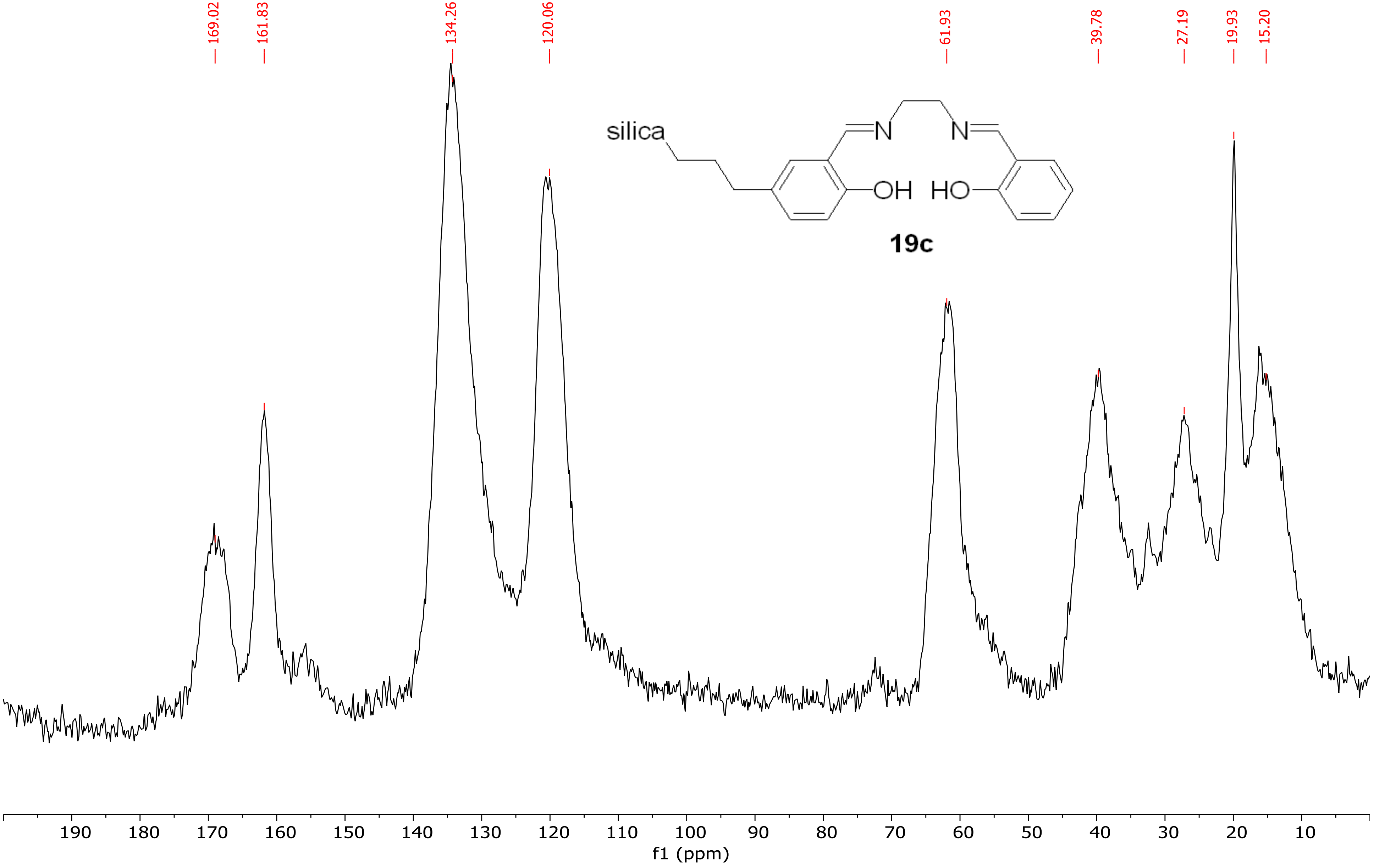

Silica-supported salen 19d

Analysis

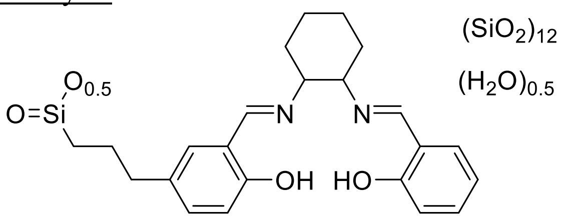

Chemical Formula: $C_{23}H_{28}N_2O_{28}Si_{13}$
Molecular Weight: 1145.5680
Elemental Analysis: C, 24.11; H, 2.46; N, 2.45

Found: C, 23.8; H, 2.4; N, 2.3%.

Mass of water: 0.5x18 = 9
So predicted %water: = 0.8%
TGA weight loss below 100 ºC: = 0.8%

% yield calculation:
Product should contain
0.63 mmol of salophen unit with RMM of 415.6 = 415.6 x 0.63 mg = 261.8 mg
7.56 mmol of SiO_2 with RMM of 60 = 60 x 7.56 mg = 453.6 mg
0.32 mmol of H_2O with RMM of 18 = 18 x 0.32 mg = 5.8 mg
So 100% yield = 721 mg

Actual mass of product = 400 mg
So %yield = 55%

Loading calculation
1.146 g contains 1 mmol of salen
So loading is: 1 / 1.146 = 0.87 mmol of salen per gram

Thermogravimetric Analysis

Full trace

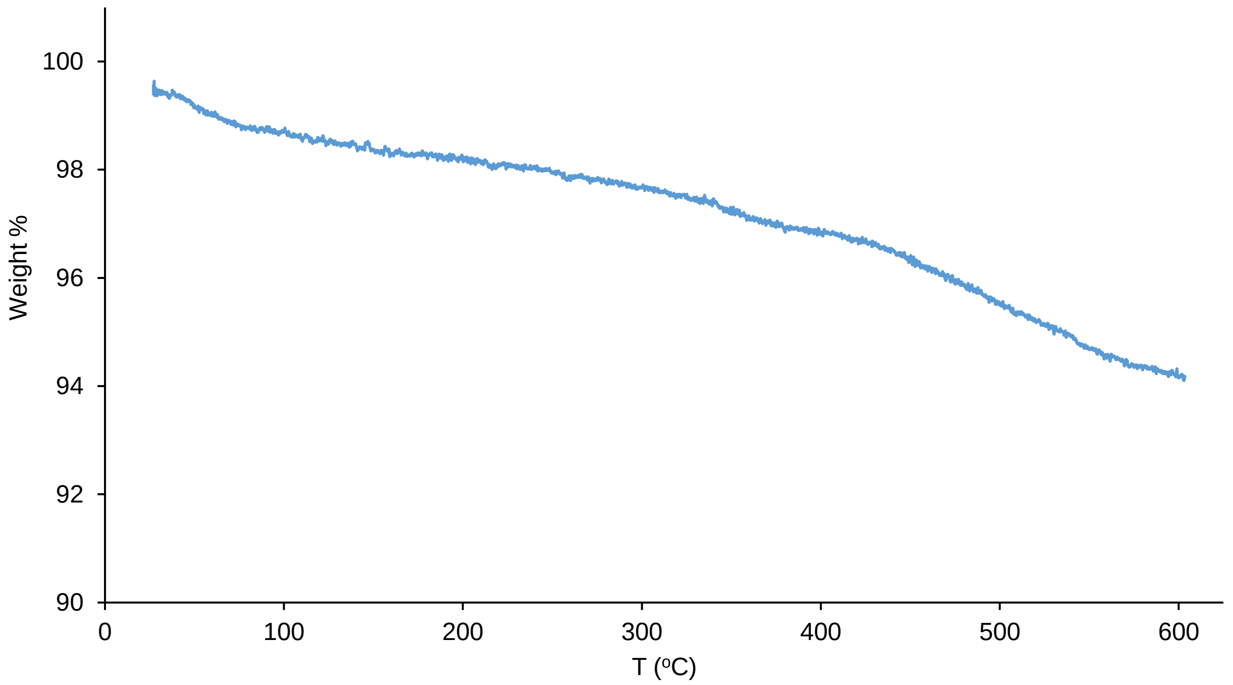

Expansion of region below 150 ºC

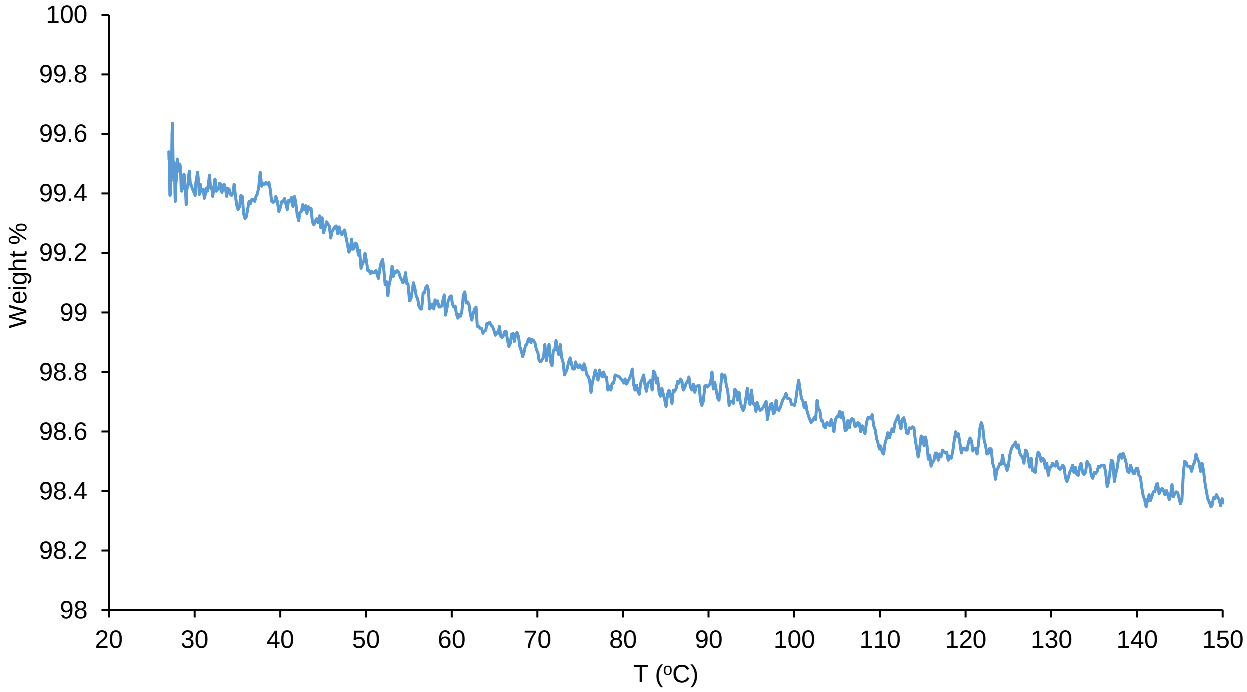

IR spectrum

Full spectrum

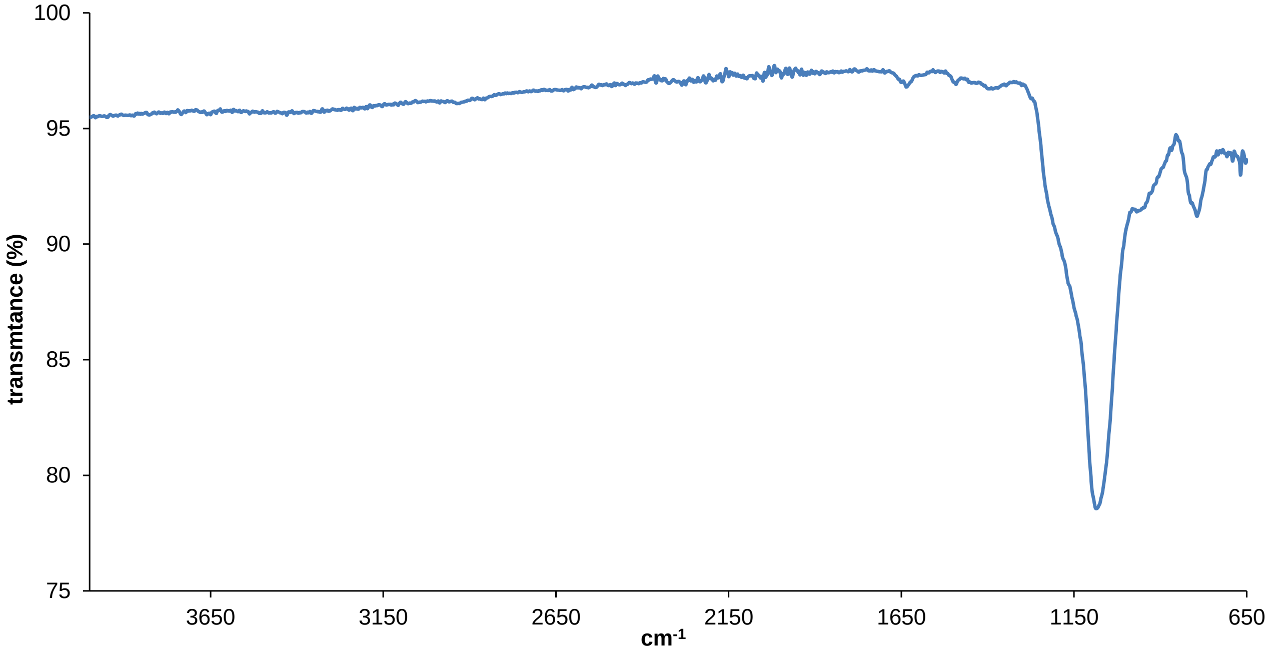

Expansion of 4000-1400 cm^{-1}

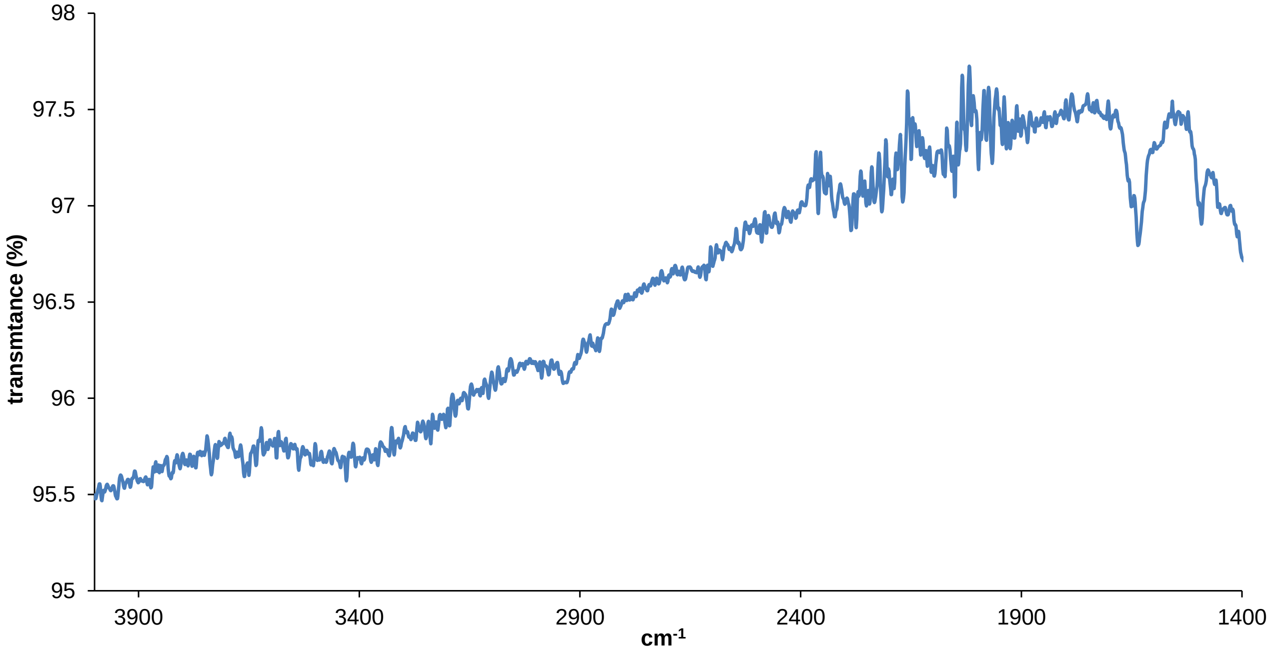

Solid State $^{13}C\{^{1}H\}$ NMR Spectrum (100 MHz)

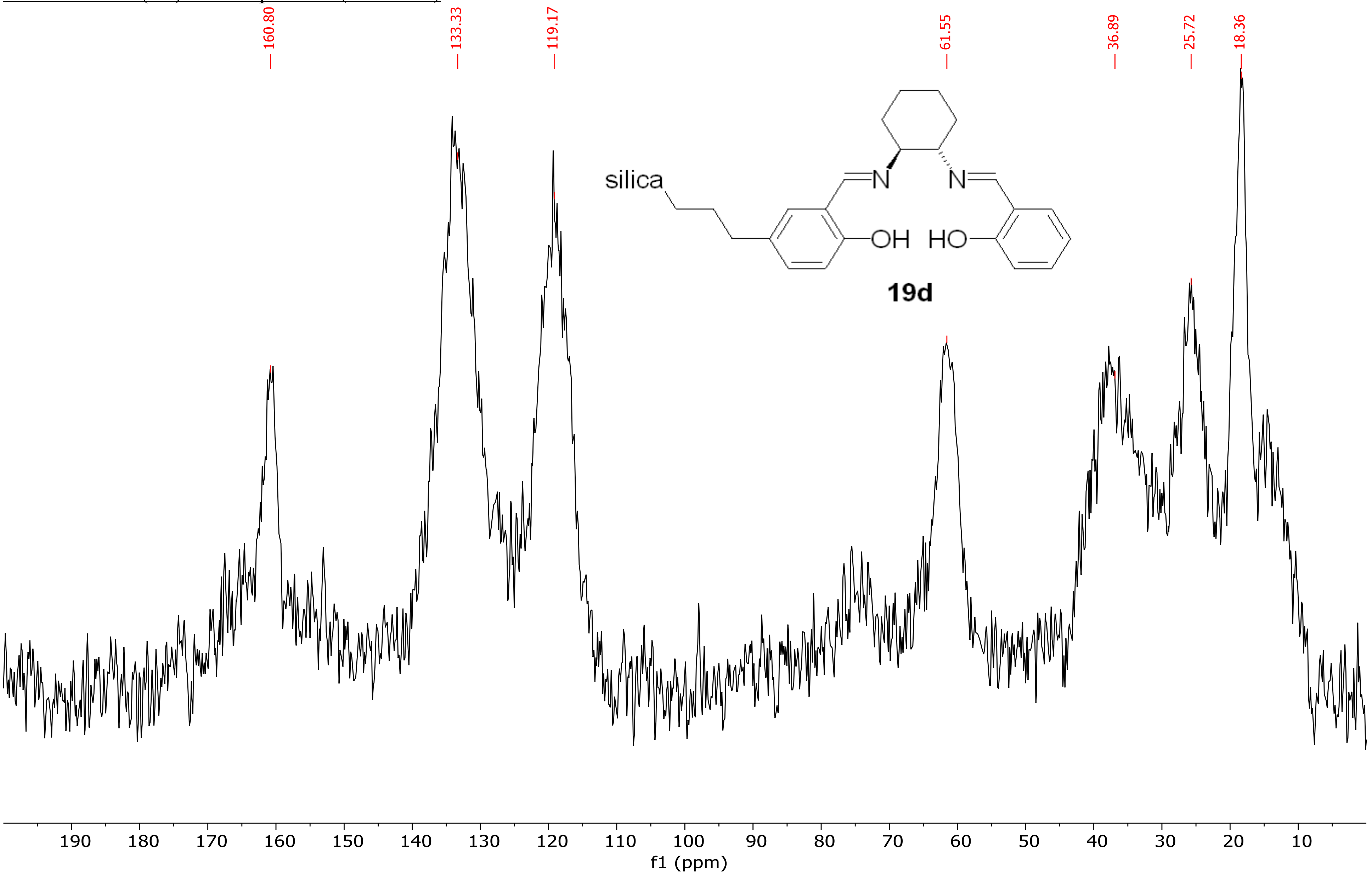

Silica-supported salophen 19e

Analysis

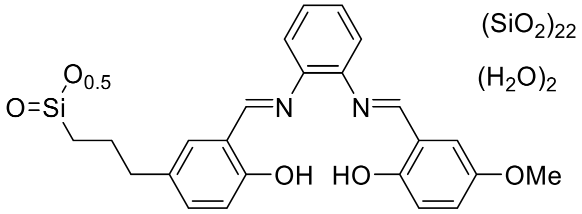

Chemical Formula: $C_{24}H_{27}N_2O_{50.5}Si_{23}$
Molecular Weight: 1797.40
Elemental Analysis: C, 16.04; H, 1.51%

Found: C, 16.1; H, 1.4%.

Mass of water: 2x18 = 36
So predicted %water: = 2.0%
TGA weight loss below 100 ºC: = 2.0%

% yield calculation:
Product should contain
0.63 mmol of salophen unit with RMM of 439.5 = 409.5 x 0.63 mg = 277 mg
13.86 mmol of SiO_2 with RMM of 60 = 60 x 13.86 mg = 832 mg
1.26 mmol of H_2O with RMM of 18 = 18 x 1.26 mg = 22.7 mg
So 100% yield = 1.132 g

Actual mass of product = 640 mg
So %yield = 56%

Loading calculation
1.797 g contains 1 mmol of salophen
So loading is: 1 / 1.797 = 0.56 mmol of salophen per gram

Thermogravimetric Analysis

Full trace

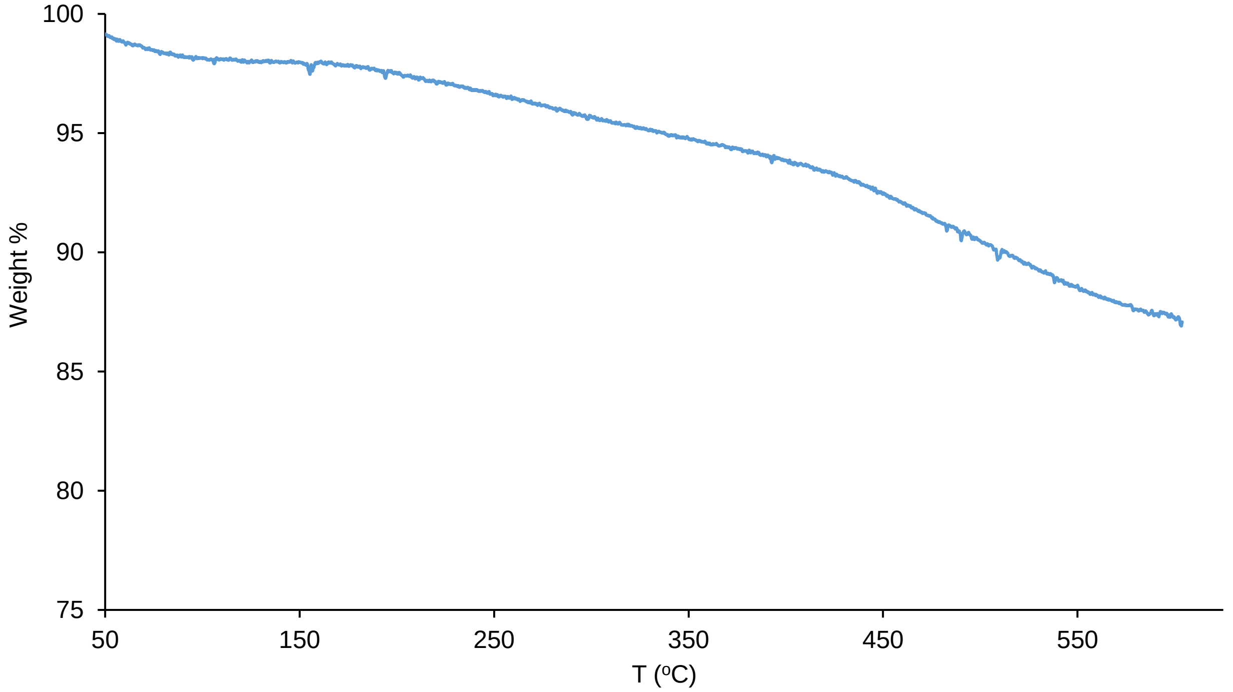

Expansion of region below 150 ºC

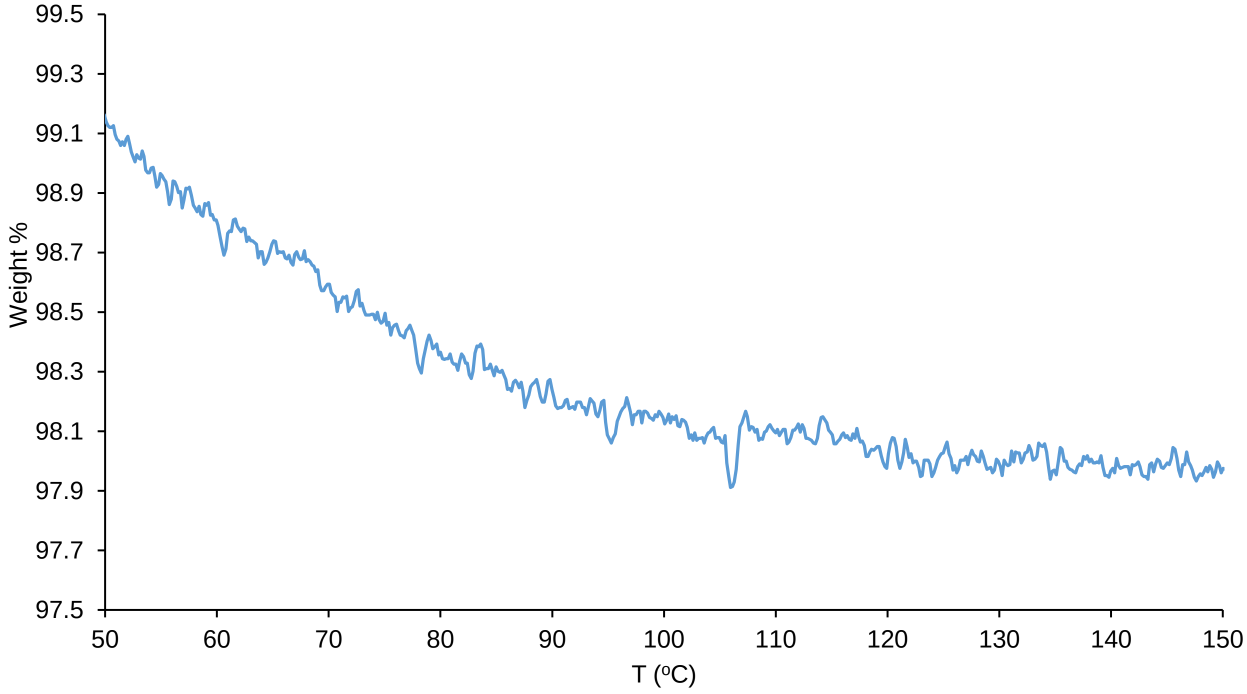

IR spectrum

Full spectrum

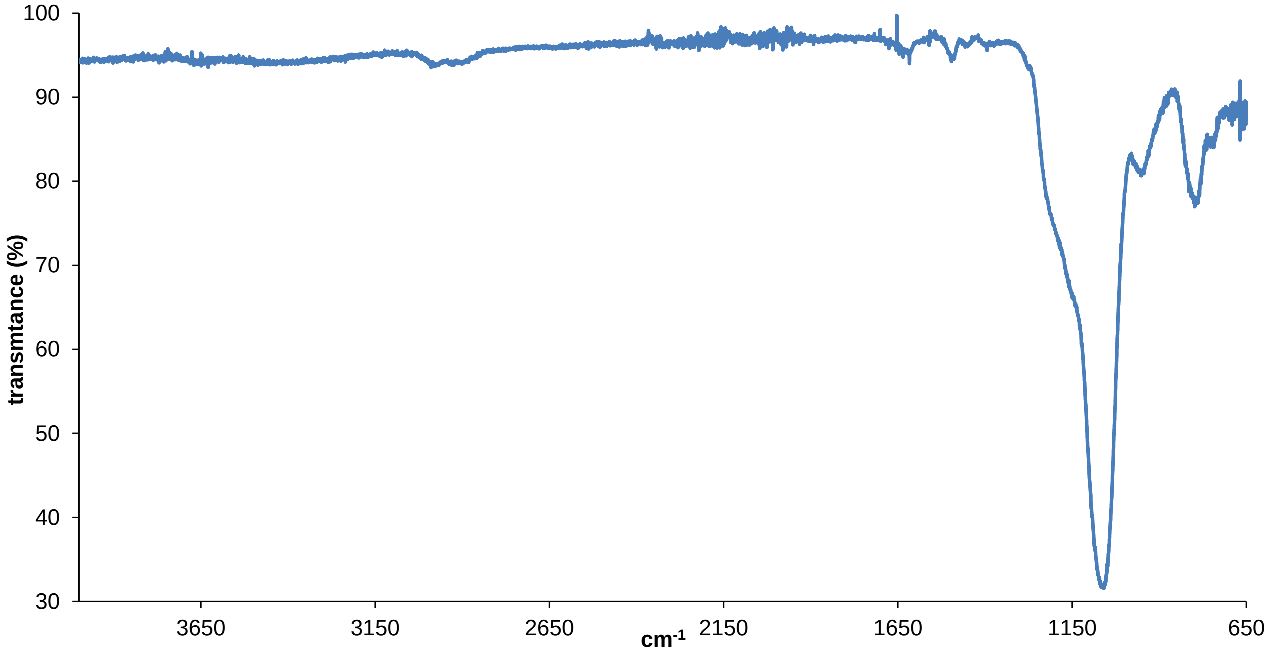

Expansion of 4000-1400 cm^{-1}

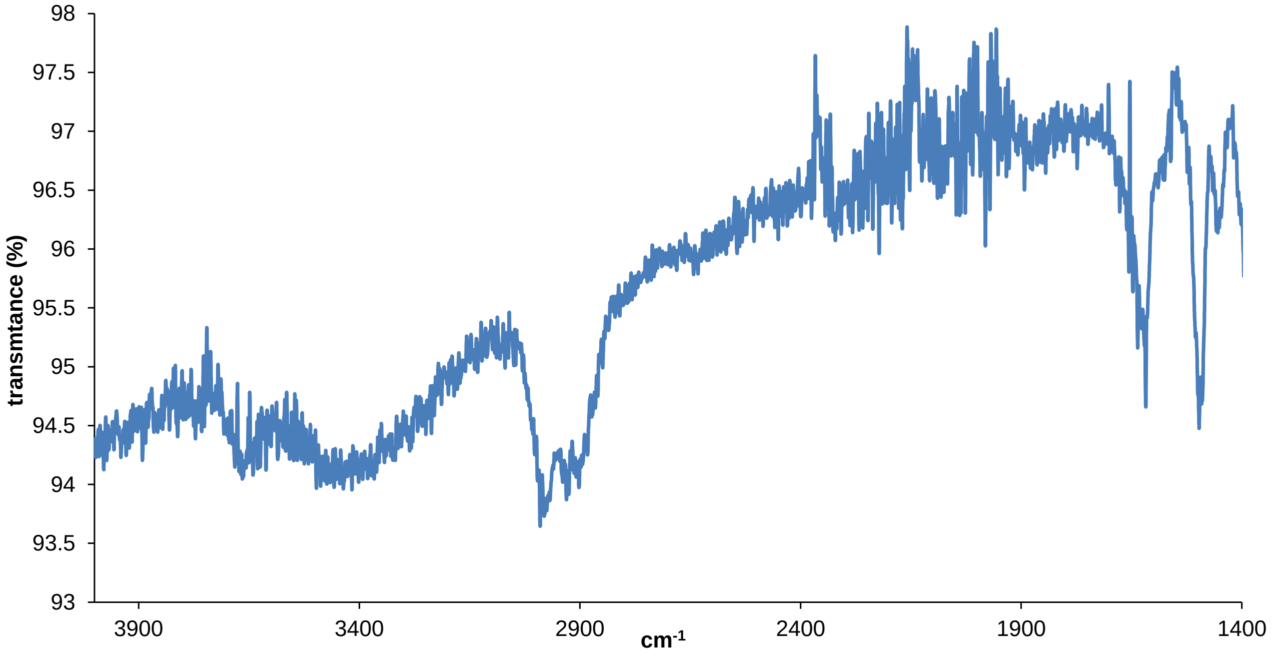

Solid State $^{13}C\{^{1}H\}$ NMR Spectrum (100 MHz)

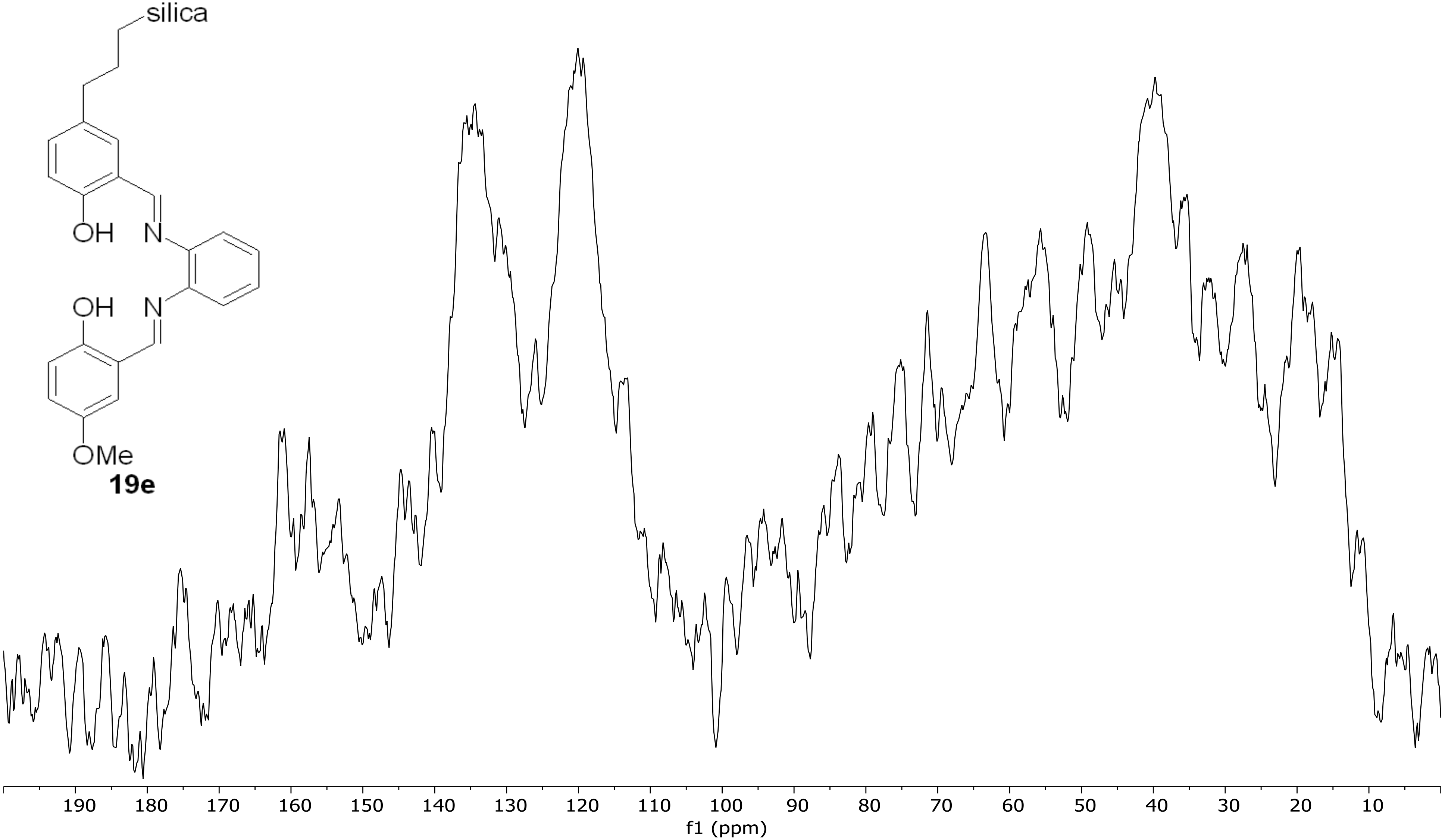

Silica-supported salophen 19f

Analysis

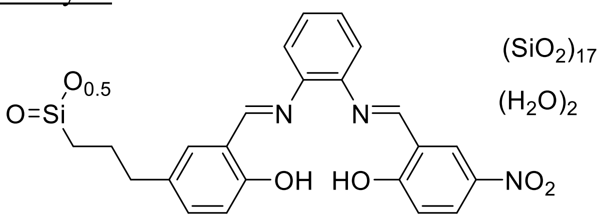

Chemical Formula: $C_{23}H_{24}N_3O_{41.5}Si_{18}$
Molecular Weight: 1511.95
Elemental Analysis: C, 18.27; H, 1.60%

Found: C, 18.6; H, 1.9%.

Mass of water: 2x18 = 36
So predicted %water: = 2.4%
TGA weight loss below 100 ºC: = 2.25%

% yield calculation:
Product should contain
0.63 mmol of salophen unit with RMM of 454.5 = 454.5 x 0.63 mg = 286 mg
10.71 mmol of SiO_2 with RMM of 60 = 60 x 10.71 mg = 642.6 mg
1.26 mmol of H_2O with RMM of 18 = 18 x 1.26 mg = 22.7 mg
So 100% yield = 951 mg

Actual mass of product = 630 mg
So %yield = 66%

Loading calculation
1.512 g contains 1 mmol of salophen
So loading is: 1 / 1.512 = 0.66 mmol of salophen per gram

Thermogravimetric Analysis

Full trace

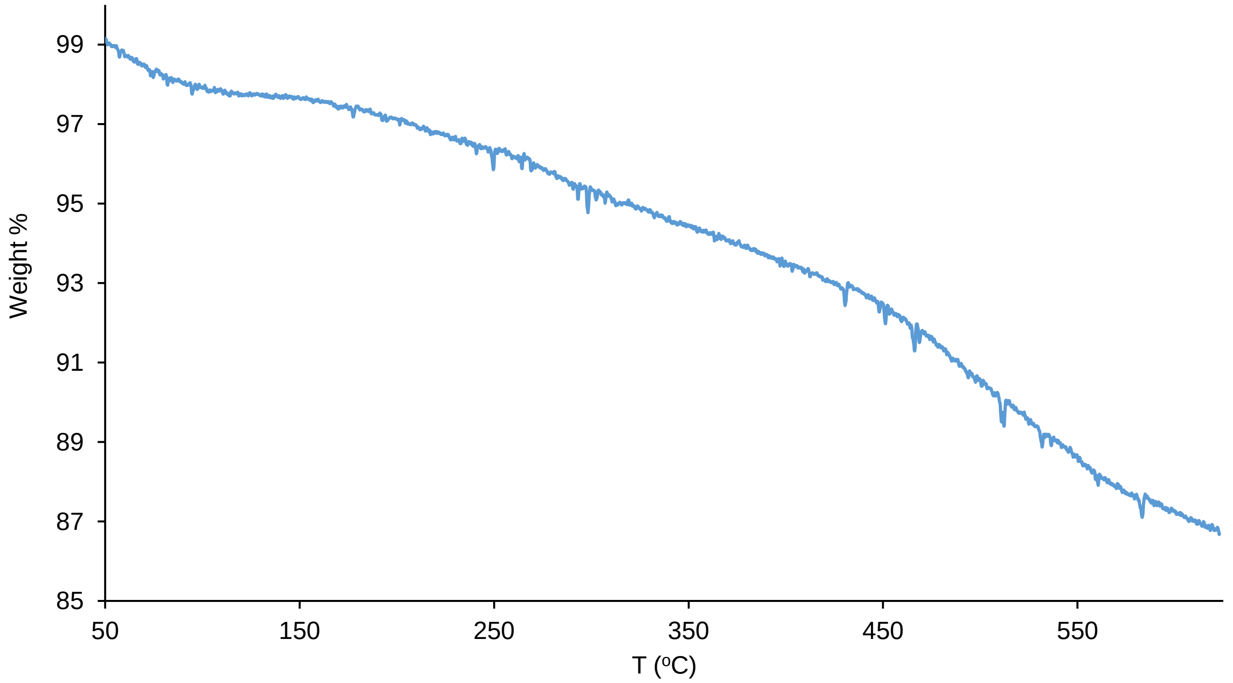

Expansion of region below 150 ºC

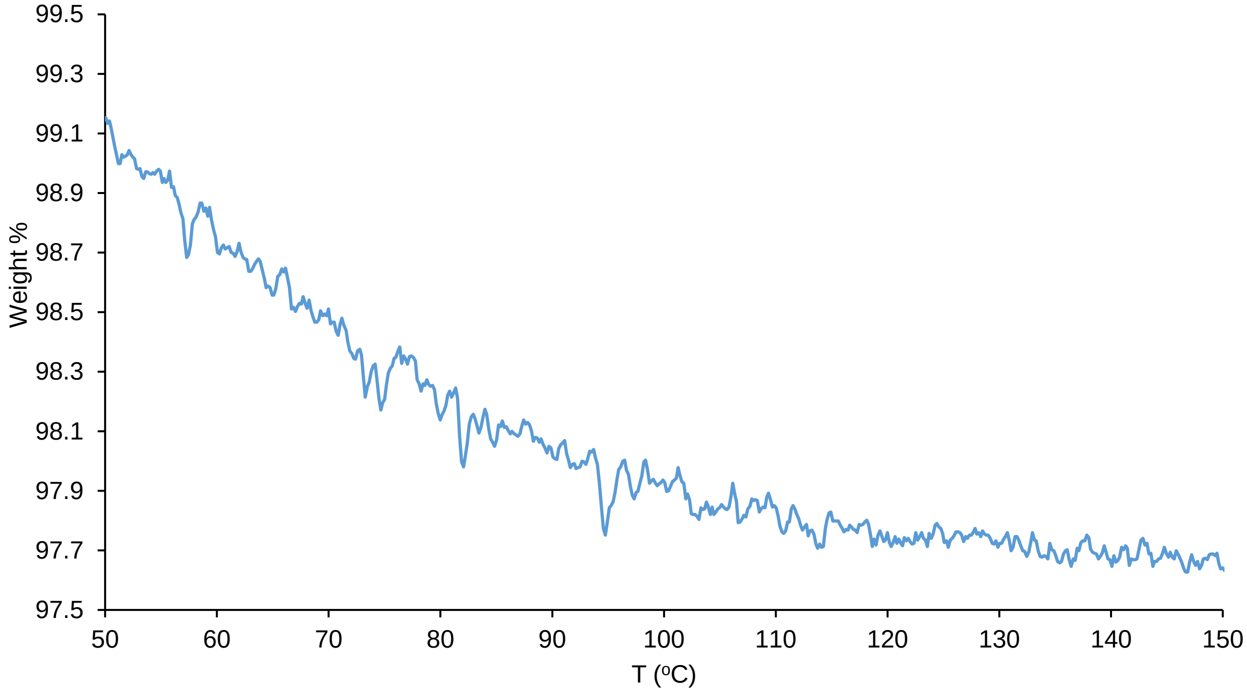

IR spectrum

Full spectrum

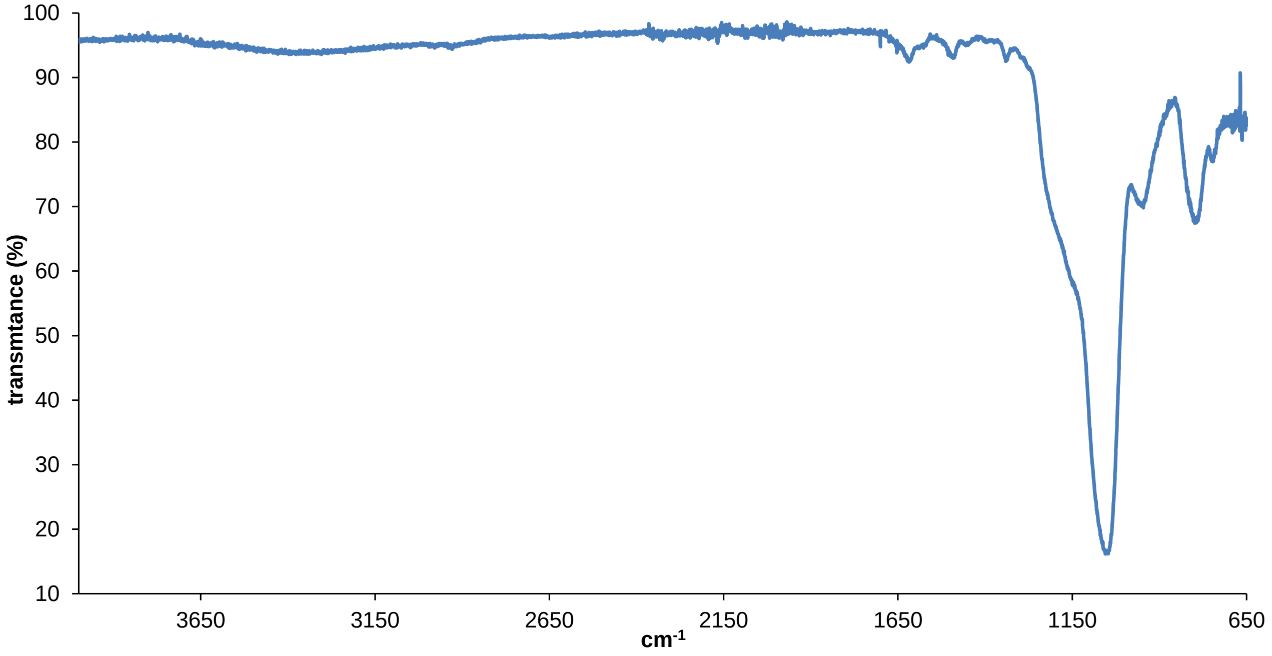

Expansion of 4000-1400 cm^{-1}

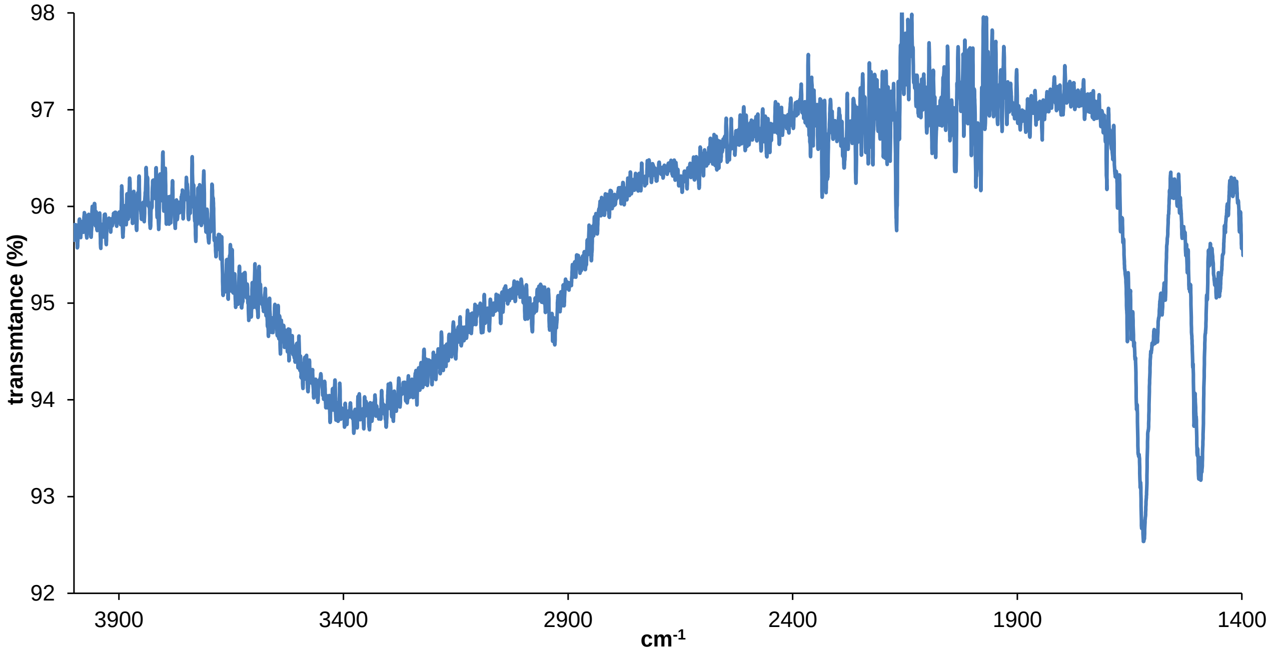

Solid State $^{13}C\{^1H\}$ NMR Spectrum (100 MHz)

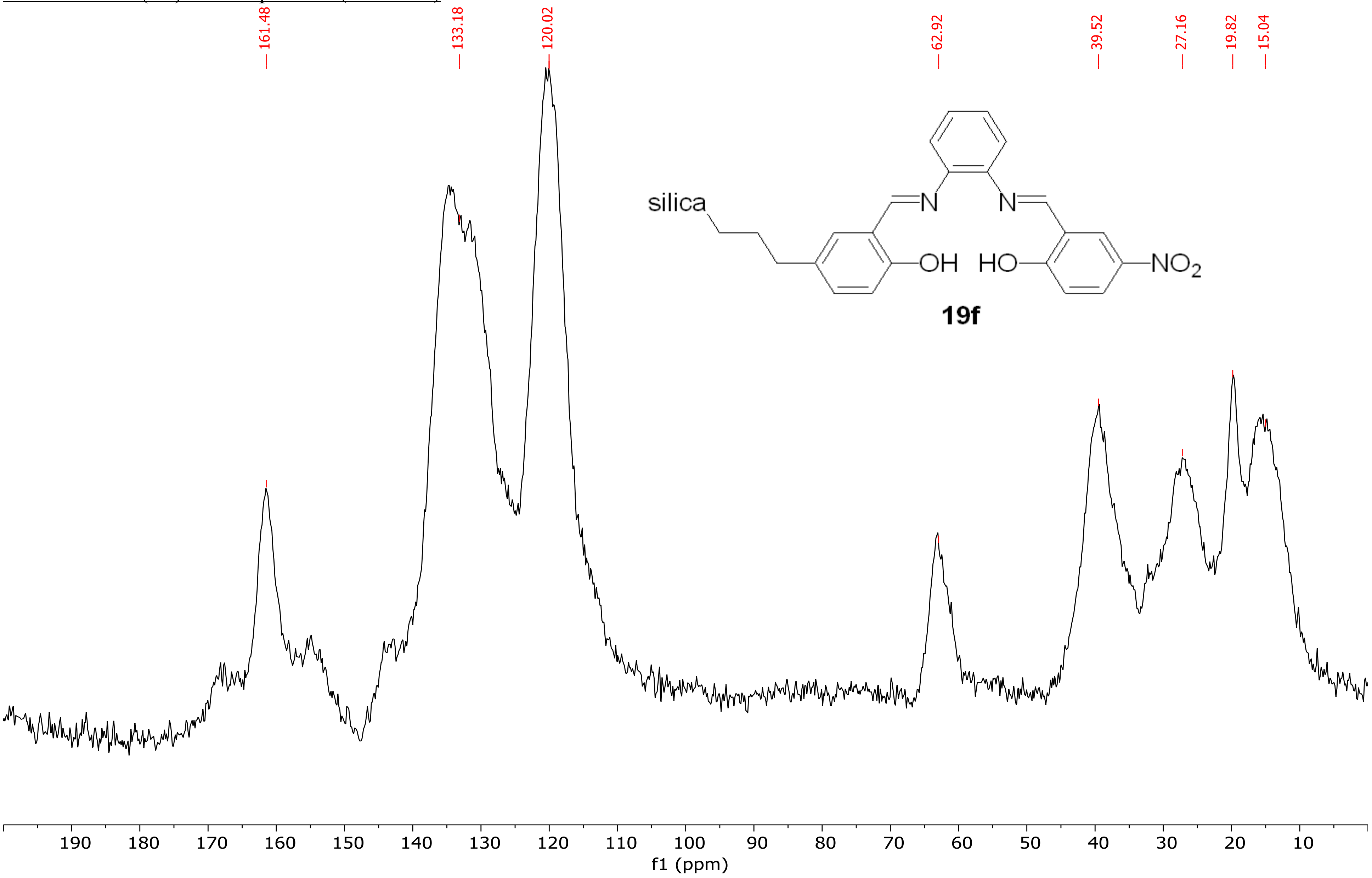

Silica-supported salophen 19g

Analysis

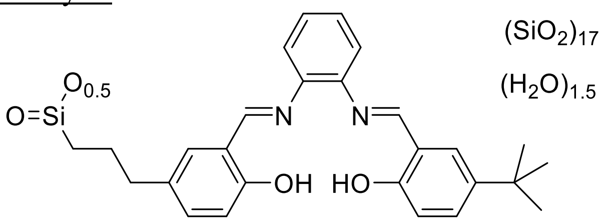

Chemical Formula: $C_{27}H_{32}N_2O_{39}Si_{18}$
Molecular Weight: 1514.06
Elemental Analysis: C, 21.42; H, 2.13%

Found: C, 21.5; H, 2.3%.

Mass of water: 1.5x18 = 27
So predicted %water: = 1.8%
TGA weight loss below 100 ºC: = 2.0%

% yield calculation:
Product should contain
0.63 mmol of salophen unit with RMM of 465.6 = 465.6 x 0.63 mg = 293 mg
10.71 mmol of SiO_2 with RMM of 60 = 60 x 10.71 mg = 642.6 mg
0.95 mmol of H_2O with RMM of 18 = 18 x 0.95 mg = 17 mg
So 100% yield = 953 mg

Actual mass of product = 610 mg
So %yield = 64%

Loading calculation
1.514 g contains 1 mmol of salophen
So loading is: 1 / 1.514 = 0.66 mmol of salophen per gram

Thermogravimetric Analysis

Full trace

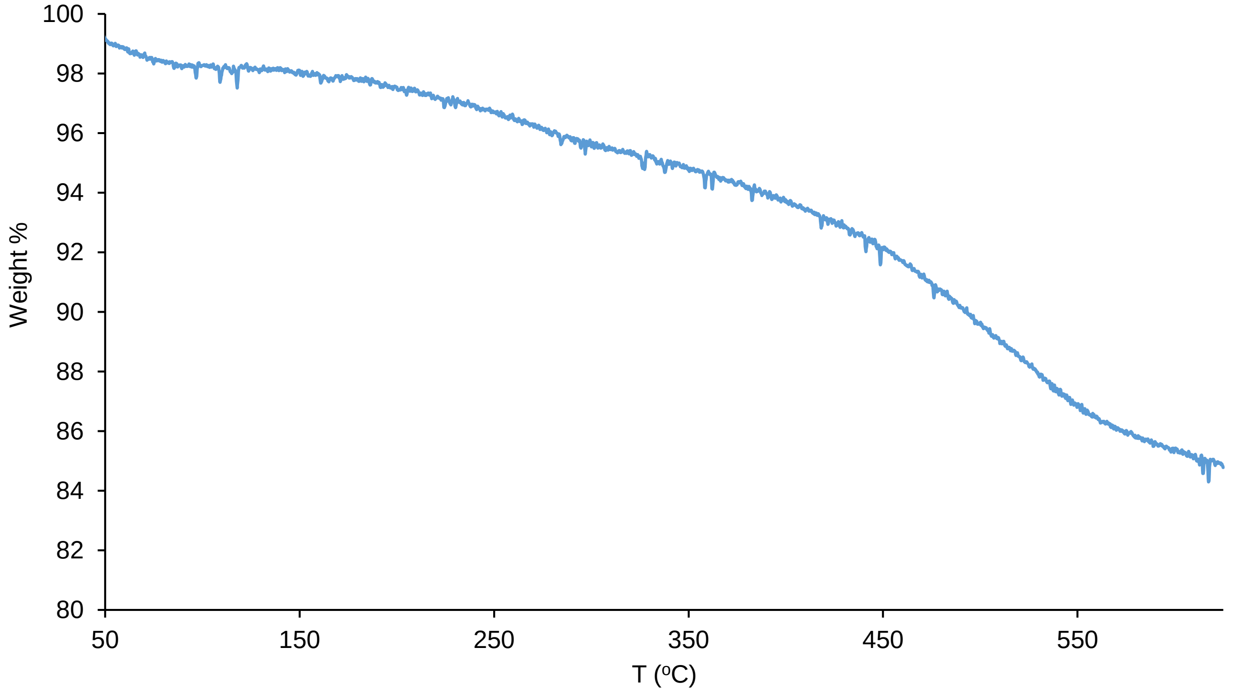

Expansion of region below 150 ºC

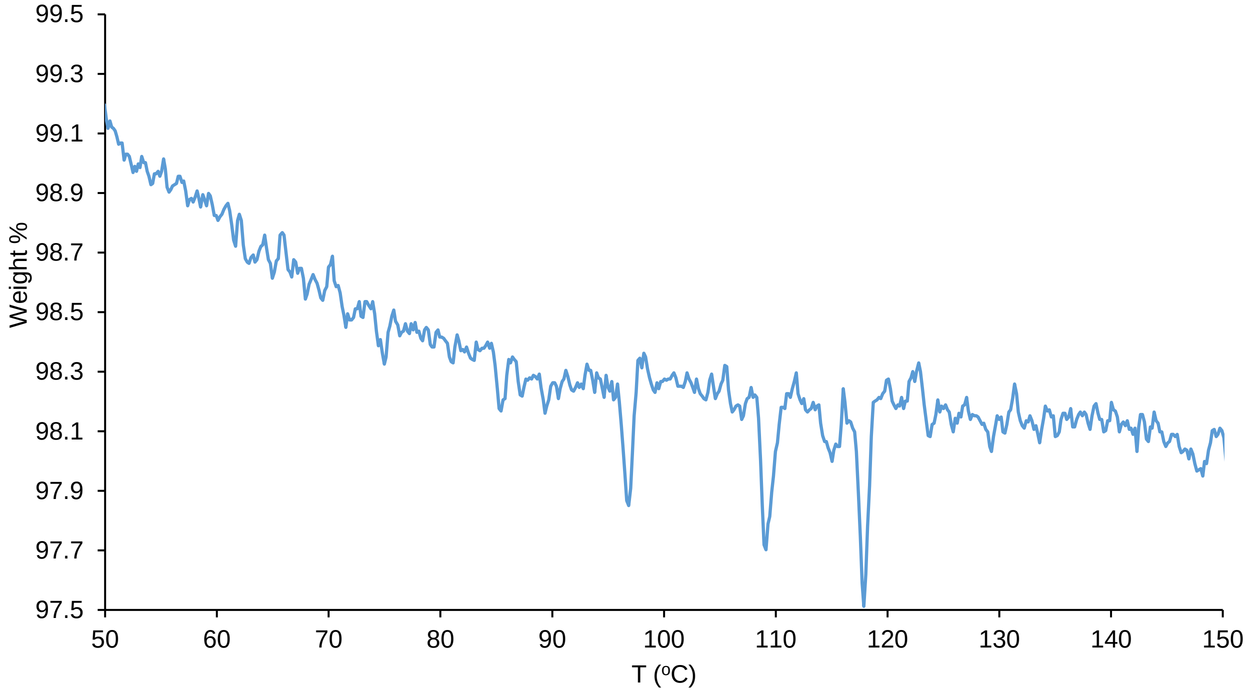

IR spectrum

Full spectrum

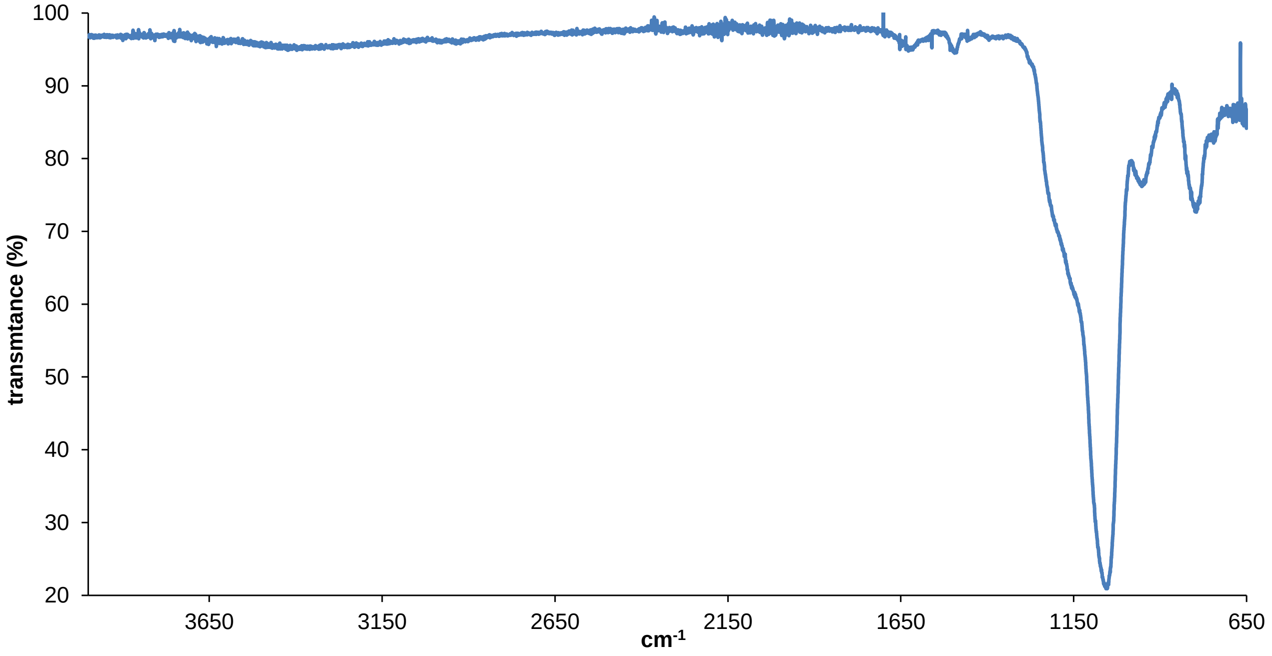

Expansion of 4000-1400 cm^{-1}

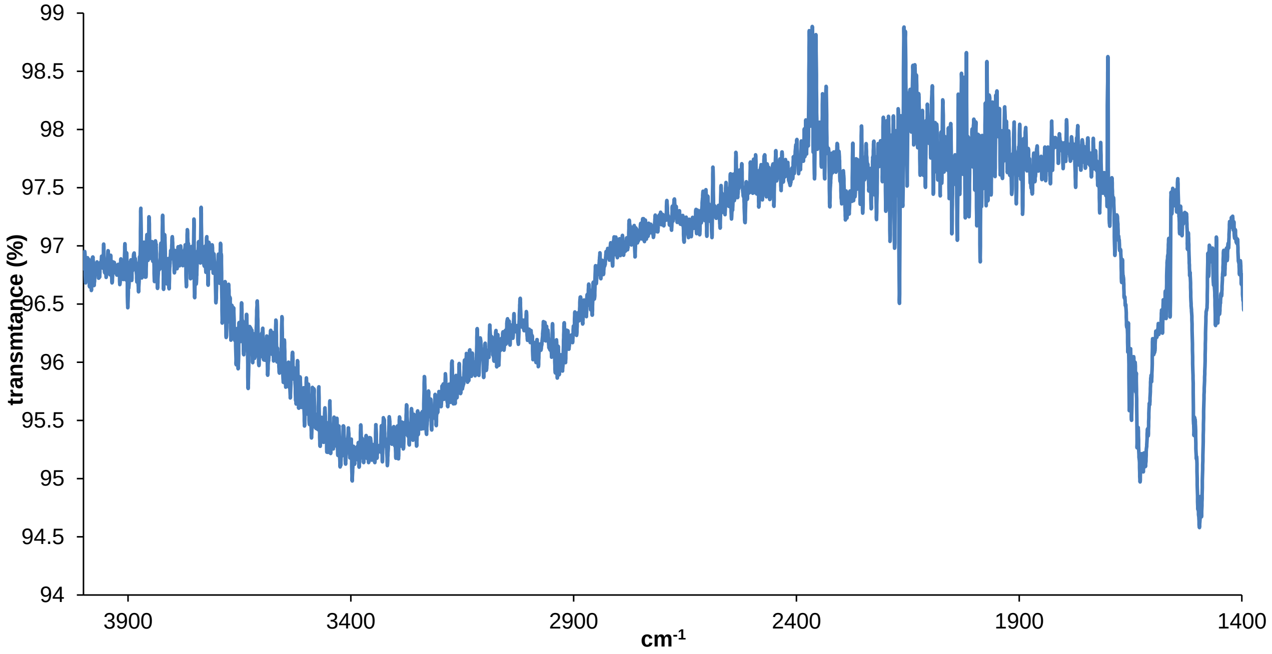

Solid State $^{13}C\{^1H\}$ NMR Spectrum (100 MHz)

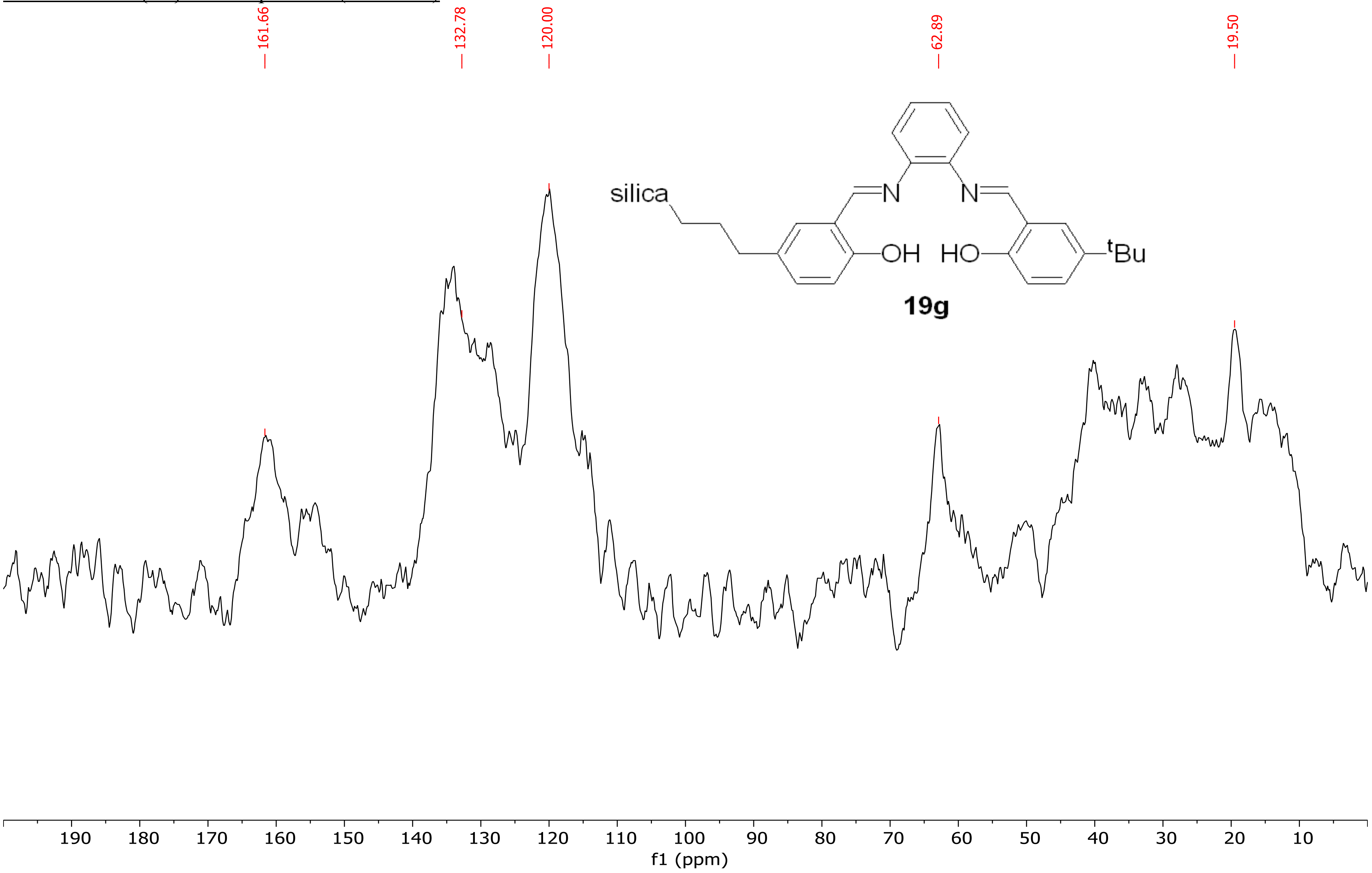

3-Phenoxypropylene carbonate (21a)

IR spectrum

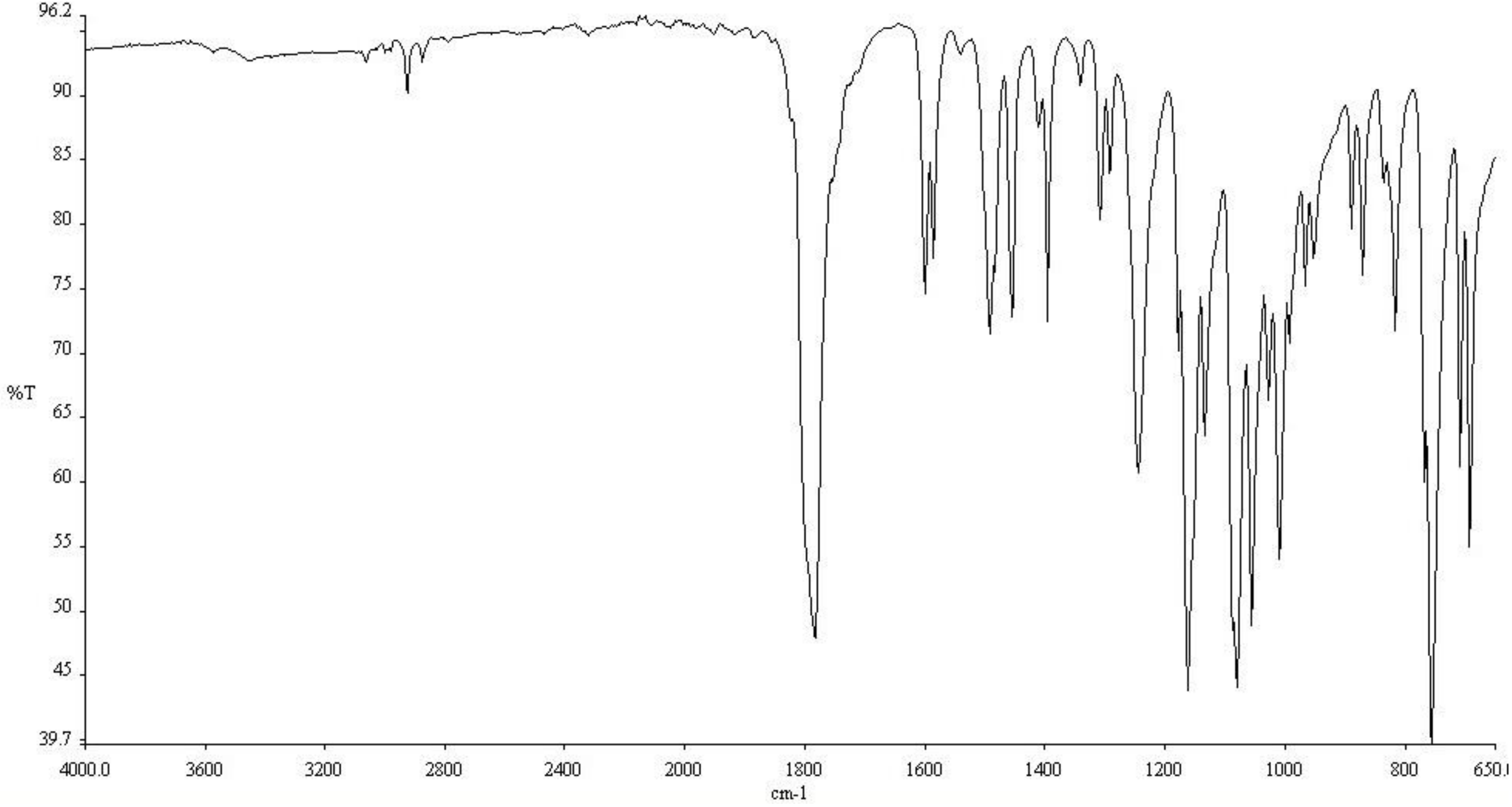

^{1}H NMR Spectrum (300 MHz, $CDCl_3$)

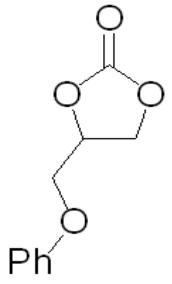

21a

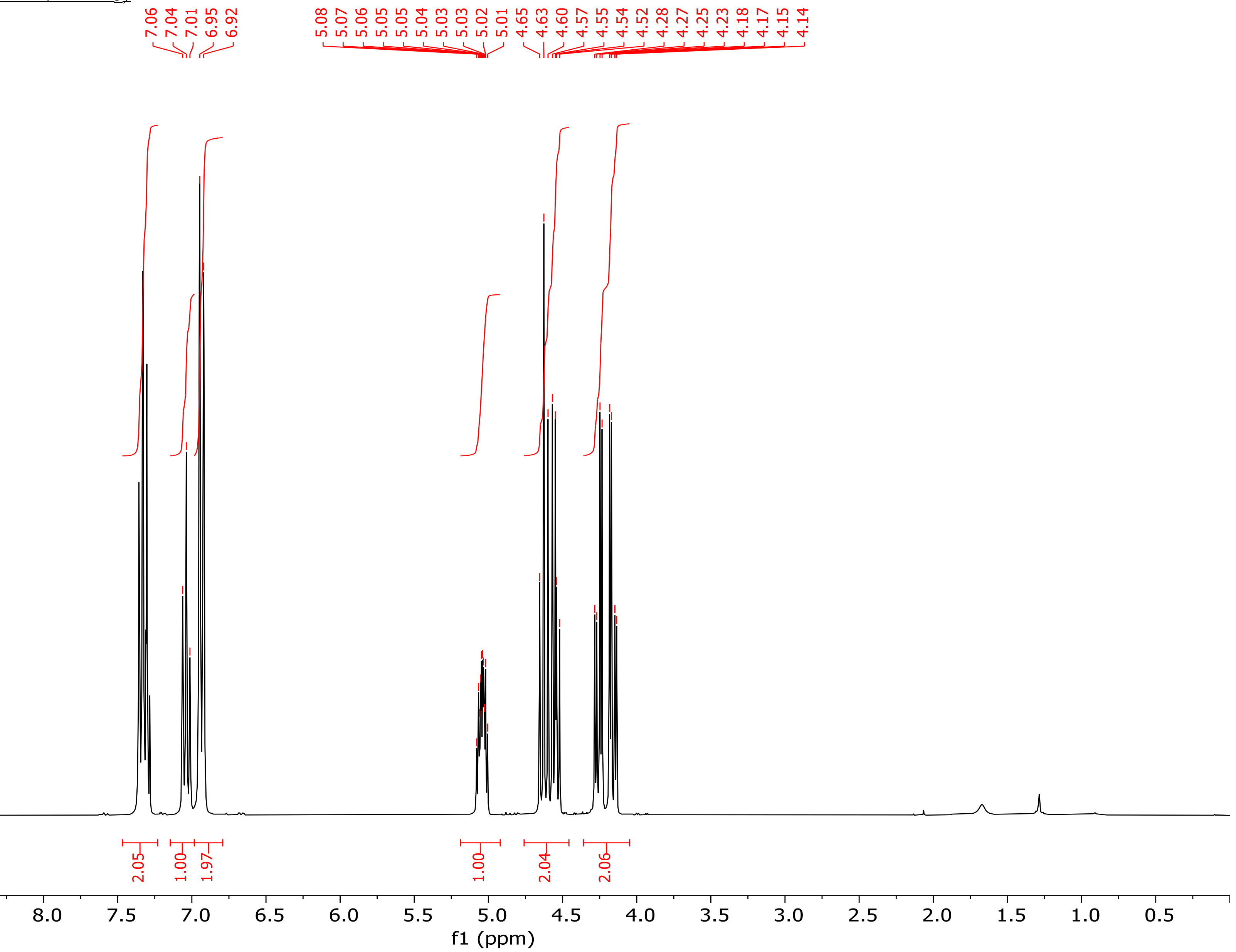

$^{13}C\{^{1}H\}$ NMR Spectrum (75 MHz, $CDCl_3$)

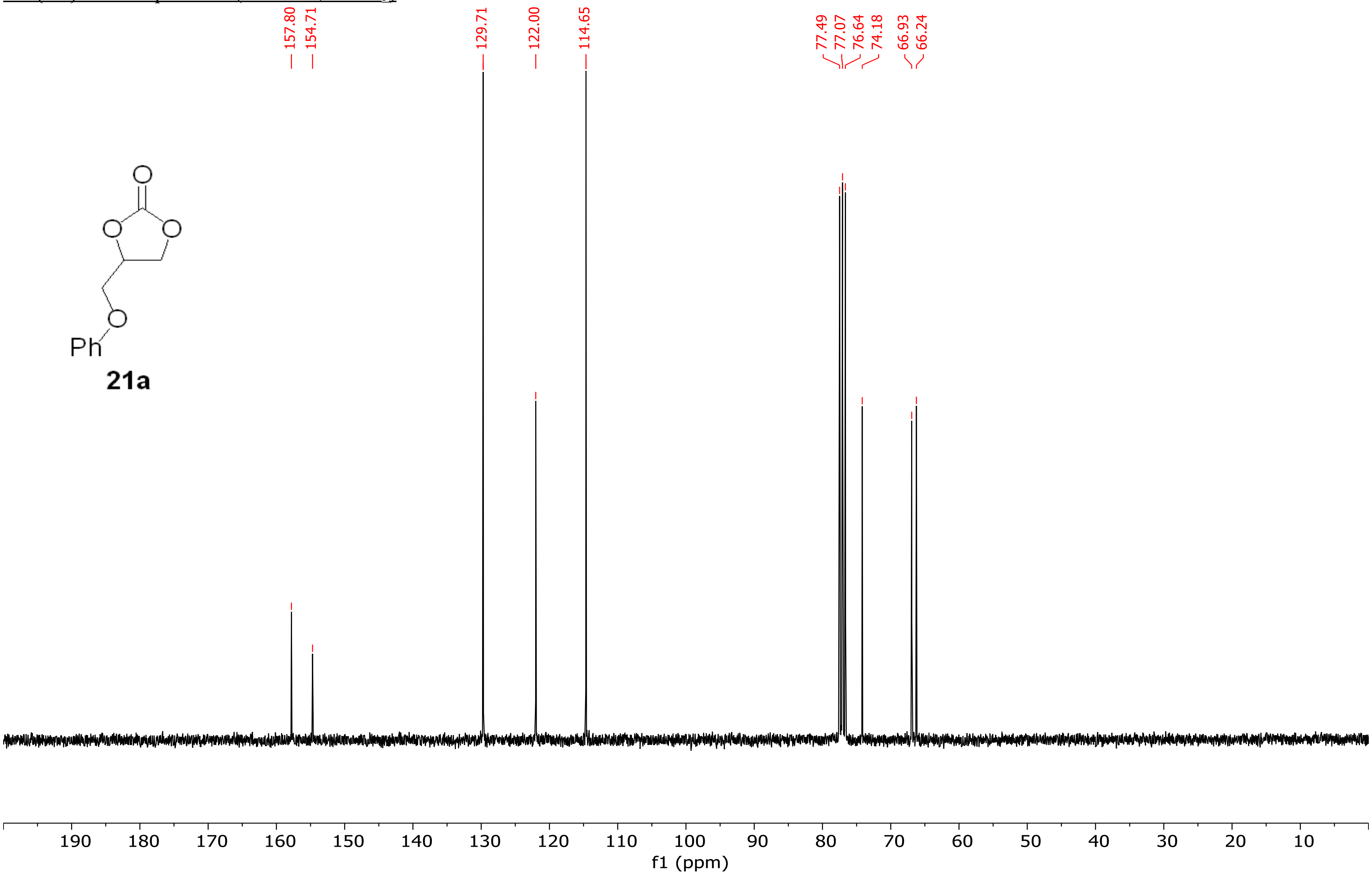

Styrene carbonate (21b)

IR spectrum

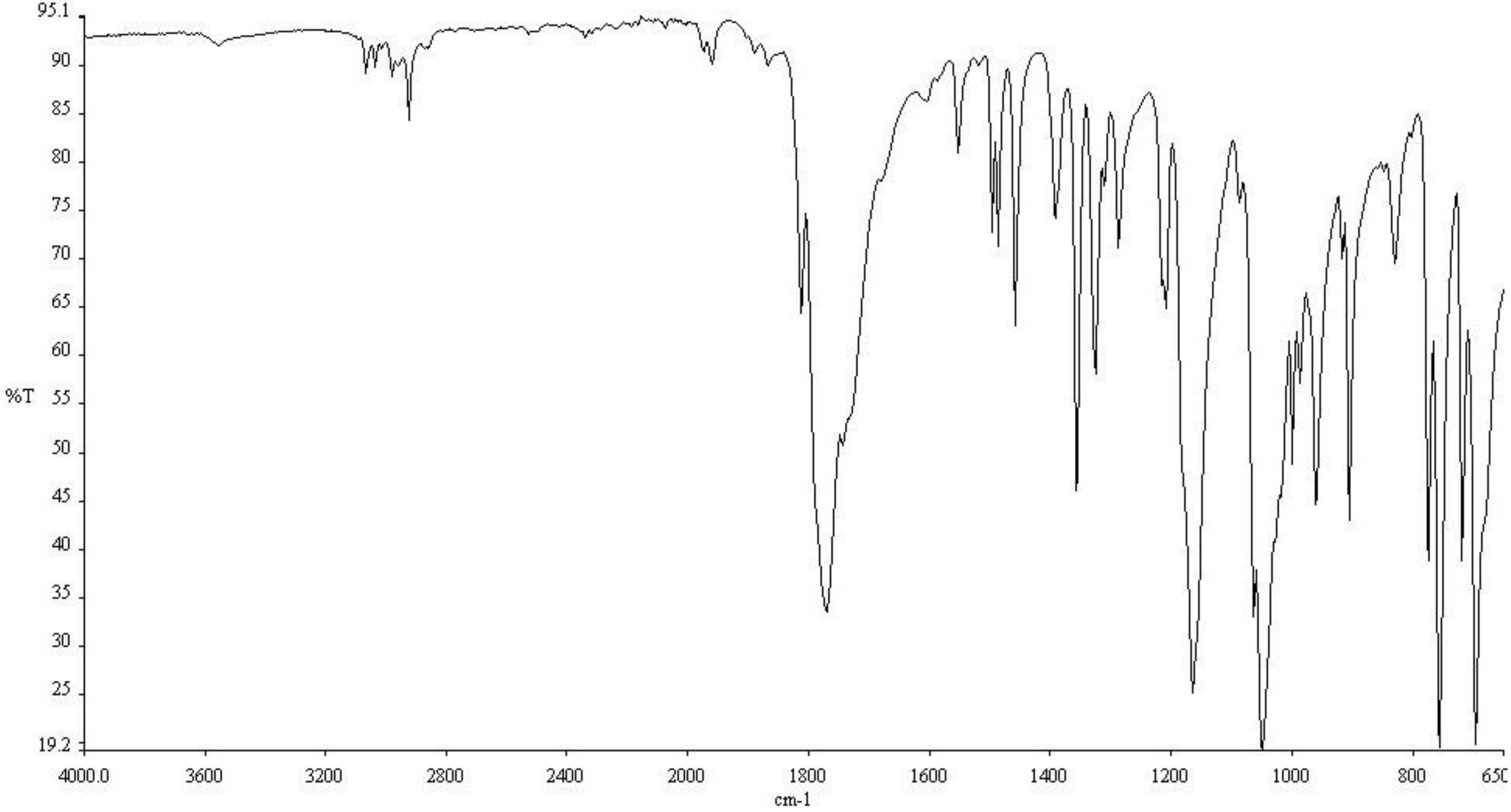

^{1}H NMR Spectrum (300 MHz, $CDCl_3$)

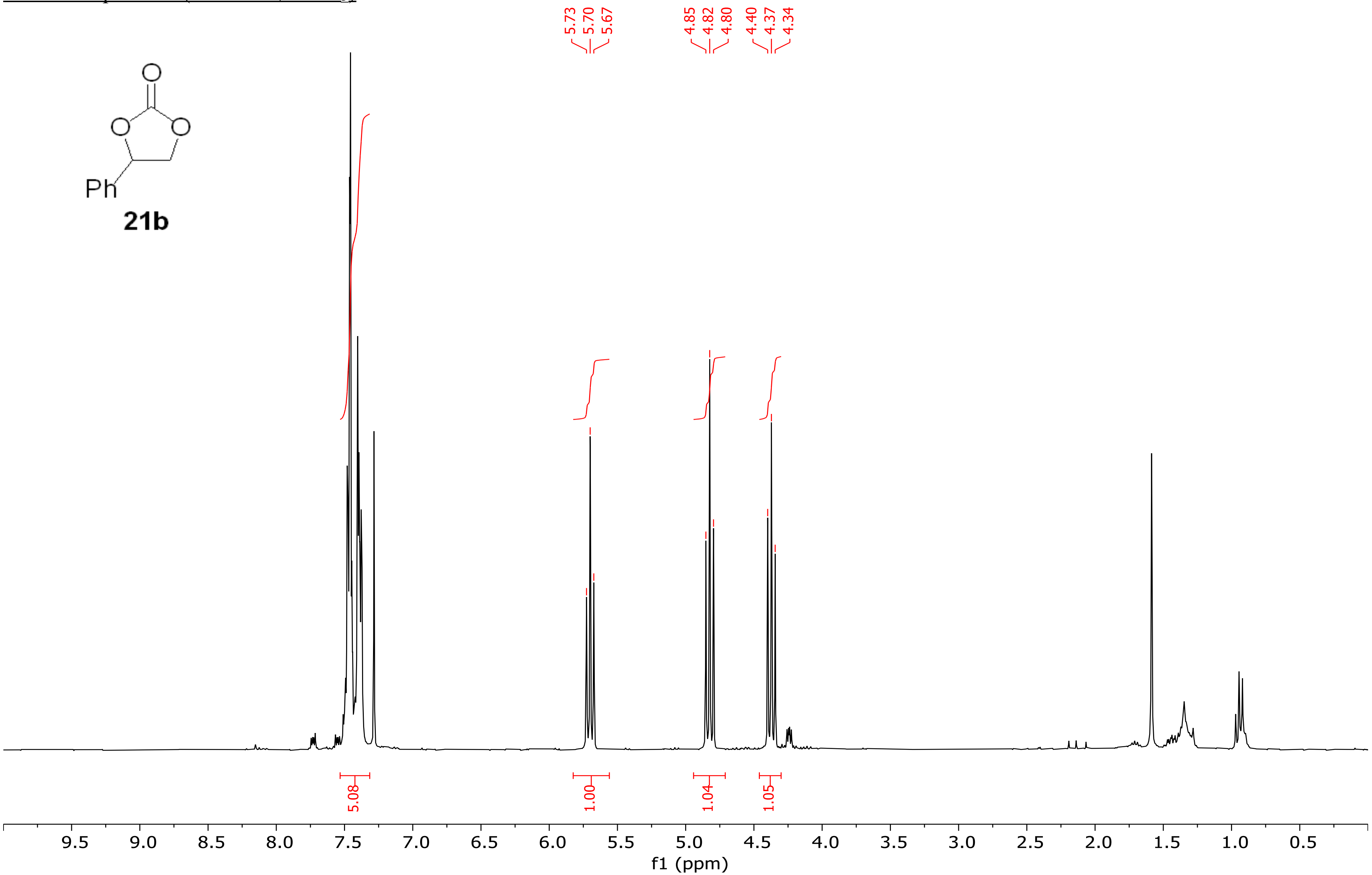

$^{13}C\{^{1}H\}$ NMR Spectrum (75 MHz, $CDCl_3$)

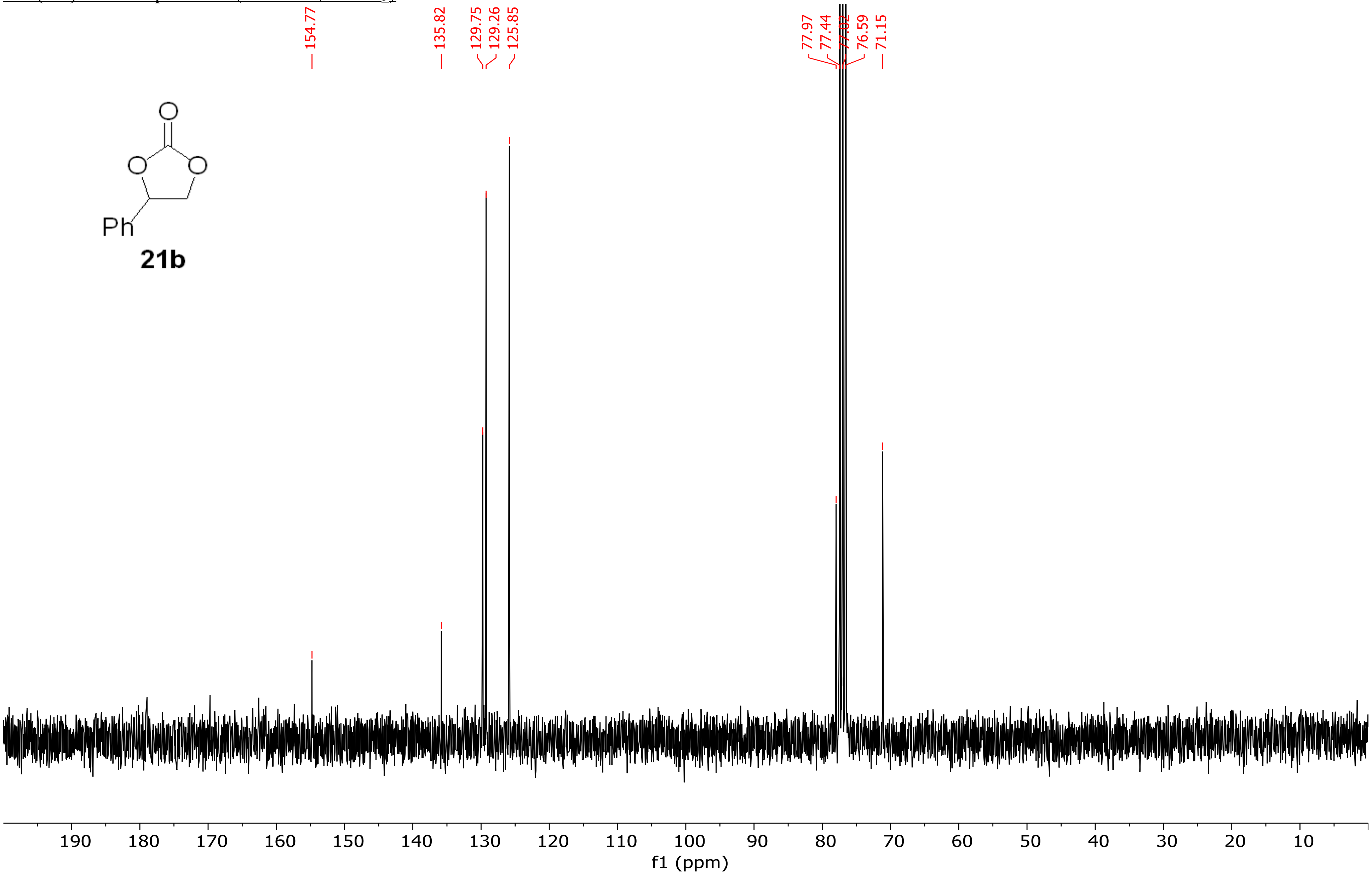

4-Chlorostyrene carbonate (21c)

IR spectrum

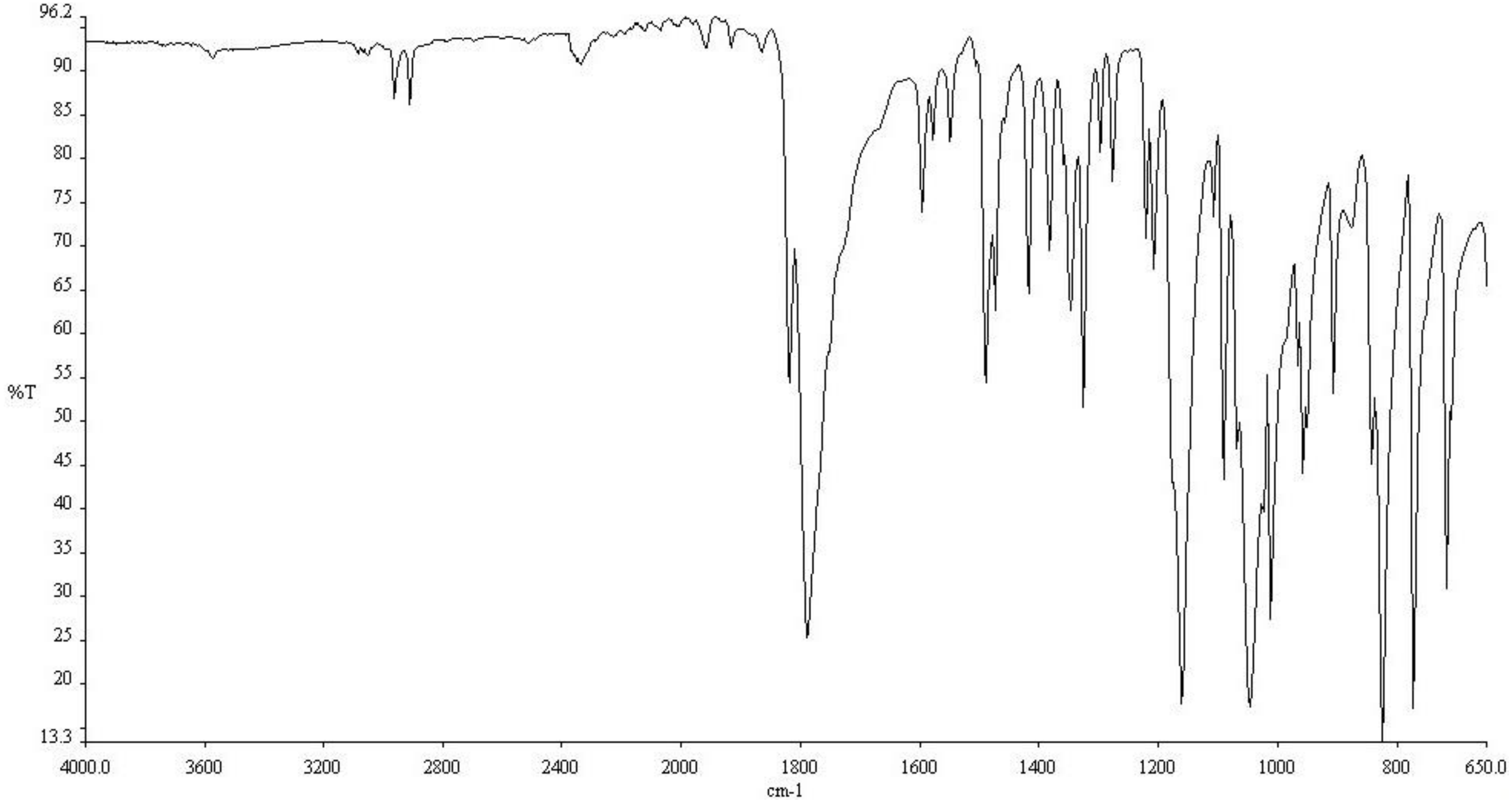

<u>^{1}H NMR Spectrum (300 MHz, $CDCl_3$)</u>

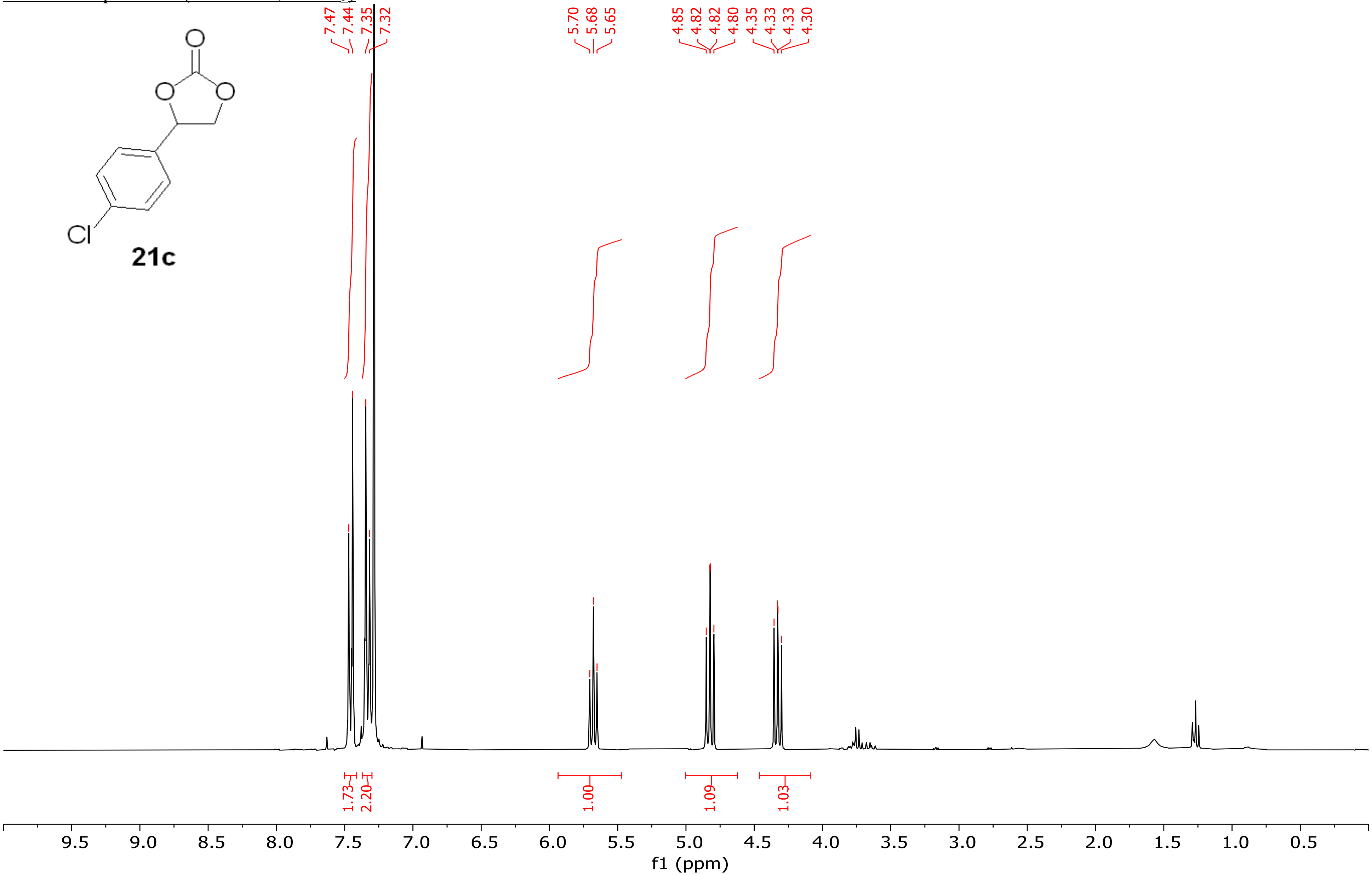

$^{13}C\{^{1}H\}$ NMR Spectrum (75 MHz, $CDCl_3$)

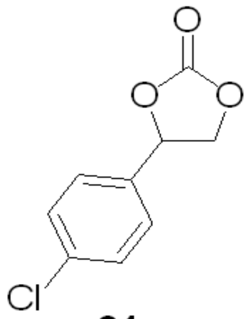

21c

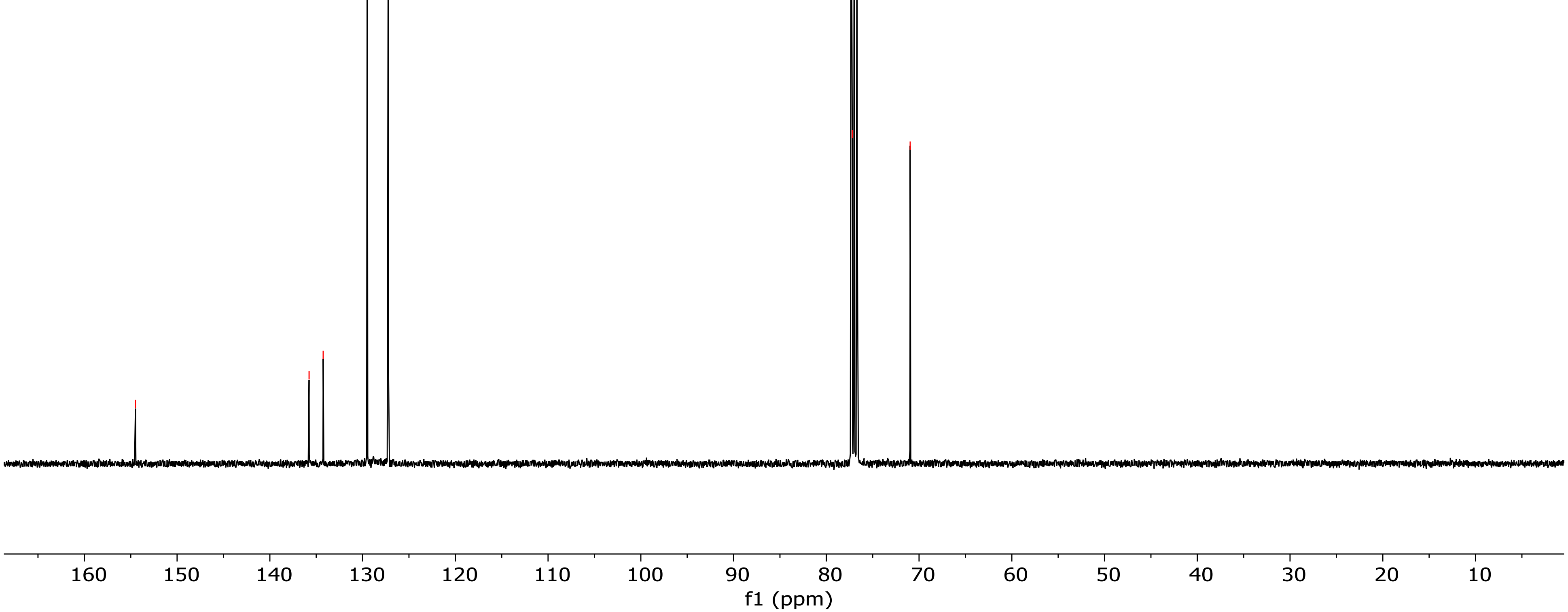

Dec-1-ene carbonate (21d)

IR spectrum

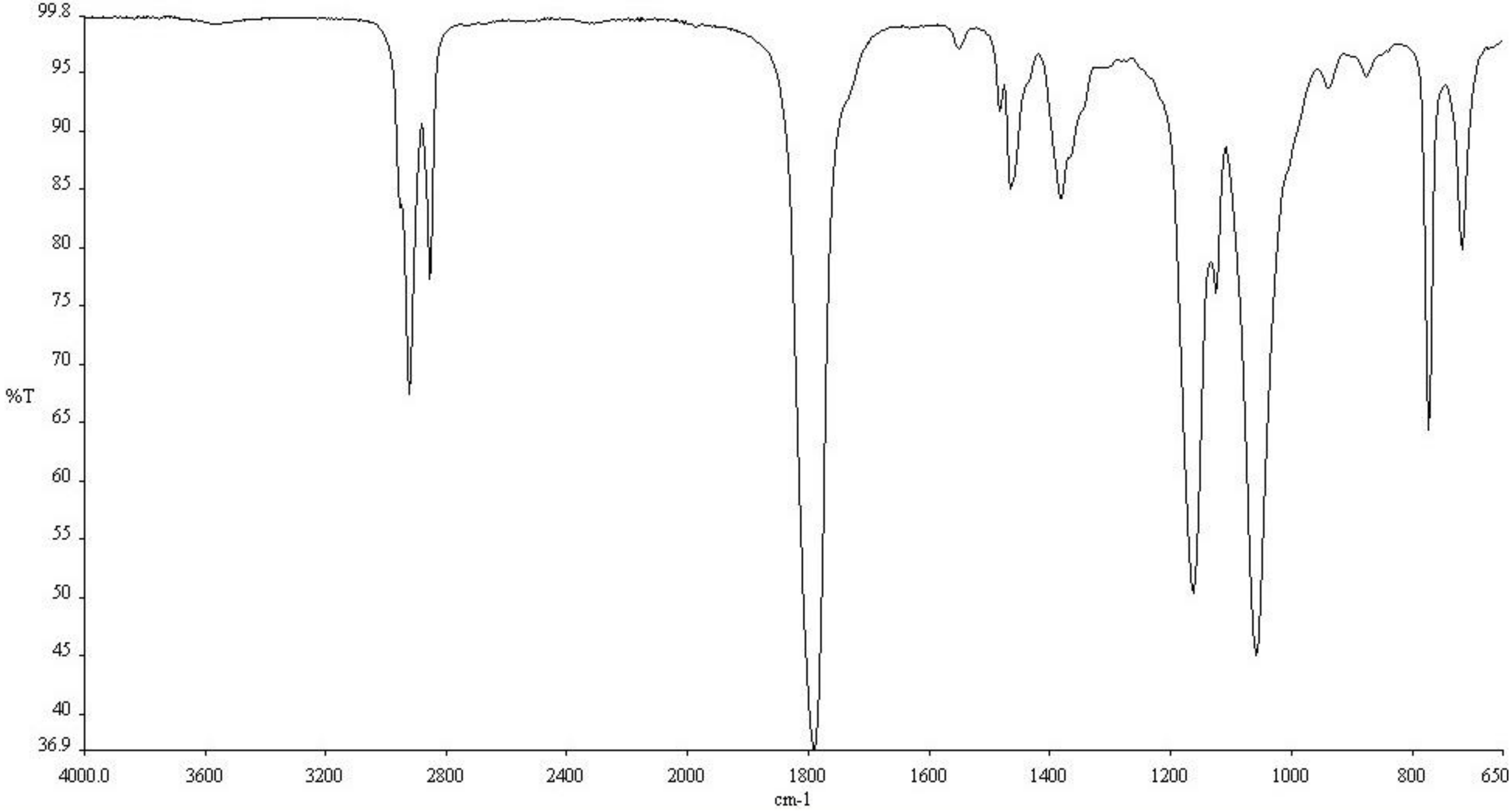

^{1}H NMR Spectrum (300 MHz, $CDCl_3$)

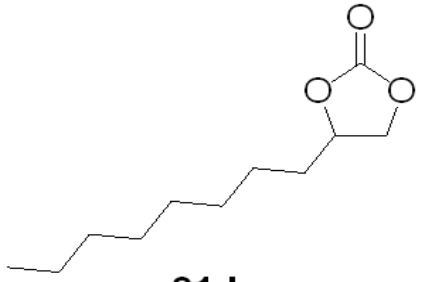

21d

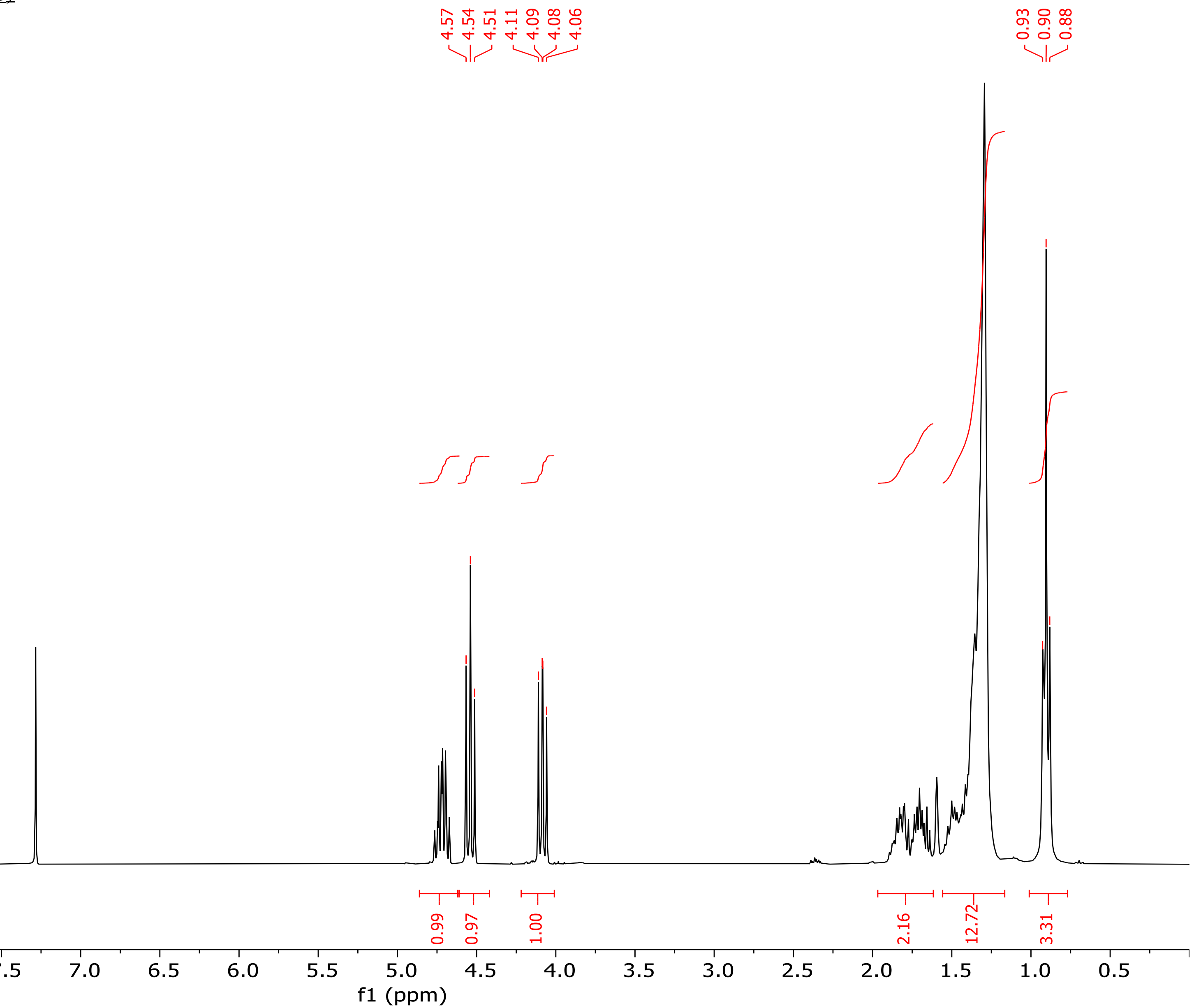

$^{13}C\{^{1}H\}$ NMR Spectrum (75 MHz, $CDCl_3$)

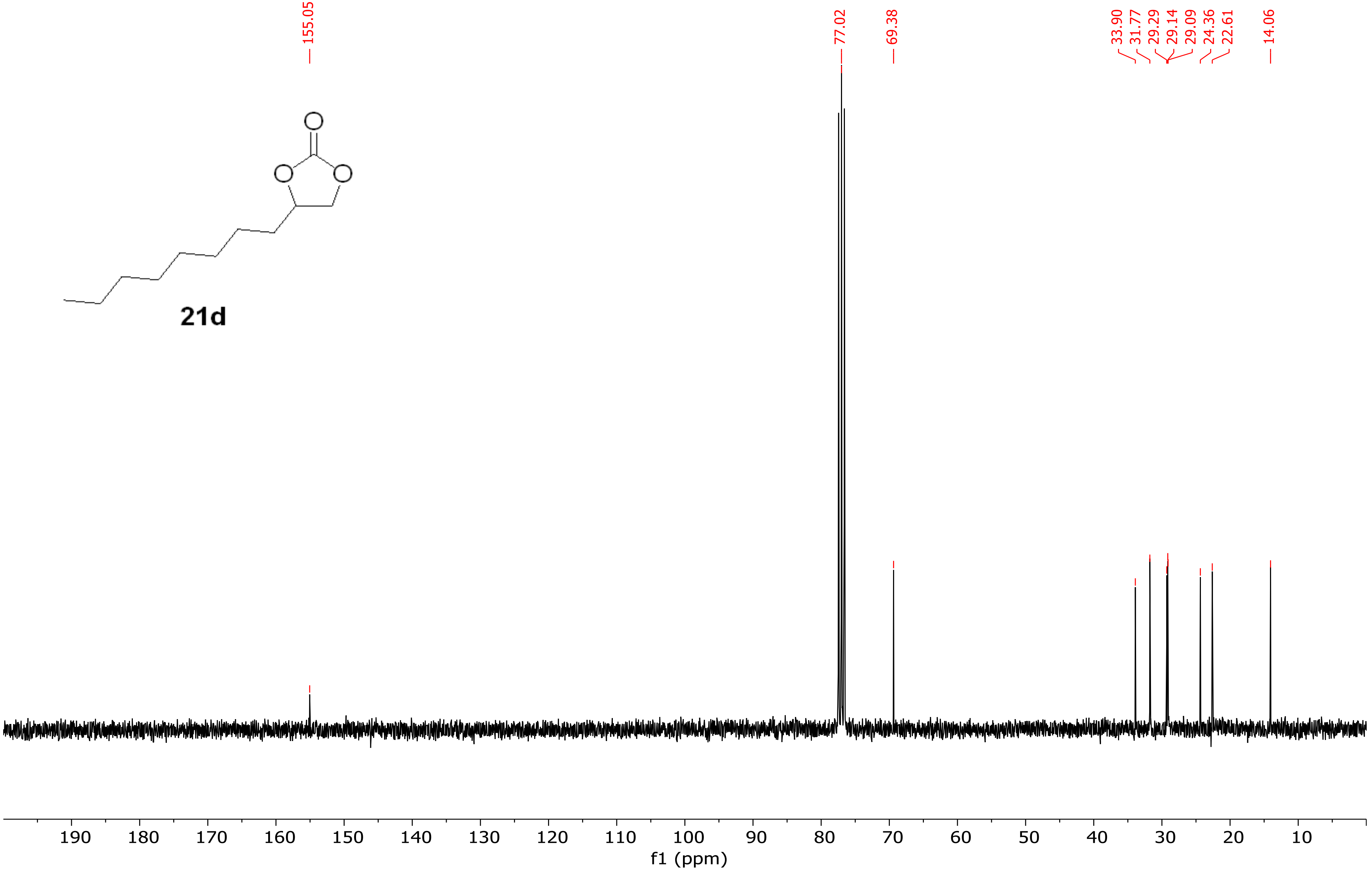

Dodec-1-ene carbonate (21e)

<u>IR spectrum</u>

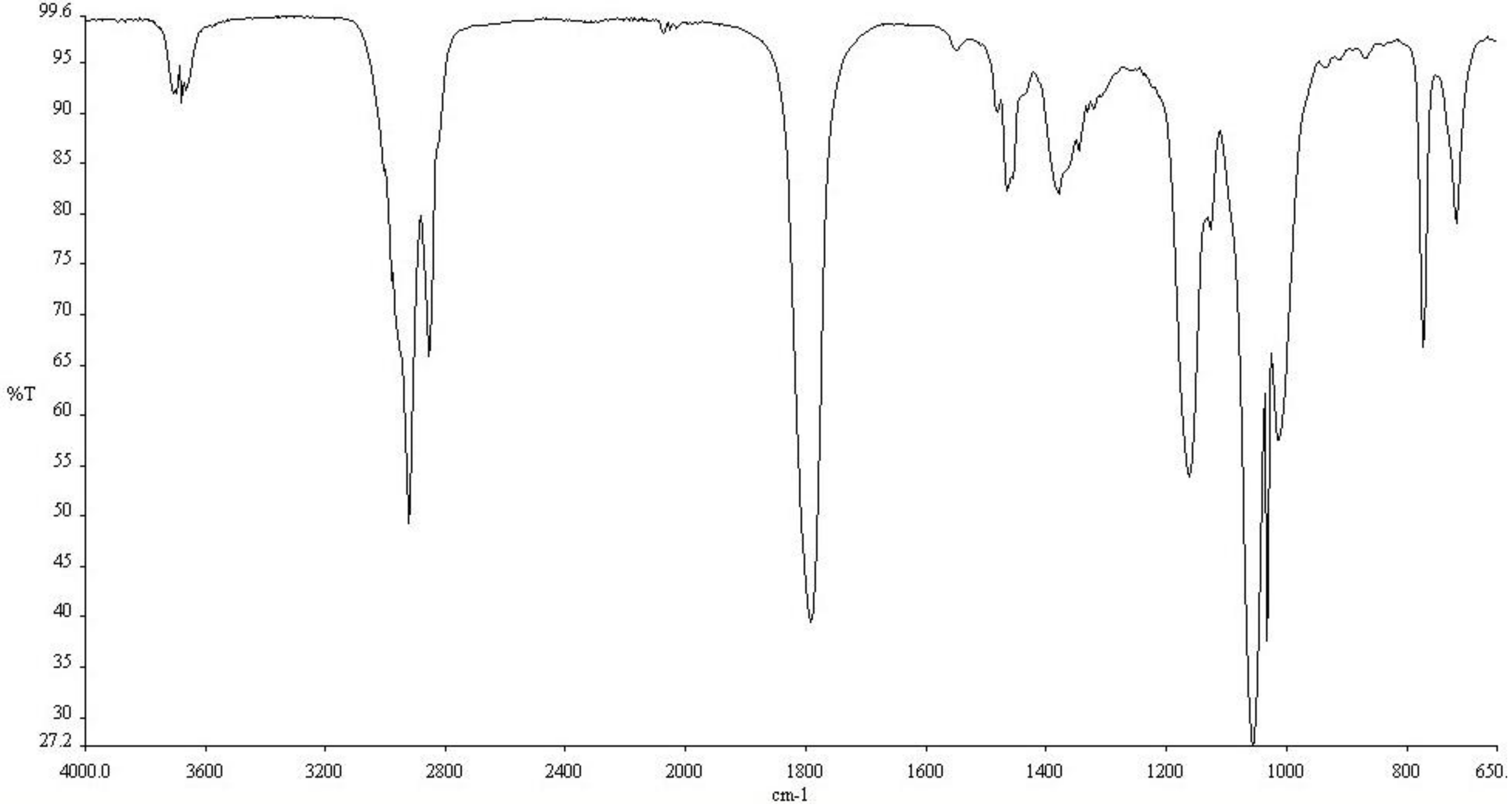

^{1}H NMR Spectrum (300 MHz, $CDCl_3$)

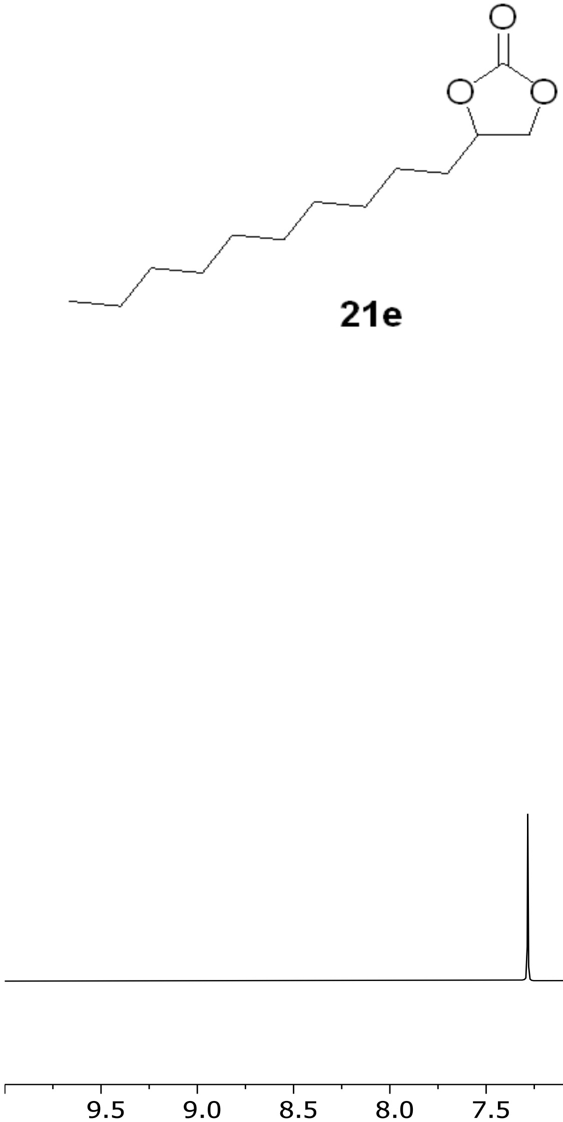

21e

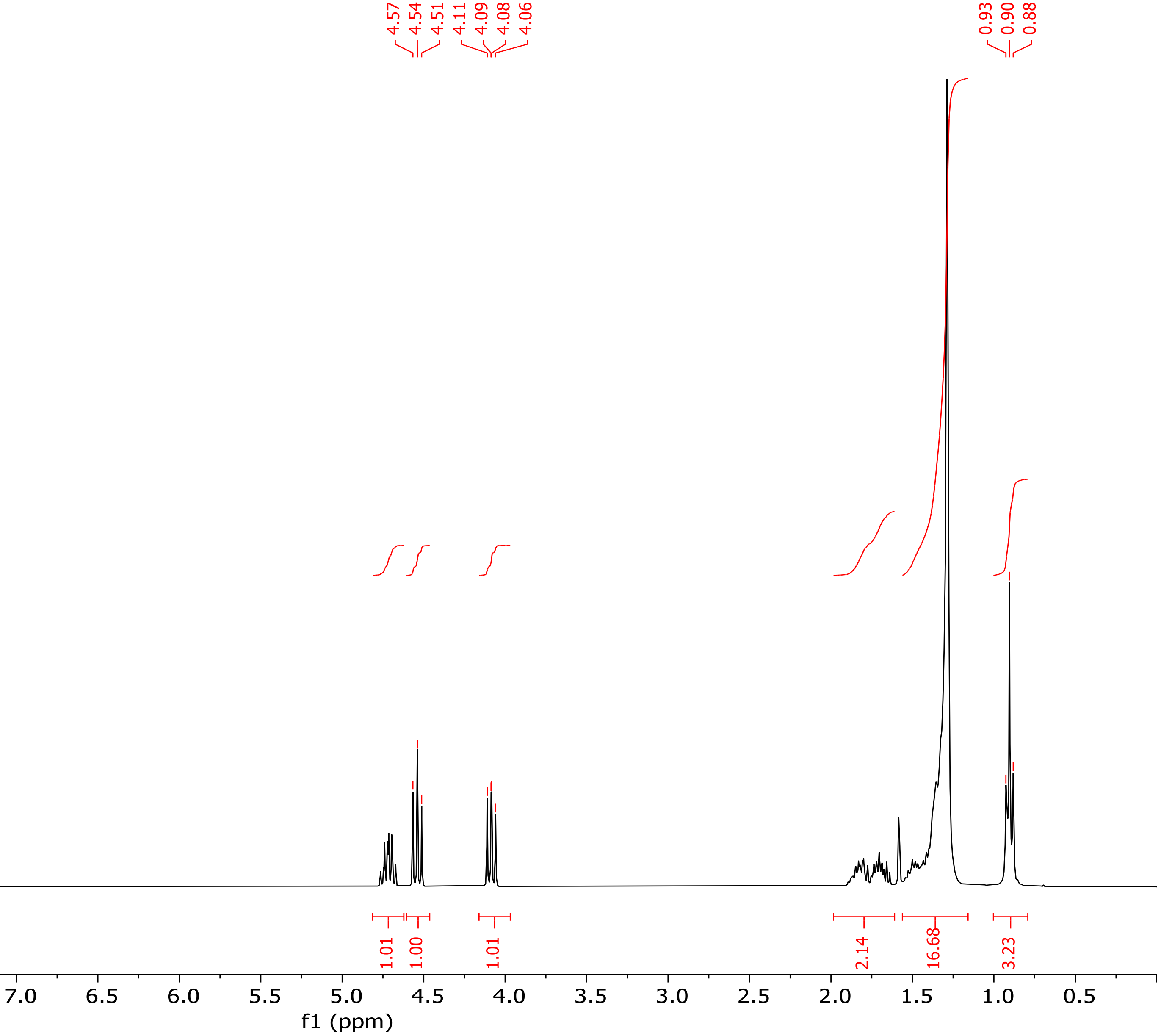

$^{13}C\{^{1}H\}$ NMR Spectrum (75 MHz, $CDCl_3$)

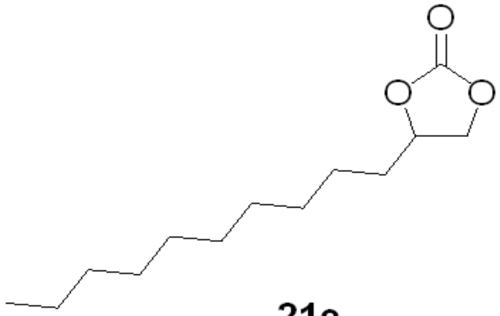

21e

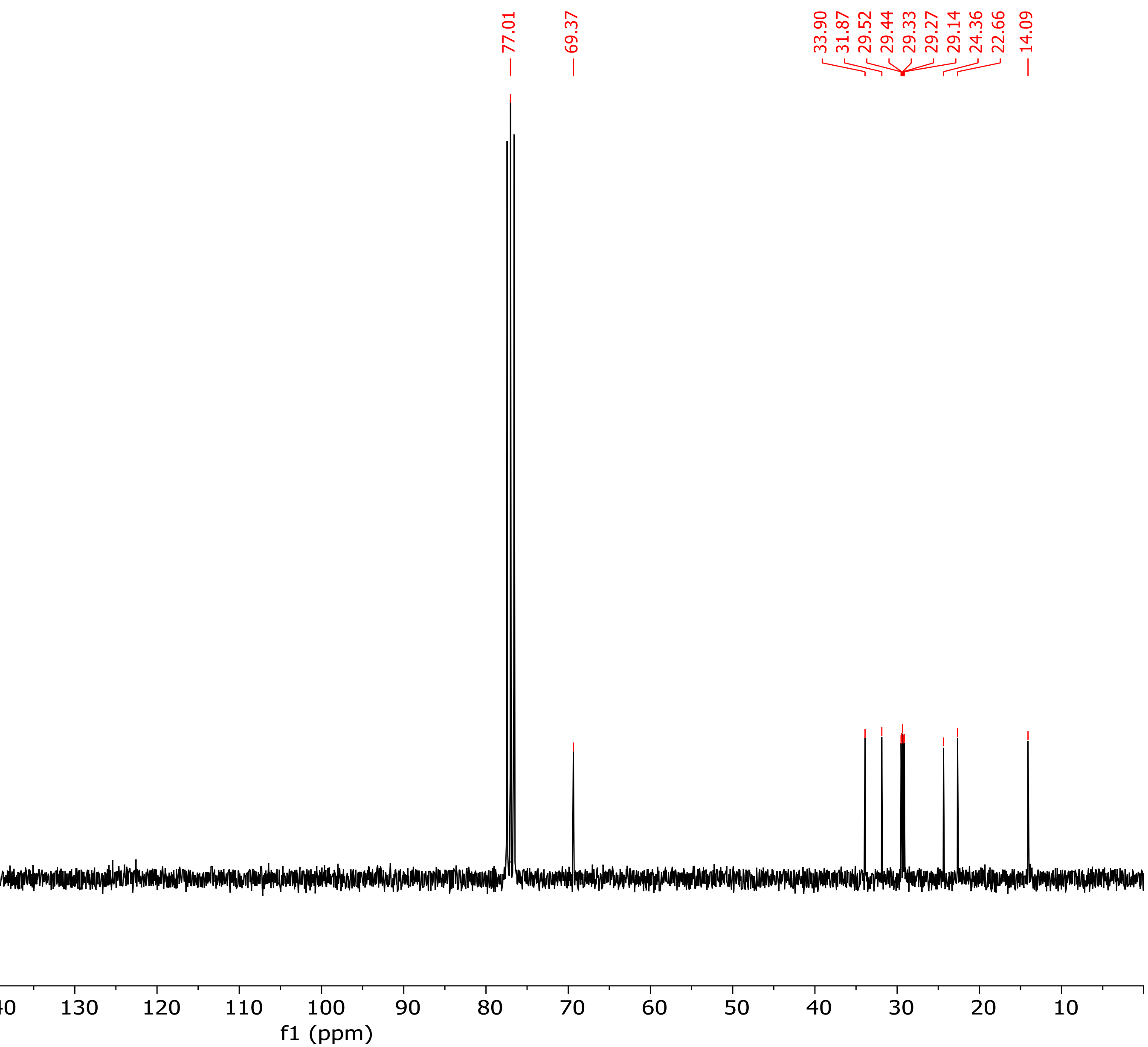

3-Bromopropylene carbonate (21f)

IR spectrum

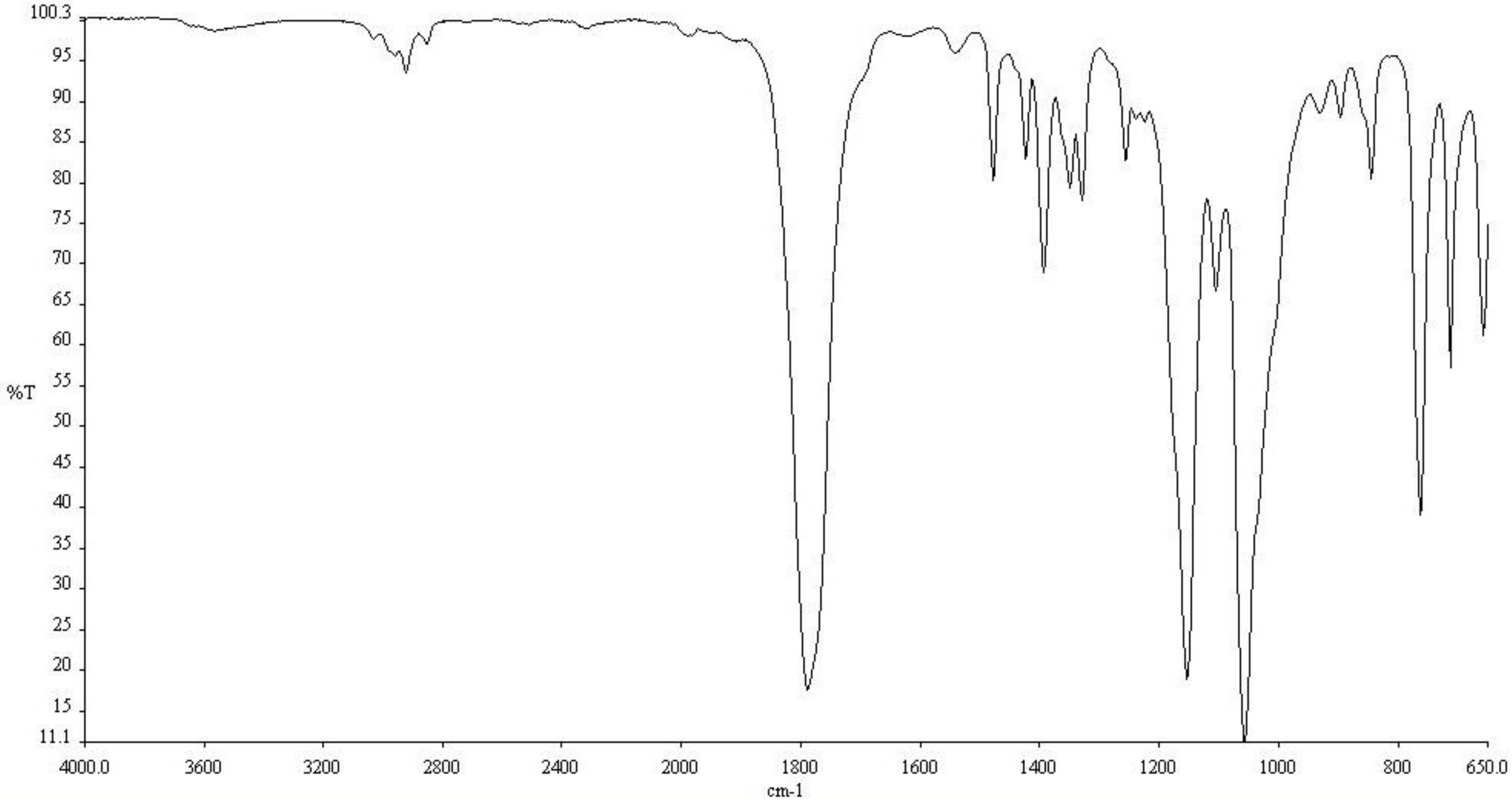

^{1}H NMR Spectrum (300 MHz, $CDCl_3$)

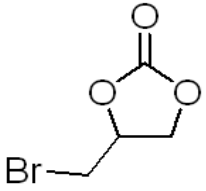

21f

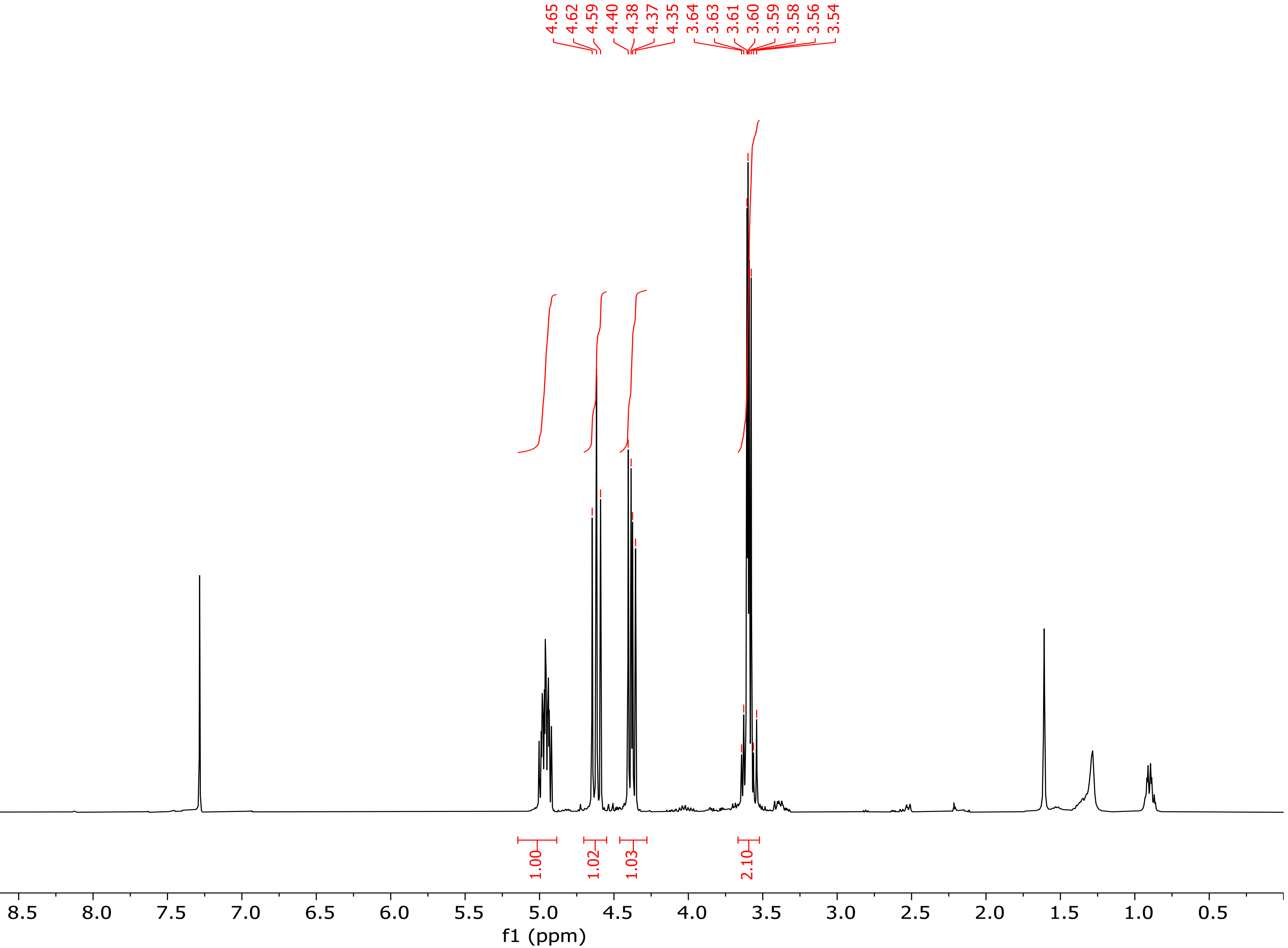

$^{13}C\{^{1}H\}$ NMR Spectrum (75 MHz, $CDCl_3$)

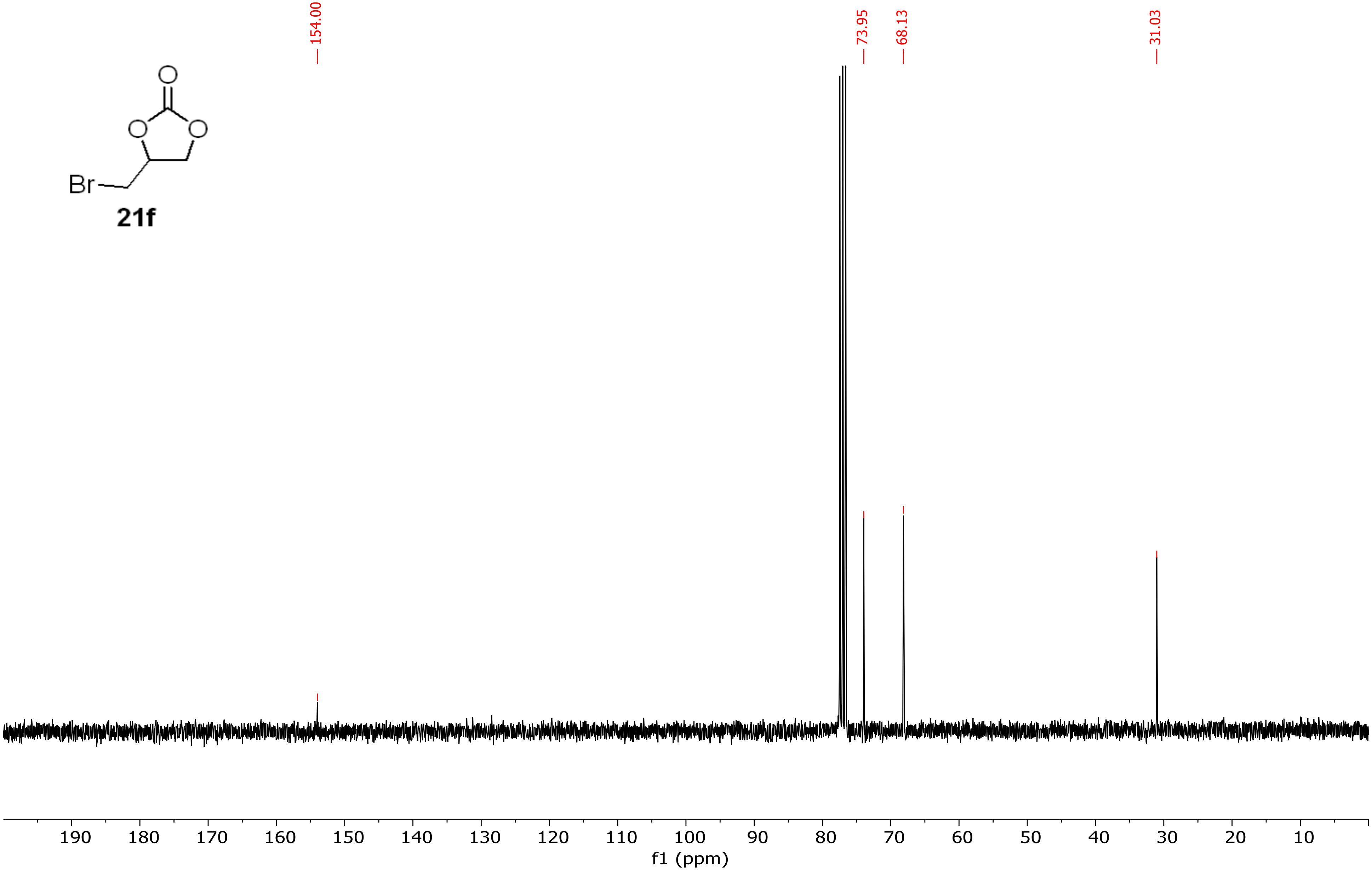

21f

3-Chloropropylene carbonate (21g)

IR spectrum

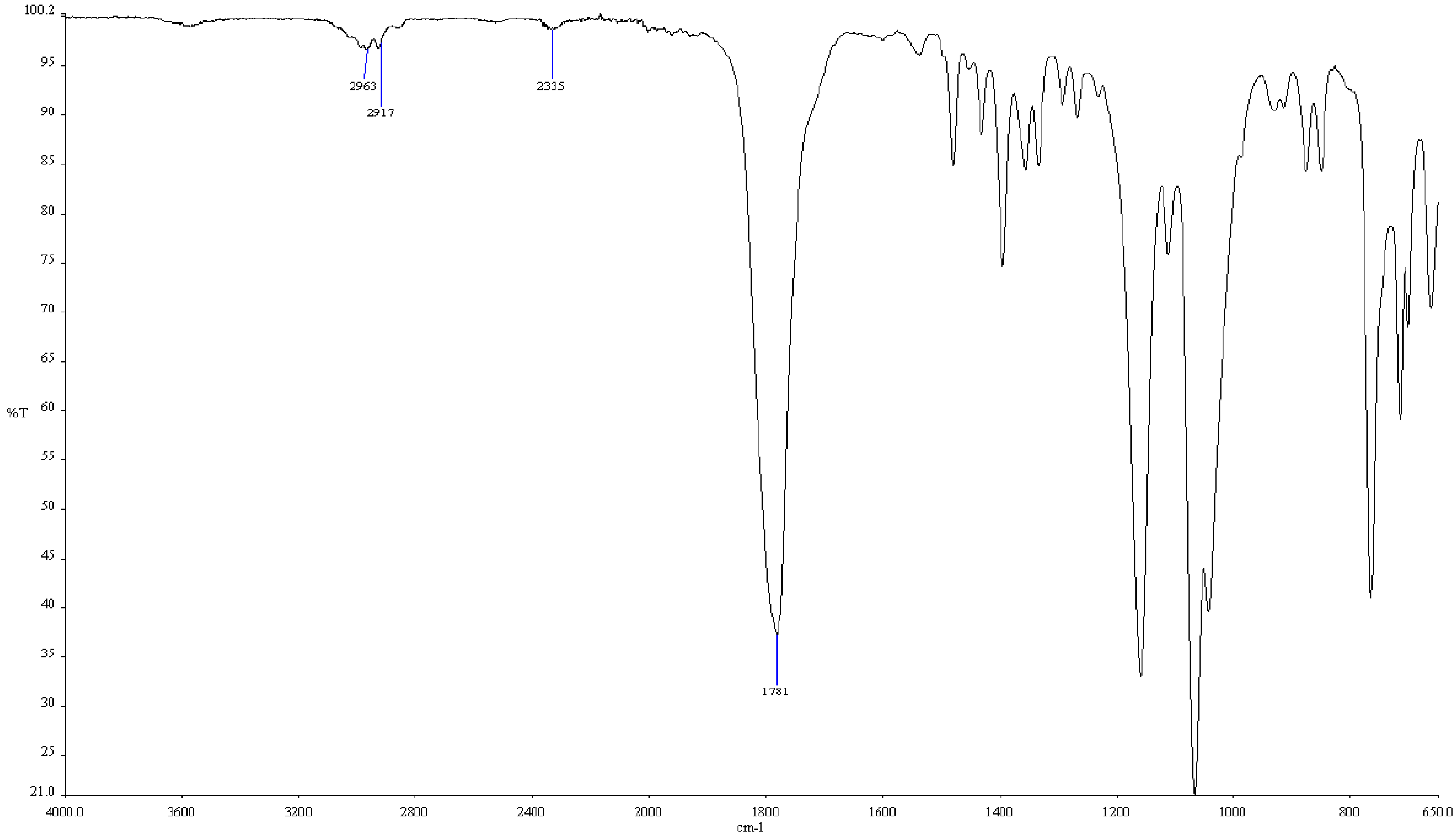

^{1}H NMR Spectrum (400 MHz, $CDCl_3$)

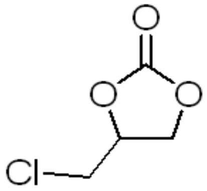

21g

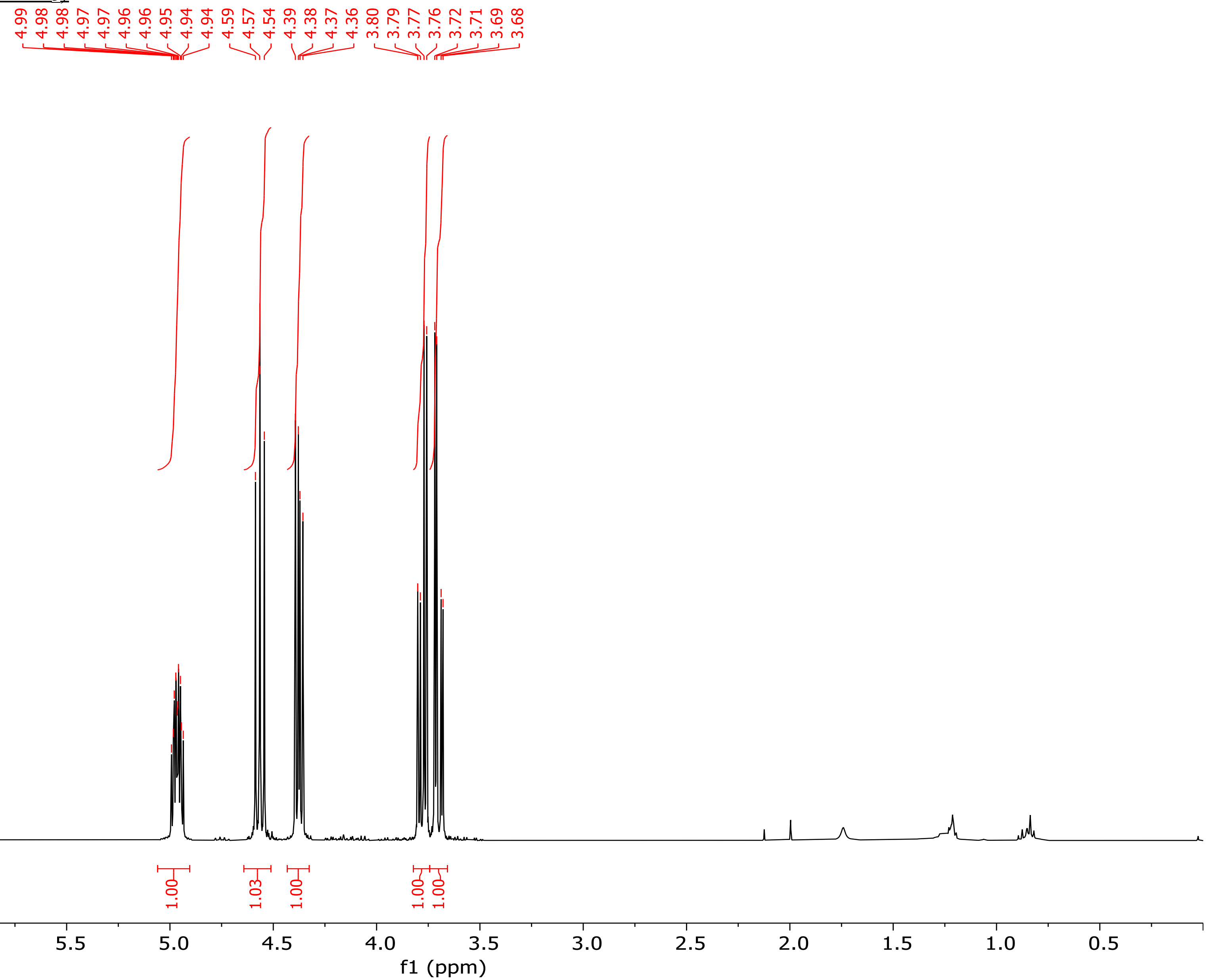

$^{13}C\{^{1}H\}$ NMR Spectrum (100 MHz, $CDCl_3$)

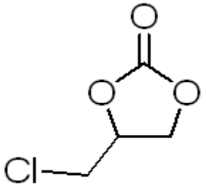

21g

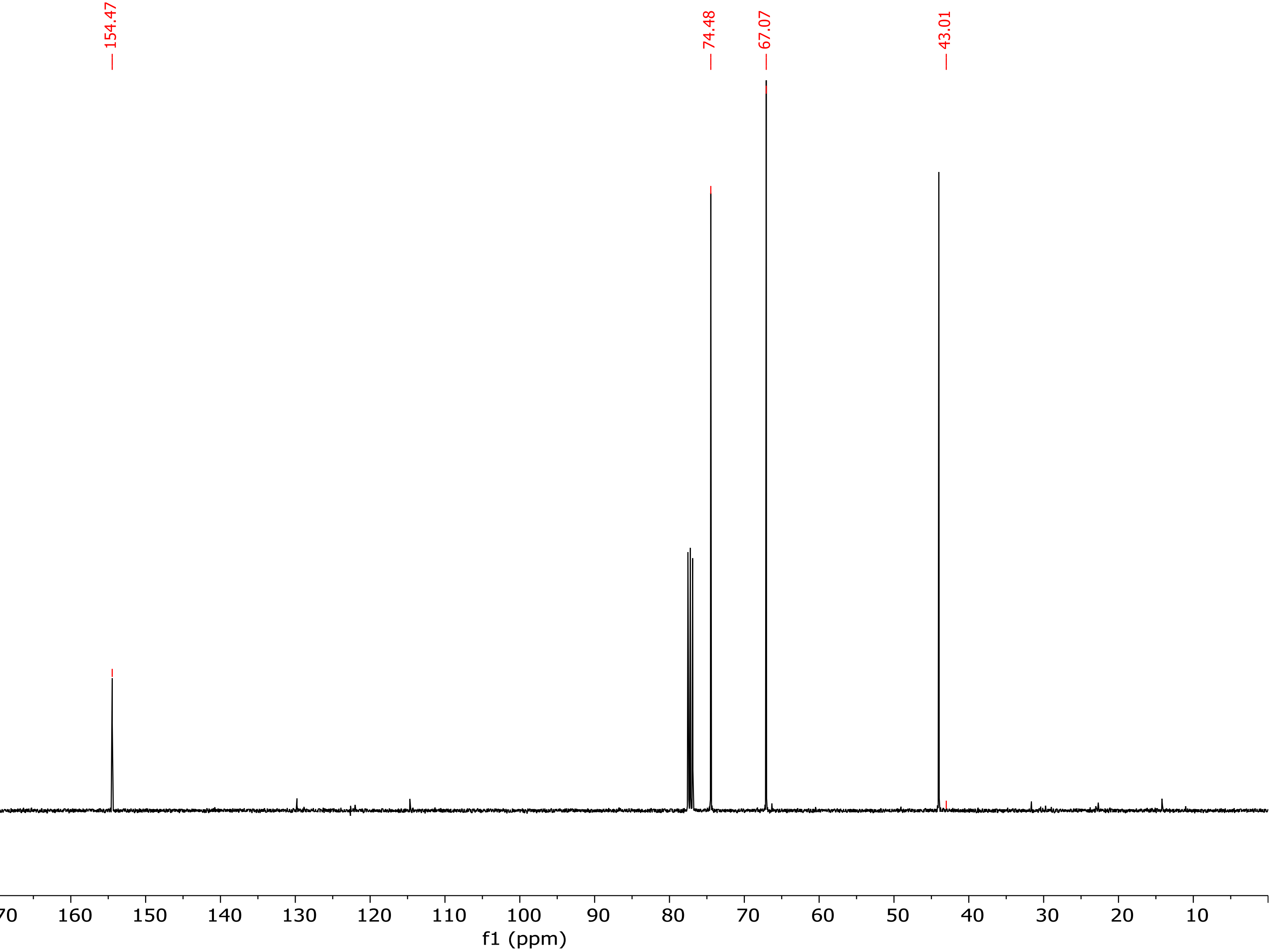

Silica-supported aluminum salophen complex 22a

IR spectrum

Full spectrum

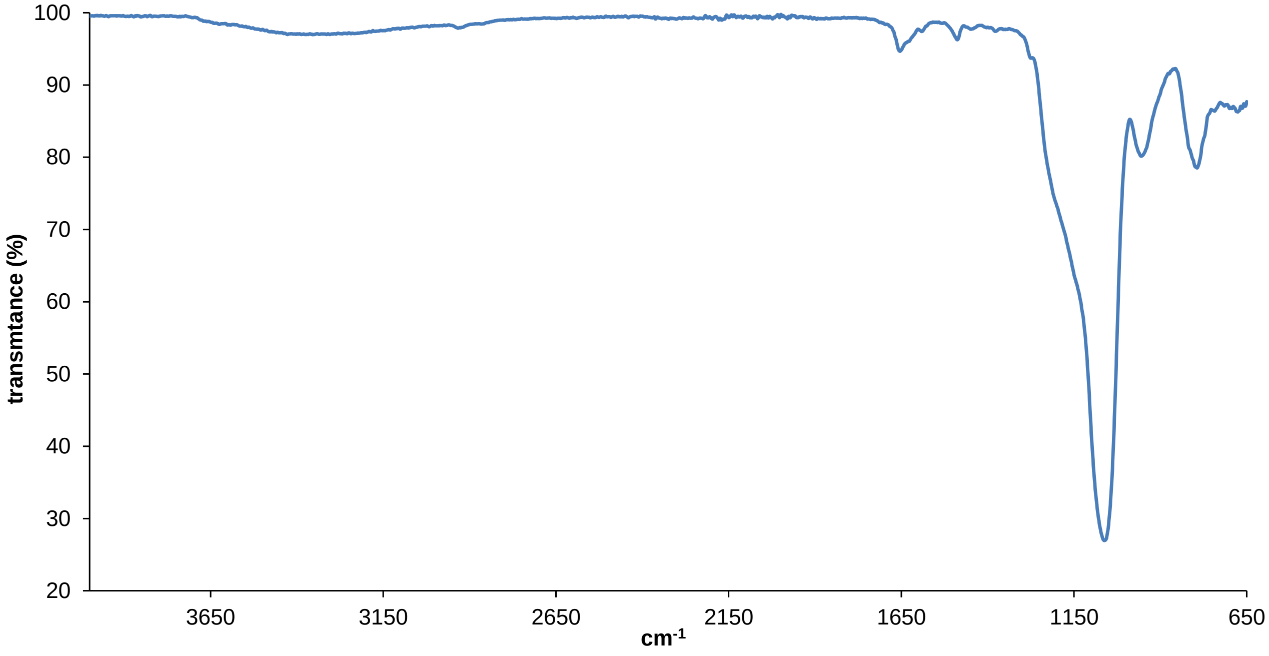

Expansion of 4000-1400 cm^{-1}

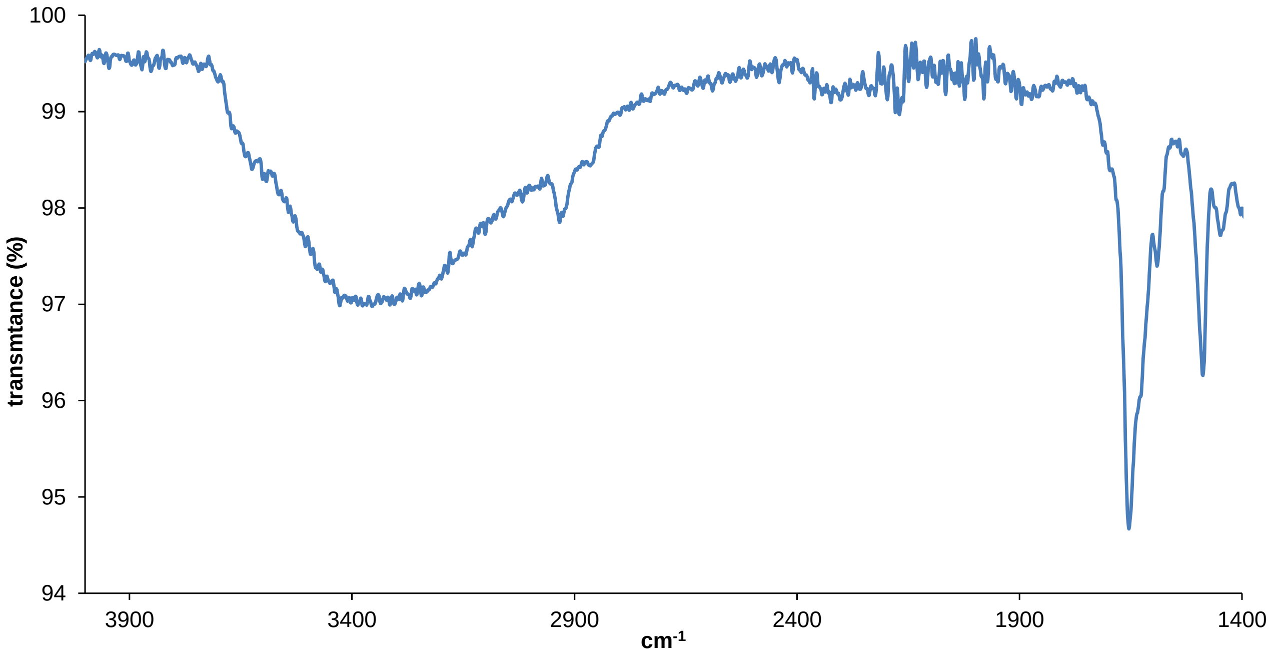

Solid State $^{13}C\{^1H\}$ NMR Spectrum (100 MHz)

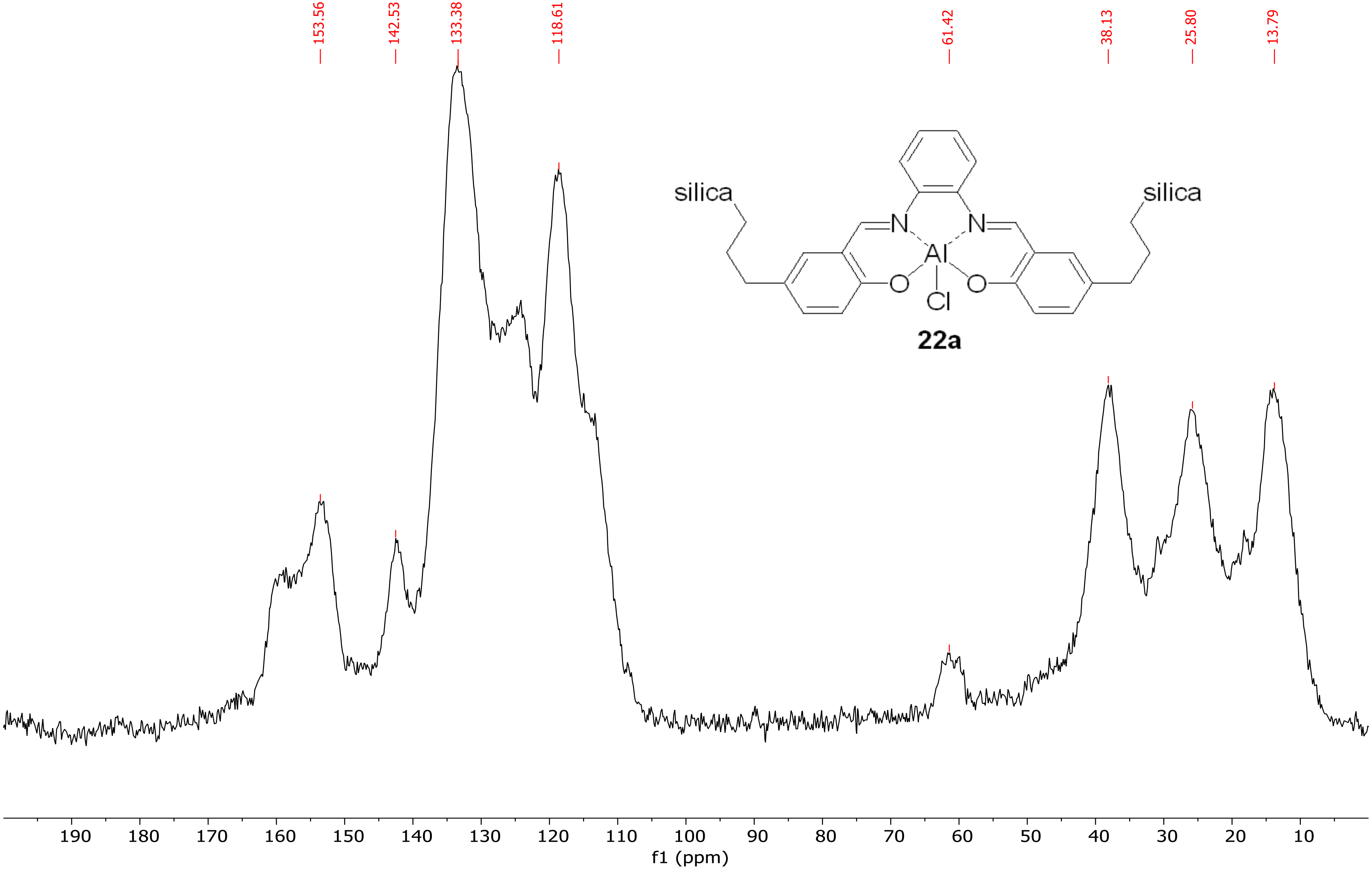

Silica-supported aluminum salophen complex 22b

IR spectrum

Full spectrum

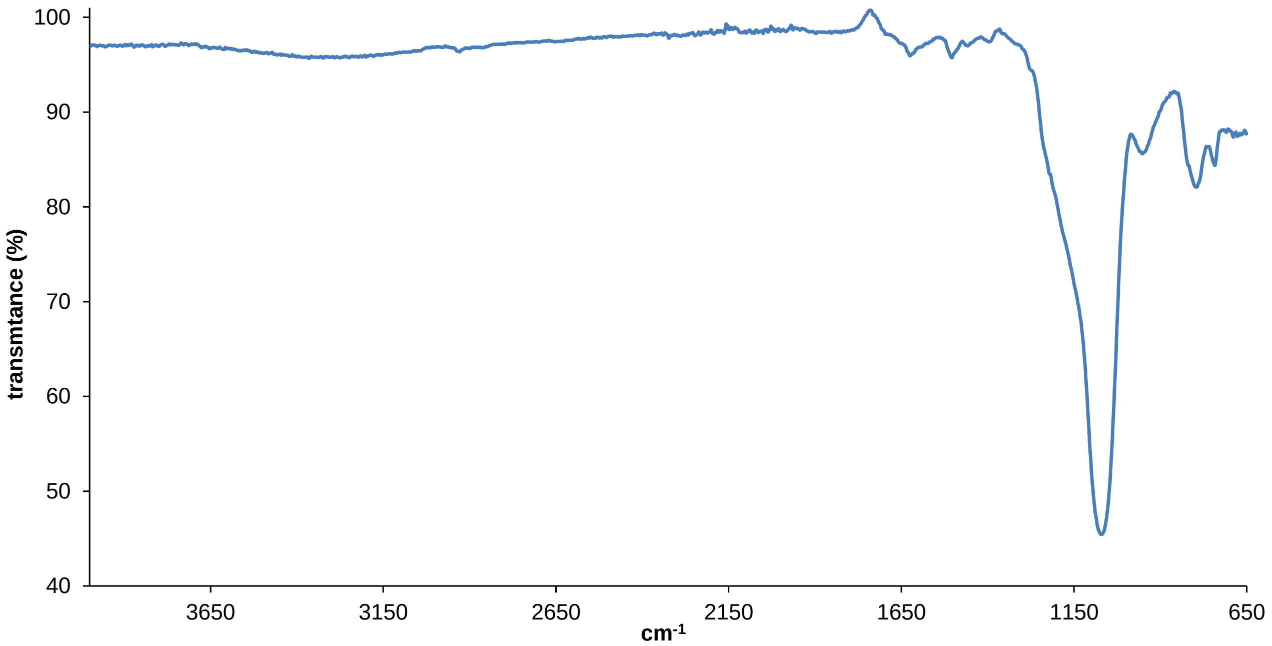

Expansion of 4000-1400 cm^{-1}

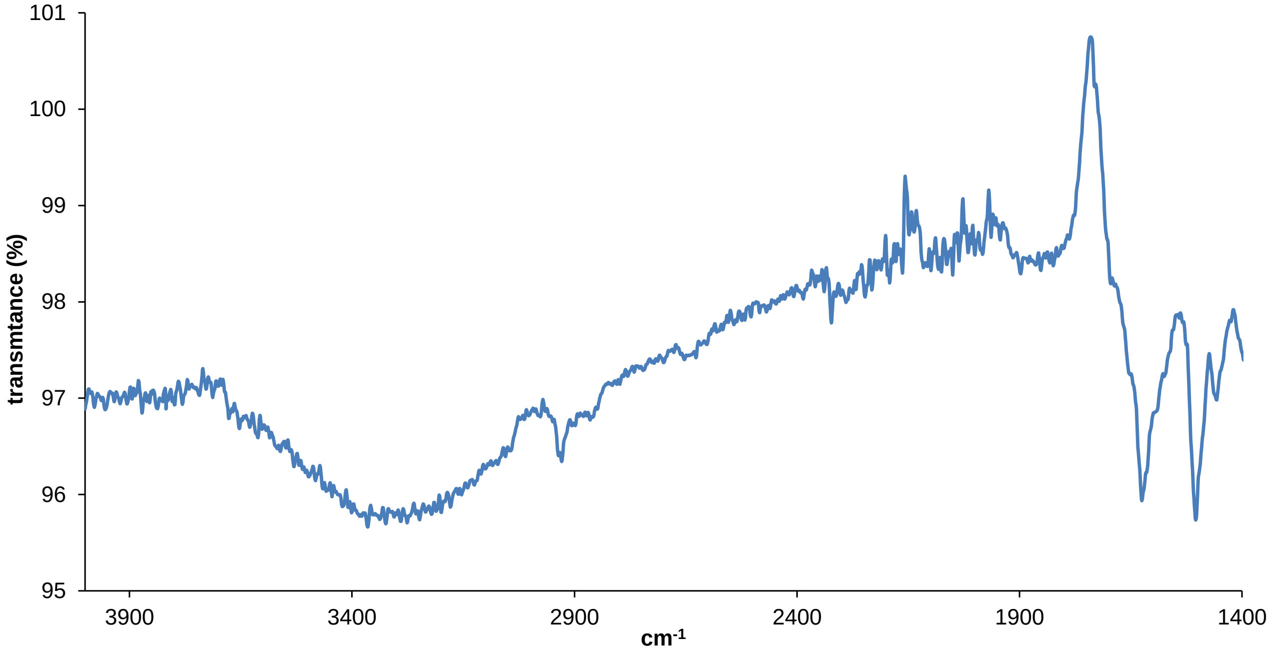

Solid State $^{13}C\{^1H\}$ NMR Spectrum (100 MHz)

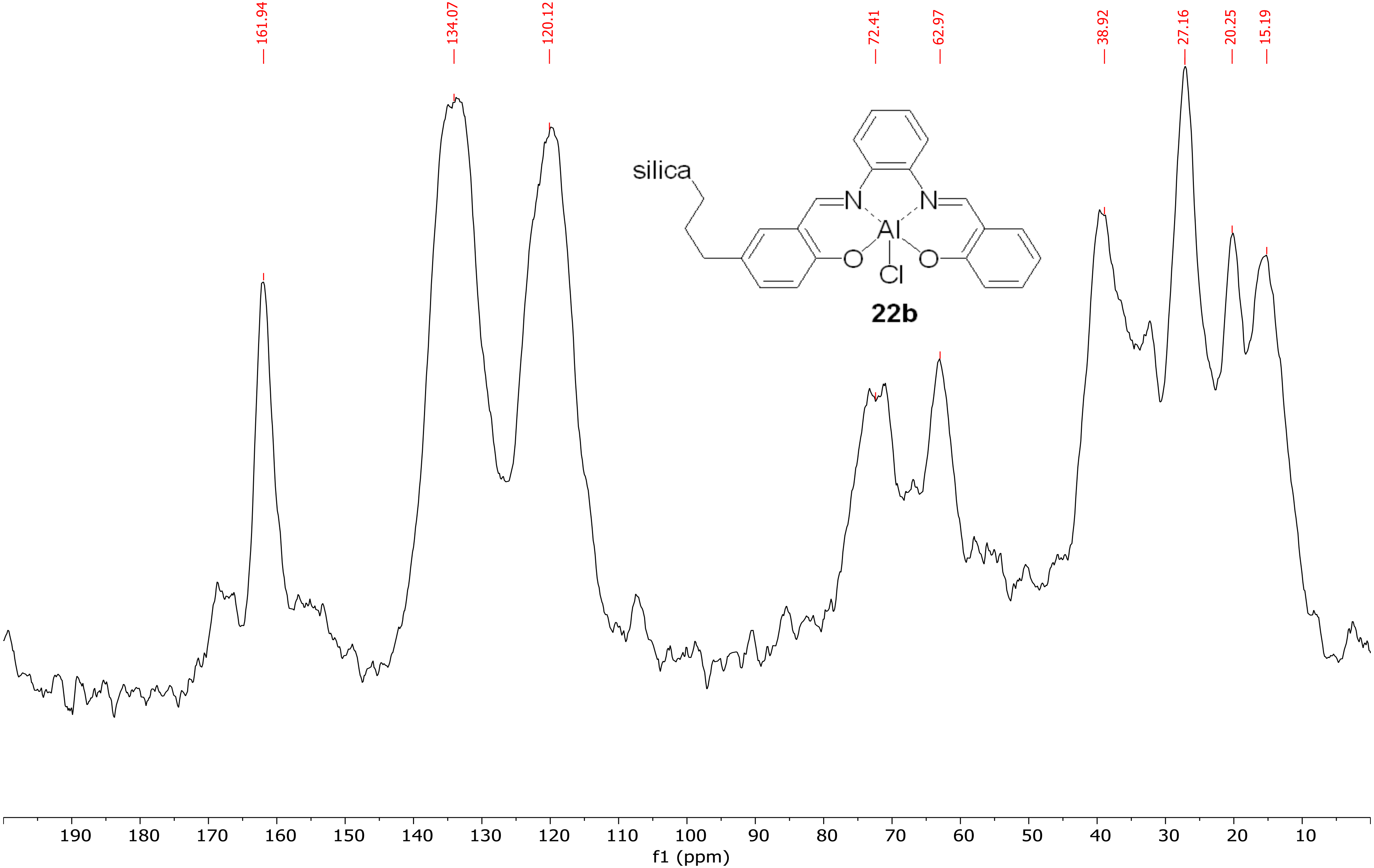

Silica-supported aluminum salen complex 22c

IR spectrum

Full spectrum

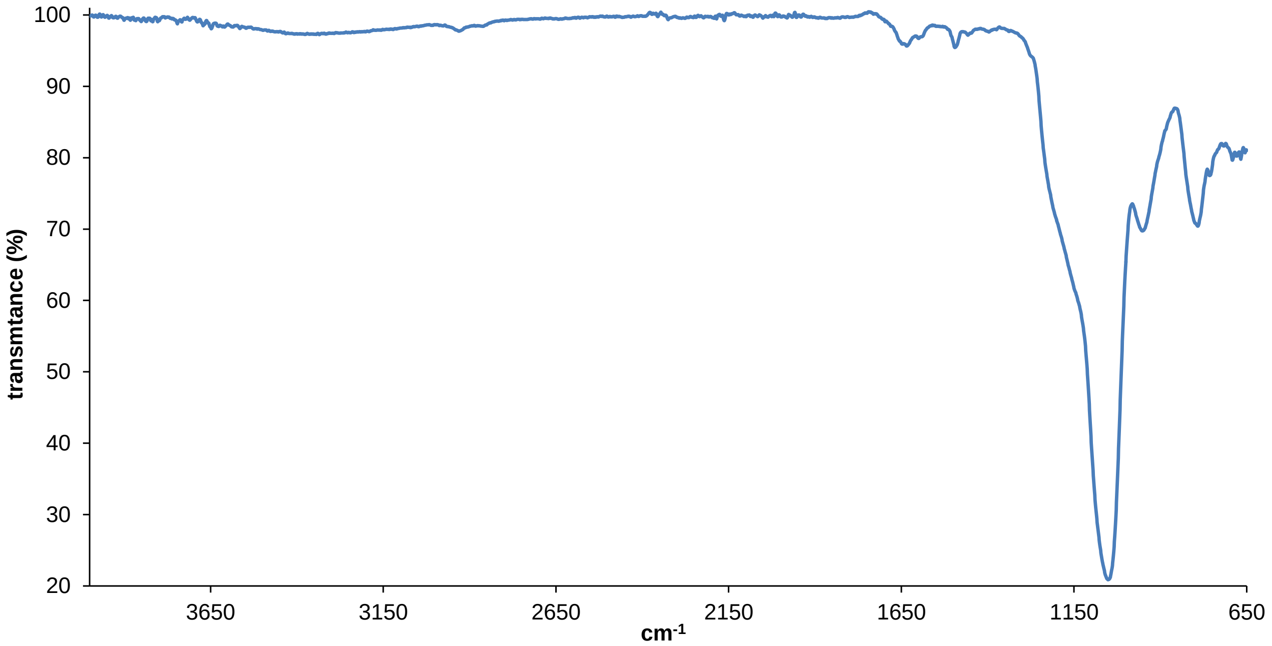

Expansion of 4000-1400 cm^{-1}

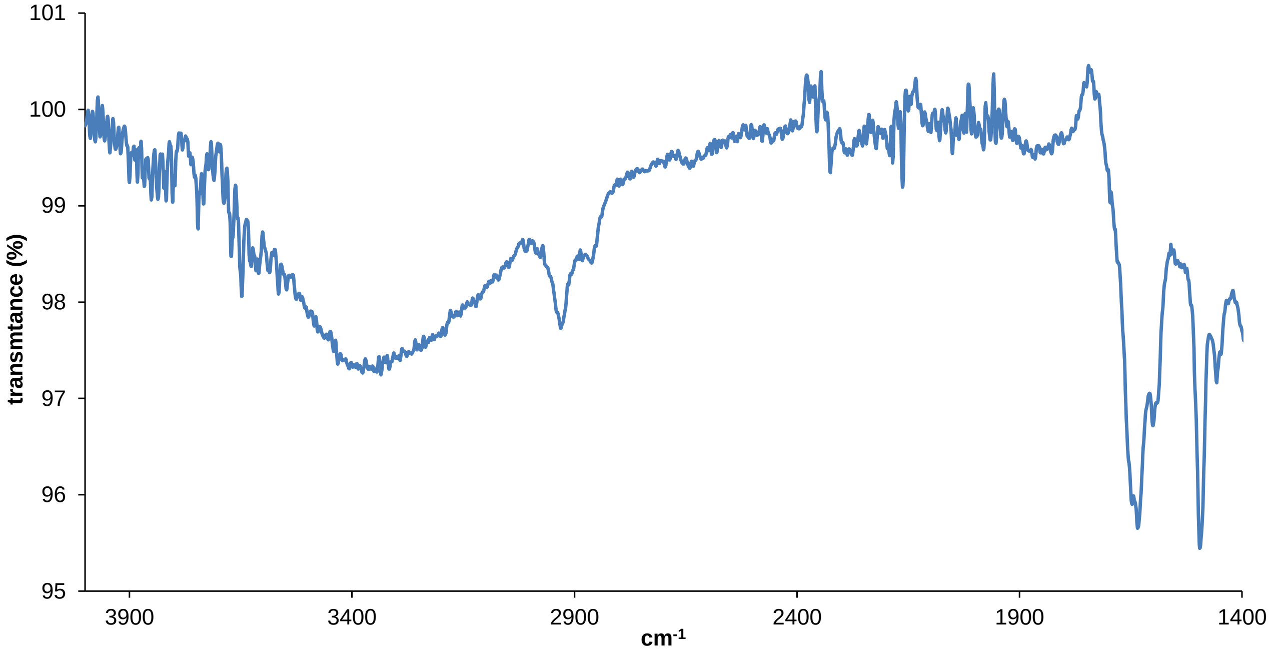

Solid State $^{13}C\{^1H\}$ NMR Spectrum (100 MHz)

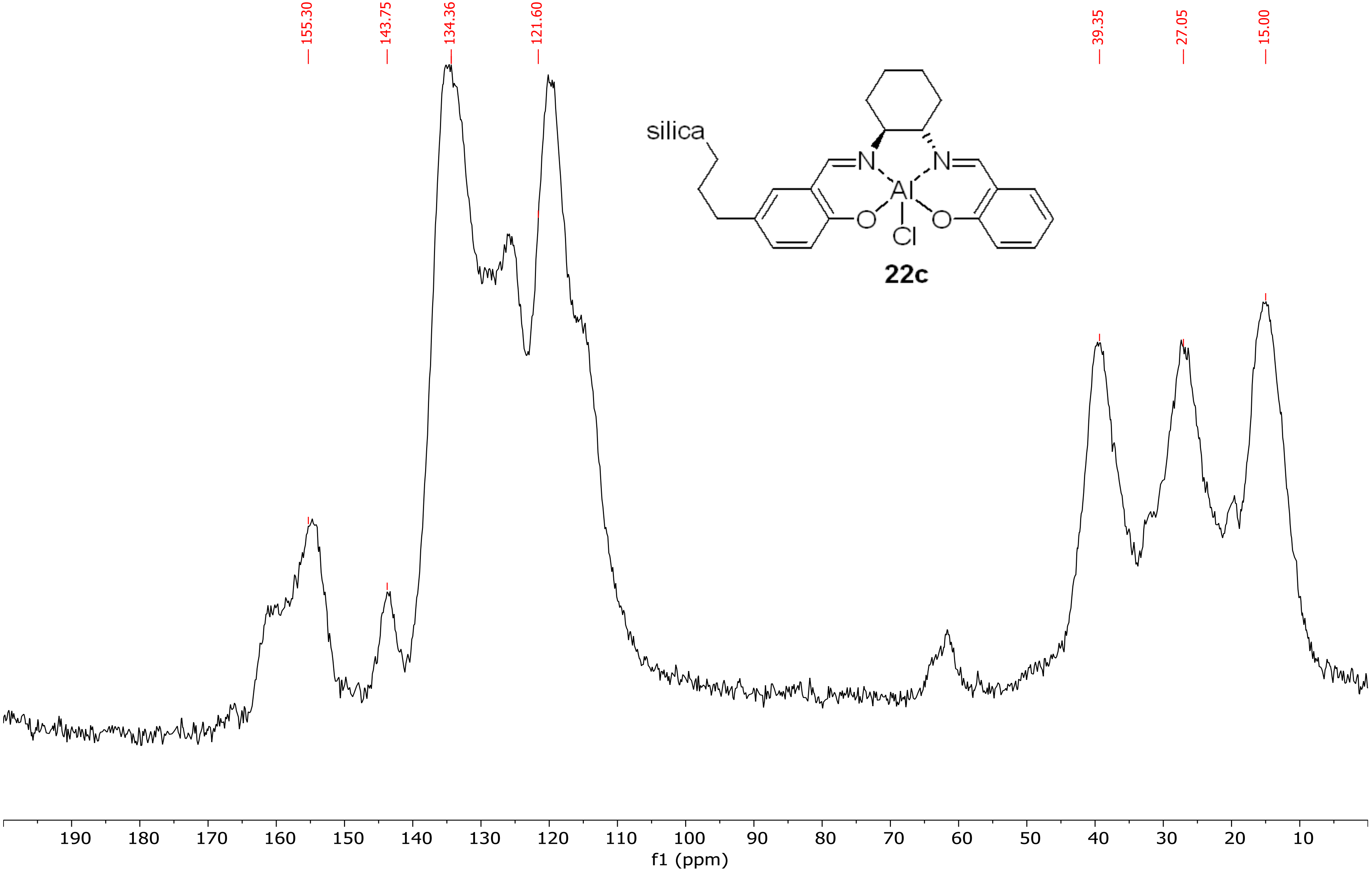

Silica-supported manganese salophen complex 22d

IR spectrum

Full spectrum

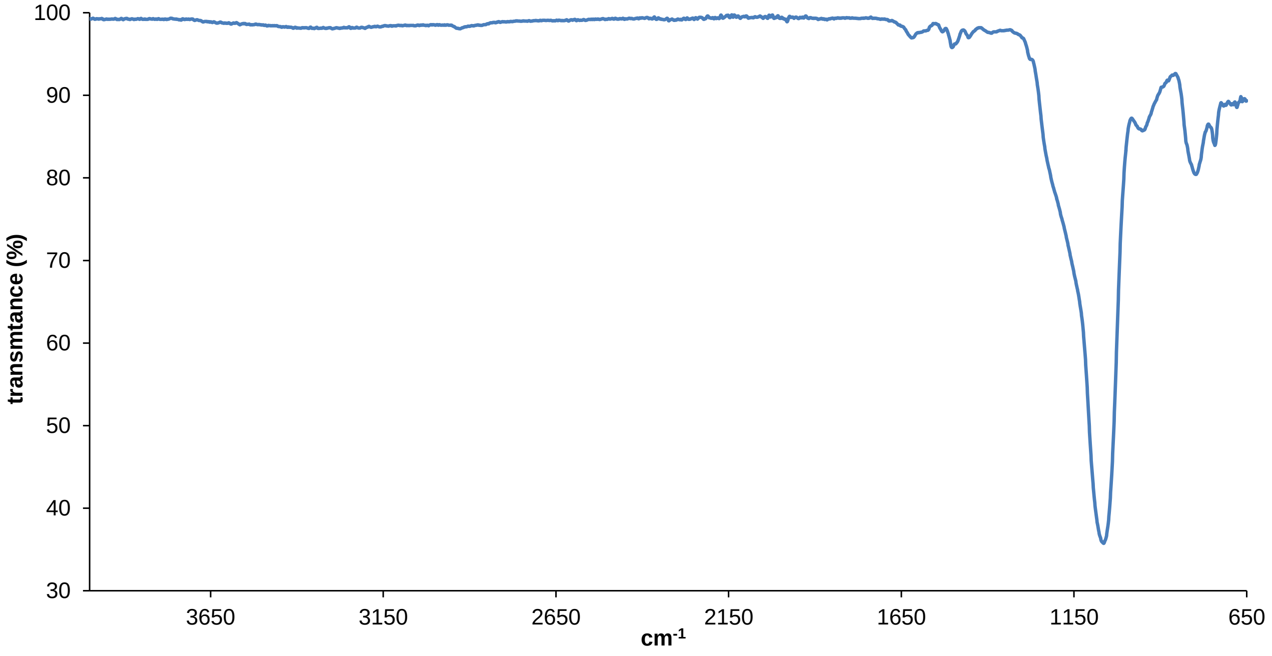

Expansion of 4000-1400 cm^{-1}

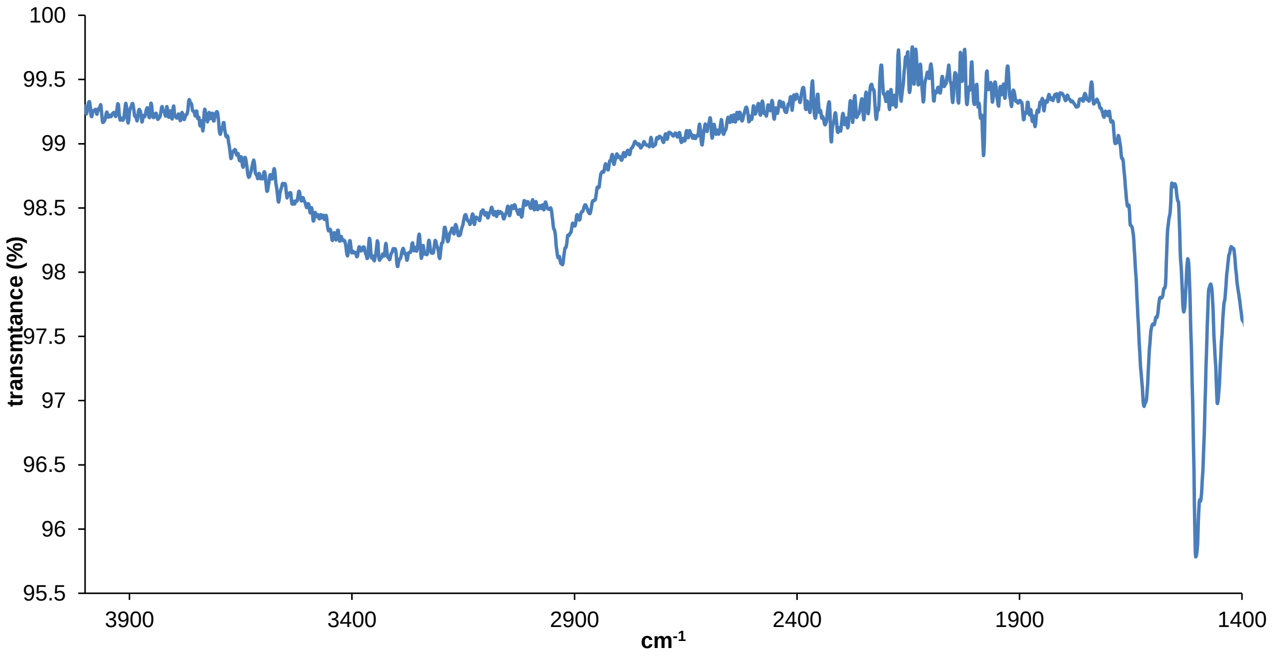

Silica-supported manganese salen complex 22e

IR spectrum

Full spectrum

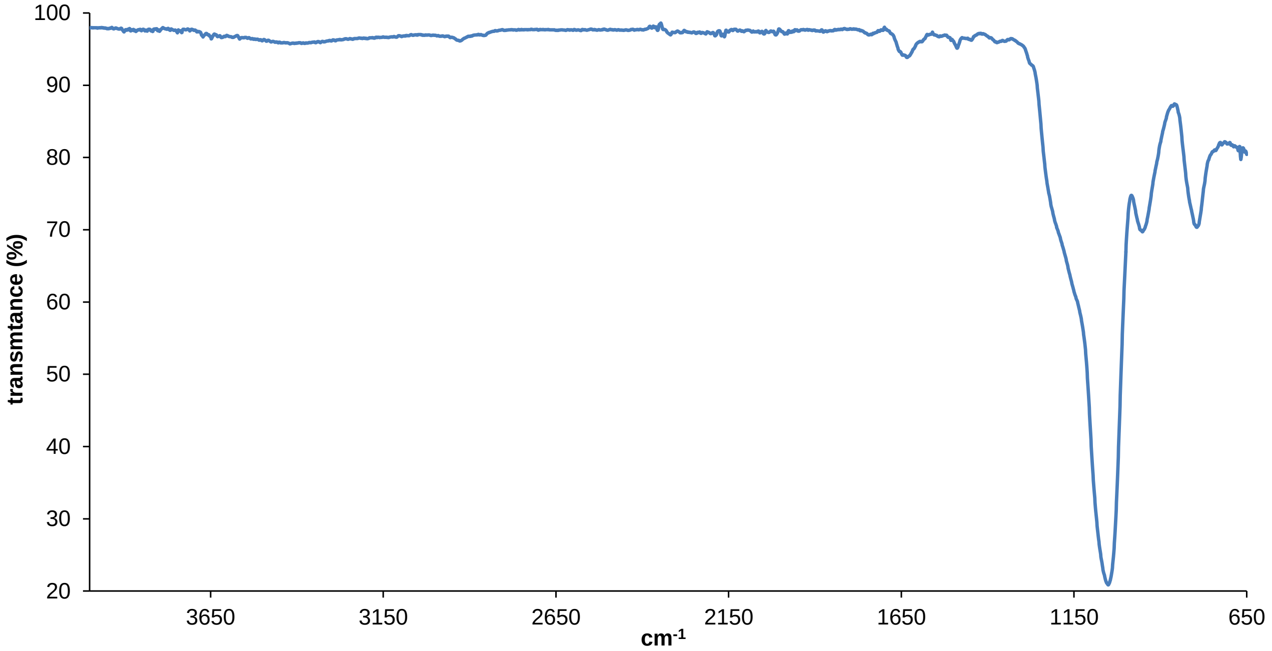

Expansion of 4000-1400 cm^{-1}

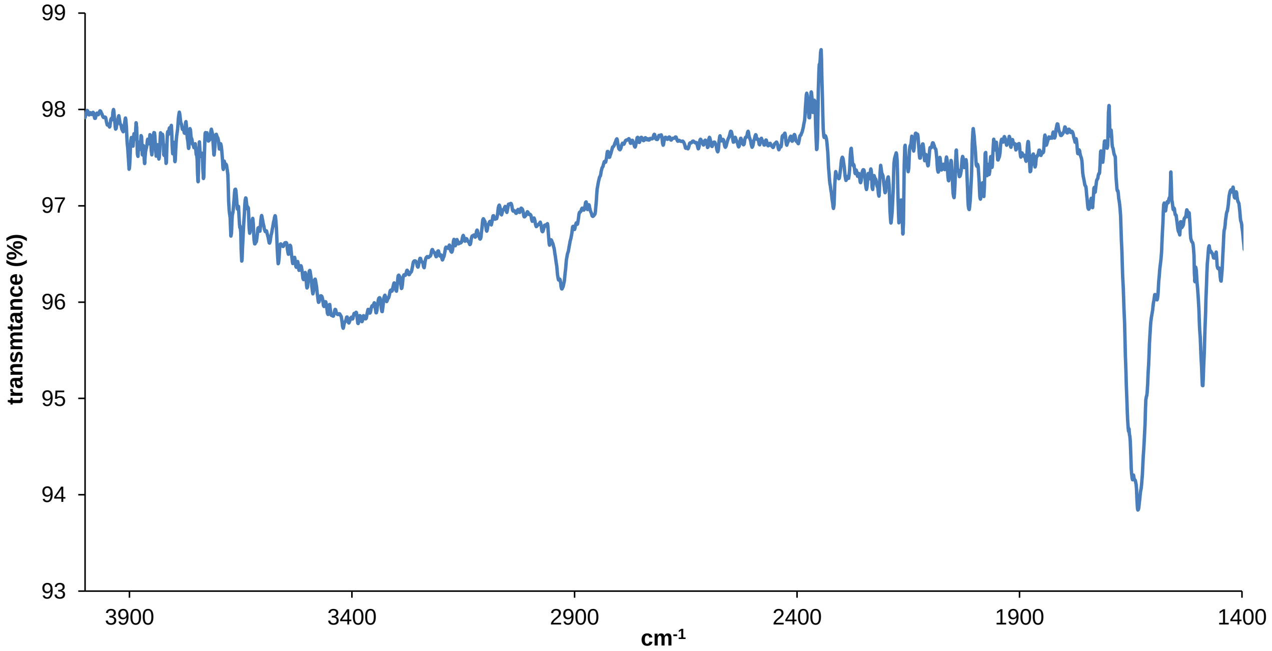

Silica-supported copper salophen complex 22f

IR spectrum

Full spectrum

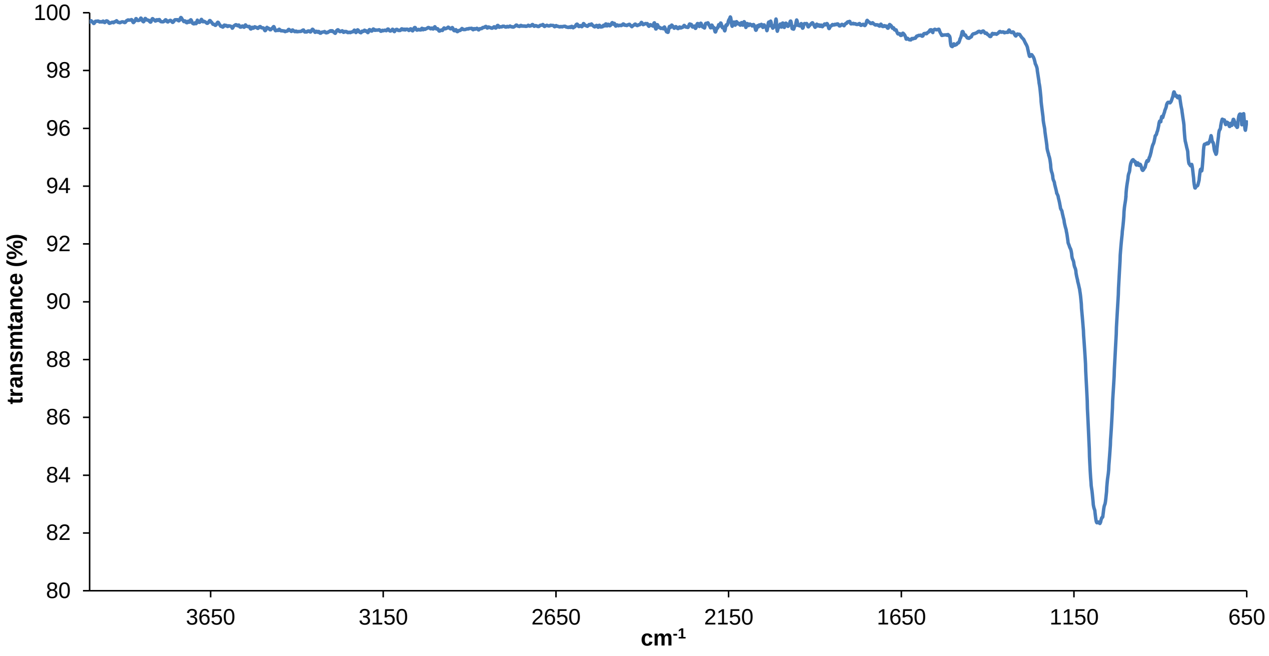

Expansion of 4000-1400 cm^{-1}

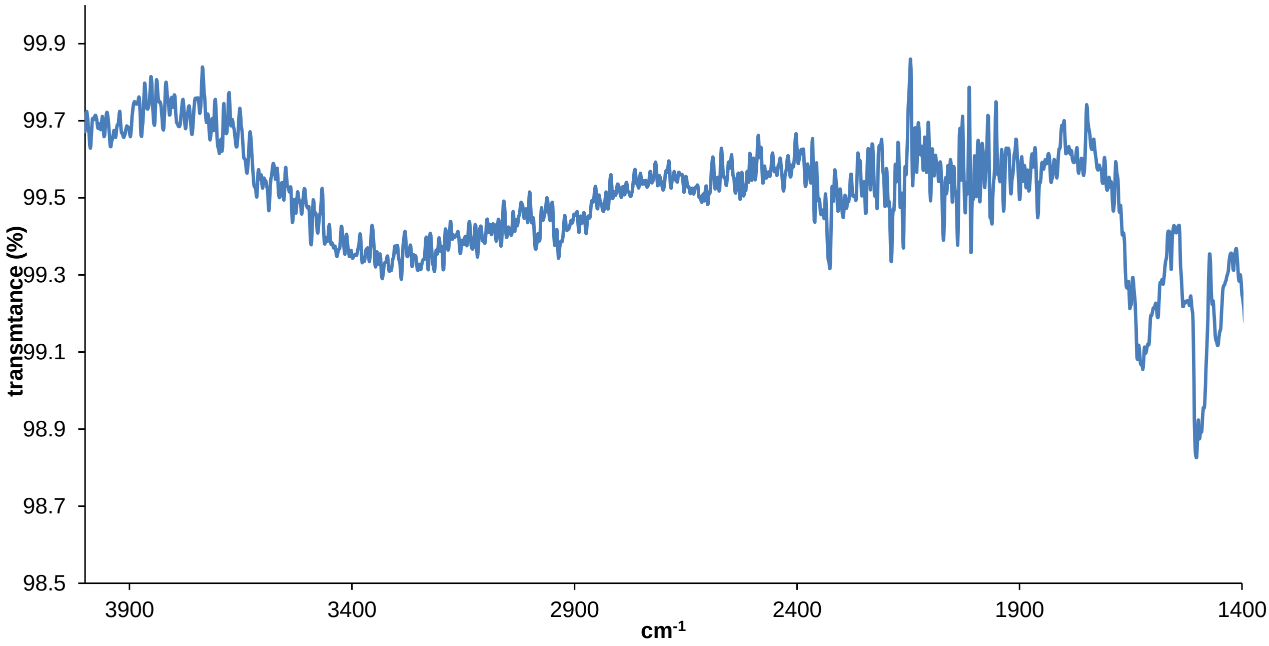

Silica-supported vanadyl salophen complex 22g

IR spectrum

Full spectrum

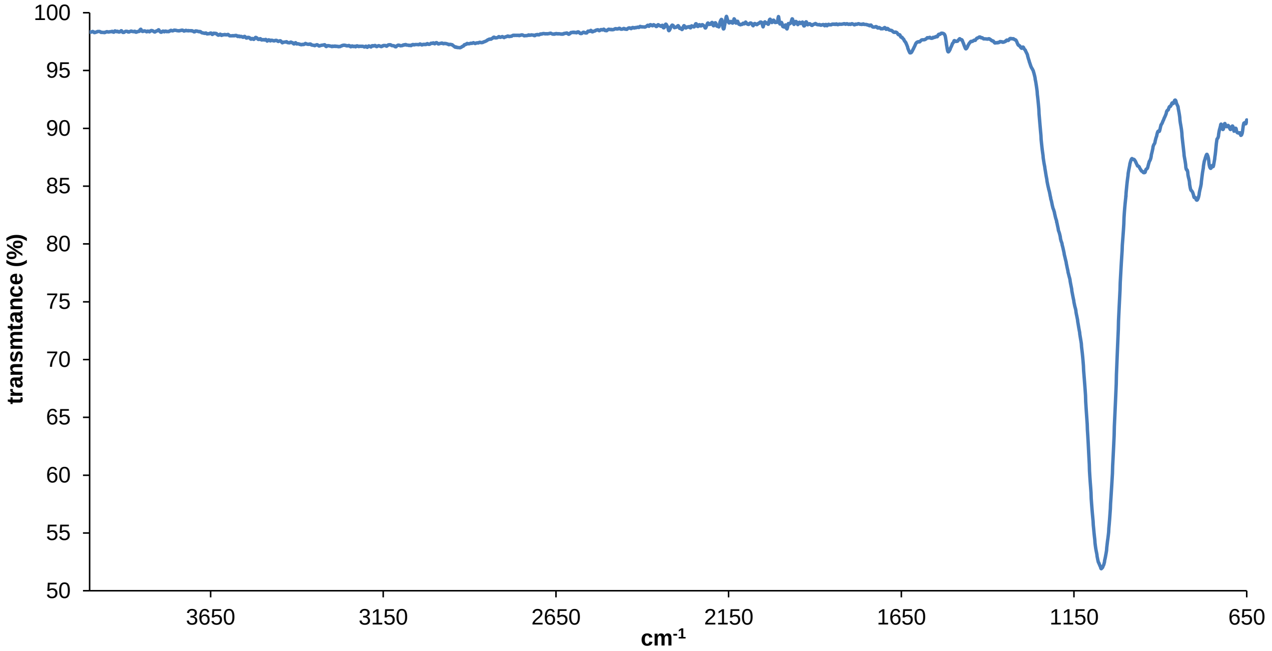

Expansion of 4000-1400 cm^{-1}

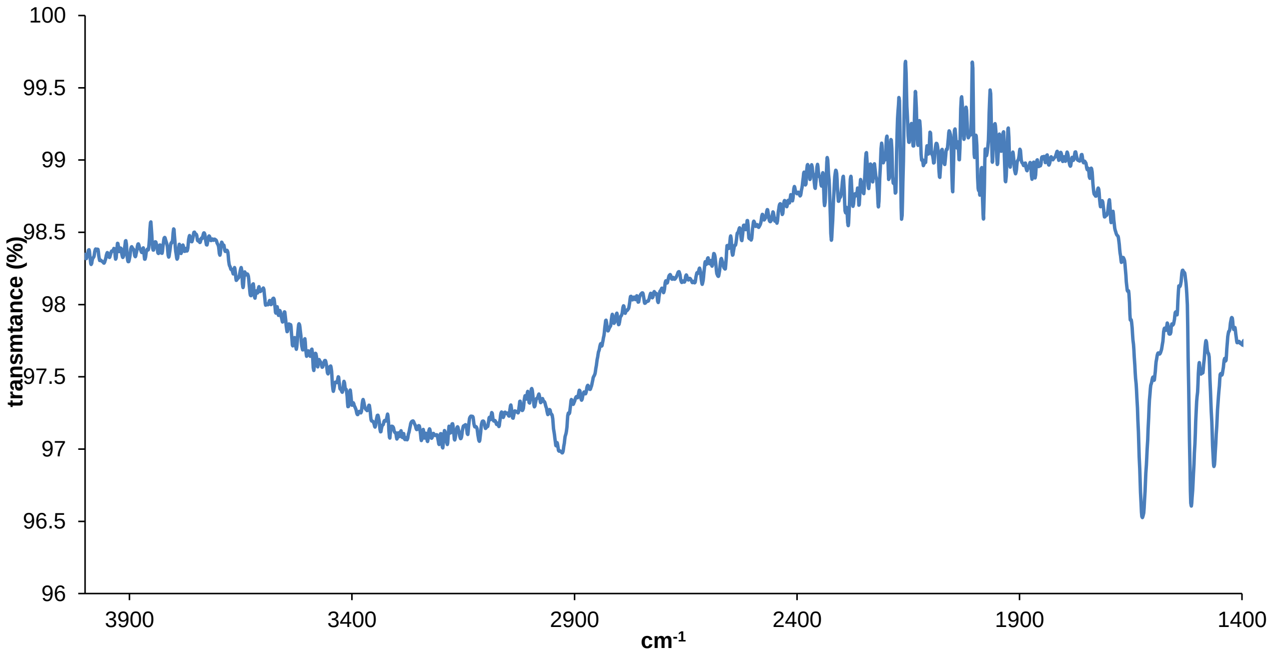

Flow reactor results

A flow reactor capable of utilizing non-volatile epoxides was constructed from stainless steel tubing as shown schematically in Figure S1. Epoxide **20a** was dissolved in ethyl acetate (0.6 g mL^{-1}) to prevent solid cyclic carbonate **21a** or byproducts from blocking the tubing. This solution was then pumped into a tubular reactor (9.5 mm internal diameter and 15 cm long) packed with silica supported catalyst **13a** dispersed with inert silicon carbide to improve the flow through the reactor and to prevent the formation of catalyst hot spots. Simultaneously, a flow regulator was used to supply carbon dioxide with hydrogen as carrier gas to the reactor. The tubular reactor was held within an oven heated to 120 or 140 °C. A back-pressure regulator was used to control the pressure in the flow reactor and non-gaseous products dissolved in the ethyl acetate were collected in a cold trap. Samples were collected every 20 minutes and analyzed by GC coupled with a quadrupole mass-spectrometry detector to determine the conversion of epoxide **20a** into cyclic carbonate **21a** and the selectivity for formation of cyclic carbonate **21a** versus side-products. The reactor was initially heated to 120 °C for 400 minutes and gave the single-pass results shown in Figure S2. During the first 100 minutes, the reactor was equilibrating to steady state conditions and thereafter the selectivity stayed above 90% and reached 100% for the last 100 minutes. The conversion dropped slowly from 26 to 14% during this time. A second run was carried out at 140 °C to see if the higher temperature would increase the conversion, but catalyst deactivation occurred over the first 240 minutes of the reaction so that the conversion dropped from the initial 28% to just 6% (Figure S2). Never the less, the results obtained demonstrated the viability of using silica-supported catalysts **13** in flow chemistry.

Experimental

The reactor was set up as shown in Figure S1 and the tubular reactor was charged with a mixture of catalyst **13a** (0.6 g) and silicon carbide (0.4 g). The reactor tubing was purged with N_2 gas (30 mL min^{-1}) whilst the reactor was heated to either 120 or 140 °C for one hour with the back pressure

regulator set to 6 bar to dry the reactor system. A solution of epoxide **20a** in ethyl acetate (0.6 g mL^{-1}) was used to prime the liquid line and this was then pumped through the reactor at a rate of 0.05 mL min^{-1} whilst simultaneously, the gas supply was switched to a 1:3 molar mixture of CO_2 and H_2 (30 mL min^{-1} total gas flow rate), again with a back pressure of 6 bar. The cold trap was cooled to 0 °C to collect non-volatile products which were analysed off-line by GC-MS every 20 minutes to give the data shown in Figure S2.

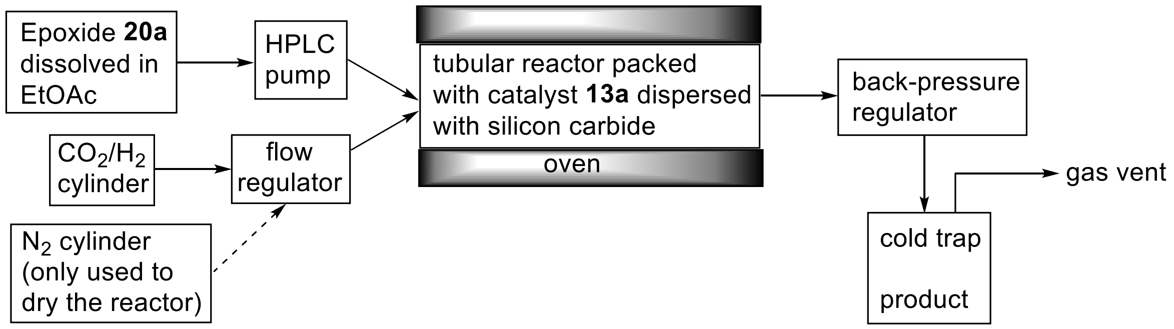

Figure S1. Schematic representation of the flow-reactor for cyclic carbonate synthesis.

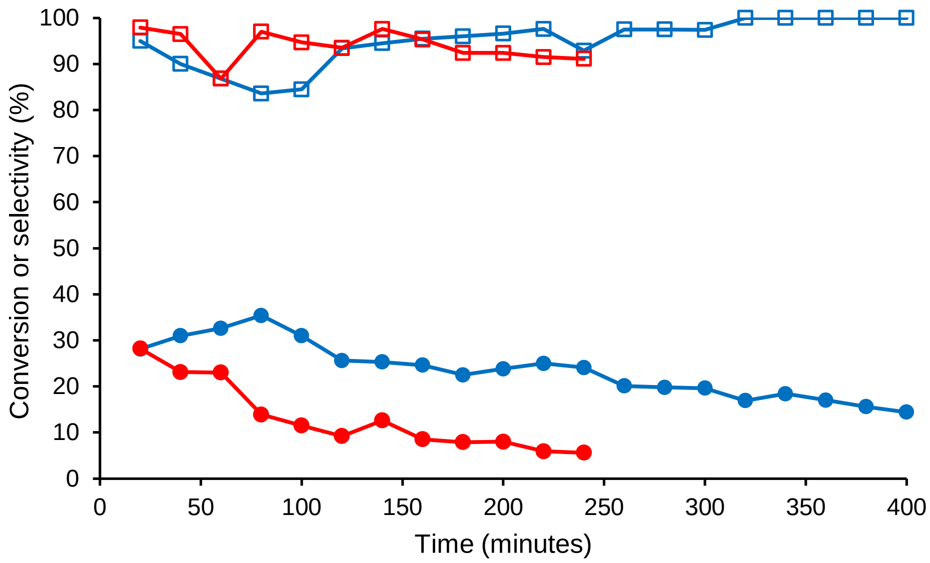

Figure S2. Conversions and selectivities for the formation of cyclic carbonate **21a** in the flow reactor. Blue (120 °C), red (140 °C); filled circles (conversion), open squares (selectivity).
